# Novel Insights Into DLAT's Role in Alzheimer's Disease‐Related Copper Toxicity Through Microglial Exosome Dynamics

**DOI:** 10.1111/cns.70064

**Published:** 2024-10-20

**Authors:** Xiang Ma, Yusheng Sun, Changchun Li, Man Wang, Qijiao Zang, Xuxia Zhang, Feng Wang, Yulan Niu, Jiai Hua

**Affiliations:** ^1^ Laboratory of Biochemistry and Pharmacy Taiyuan Institute of Technology Taiyuan P. R. China; ^2^ Department of Chemistry and Chemical Engineering Taiyuan Institute of Technology Taiyuan P. R. China

**Keywords:** 5xFAD transgenic model, exosome‐mediated signaling, gene expression profiling in AD, neurodegeneration mechanisms, neuronal copper toxicity, therapeutic target discovery

## Abstract

**Background:**

Alzheimer's disease (AD) is a complex neurodegenerative disorder, with recent research emphasizing the roles of microglia and their secreted extracellular vesicles in AD pathology. However, the involvement of specific molecular pathways contributing to neuronal death in the context of copper toxicity remains largely unexplored.

**Objective:**

This study investigates the interaction between pyruvate kinase M2 (PKM2) and dihydrolipoamide S‐acetyltransferase (DLAT), particularly focusing on copper‐induced neuronal death in Alzheimer's disease.

**Methods:**

Gene expression datasets were analyzed to identify key factors involved in AD‐related copper toxicity. The role of DLAT was validated using 5xFAD transgenic mice, while in vitro experiments were conducted to assess the impact of microglial exosomes on neuronal PKM2 transfer and DLAT expression. The effects of inhibiting the PKM2 transfer via microglial exosomes on DLAT expression and copper‐induced neuronal death were also evaluated.

**Results:**

DLAT was identified as a critical factor in the pathology of AD, particularly in copper toxicity. In 5xFAD mice, increased DLAT expression was linked to hippocampal damage and cognitive decline. In vitro, microglial exosomes were shown to facilitate the transfer of PKM2 to neurons, leading to upregulation of DLAT expression and increased copper‐induced neuronal death. Inhibition of PKM2 transfer via exosomes resulted in a significant reduction in DLAT expression, mitigating neuronal death and slowing AD progression.

**Conclusion:**

This study uncovers a novel pathway involving microglial exosomes and the PKM2‐DLAT interaction in copper‐induced neuronal death, providing potential therapeutic targets for Alzheimer's disease. Blocking PKM2 transfer could offer new strategies for reducing neuronal damage and slowing disease progression in AD.

## Graphical abstract

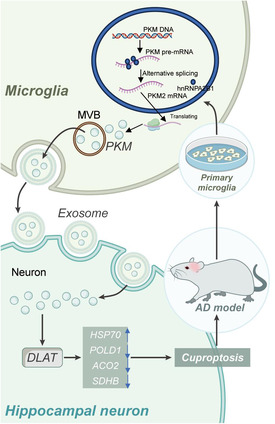

## 1 Introduction

Alzheimer's disease (AD), a prevalent neurodegenerative ailment, manifests as a progressive decline in memory and cognitive capabilities, particularly in the aging population globally [1]. Recently, an emergent research paradigm has spotlighted the potential link between imbalanced copper ion homeostasis in neurons and AD progression. Accumulated evidence suggests that an overabundance of copper ions might instigate neuronal oxidative stress, culminating in a phenomenon termed neuronal copper death [2, 3, 4, 5]. Nevertheless, the intricate molecular pathways that precipitate this neuronal copper dyshomeostasis remain shrouded in ambiguity [6].

Microglia, the predominant glial cells within the brain, undertake pivotal roles encompassing immune response modulation, neuroprotection, and homeostasis maintenance. Their neuroprotective capabilities become particularly evident post neuronal damage or injury [7, 8, 9]. Emerging literature postulates that microglia, another type of glial cell, possess the ability to alter neighboring cell functions by secreting extracellular vesicles [10, 11]. Intriguingly, during the course of AD, these vesicles could act as mediators, allowing microglial cells to modulate neuronal functionalities, although the granular mechanisms warrant deeper exploration [12].

The tricarboxylic acid cycle serves as a crucial core component of organismal energy metabolism, necessitating the catalytic conversion of glucose, fatty acids, and amino acids into NADH, FADH, which in turn generate ATP through the electron transport chain (ETC). Many of these enzymatic reactions are closely associated with sulfur‐transferase proteins. In mammals, there are primarily four sulfur‐transferase‐modified proteins: DBT (component of the branched‐chain α‐keto acid dehydrogenase complex), GCSH (glycine cleavage system protein H), DLST (component of α‐ketoglutarate dehydrogenase complex), and DLAT (component of pyruvate dehydrogenase complex) [13]. Among these, DLAT is a crucial component of the pyruvate dehydrogenase complex in the tricarboxylic acid cycle, converting pyruvate to acetyl‐CoA, which participates in the tricarboxylic acid cycle [14]. Tsvetkov et al. found that sulfur‐containing proteins readily bind to cuprous ions. During copper overload, copper ions can directly bind to the esterified proteins within the tricarboxylic acid cycle, leading to oligomerization of DLAT induced by the FDX1 (Ferredoxin 1)/LIAS (lipoic acid synthase)‐mediated lipoic acid (LA) pathway (sulfur pathway), resulting in metabolic abnormalities in the tricarboxylic acid cycle, triggering cellular protein toxicity stress and ultimately cell death [15]. Recent studies have shown the upregulation of DLAT expression in brain samples of AD cases, suggesting its potential involvement in the pathogenesis of AD [16]. However, whether the upregulation of DLAT expression in AD affects its binding with copper ions, leading to neuronal copper death and consequently AD occurrence, remains unclear.

Furthermore, PKM2, a cardinal glycolytic enzyme, stands at the crossroads of cellular energy metabolism [17]. Recent scientific endeavors hint at PKM2's capability to modulate DLAT expression and activity, thereby potentially affecting neuronal copper equilibrium [18]. Building upon this premise, we postulate that microglial cells might employ extracellular vesicles as conduits to transmit PKM2, subsequently influencing DLAT expression. This cascade might destabilize neuronal copper balance, culminating in neuronal copper death. Nevertheless, this hypothesis requires rigorous experimental validation.

In this endeavor, we set forth to delineate the intricate mechanisms underpinning PKM2's influence on DLAT‐mediated neuronal death in AD, emphasizing the role of microglia‐derived extracellular vesicles. Harnessing the prowess of high‐throughput sequencing coupled with advanced bioinformatics, we aim to pinpoint genes manifesting differential expression patterns in AD. Validating these gene signatures using a mouse model will potentially unveil novel insights into AD pathogenesis, paving the way for innovative diagnostic and therapeutic stratagems.

## 2 Materials and Methods

### 2.1 Transcriptome Data and snRNA‐Seq Acquisition

Gene expression profiles relevant to AD were downloaded from the Gene Expression Omnibus (GEO), specifically datasets GSE97760 and GSE163857. These datasets include peripheral blood samples from late‐stage AD patients and control groups, along with samples from AD model mice. Differential gene expression analysis was performed using the limma and edgeR packages in R, adhering to thresholds of |logFC| > 0.5 and *p*‐value < 0.05. Data visualization tools such as ggplot2 and heatmap facilitated the generation of volcano plots and heatmaps, respectively [19].

Furthermore, the AD‐related single‐nucleus RNA sequencing (snRNA‐seq) dataset GSE140510 was obtained from the GEO database, from which three samples of WT group and three samples of AD group brain tissue single‐nucleus samples were selected.

### 2.2 Gene Differential Expression Analysis

Differential gene expression analysis was conducted by using the “limma” package in R to screen for differentially expressed genes in dataset GSE97760 and by utilizing the “edgeR” package for DEG selection in the high‐throughput sequencing dataset GSE163857. The selection criteria were set at |logFC| > 0.5 and *p*‐value < 0.05. Subsequently, volcano plots and gene differential expression boxplots were generated using the “ggplot2” package in R, and a heatmap of gene expression differences was created using the “heatmap” package [19].

### 2.3 PPI Network Construction and Gene Ontology (GO)/Kyoto Encyclopedia of Genes and Genomes (KEGG) Enrichment Analysis

The intersection of differentially expressed genes linked to copper toxicity from datasets and extracellular vesicle proteins listed in the ExoCarta database was analyzed. Using the STRING database and Cytoscape software, a PPI network was constructed and visualized. Concurrently, GO and KEGG enrichment analyses were performed using the KOBAS database to identify significant pathways [20].

### 2.4 Lasso Regression and SVM‐RFE Analysis Are Used to Select Disease‐Related Feature Genes

Two machine learning algorithms, Support Vector Machine Recursive Feature Elimination (SVM‐RFE) and Least Absolute Shrinkage and Selection Operator (LASSO), were used to screen key copper death‐related disease feature genes. SVM‐RFE is a widely used supervised machine learning protocol that could be used for classification and regression, utilizing the “e1071” package in the R programming language for analysis. The SVM‐RFE algorithm is used to identify genes with higher discriminative power. LASSO is performed using the “glmnet” package in R, representing a regression analysis algorithm that applies regularization to variable selection. Taking the intersection of genes recognized by two algorithms, key genes related to copper death‐associated diseases were selected [21, 22].

### 2.5 snRNA‐Seq Analysis

snRNA‐seq data analysis was performed using the “Seurat” package (version 3.1) in R (https://github.com/satijalab/seurat). Data quality control was conducted based on criteria of 200 < nFeature_RNA < 5000 and percent.mt < 20, followed by the selection of the top 2000 highly variable genes based on variance [23]. To reduce the dimensionality of the snRNA‐seq dataset, principal component analysis (PCA) was applied to the highly variable genes. The top 20 principal components were selected for downstream analysis using the Elbowplot function in the Seurat package. Cell subgroups were identified using the FindClusters function in Seurat with a default resolution set at res = 1. Subsequently, the UMAP algorithm was utilized for nonlinear dimensionality reduction of the scRNA‐seq sequencing data. Cell annotation was performed by integrating known lineage‐specific marker genes and leveraging the online platform CellMarker (http://xteam.xbio.top/CellMarker/) for annotation [24]. Cell communication analysis was conducted using the “CellChat” package in R (https://github.com/sqjin/CellChat).

### 2.6 Constructing AD Transgenic Mouse Models

Three‐month‐old male 5xFAD transgenic mice (C57BL6 background) were purchased from the RAMP model (LM000176). C57BL6 mice (213, Vital River, Beijing, China) were used as the wild‐type (WT) control group. The animals were housed in SPF‐grade animal rooms for 1 week, with constant humidity (45%–50%) and temperature (25°C–27°C). They were exposed to 12 h of light and 12 h of darkness each day to adapt to the experimental environment. The Ethics Committee has approved all animal experimental procedures for animal experiments in our institute.

The male mice of WT and 5xFAD were divided into the following seven groups (Figure S1): WT group; 5xFAD group (raised until 6 months of age); 5xFAD + sh‐NC group, injection of sh‐NC lentivirus; 5xFAD + sh‐DLAT group, injection of DLAT knockdown lentivirus; 5xFAD + M‐exo‐sh‐NC + oe‐NC group, injection of M‐exo‐sh‐NC exosomes and oe‐NC lentivirus; 5xFAD + M‐exo‐sh‐PKM + oe‐NC group, injection of M‐exo‐sh‐PKM exosomes and oe‐NC lentivirus; and 5xFAD + M‐exo‐sh‐PKM + oe‐DLAT group, injection of M‐exo‐sh‐PKM exosomes and DLAT overexpression lentivirus. The lentivirus overexpression system utilizes lentiviral vectors (LV4, Genewiz, Shanghai, China) and interference vectors (pGLVU6/GFP, C06001, Genewiz, Shanghai, China) for construction. sh‐DLAT (Target sequence: CCATTGCTAGTGATGTTGTTT) and sh‐NC (CTCGCTTGGGCGAGAGTAA) [25]. The lentivirus was purchased from Sigma‐Aldrich (USA) and titrated to 10^9^ TU/mL. Dissolve the virus in 5 μL of sterile PBS.

At 4 months of age, mice were anesthetized with isoflurane using the SomnoSuite low‐flow anesthesia system (Kent Scientific Corporation, Torrington, CT, USA) and then injected with a slow virus at the following coordinates using a stereotaxic apparatus with a special adapter (SR‐5R, Narishige Scientific Instrument Laboratory, Japan): AP = −2.5 mm, L = 1.5 mm, DV = 1.7 mm. Each group of exosomes (100 μg) was resuspended in 20 μL of PBS, and then 2 μL of the exosome suspension was injected stereotaxically into the brain of 6‐month‐old mice, using the same coordinates as before [26]. Three weeks after injection, the anesthetized mice were sacrificed, and hippocampal tissues were collected for subsequent analysis [27, 28, 29, 30, 31].

### 2.7 Morris Water Maze Experiment (MWM)

The Morris Water Maze (MWM) was used to evaluate learning and memory function in mice. The device consisted of a circular steel water tank with a diameter of 122 cm and a height of 60 cm. Inside the tank, there was a water platform positioned 1 cm above the water surface. The platform had a diameter of 10 cm and a depth of 30 cm. Surrounding the tank, there was a blue backdrop and a pool cue. The entire setup was placed in an isolated room with an ambient temperature of 20°C and humidity of 60%. The water temperature was maintained at 21°C. To make the water opaque, titanium dioxide was added.

During the positioning trial phase, which spanned the first 4 days of the experiment (P40‐P43), mice were placed in the water from four different quadrants—south, north, west, and east. The escape latency was recorded as the duration taken by the mouse to find the platform and stay on it for three consecutive seconds within a time limit of 90 s. If a mouse failed to find the platform within 90 s, it was guided to the platform and allowed to stay there for 15 s, during which the latency time was recorded as 90 s. Following the positioning trial phase, a space exploration experiment was conducted on the fifth day. During this phase, the platform was removed, and each mouse was released from the first quadrant into the water for 90 s of free exploration. All activities were recorded, including the time spent in each quadrant, the time spent crossing the platform, and the swimming path [27, 30, 32, 33].

### 2.8 Nissl Staining

First, mouse hippocampal tissue slices were dehydrated in graded ethanol solutions (75%, 85%, 95%, and 100%) and stained with 0.5% crystal violet (190‐M, Sigma‐Aldrich, USA). Then, remove the excess crystal violet three times with PBS, each time for 5 min, and quickly dehydrate the slices in 100% ethanol for 10 s. Finally, the slices were imaged using an optical microscope (Ts2R, Nikon, Japan) and analyzed using ImageJ software [34].

### 2.9 Immunohistochemistry Staining

Hippocampal tissues were embedded in paraffin and sectioned at 5 μm for subsequent staining. Sections were incubated with 3% hydrogen peroxide for 10 min at room temperature to inhibit endogenous peroxidase activity. Non‐specific antibody binding was minimized by incubating the sections in normal goat serum for 10 min. Overnight incubation at 4°C was performed using primary antibodies, mouse anti‐DLAT (1:500, 68303‐1‐Ig, Proteintech, Wuhan, China) and rabbit anti‐PKM2 (1:800, #4053, CST, USA). Sections were incubated with streptavidin‐peroxidase solution (1:500, BM3894/BM3895, Boster, Wuhan, China) for 20 min at 37°C. Secondary antibodies appropriate for rabbit and mouse IgGs were applied subsequently and incubated at room temperature for 10 min. Diaminobenzidine (DAB) was used for visualization, followed by hematoxylin counterstaining. Slides were then dehydrated, cleared, and mounted. Slides were examined under a microscope to identify protein‐positive cells, which appear brownish‐yellow. Negative controls were processed using PBS instead of the primary antibody. Protein expression was quantified using Image‐ProPlus 6.0 by measuring the integrated optical density (IOD) [35].

### 2.10 Immunofluorescence Assay Detected Expression of DLAT and Activation of Microglia

The cells were seeded on covered glass in a six‐well plate overnight and treated as instructed. Subsequently, the cells were incubated with 100 nM Mitotracker Red CMXRos (M7512, Thermo Fisher Scientific, Waltham, MA, USA) for 30 min for mitochondrial staining. The cells were then fixed with 4% paraformaldehyde for 10 min, permeabilized in 0.25% Triton X‐100 for 10 min and blocked with 3% BSA‐PBS for 1 h. Following overnight incubation at 4°C with primary antibody DLAT (rabbit, 1:100, 13426‐1‐AP, Proteintech, Wuhan, China), the cells were washed three times with 0.1% PBST and incubated at 37°C for 1 h with secondary antibody (Alexa Fluor 488; 1:1000, ab150077, Abcam, UK). Mice were anesthetized, and the heart was perfused with 0.9% saline and 4% paraformaldehyde. The brain was then sliced, permeabilized, blocked in 1% BSA (dissolved in PBS containing 0.3% Triton X‐100) for 1 h and incubated overnight at 4°C with primary antibodies DLAT (1:100, 13426‐1‐AP, Proteintech, Wuhan, China), NeuN (1:100, ab177487, Abcam), and Anti‐Iba1 (1:100, ab5076, Abcam). Subsequently, the slides were incubated with the respective secondary antibodies (Alexa Fluor 488; 1:1000, ab150077, Abcam, UK) at 37°C for 1 h.

To assess the activation status of microglial cells in brain tissue, we co‐labeled with rabbit anti‐Iba‐1 (1:200, ab178846, Abcam, UK) and mouse anti‐CD68 (1:50, ab955, Abcam, UK) antibodies, followed by incubation with secondary antibodies: goat anti‐rabbit (Alexa Fluor 488; 1:1000, ab150077, Abcam, UK) or goat anti‐mouse (Cy3; 1:500, ab97035, Abcam, UK). Iba1 recognizes all microglial cells, while CD68 is a marker for activated microglial lysosomes. The staining results were visualized using an IX53 fluorescence microscope (CLSM; LSM 510 META, Carl Zeiss AG). Image‐Pro Plus 6.0 software was utilized for analysis and quantification [15, 36, 37, 38].

### 2.11 Determination of Copper Ion Content

To determine the copper ion content in the hippocampal tissue, the copper assay kit (MAK127, Sigma‐Aldrich, USA) was utilized. A specific amount of hippocampal tissue sample was taken and homogenized in 1 mL of distilled water, followed by centrifugation to obtain the supernatant. Then, 100 μL of supernatant from each well was transferred into a flat‐bottom 96‐well UV detection plate. The sample was thoroughly mixed with 35 μL of reagent, 5 μL of reagent B, and 150 μL of reagent C. After an incubation period at room temperature for 5 min, the absorbance was read at 359 nm using the Epoch microplate spectrophotometer. The copper content was normalized based on the protein concentration [39].

### 2.12 Copper Ion Content Determination by ICP‐MS

Neuronal cell samples from the hippocampus were collected and stored in metal‐free plastic collection tubes for subsequent analysis by ICP‐MS. The samples were dissolved in concentrated nitric acid and then heated in a high‐temperature furnace for resolution. Subsequently, all samples were diluted with purified water. Standard samples (Sero, #1103129) were procured and utilized for quantification. Copper concentration was quantified using ICP‐MS on the NexION instrument, with analysis conducted using argon gas [40].

### 2.13 Isolation of Primary Glial Cells and Hippocampal Neurons

After euthanizing male 5xFAD and WT mice and performing rapid transcardial perfusion with saline, their brains were extracted. Simply put, the brains were minced into 40 μm cell suspensions, washed with PBS at room temperature for 5 min, followed by discarding the supernatant. The cell pellets were resuspended in 6 mL of 37% isotonic Percoll (SIP) solution (90% Percoll and 10% 10X HBSS). The cell suspension was then transferred to a 15 mL conical tube and slowly overlaid with 2 mL of 70% SIP. Subsequently, a slow overlay of 2 mL of 30% SIP was added on top of the 37% SIP layer. The samples were centrifuged at 800 *g*, zero braking, for 25 min at 20°C to remove the top‐floating myelin layer. Carefully collecting 3 mL from the 70%–37% interface without disturbing the 70% SIP layer.

The cells were washed three times with 10 mL of cold PBS and then resuspended the microglial cells in 100 μL of an appropriate buffer. The microglial cells were cultured in DMEM medium (11965084, Thermo Fisher Scientific, USA) containing 10% FBS (26140079, Thermo Fisher Scientific, Waltham, MA, USA). After 5 days of culture, GM‐CSF (PHC2013, Thermo Fisher Scientific, Waltham, MA, USA) was added at a concentration of 25 ng/mL and removed before harvesting.

Primary neurons were dissected from the hippocampus of pregnant female C57BL6 mice at embryonic day 17–18 (E17‐E18), and hippocampal neurons were microdissected and separated from the hippocampus using a stereomicroscope. Disperse the tissues by digesting with trypsin and DNase I (25200114/18047019, Thermo Fisher Scientific, USA) for 30 min at 37°C, followed by incubation in DMEM supplemented with penicillin/streptomycin and HEPES. The neurons were cultivated separately on glass slides coated with a poly‐D‐lysine layer or a poly‐D‐lysine‐coated surface in a microfluidic chamber system. Neurons are cultured in Neurobasal Medium Plus supplemented with B27 plus (A3582801, Thermo Fisher Scientific, Waltham, MA, USA), glutamine, and penicillin/streptomycin. The medium is changed every 2–3 days, with half of the medium replaced.

Primary microglia and hippocampal neurons were identified using immunofluorescence. Cells were fixed with 4% paraformaldehyde at room temperature for 30 min. After washing, blocking solution (comprising 5% donkey serum and 0.1% Triton X‐100 in PBS) was added to the cells and incubated for 2 h at room temperature. Subsequently, 100 μL of rabbit anti‐Iba‐1 (1:200, ab178846, Abcam, UK) or rabbit anti‐MAP‐2 (1:200, #8707, CST, USA) was added onto coverslips and left to incubate overnight at 4°C. Following this, cells were treated with secondary antibodies (goat anti‐rabbit Cy3 at 1:1000, ab6939, Abcam, UK, or Alexa Fluor 488 at 1:1000, ab150077, Abcam, UK) at room temperature for 1 h according to the primary antibody used. Finally, cell observation and image acquisition were performed using a confocal fluorescence microscope [26, 29, 41, 42].

### 2.14 Extraction and Purification of Microglial Exosomes (M‐exosomes, M‐exo)

Primary microglia were cultured in a medium containing 10% FBS (with exosomes excluded, removed by overnight centrifugation at 100,000 *g* at 4°C). Following 48 h of culturing, the cell culture supernatant was collected, and the M‐exosomes were isolated using ultracentrifugation. The specific steps were as follows: centrifugation was initially performed at 300 *g* for 5 min at 4°C, followed by centrifugation at 2000 *g* for 10 min, and then at 10,000 *g* for 30 min to eliminate cells and large debris. Subsequently, the supernatant was filtered using a 0.22 μm micro‐pore filter (SLHV033N, Thermo Fisher Scientific, Waltham, MA, USA). Ultracentrifugation was carried out at 140,000 *g* for 3 h at 4°C, and the supernatant was collected. The pellet was washed with 10 mL of 1× phosphate‐buffered saline, followed by centrifugation at 140,000 *g* for 2 h at 4°C. Finally, the pellet was resuspended in 1× PBS to obtain M‐exosomes, and the amount of M‐exosomes was quantified using the EXOCEP Exosome Quantification Assay Kit (EXOCET96A‐1, System Biosciences Inc., USA) according to the manufacturer's instructions [43, 44].

### 2.15 M‐Exo Identification

Transmission Electron Microscopy (TEM): 20 μL of microglial exosome suspension was applied to a 200‐mesh carbon‐coated grid, left to stand for 3 min, and excess liquid was removed using filter paper. The grid was air‐dried for 1 min and stained with 3% phosphotungstic acid for 5 min. Samples were then examined with a JEM‐2000EX transmission electron microscope (JEOL, Japan).

Nanoparticle Tracking Analysis (NTA): Exosome size and concentration were analyzed using an NS300 Nanoparticle Tracking Analyzer (Malvern Instruments Ltd., UK).

Western Blot Analysis: Expression levels of exosome markers CD9, CD81, and Alix, along with non‐exosomal proteins HSP90 and histone H3, were determined using Western blot. Antibodies used included mouse anti‐CD9 (1:1000, Santa Cruz), rabbit anti‐CD81 (1:1000, Proteintech), rabbit anti‐Alix (1:2000, Proteintech), rabbit anti‐HSP90 (1:1000, CST), and rabbit anti‐histone H3 (1:2000, Abcam) [44, 45, 46].

### 2.16 M‐exo Cell Uptake Experiment

Cells cultured on a four‐chambered glass slide were washed thrice with PBS and then fixed with 4% paraformaldehyde for 15 min. Afterward, wash again with PBS and incubate with 0.5% Triton‐X 100 for 20 min to permeabilize. For the tracking of M‐exo, M‐exo was labeled with PKH67 green fluorescent dye (MINI67, Sigma‐Aldrich, USA). Incubate at room temperature for 5 min with labeled 10 μg/mL M‐exo [31]. Suspension was plated onto a basal medium and incubated with primary hippocampal neurons at 37°C for 12 h. Wash the cells twice with PBS afterward. F‐actin was stained using Atto 633 labeled phalloidin (red; 68825, Sigma‐Aldrich, USA). DAPI is used to label cell nuclei. Stained cells were observed under an IX53 fluorescence microscope (CLSM; LSM 510 META, Carl Zeiss AG) [47, 48].

### 2.17 Plasmid Transfection and Cell Experiment Grouping

Primary microglial cells and hippocampal neurons in a healthy growth state were digested with trypsin and seeded at a density of 8 × 10^3^ cells per well in a 24‐well plate to culture them into a monolayer. Subsequently, the culture medium was removed, and transfection was carried out following the instructions of Lipofectamine 3000 (L3000150, Invitrogen, Thermo Fisher Scientific, Waltham, MA, USA). Post‐transfection, the cells were cultured at 37°C with 5% CO_2_ for 6–8 h, after which the complete culture medium was replaced. RNA and protein extraction were performed 48 h post‐culturing for subsequent experiments.

The specific groups are as follows (Figure S2):

- Microglia: sh‐NC group, sh‐hnRNPA2B1–1 group, sh‐hnRNPA2B1–2 group, sh‐PKM‐1 group, and sh‐PKM‐2 group;
- Hippocampal neurons:
  1. PBS group, WT‐M‐exo group, 5xFAD‐M‐exo group with 1 μg M‐exo treatment for 24 h;
  2. DMSO group, GW4869 group, for 5xFAD group, microglia cells were treated with exosome inhibitor GW4869 (10 μM; D1692, Sigma‐Aldrich, USA) or 0.1% DMSO for 48 h, followed by co‐culture with hippocampal neurons for 24 h;
  3. PBS group, M‐exo group (5xFAD‐M‐exo), M‐exo‐sh‐NC group (exosomes extracted from primary microglia cells transfected with sh‐NC), and M‐exo‐sh‐PKM group (exosomes extracted from primary microglia cells transfected with sh‐PKM), with 1 μg M‐exo treatment for 24 h; and
  4. M‐exo‐sh‐NC + oe‐NC group, M‐exo‐sh‐PKM + oe‐NC group, and M‐exo‐sh‐PKM + oe‐DLAT group, hippocampal neurons were transfected with oe‐NC or oe‐DLAT for 48 h, followed by 1 μg M‐exo treatment for 24 h.

The reference manuals of each plasmid were consulted for their recommended concentrations and adjusted according to actual conditions. The overexpression plasmid for DLAT (oe‐DLAT) and its control plasmid (oe‐NC) were purchased from Ruibo Biotechnology (R11091.1, Ruibo Biotechnology, Guangzhou, China) and constructed using the pEXP‐RB‐Mam plasmid vector (R11091.1). The shRNA sequences used were as follows: sh‐PKM‐1 (TRCN0000366062; target sequence: GACTGGAAACCCTGACTTTAT), sh‐PKM‐2 (TRCN0000362351; target sequence: GGCTCTGGACCATCTACATAG), sh‐hnRNPA2B1–1 (TRCN0000366062; target sequence: GTCACAATGCAGAAGTTAGAA), sh‐hnRNPA2B1–2 (TRCN0000362351; target sequence: CGATAGGCAGTCTGGAAAGAA), and the negative control (sh‐NC) purchased from Sigma‐Aldrich (USA) [44, 49].

### 2.18 Cell Counting Kit‐8 (CCK‐8) Assay

Healthy hippocampal primary neurons were seeded in a 96‐well plate at a density of 3 × 10^3^ to 6 × 10^3^ cells per well and incubated in a cell culture incubator for 24 h. The following treatments were applied: (1) Incubation of cells for 24 h with CuSO4 solutions of varying concentrations (0, 20, 40, 60, 80, 100, 120, 140 μM) (anhydrous CuSO4: 451657, Sigma‐Aldrich, USA); (2) addition of the copper chelator TTM (tetrathiomolybdate; 20 μM, 323,446, Sigma‐Aldrich, USA) to the cells treated with Cu (CuSO4) for 2 h, followed by a medium change; (3) DLAT knockdown in hippocampal neurons after treatment with 80 μM CuSO4. Subsequently, 10 μL of CCK‐8 solution (96992, Sigma‐Aldrich, USA) was added to each well. After 1 h of incubation at 37°C in a humidity‐controlled cell culture incubator, the absorbance of each sample was measured at 450 nm using an Epoch microplate spectrophotometer (Bio‐Tek, Winooski, VT, USA) [32, 50, 51, 52].

### 2.19 RIP Experiment

The cell pellets were lysed with equal volumes of RIP lysis buffer (P0013B, Biyuntian, Shanghai, China) on ice for 5 min, followed by centrifugation at 14,000 *g* for 10 min at 4°C to obtain the supernatant. The RIP assay kit (17‐701, Sigma‐Aldrich, USA) was then used to detect the binding of hnRNPA2B1 and PKM. First, the magnetic beads from each co‐immunoprecipitation reaction system were resuspended in 100 μL of RIP buffer and incubated at 4°C for 6 h. Then, 50 μL of the bead‐antibody complex was added and incubated with 5 μg of rabbit anti‐hnRNPA2B1 (1:100, 14813‐1‐AP, Proteintech, Wuhan, China) or control IgG antibody (ab172730, 1:100, Abcam, UK). The bead‐antibody complexes were then resuspended in 900 μL of RIP wash buffer and incubated overnight at 4°C with 100 μL of cell extract. The samples were subsequently placed on a magnet to collect the magnetic bead‐protein complexes. The samples were then treated with proteinase K to remove proteins, and RNA was extracted and reverse transcribed into cDNA using the manufacturer's protocol provided with Platinum SuperFi DNA polymerase (12361010, Invitrogen, Thermo Fisher Scientific, Waltham, MA, USA). The PCR products were loaded onto a 2% agarose gel (D0163S, Biyuntian, Shanghai, China) and visualized using ethidium bromide staining [49, 53].

### 2.20 Co‐IP

Lyse hippocampal neurons in IP lysis buffer (P0013, BiyunTian, Shanghai, China) containing protease and phosphatase inhibitors. The cell lysate was centrifuged at 12,000 *g* for 20 min at 4°C. Then, the cell lysates containing 200 μg of protein were incubated separately with mouse anti‐PKM2 (1:50, #4053, CST, USA), rabbit anti‐DLAT (1:100, 13426‐1‐AP, Proteintech, Wuhan, China), or rabbit anti‐IgG antibody (1:50, #3900, Cell Signaling, USA) at 4°C overnight. Then, the antibody‐protein complex was captured using Protein A/G PLUS‐Agarose beads (sc‐2003, Santa Cruz, USA). After thoroughly washing with detergent buffer, boil the complex in 1× SDS loading buffer and then analyze it by western blot. The antibodies used for Western blot were rabbit anti‐PKM2 (1:250, 25659‐1‐AP, Proteintech, Wuhan, China) and mouse anti‐DLAT (1:5000, 68303‐1‐Ig, Proteintech, Wuhan, China) [54, 55].

### 2.21 RT‐qPCR

Total RNA was extracted from primary hippocampal neurons, microglia, and mouse hippocampal tissue using Trizol reagent (Invitrogen, Car, Cal, USA, catalog number 15596026). The concentration and purity of the extracted total RNA were measured using a Nanodrop2000 micro‐UV spectrophotometer (nanodrop, USA, catalog number 1011U). Subsequently, 1 μg of total RNA from microglia was reverse transcribed as a template for routine PCR reactions using M‐MLV reverse transcriptase (M1302, Sigma‐Aldrich, USA) (GoTaq DNA Polymerase, M3001, Promega, USA). The products of PKM1 and PKM2 were analyzed on agarose or acrylamide gels.

For primary hippocampal neurons and mouse hippocampal tissue, the total RNA was reverse transcribed into cDNA using the PrimeScript RT reagent Kit (Takara, Japan, catalog number RR047A) following the instruction manual. The reverse transcription conditions were 42°C for 30–50 min and 85°C for 5 s. qRT‐PCR detection was performed using the Fast SYBR Green PCR Kit (Takara, Japan, catalog number RR820A) and the ABI PRISM 7300 RT‐PCR system (Applied biosystems). The reaction conditions included 95°C pre‐denaturation for 5 min, 95°C denaturation for 30 s, 57°C annealing for 30 s, and 72°C elongation for 30 s, for a total of 40 cycles. Each hole was set with three repetitions. The relative gene expression level was analyzed using β‐actin as an internal reference by the $2^{-\Delta \Delta C_{t}}$ method, where ΔΔ*C*_t_ = (average *C*_t_ value of the target gene in the experimental group—average *C*_t_ value of the reference gene in the experimental group)—(average *C*_t_ value of the target gene in the control group—average *C*_t_ value of the reference gene in the control group). The experiment was repeated three times. The primer sequences are shown in Table S1.

### 2.22 Western Blot

Cell and tissue samples were separately added into RIPA lysis buffer (P0013B, Beyotime, Shanghai, China) containing 1% PMSF. The samples were incubated on ice for 30 min and then centrifuged at 14,000 *g*, 4°C to collect the supernatant. The protein concentration in the extraction solution was determined using the BCA method (P0012S, Biyun Tian, Shanghai, China). An appropriate amount of 5× loading buffer was added, and the mixture was boiled at 100°C for 10 min to denature the proteins. A protein loading amount of 50 μg was used. The gel and concentrated gel were prepared for electrophoresis. Subsequently, the bands containing the target protein were transferred onto the PVDF membrane.

The PVDF membrane was immersed in 5% skim milk powder, sealed at room temperature for 1 h, and then incubated with the following primary antibodies: rabbit anti‐PKM1 (1:1000, #7067, CST, USA), rabbit anti‐PKM2 (1:1000, #4053, CST, USA), mouse anti‐DLAT (1:5000, 68303‐1‐Ig, Proteintech, Wuhan Sanying, China), rabbit anti‐FDX1 (1:1000, 12592‐1‐AP, Proteintech, Wuhan Sanying, China), rabbit anti‐HSP70 (1:5000, 10995‐1‐AP, proteintech, Wuhan Sanying, China), rabbit anti‐POLD1 (1:1000, 15646‐1‐AP, Proteintech, Wuhan Sanying, China), rabbit anti‐ACO2 (1:2000, 11134‐1‐AP, Proteintech, Wuhan Sanying, China), rabbit anti‐SDHB (1:5000, 10620‐1‐AP, Proteintech, Wuhan Sanying, China), rabbit anti‐hnRNPA2B1 (1:2000, 14813‐1‐AP, Proteintech, Wuhan Sanying, China), rabbit anti‐PTBP1 (1:2000, 12582‐1‐AP, Proteintech, Wuhan Sanying, China), mouse anti‐hnRNPA1 (1:20,000, 67844‐1‐Ig, Proteintech, Wuhan Sanying, China), and rabbit anti‐β‐actin (1:1000, ab8226, Abcam, UK).

The membrane was then incubated with HRP‐labeled sheep anti‐rabbit/sheep anti‐mouse IgG secondary antibody (1:10,000, BA1054/BA1050, Proteintech, Wuhan, China) at room temperature for 1 h, using β‐actin as the reference. Washes were carried out six times with phosphate‐buffered saline with Tween (PBST) for 5 min each. The ECL reaction solution (AR1172, Boster, Wuhan, China) was evenly added to the membrane, which was then exposed in an Amersham Imager 600 (USA). Grayscale analysis was performed using Image J. The experiment was repeated thrice [56, 57].

### 2.23 Statistical Analysis

The data statistical analysis in this study was conducted using Graphpad Prism 8.1. Metric data were presented as mean ± standard deviation. Prior to the statistical analysis, normality of distribution was assessed using the Shapiro–Wilk test, along with homogeneity of variances test. For data following a normal distribution, between‐group comparisons were performed using unpaired t‐test; for comparisons involving multiple groups, one‐way analysis of variance (ANOVA) or repeated measures ANOVA was employed, followed by post hoc analysis using Tukey's test. Repeated measures ANOVA was utilized for behavioral experiments analysis, with post hoc testing adjusted with Bonferroni correction as necessary. Two‐way ANOVA was applied for the analysis of weight and Western blot quantification. Unpaired *t*‐test was used for between‐group comparisons of normally distributed data. For multiple group comparisons, one‐way ANOVA or repeated measures ANOVA was conducted, followed by Tukey post hoc analysis if needed. Non‐parametric tests such as Mann–Whitney *U* test or Kruskal–Wallis test were employed for data not conforming to normal distribution. A *p*‐value < 0.05 was considered statistically significant.

## 3 Results

### 3.1 Identification of 11 Copper Death‐Related Genes Differentially Expressed in AD

Copper death is a novel form of cell death. According to reports, copper metabolism disorder is associated with some neurodegenerative diseases, including AD and Parkinson's disease [32]. Despite the ongoing research, it has been found that there are neuronal damage and apoptosis in AD [58]. However, there are few reports on whether the damage of neurons in AD could be mediated by copper‐induced death.

First, we obtained the AD‐related gene expression dataset GSE97760 from the GEO database. Differential analysis was then conducted to identify 7861 differentially expressed genes, including 4100 upregulated genes and 3761 downregulated genes (Figure S3A,B). The log2FC values and corresponding *p*‐values of all differentially expressed genes were found in Table S2. Taking the intersection of differentially expressed genes with copper death‐related genes, we obtained 11 differentially expressed genes related to copper death (Figure S3C). The differential expression results of these 11 copper death genes are shown in Figure S3D. DBT, DLAT, DLD, FDX1, GCSH, GLS, LIAS, and LIPT1 are overexpressed in peripheral blood samples of AD patients. NLRP3 is underexpressed in peripheral blood samples of AD patients. CDKN2A and SLC31A1 show no differential expression changes.

The above results indicate that we have identified 11 copper death‐related genes with differential expression in AD through bioinformatics analysis.

### 3.2 DLAT May Be a Key Gene Mediating Neuronal Copper Death in AD

To further select the key copper death‐related genes, we used the lasso and SVM‐RFE machine learning algorithms to screen for disease‐specific genes among the 11 overlapping copper death genes. The lasso and SVM‐RFE analyses identified six and eight disease‐specific genes, respectively, and the intersection of these genes yielded four key disease‐specific genes (CDKN2A, DLAT, GCSH, and NLRP3) (Figure S4A–C). Import the proteins encoded by 11 intersecting copper death genes into the STRING database for protein interaction analysis. DLD, GCSH, LIAS, DBT, DLAT, and LIPT1 are located in the relative core position of the protein interaction network (Figure S4D). We speculate that DLAT and GCSH may be more prominent copper death genes based on the above information.

In addition, GO and KEGG enrichment analyses were performed on 11 intersecting copper death genes. The GO enrichment analysis results showed that these 11 intersecting copper death genes are mainly located in the mitochondria and mitochondrial matrix, and they participate in processes such as protein acylation, cellular nitrogen compound metabolism, and negative regulation of NF‐kappaB transcription factor activity as part of the mitochondrial pyruvate dehydrogenase complex (Figure S4E). The KEGG pathway enrichment analysis results indicate that the 11 intersected copper death genes are mainly enriched in metabolic pathways, platinum resistance, carbon metabolism, microRNAs in cancer, and the tricarboxylic acid (TCA) cycle (Figure S4F). Copper death is caused by the binding of copper with acyltransferase in the TCA cycle, leading to subsequent protein aggregation, protein toxicity stress, and cellular death [15, 59]. KEGG enrichment analysis revealed that DLAT is the main enriched gene in the TCA cycle pathway. According to research reports, DLAT is acylated dihydroceramide S‐acyltransferase, which serves as the E2 component of the pyruvate dehydrogenase complex and is one of the key molecules involved in copper‐induced cell death. Additionally, copper could promote the expression of copper death‐related protein FDX1 and DLAT, inducing neuronal degeneration and oxidative damage, leading to cognitive dysfunction in mice [15, 32]. GCSH has not been studied in AD diseases yet.

Therefore, we speculate that DLAT may mediate neuronal copper death in AD.

### 3.3 Evidence of Copper Death and Elevated DLAT Expression in the Hippocampal Tissue of a 5xFAD AD Mouse Model

**FIGURE 1:**
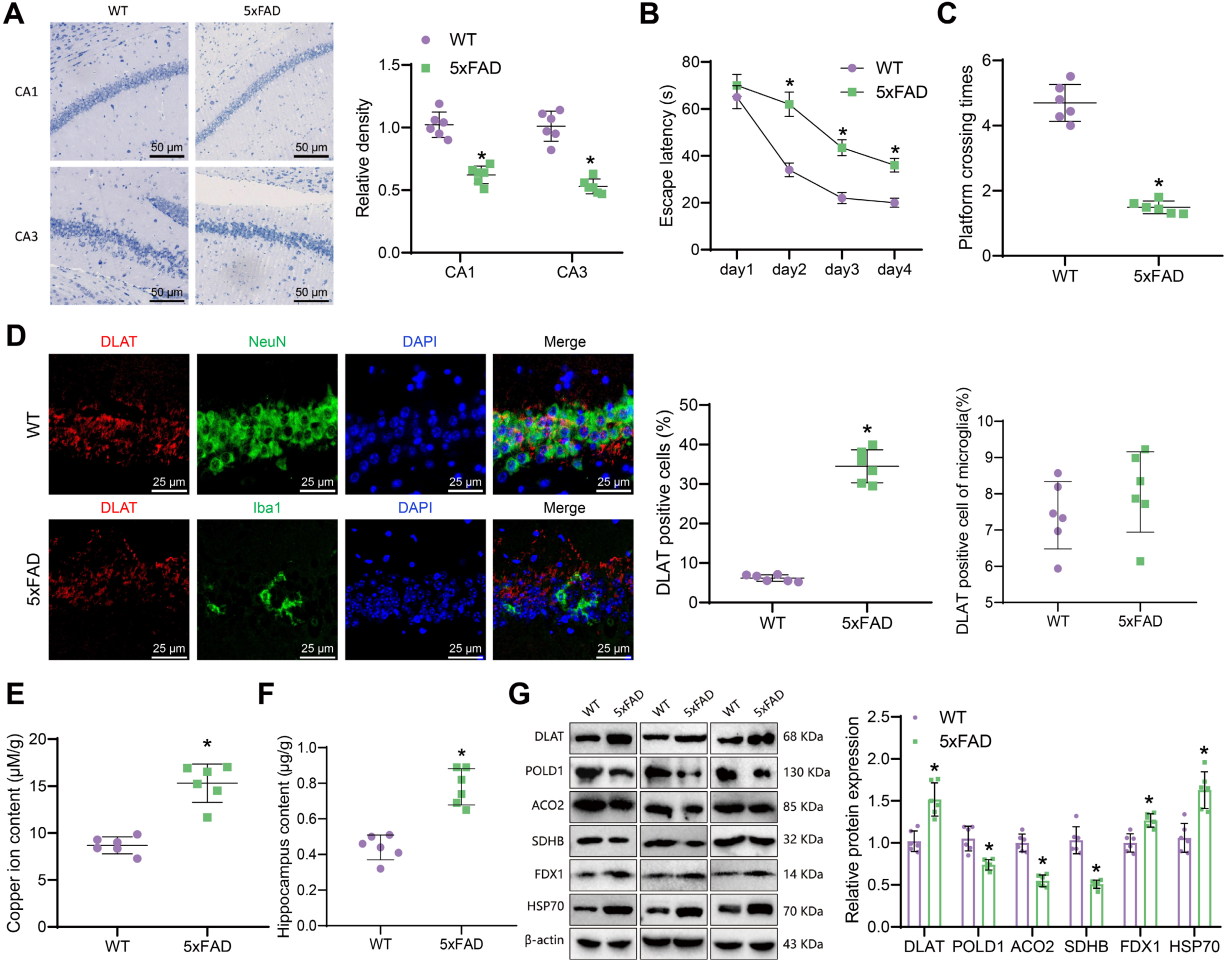
Expression of copper‐induced cell death‐related proteins in the hippocampal tissue of an AD mouse model. *Note:* (A) Nissl staining of neuronal damage (50 μm) in the CA1 and CA3 regions of the hippocampal tissue of WT and 5xFAD mice; (B) escape latency of mice in the Morris water maze test; (C) time spent crossing the platform in the Morris water maze test; (D) immunofluorescence staining of DLAT, NeuN and Iba1 expression in the hippocampal tissue of mice (25 μm); (E) copper ion content in the hippocampal tissue of mice; (F) the copper ion content in hippocampal neurons of each group; (G) western blot analysis of DLAT, Fe‐S cluster proteins, and protein toxicity stress markers in the hippocampal tissue of mice, *represents *p* < 0.05 compared to the WT group, each group consists of six mice.

To verify the expression of DLAT in AD, we used the 5xFAD transgenic mouse model to construct an AD model. First, we evaluated the neuronal damage in mouse hippocampal tissue using Nissl staining. The results showed that the Nissl bodies in the hippocampal tissue of 5xFAD mice, an AD mouse model, were reduced, indicating substantial neuronal damage in the hippocampus (Figure 1A). The Morris water maze experiment results showed that, compared to the WT group, the escape latency of the 5xFAD group mice was prolonged, and the time to cross the platform was reduced (Figure 1B,C). Subsequently, we utilized immunofluorescence to detect the expression of DLAT. The results revealed that DLAT is exclusively expressed in neurons labeled with NeuN and not in microglial cells marked with Iba1, indicating the specificity of DLAT expression (Figure 1D). Furthermore, the expression of DLAT in the hippocampal tissue of 5xFAD mice was significantly elevated compared to the WT group, with noticeable aggregation of DLAT in the hippocampal tissue of 5xFAD mice (Figure 1D).

Research reports that the characteristics of copper death include copper ion accumulation, lipoprotein aggregation, and subsequent loss of iron–sulfur (Fe‐S) cluster proteins, causing protein toxicity stress and ultimately leading to cell death [15]. Therefore, we further detected the content of copper ions in the hippocampus tissue. The results showed that compared with the WT group, the content of copper ions in the hippocampus tissue of the 5xFAD group mice increased (Figure 1E). Furthermore, to precisely measure the copper ion content within hippocampal neurons, we employed ICP‐MS for further analysis. The results demonstrated a significant increase in copper ion levels in neurons of the hippocampal tissue of 5xFAD mice compared to the WT group (Figure 1F).

Western blot detected the expression of DLAT, Fe‐S cluster proteins, and protein toxicity stress markers. The results showed that the expression of DLAT, FDX1, and HSP70 proteins in the hippocampal tissue of 5xFAD mice was increased compared to the WT group. Fe‐S cluster proteins POLD1, ACO2, and SDHB expression decreased (Figure 1G). The result indicates that copper death occurred in the hippocampal tissue of AD mice.

The above results indicate that copper death occurs in the hippocampal tissue of AD mice, and DLAT is highly expressed in the hippocampal tissue of AD mice.

### 3.4 Knockdown of DLAT Alleviates Copper‐Induced Cell Death and Cognitive Dysfunction in 5xFAD AD Mouse Model

**FIGURE 2:**
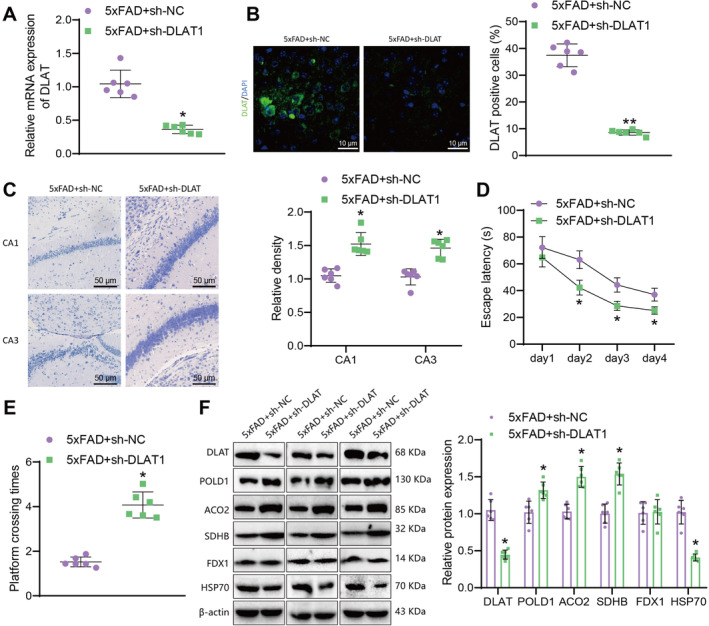
Effects of DLAT knockdown on copper‐induced cell death and cognitive impairment in an AD mouse model. *Note:* (A) RT‐qPCR analysis of DLAT mRNA expression in the hippocampal tissue of mice after DLAT knockdown; (B) immunofluorescence staining of DLAT expression in the hippocampal tissue of mice (10 μm); (C) Nissl staining of neuronal damage (50 μm) in the CA1 and CA3 regions of the hippocampal tissue of mice; (D) escape latency of mice in the Morris water maze test; (E) time spent crossing the platform in the Morris water maze test; (F) western blot analysis of DLAT, Fe‐S cluster proteins, and protein toxicity stress markers in the hippocampal tissue of mice, *represents *p* < 0.05 compared to the 5xFAD + sh‐NC group, **represents *p <* 0.01 compared to the 5xFAD + sh‐NC group, each group consists of 6 mice.

Next, we will investigate whether knocking down DLAT could suppress copper‐induced cell death and improve hippocampal tissue damage in AD model mice. 5xFAD mice were respectively injected with sh‐DLAT or sh‐NC lentivirus through the lateral ventricle. At the end of the experiment, hippocampal tissue samples were collected from each group of mice. RT‐qPCR and immunofluorescence staining results showed that compared with the 5xFAD + sh‐NC group, the expression of DLAT in the hippocampal tissue of 5xFAD + sh‐DLAT group mice was reduced, and the accumulation of DLAT in the hippocampal tissue of the mice disappeared (Figure 2A,B). Nissl staining results showed that compared with the 5xFAD + sh‐NC group, the 5xFAD + sh‐DLAT group exhibited an increase in Nissl bodies in the hippocampal tissue of mice, indicating a reduction in hippocampal neuronal damage (Figure 2C). The results of the Morris water maze experiment showed that compared to the 5xFAD + sh‐NC group, the escape latency decreased, and the time spent crossing the platform increased in the 5xFAD + sh‐DLAT group (Figure 2D,E), indicating that knocking down DLAT alleviated cognitive impairment in AD mice.

By further detecting the expression of DLAT and copper death‐associated proteins through Western blot, it was found that compared to the 5xFAD + sh‐NC group, the expression of DLAT and HSP70 proteins in the hippocampal tissue of mice in the 5xFAD + sh‐DLAT group was decreased. The expression of Fe‐S cluster proteins POLD1, ACO2, and SDHB was increased. FDX1, as an upstream regulatory factor of DLAT, is involved in this process [15]. There was no change in the expression of FDX1 protein (Figure 2F).

The above results indicate that knocking down DLAT could inhibit copper‐induced cell death and improve cognitive dysfunction in AD model mice.

### 3.5 Analysis of snRNA‐Seq Data Revealed an Increase in Microglial Cells in AD, Which Interact With Neurons

Research has shown that microglial cells play crucial roles in neurodegenerative diseases such as Parkinson's disease (PD), AD, and amyotrophic lateral sclerosis (ALS). The exosomes released by microglial cells can be transported to neurons and taken up by them, which may have beneficial or detrimental effects on central nervous system disorders [60]. In this study, we conducted single‐nucleus RNA sequencing (snRNA‐seq) analysis on the GEO database dataset GSE140510 to investigate the interaction between microglial cells and neurons.

After quality control and filtering of the snRNA‐seq data, we obtained high‐quality cell data and selected the top 2000 highly variable genes based on variance for further analysis. Subsequently, principal component analysis was performed on the filtered cells, showing the distribution of cells in PC_1 and PC_2 (Figure S5A). It was observed that there were batch effects among the samples. Therefore, batch correction was carried out, and PC_1‐PC_20 could effectively reflect the information contained in the selected highly variable genes with significant analytical implications (Figure S5B,C). The batch correction results demonstrated the successful elimination of sample batch effects (Figure S5D).

**FIGURE 3:**
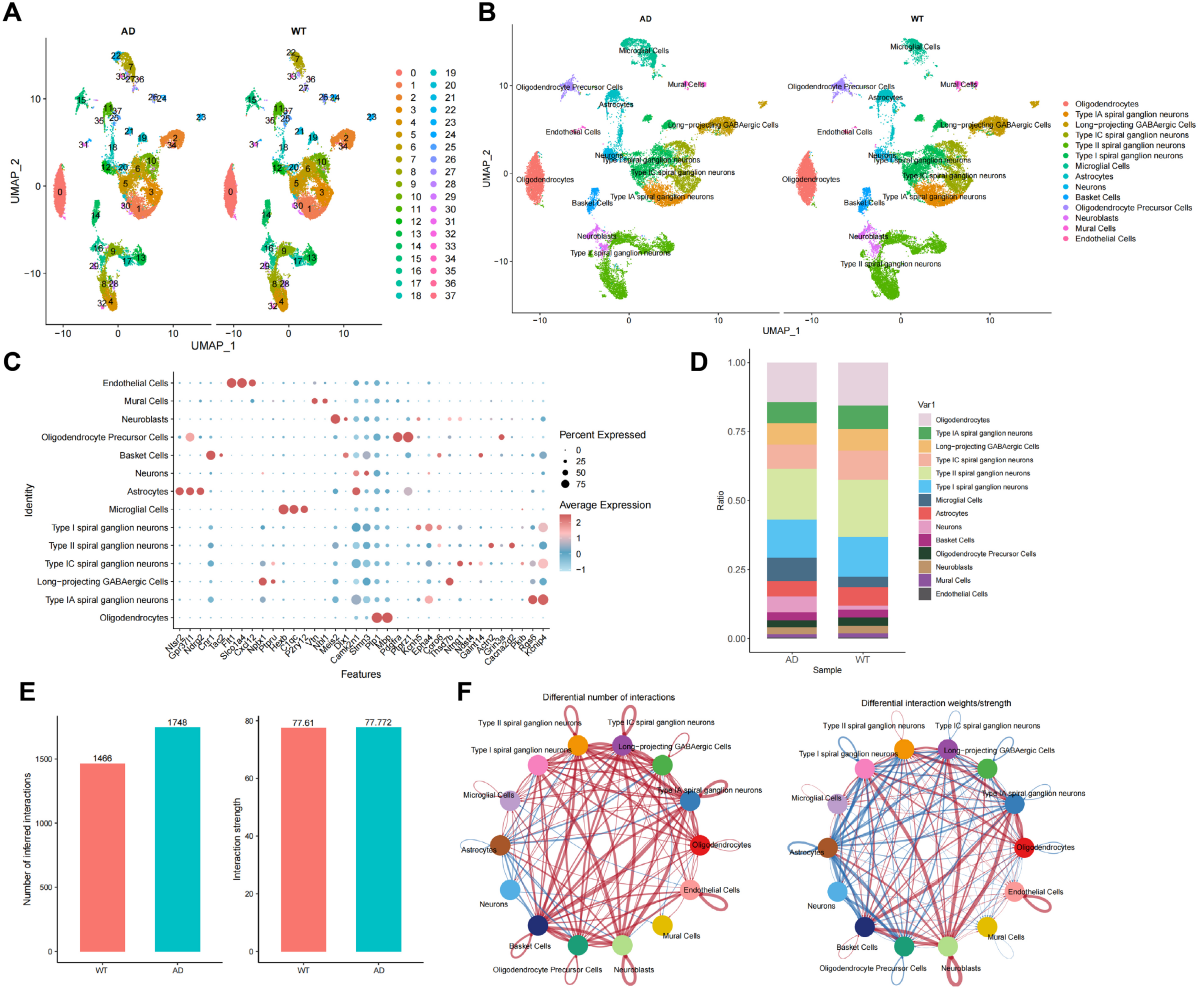
Analysis of cell abundance variations and intercellular communication in AD revealed by snRNA‐seq data. *Note:* (A) Visualization of cell annotation results based on UMAP clustering; (B) group visualization of cell annotation results based on UMAP clustering; (C) dot plot showing the expression of marker genes for 14 cell types in each cell subpopulation, with darker red indicating higher average expression levels; (D) stacked bar chart depicting the proportion of the 14 cell types in each sample group; (E) statistics on the total number of cell interactions and interaction strength between WT and AD groups; (F) circular diagram illustrating alterations in cell communication between AD and WT groups, where red lines represent increased communication in AD, blue lines indicate decreased communication in AD, and the thickness of the lines on the left correlates with the number of pathways, while the thickness of the lines on the right represents the interaction strength.

Next, we applied the UMAP algorithm to nonlinearly reduce the top 20 principal components and clustered all cells into 38 cell clusters (Figure 3A). Using known cell lineage‐specific marker genes obtained from literature search and CellMarker, we annotated the cells and identified 14 cell types, including microglia, basket cells, endothelial cells, GABAergic Cells, microglial cells, mural cells, neuroblasts, neurons, oligodendrocytes, oligodendrocyte precursor cells, Type I, IC, and II spiral ganglion neurons, and Type IA spiral ganglion neurons (Figure 3B). Additionally, we displayed the expression of cell marker genes in these 14 cell types (Figure 3C). Through *T*‐tests, we analyzed the quantitative differences of these cells between two sample groups, revealing a significant increase in microglial cells in AD (Figure 3D).

Subsequently, we utilized the R package “CellChat” for cell communication network analysis. Initially, we conducted a statistical analysis of the total number and strength of interactions between cells in the WT and AD groups, showing a significant increase in cell interactions in the AD group (Figure 3E). Further analysis of the changes in cell communication between the AD and WT groups revealed a significant increase in interactions between microglial cells and Type I spiral ganglion neurons and other neurons in the AD group compared to the WT group (Figure 3F, Figure S6).

These findings suggest that microglial cells increase in AD and their interactions with neurons are enhanced, indicating a potentially significant role of microglial cells in the progression of AD.

### 3.6 Delivery of PKM2 Into Hippocampal Neurons by Extracellular Vesicles Secreted by Microglia

**FIGURE 4:**
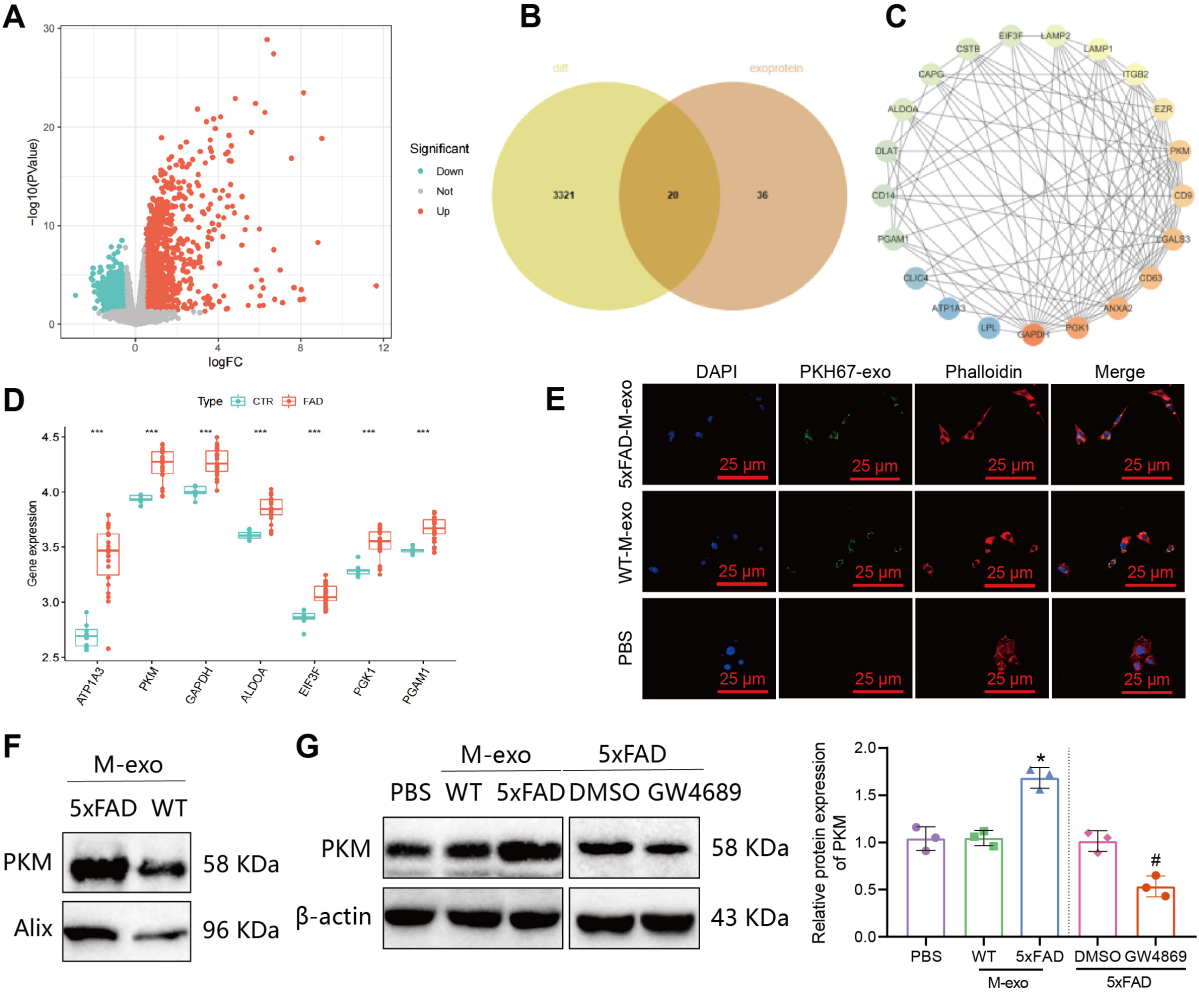
Involvement of microglia‐derived extracellular vesicles in the transfer of PKM2 to hippocampal neurons in AD. *Note:* (A) Volcano plot showing differentially expressed genes between control and 5xFAD mouse microglia in the GSE163857 dataset, CTR group, *n* = 8, FAD group, *n* = 22; (B) Venn diagram showing the intersection between differentially expressed genes in the GSE163857 dataset and genes from the ExoCarta database related to microglia‐derived extracellular vesicles; (C) protein–protein interaction network of the 20 intersecting genes and DLAT‐encoded protein, color gradient from red to blue indicates decreasing core degree; (D) boxplots of the differential expression of 7 genes interacting with DLAT in different treatment groups, CTR (control) group, *n* = 8, FAD group, *n* = 22; (E) immunofluorescence staining of M‐exo uptake in hippocampal neurons (25 μm), DAPI (blue) staining represents nuclei, PKH67 (green) staining represents M‐exo, Phalloidin (red) staining represents cytoskeleton; (F) western blot analysis of PKM2 expression in M‐exo from 5xFAD and WT mice; (G) western blot analysis of PKM2 expression in hippocampal neurons after co‐culture with M‐exo or microglia‐conditioned medium in different treatment groups, *represents *p* < 0.05 compared to the PBS group, #represents *p* < 0.05 compared to the DMSO group, each experiment was repeated three times.

To investigate the role of microglia in AD, we assessed the activation status of microglia in AD. Immunofluorescence results revealed a significant increase in activated microglia levels in the hippocampal tissue of 5xFAD mice (Figure S7A). Subsequently, utilizing the GEO database, we obtained a high‐throughput sequencing dataset, GSE163857, related to microglia in AD mice. Differential analysis indicated a total of 1382 significantly upregulated genes and 1959 significantly downregulated genes in AD mouse microglia (Figure 4A). The log2 Fold Change (FC) values and corresponding *p*‐values of all differentially expressed genes can be found in Table S3.

Moreover, by accessing the ExoCarta database, we identified 20 intersecting genes expressed in microglial exosomes, matching the differentially expressed genes from the sequencing dataset (Figure 4B). Protein–protein interaction analysis was conducted for these 20 intersecting genes, with results showing that GAPDH, PGK1, ANXA2, CD63, LGALS3, CD9, and PKM were located at the core of the network. Interacting proteins with DLAT included ATP1A3, PKM, GAPDH, PGK1, ALDOA, EIF3F, PGAM1 (Figure 4C). Notably, the proteins interacting with DLAT exhibited high expression levels in AD mouse microglia, with ATP1A3 and PKM showing the most significant differences as illustrated in (Figure 4D). Further prediction via the PPA‐Pred website suggested a potent binding affinity between ATP1A3 and PKM with DLAT (Kd < 10–8 M, indicative of strong binding, see Table S4). Literature reports indicate a specific upregulation of pyruvate kinase M2 (PKM2) in the cerebrospinal fluid of AD patients, and high expression of PKM2 in the brains of AD patients and mouse models [61, 62]. However, no previous studies have reported on the expression changes of ATP1A3 in AD. Thus, we postulate that microglial exosomes may transfer PKM2 to promote the progression of AD.

Subsequently, we isolated primary microglia and hippocampal neurons. The identification of primary microglia and hippocampal neurons is depicted in Figure S7B. Compared to the WT group, the expression of PKM2 in primary microglia from the 5xFAD group significantly increased, as shown in Figure S7C. Subsequently, M‐exos were extracted from the primary microglia of the 5xFAD and WT groups. Electron microscopy observations revealed cup‐shaped vesicles surrounded by a lipid bilayer membrane, with a densely stained outer layer and an uneven lightly stained inner zone, displaying typical M‐exo morphological features, as depicted in Figure S8A. Nanoparticle tracking analysis (NTA) indicated that the majority of M‐exos had a diameter within the range of 124.1 ± 4.5 nm, as shown in Figure S8B. Western blot analysis demonstrated the expression of the M‐exo markers CD81, CD9, and Alix in M‐exos from both the 5xFAD and WT groups, while HSP90 and histone H3 were expressed in cells but almost absent in M‐exos, as illustrated in Figure S8C.

Subsequently, M‐exos from the 5xFAD and WT groups were co‐incubated with hippocampal neurons for 24 h. Fluorescence microscopy revealed green fluorescence in the cytoplasm of hippocampal neurons, indicating uptake of PKH67‐labeled M‐exos from both 5xFAD and WT groups (Figure 4E), suggesting that microglial M‐exos can be internalized by hippocampal neurons. Further evaluation of PKM2 expression in M‐exos from the 5xFAD and WT groups showed a significant increase in PKM2 protein expression in 5xFAD group M‐exos compared to the WT group (Figure 4F). Following co‐incubation with M‐exos from the 5xFAD group, hippocampal neurons displayed a notable increase in PKM2 expression. Additionally, after treatment of 5xFAD group microglia with the exosome inhibitor GW4689, co‐incubation of conditioned medium with hippocampal neurons revealed a substantial decrease in PKM2 expression in the neurons (Figure 4G).

These results collectively indicate that microglial exosome‐mediated transfer of PKM2 can be internalized by hippocampal neurons.

### 3.7 HnRNPA2B1 Promotes PKM2 Packaging Into Microglial Extracellular Vesicles

**FIGURE 5:**
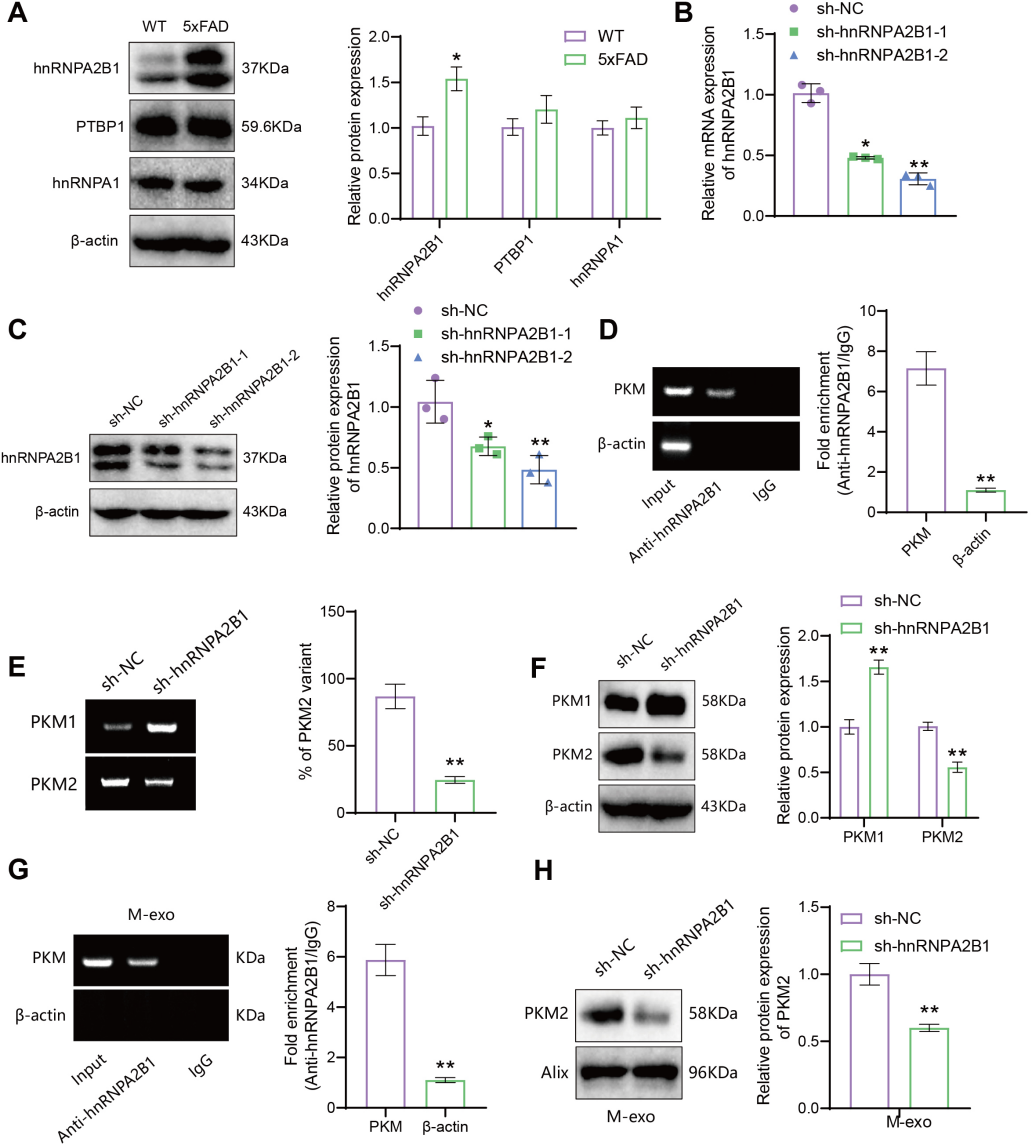
hnRNPA2B1 regulates the expression of PKM2 in microglia and their extracellular vesicles. *Note:* (A) Western blot analysis of the expression of PTBP1, hnRNPA2B1, and hnRNPA1 in primary microglia. * indicates *p* < 0.05 compared to the WT group; (B, C) RT‐qPCR analysis of hnRNPA2B1 mRNA and protein expression after hnRNPA2B1 knockdown. * indicates *p* < 0.05, ** indicates *p* < 0.01 compared to the sh‐NC group; (D) RIP experiment to detect the binding of hnRNPA2B1 and PKM in primary microglia. ** indicates *p* < 0.01; (E) PCR analysis of PKM isoforms PKM1 and PKM2 expression in various cell groups. ** indicates *p* < 0.01 compared to the sh‐NC group; (F) western blot analysis of PKM1 protein expression in various cell groups. ** indicates *p* < 0.01 compared to the sh‐NC group; (G) RIP experiment to detect the binding of hnRNPA2B1 and PKM in microglia‐derived extracellular vesicles. ** indicates *p* < 0.01; (H) western blot analysis of PKM1 and PKM2 protein expression in extracellular vesicles from various cell groups. ** indicates *p* < 0.01 compared to the sh‐NC group. Cell experiments were repeated three times.

Research reports that selective splicing of PKM plays an important role in epilepsy [63]. Three hnRNP proteins, hnRNPI/PTBP1, hnRNPA2B1, and hnRNPA1, act as selective splicing factors that promote PKM2 splicing [64]. To investigate whether the expression of PKM2 in AD is regulated by splicing factors, we first examined the expression of hnRNPI/PTBP1, hnRNPA2B1, and hnRNPA1 in primary microglia from the 5xFAD mouse model. The results showed that compared to the wild‐type (WT) group, the 5xFAD group had significantly increased expression of hnRNPA2B1, while the expression of PTBP1 and hnRNPA1 showed no significant change (Figure 5A).

To test whether hnRNPA2B1 can be recruited to PKM transcripts, we performed RIP experiments to determine the binding between hnRNPA2B1 and PKM after knocking down hnRNPA2B1. First, we measured the knockdown efficiency of hnRNPA2B1 using RT‐qPCR and Western blotting. The results showed that the expression of hnRNPA2B1 was significantly decreased in the sh‐hnRNPA2B1‐1 and sh‐hnRNPA2B1‐2 groups compared to the sh‐NC group, with better knockdown efficiency observed in the sh‐hnRNPA2B1‐2 group, which was used for subsequent experiments (Figure 5B,C). RIP assays revealed that hnRNPA2B1 can bind to PKM, and the binding between hnRNPA2B1 and PKM was significantly reduced after hnRNPA2B1 knockdown (Figure 5D). Examination of the splicing and protein expression changes of PKM2 showed that knockdown of hnRNPA2B1 led to reduced splicing of PKM2, significantly decreased protein expression of PKM2, and increased splicing and protein expression of PKM1 (Figure 5E,F).

To further investigate the effect of hnRNPA2B1 on PKM2 in exosomes, we performed RIP experiments on exosome lysates from microglia and observed the interaction between hnRNPA2B1 and PKM in exosomes (Figure 5G). Further examination of PKM2 protein expression in exosomes showed that knockdown of hnRNPA2B1 led to a significant decrease in PKM2 protein expression in exosomes (Figure 5H).

These results indicate that hnRNPA2B1 promotes selective splicing of PKM2 and facilitates the packaging of PKM2 into microglia exosomes.

### 3.8 Downregulation of PKM2 in Microglial‐Derived Exosomes Reduces DLAT Expression and Copper‐Induced Neuronal Death in the Hippocampus

**FIGURE 6:**
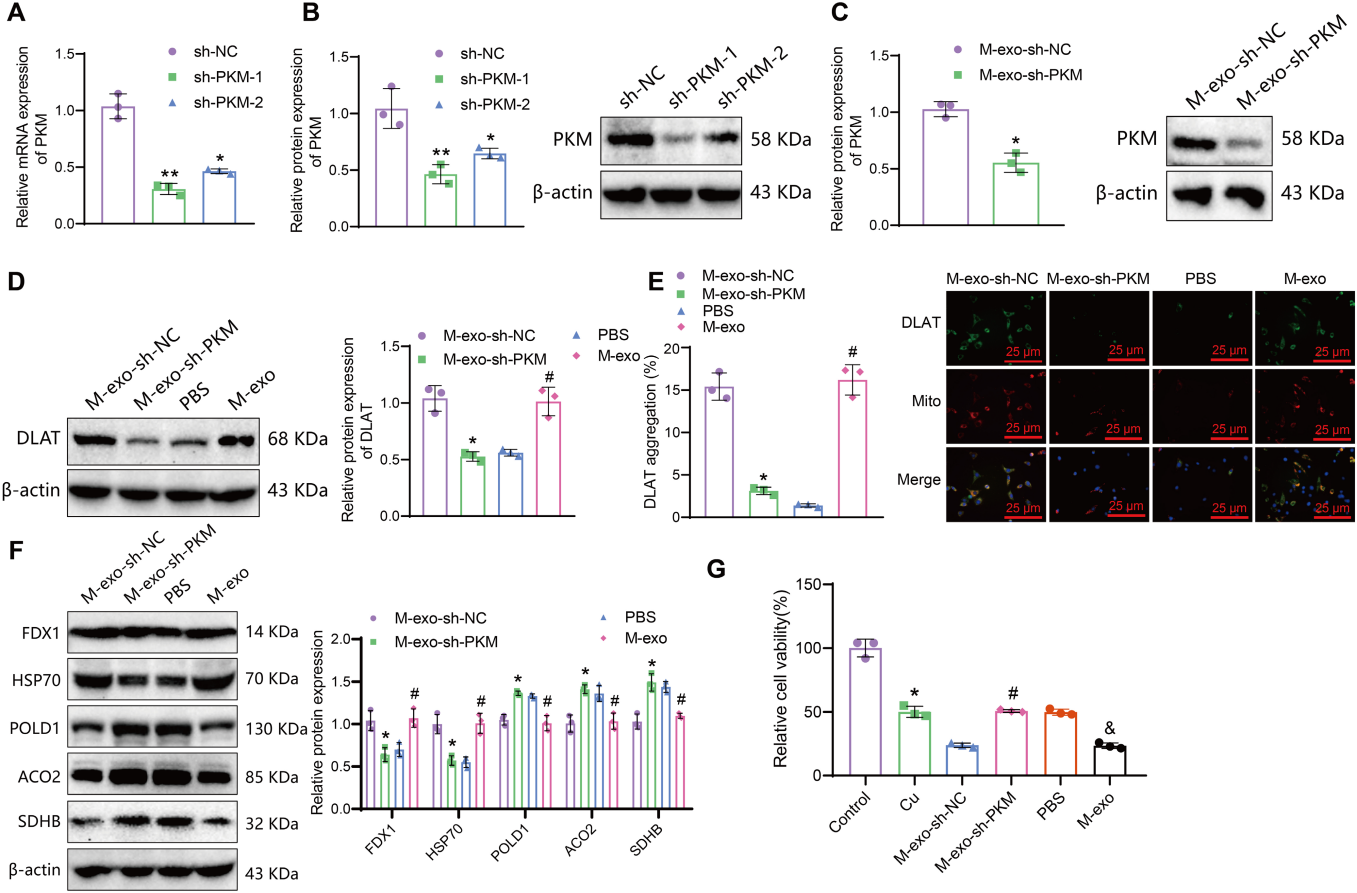
Effects of PKM2 Transfer via microglial extracellular vesicles on the expression of DLAT and copper‐induced cell death in hippocampal neurons. *Note:* (A) RT‐qPCR was used to measure the mRNA expression of PKM in microglial cells after knockdown, and the results show a significant difference compared with the sh‐NC group at *p* < 0.05 and *p* < 0.01. (B) Western blot was performed to examine the protein expression of PKM2 in microglial cells after knockdown, and the results show a significant difference compared with the sh‐NC group at *p* < 0.05 and *p* < 0.01. (C) Western blot was performed to examine the protein expression of PKM2 in hippocampal neurons after co‐cultivation with M‐exo obtained from knockdown PKM2 microglial cells. (D) Western blot was performed to measure the protein expression of DLAT in hippocampal neurons after co‐cultivation with M‐exo from different treatment groups. (E) Immunofluorescence staining was used to detect the aggregation of DLAT in the mitochondria of hippocampal neurons (25 μm). Green fluorescence represents DLAT, and red fluorescence represents mitochondria. (F) Western blot was performed to measure the expression of copper‐induced cell death‐related proteins in different groups of hippocampal neurons. (G) CCK‐8 assay to assess the relative cell viability of hippocampal neurons in different treatment groups. In panels C‐F, * indicates *p* < 0.05 compared to the M‐exo‐sh‐NC group, # indicates *p* < 0.05 compared to the PBS group; in panel G, * indicates *p* < 0.05 compared to the Control group, # indicates *p* < 0.05 compared to the M‐exo‐sh‐NC group, & indicates *p* < 0.05 compared to the PBS group. Cell experiments were repeated 3 times.

We further investigated the impact of exosomal transfer of PKM2 in the extracellular matrix of oligodendrocytes on neuronal death. Downregulate PKM2 in microglia cells and select sh‐PKM‐1 (sh‐PKM), which has a better downregulation efficiency, for further experiments (Figure 6A,B). Co‐culture of M‐exo with hippocampal neurons was performed, and the results demonstrated that compared to the M‐exo‐sh‐NC group, the expression of PKM2 in hippocampal neurons was decreased in the M‐exo‐sh‐PKM group (Figure 6C). Compared with the PBS group, the expression of DLAT protein in hippocampal neurons was increased in the M‐exo group, while compared with the M‐exo‐sh‐NC group, the expression of DLAT protein in hippocampal neurons was reduced in the M‐exo‐sh‐PKM group (Figure 6D).

Research reports suggest that a hallmark of copper death is the aggregation of mitochondrial acyltransferase proteins such as DLAT [15]. Therefore, we used immunofluorescence staining to detect the aggregation of DLAT. The results showed that compared to the PBS group, the DLAT aggregation in the mitochondria of the M‐exo group increased. On the other hand, compared to the M‐exo‐sh‐NC group, the DLAT aggregation in the mitochondria of the M‐exo‐sh‐PKM group decreased (Figure 6E). Western blot was used to detect the expression of copper death‐related proteins. The results showed that compared with the PBS group, the M‐exo group exhibited increased FDX1 and HSP70 proteins and decreased expression of Fe‐S cluster proteins POLD1, ACO2, and SDHB. Compared with the M‐exo‐sh‐NC group, the M‐exo‐sh‐PKM group showed decreased expression of FDX1 and HSP70 proteins and increased expression of Fe‐S cluster proteins POLD1, ACO2, and SDHB (Figure 6F).

Subsequent co‐incubation of hippocampal neurons treated with 80 μM CuSO4 with M‐exo showed a marked decrease in relative cell survival rates post‐Cu treatment. A decrease in relative cell survival rates was noted in the M‐exo group compared to the PBS group, while an increase was observed in the M‐exo‐sh‐PKM group compared to the M‐exo‐sh‐NC group (Figure 6G).

The above results indicate that extracellular vesicles derived from microglia could transfer PKM2 to promote DLAT expression and copper‐induced neuronal death in the hippocampus.

### 3.9 Microglial Exosomal PKM2 Drives DLAT Expression and Neuronal Copper Death via Protein–Protein Interaction

**FIGURE 7:**
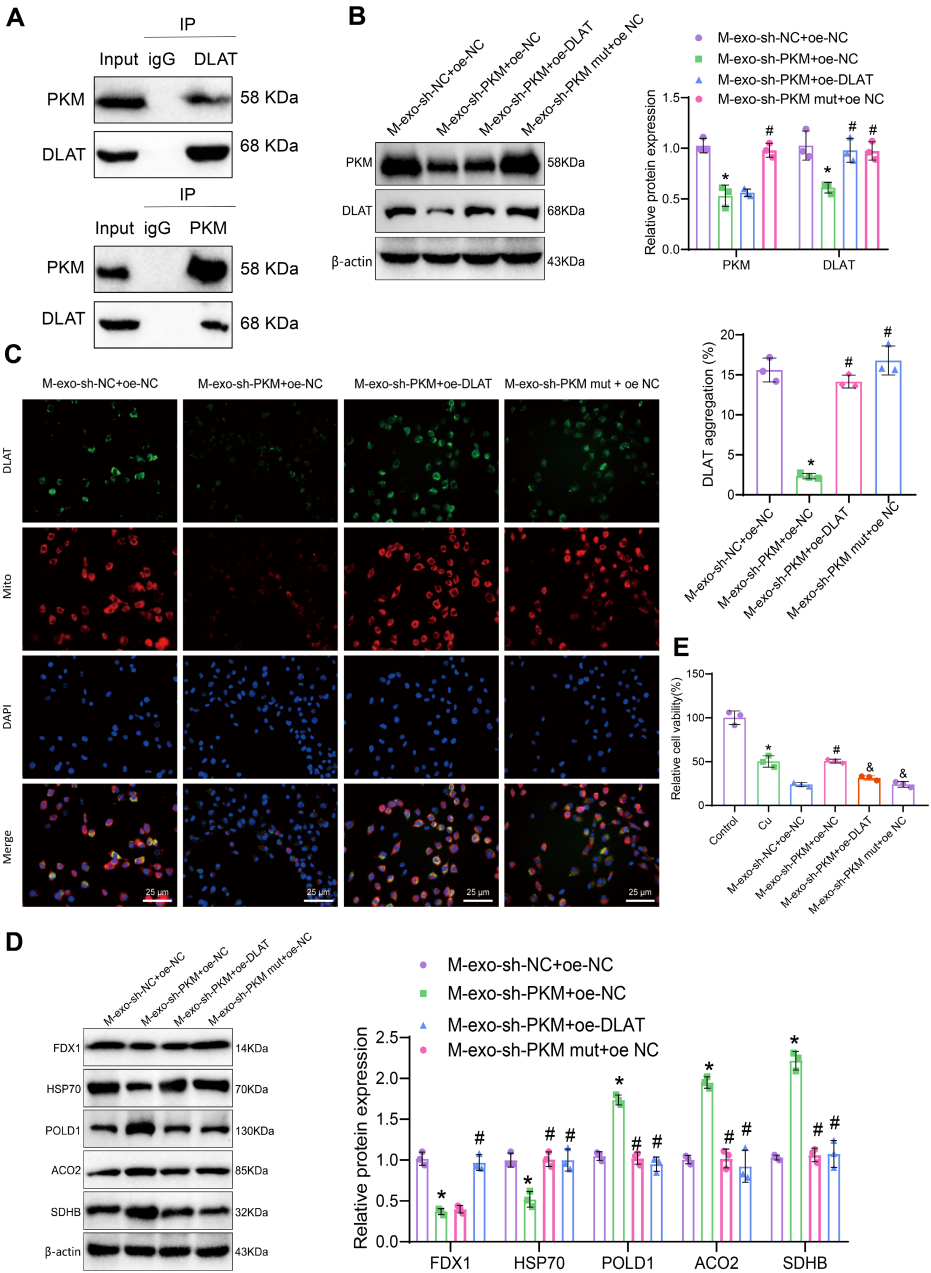
Effects of PKM2‐mediated DLAT expression via microglial extracellular vesicles on copper‐induced cell death in hippocampal neurons. *Note:* (A) Co‐immunoprecipitation experiments were performed to examine the interaction between PKM2 and DLAT. (B) Western blot was conducted to measure the protein expression of PKM2 and DLAT in different groups of hippocampal neurons. (C) Immunofluorescence staining was used to detect the aggregation of DLAT in the mitochondria of hippocampal neurons (25 μm). Green fluorescence represents DLAT, and red fluorescence represents mitochondria. (D) Western blot was performed to measure the expression of copper‐induced cell death‐related proteins in different groups of hippocampal neurons. (E) CCK‐8 assay was conducted to determine the relative cell survival rate of different groups of hippocampal neurons. In Figures (A–D), * indicates a significance level of *p* < 0.05 compared to the M‐exo‐sh‐NC + oe‐NC group, while # indicates a significance level of *p* < 0.05 compared to the M‐exo‐sh‐PKM + oe‐NC group. In (E), * denotes a significance level of *p* < 0.05 compared to the Control group, # indicates a significance level of *p* < 0.05 compared to the M‐exo‐sh‐NC + oe‐NC group, and & indicates a significance level of *p* < 0.05 compared to the M‐exo‐sh‐PKM + oe‐NC group. The cell experiments were repeated three times.

To further investigate the transfer of PKM2 via exosomes from microglia and its effects on neuronal cell death by regulating DLAT expression, we first validated the protein–protein interaction between PKM2 and DLAT through co‐IP experiments. The results showed a protein–protein interaction between PKM2 and DLAT (Figure 7A). Moreover, compared to the M‐exo‐sh‐PKM + oe‐NC group, the M‐exo‐sh‐PKM + oe‐DLAT group showed a significant increase in DALT expression in hippocampal neurons (Figure 7B). Immunofluorescent staining showed that compared with the M‐exo‐sh‐NC + oe‐NC group, there was a reduction in DLAT aggregation in the mitochondria of the M‐exo‐sh‐PKM + oe‐NC group. Additionally, there was a significant increase in DLAT aggregation in mitochondria in the M‐exo‐sh‐PKM mut + oe NC group compared to the M‐exo‐sh‐PKM + oe‐NC group. In contrast, compared with the M‐exo‐sh‐PKM + oe‐NC group, there was an increase in DLAT aggregation in the mitochondria of the M‐exo‐sh‐PKM + oe‐DLAT group (Figure 7C). The expression analysis of copper‐death‐related proteins revealed the following trends: compared to the M‐exo‐sh‐NC + oe‐NC group, the M‐exo‐sh‐PKM + oe‐NC group exhibited a significant decrease in the expression of FDX1 and HSP70 proteins, while the expression of Fe‐S cluster proteins POLD1, ACO2, and SDHB was notably increased. Moreover, the M‐exo‐sh‐PKM mut + oe NC group showed a significant increase in the expression of FDX1 and HSP70 proteins compared to the M‐exo‐sh‐PKM + oe‐NC group, with a corresponding decrease in the expression of Fe‐S cluster proteins POLD1, ACO2, and SDHB. In comparison to the M‐exo‐sh‐PKM + oe‐NC group, the M‐exo‐sh‐PKM + oe‐DLAT group displayed a marked increase in HSP70 protein expression, a noticeable decrease in the expression of Fe‐S cluster proteins POLD1, ACO2, and SDHB, with no significant change in FDX1 expression (Figure 7D).

The CCK‐8 assay results showed that compared to the M‐exo‐sh‐NC + oe‐NC group, the hippocampal neuron cell viability was increased in the M‐exo‐sh‐PKM + oe‐NC group. Conversely, the relative cell survival rate of hippocampal neurons was significantly reduced in the M‐exo‐sh‐PKM mut + oe NC group compared to the M‐exo‐sh‐PKM + oe‐NC group. Furthermore, the relative cell survival rate of hippocampal neurons was decreased in the M‐exo‐sh‐PKM + oe‐DLAT group compared to the M‐exo‐sh‐PKM + oe‐NC group (Figure 7E). This indicates that mutation of the targeted PKM1 sequence, as in M‐exo‐sh‐PKM mut + oe NC, has a similar impact to M‐exo‐sh‐NC + oe‐NC, suggesting the specific influence of the PKM silencing sequence on PKM levels and regulation.

The above results indicate that exosomes from microglia could promote DLAT expression in hippocampal neurons by transmitting PKM2, leading to neuronal copper death.

### 3.10 Microglial‐Derived Extracellular Vesicles Mediate PKM2 Transfer to Regulate DLAT Expression, Neuronal Survival, and Cognitive Function in 5xFAD AD Mice

**FIGURE 8:**
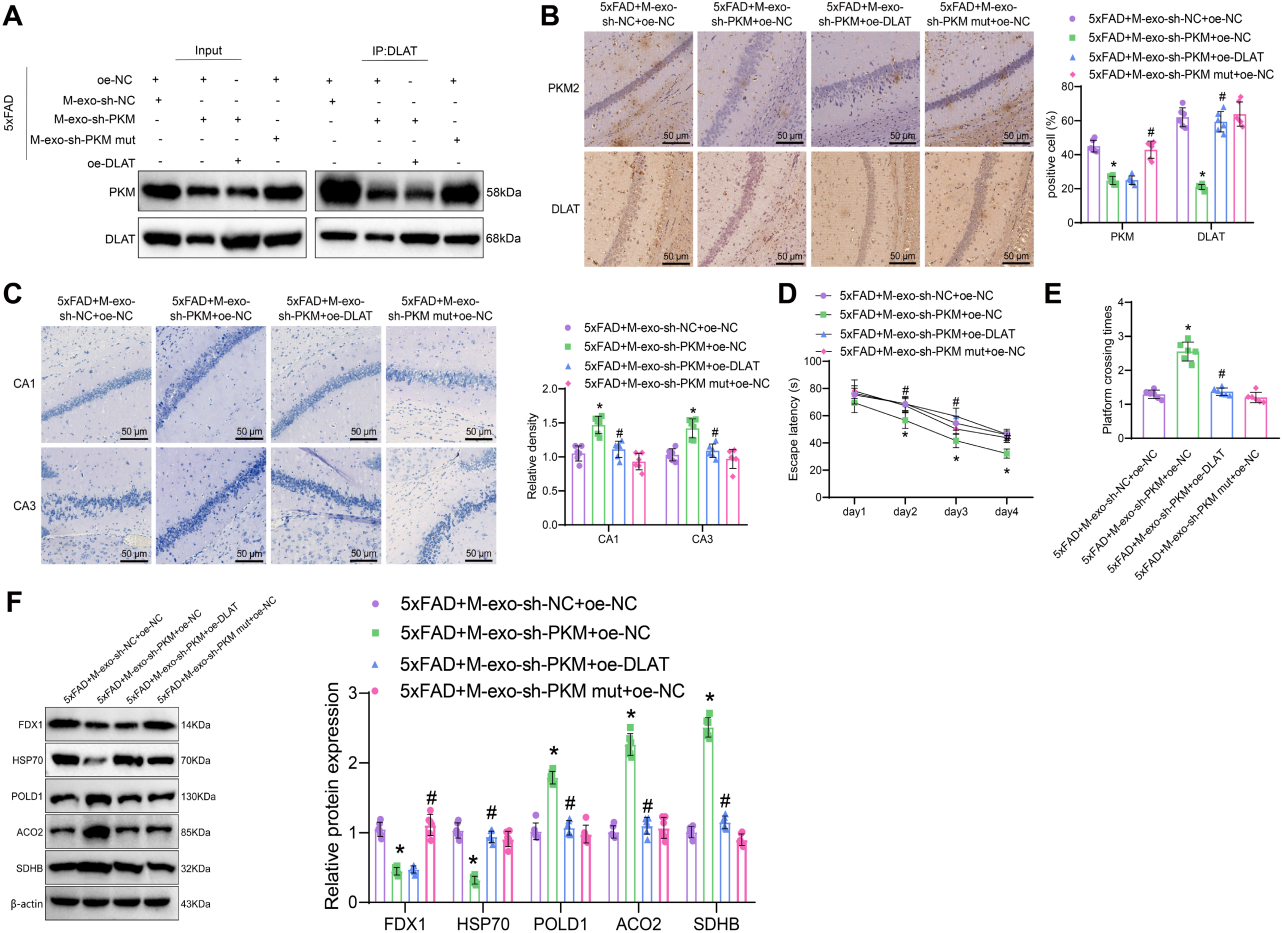
Microglial exosomes transfer PKM2 mediating neuronal copper‐induced death impacting AD progression. *Note:* (A) Co‐Immunoprecipitation assay to detect the protein–protein interaction of PKM2 and DLAT in the hippocampal tissue of each group of mice; (B) immunohistochemistry to assess the protein expression of PKM2 and DLAT in the hippocampal tissue of each group of mice (scale: 50 μm); (C) Nissl staining to evaluate neuronal damage in the CA1 and CA3 regions of the hippocampal tissue of WT and 5xFAD mice (scale: 50 μm); (D) escape latency of each group of mice in the Morris water maze test; (E) time taken by each group of mice to cross the platform in the Morris water maze test; (F) western blot analysis of the expression of Fe‐S cluster proteins and protein toxicity stress markers in the hippocampal tissue of each group of mice. * indicates comparison with the 5xFAD + M‐exo‐sh‐NC + oe‐NC group, *p* < 0.05; # indicates comparison with the 5xFAD + M‐exo‐sh‐PKM + oe‐NC group, *p* < 0.05, with 6 mice in each group.

Finally, we further validated in the 5xFAD transgenic mouse model the molecular mechanism of how extracellular vesicle‐mediated transfer of PKM2 by microglia affects neuronal copper‐induced death and the progression of AD. Initially, we confirmed the interaction between PKM2 and DLAT through co‐immunoprecipitation experiments (Figure 8A). Subsequently, immunohistochemistry was performed to assess the expression of PKM2 and DLAT in the hippocampus of the mouse groups. It was observed that compared to the 5xFAD + M‐exo‐sh‐NC + oe‐NC group, the expression of PKM2 and DLAT1 was significantly decreased in the hippocampal tissue of the 5xFAD + M‐exo‐sh‐PKM + oe‐NC group. Conversely, in the 5xFAD + M‐exo‐sh‐PKM mut + oe NC group, there was a marked increase in the expression of PKM2 and DLAT1 compared to the 5xFAD + M‐exo‐sh‐PKM + oe‐NC group. Furthermore, when comparing the 5xFAD + M‐exo‐sh‐PKM + oe‐NC group with the 5xFAD + M‐exo‐sh‐PKM + oe‐DLAT group, a notable increase in DLAT1 expression was observed in the latter group (Figure 8B). The Nissl staining results showed that compared with the 5xFAD + M‐exo‐sh‐NC + oe‐NC group, the Nissl bodies in the hippocampal tissue of the 5xFAD + M‐exo‐sh‐PKM + oe‐NC group mice were increased, indicating a reduction in hippocampal neuronal damage. In comparison, the Nissl bodies in the hippocampal tissue of mice were significantly reduced in the 5xFAD + M‐exo‐sh‐PKM mut + oe NC group compared to the 5xFAD + M‐exo‐sh‐PKM + oe‐NC group, indicating a notable increase in hippocampal neuronal damage. Compared with the 5xFAD + M‐exo‐sh‐PKM + oe‐NC group, the Nissl bodies in the hippocampal tissue of the 5xFAD + M‐exo‐sh‐PKM + oe‐DLAT group mice were decreased, indicating an increase in hippocampal neuronal damage (Figure 8C). The Morris water maze experiment results showed that compared with the 5xFAD + M‐exo‐sh‐NC + oe‐NC group, the escape latency of mice in the 5xFAD + M‐exo‐sh‐PKM + oe‐NC group was reduced, and the time to cross the platform was increased. In comparison, the escape latency of mice showed a significant increase in the 5xFAD + M‐exo‐sh‐PKM mut + oe NC group compared to the 5xFAD + M‐exo‐sh‐PKM + oe‐NC group, while the time taken to cross the platform was notably reduced. Compared with the 5xFAD + M‐exo‐sh‐PKM + oe‐NC group, the escape latency of mice in the 5xFAD + M‐exo‐sh‐PKM + oe‐DLAT group was increased, and the time to cross the platform was reduced (Figure 8D,E).

In the analysis through Western blot for copper death‐related protein expression, the results revealed the following: in comparison to the 5xFAD + M‐exo‐sh‐NC + oe‐NC group, the hippocampal tissue of mice in the 5xFAD + M‐exo‐sh‐PKM + oe‐NC group showed a significant decrease in FDX1 and HSP70 protein expression, while the expression of Fe‐S cluster proteins POLD1, ACO2, and SDHB was notably increased. Conversely, the 5xFAD + M‐exo‐sh‐PKM mut + oe NC group exhibited a significant increase in FDX1 and HSP70 protein expression, and a decrease in Fe‐S cluster proteins POLD1, ACO2, and SDHB expression compared to the 5xFAD + M‐exo‐sh‐PKM + oe‐NC group. Furthermore, when compared to the 5xFAD + M‐exo‐sh‐PKM + oe‐NC group, the 5xFAD + M‐exo‐sh‐PKM + oe‐DLAT group displayed a significant increase in HSP70 protein expression, alongside a notable decrease in Fe‐S cluster proteins POLD1, ACO2, and SDHB expression, while FDX1 protein expression showed no significant changes (Figure 8F). The findings suggest that mutating the effective target site of PKM1 to 5xFAD + M‐exo‐sh‐PKM mut + oe NC results in similar impacts compared to 5xFAD + M‐exo‐sh‐NC + oe‐NC, indicating that the silencing sequence of PKM has a specific influence on PKM levels and regulation.

The results indicate that extracellular vesicles derived from microglial cells mediate the transfer of sh‐PKM, downregulate DLAT expression, and inhibit copper‐induced cell death, thereby alleviating cognitive impairment in AD mice.

## 4 Discussion

**FIGURE 9:**
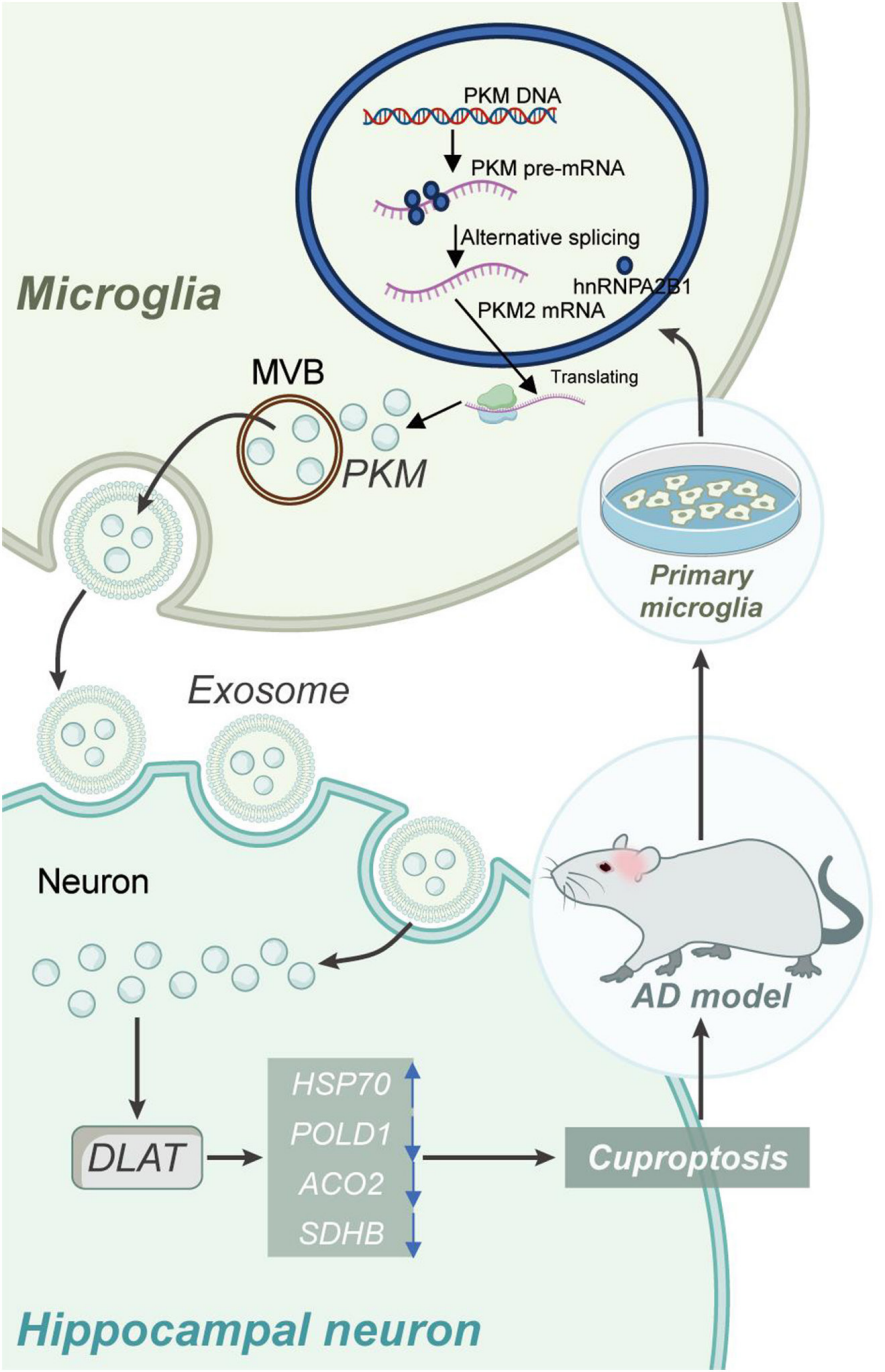
Schematic representation of the molecular mechanism by which extracellular vesicles derived from microglia deliver PKM2 to regulate DLAT expression, influencing neuronal copper‐induced death and AD progression.

In conclusion, our findings suggest that microglia‐derived extracellular vesicles can deliver PKM2 to hippocampal neurons, upregulate DLAT protein expression, leading to copper‐mediated neuronal death in the hippocampus, and exacerbate cognitive dysfunction in AD mice (Figure 9). AD is a severe neurodegenerative disorder, and current treatment options offer limited therapeutic benefits, highlighting the urgent need for innovative research breakthroughs [65, 66, 67]. This study has made groundbreaking discoveries in understanding the mechanisms of neuronal copper‐mediated death and the role of microglia‐derived extracellular vesicles in AD. Initially, our bioinformatics analysis identified DLAT as a potential key mediator of neuronal copper‐mediated death in AD. Subsequently, through animal experiments and cell studies, we validated that microglia‐derived extracellular vesicles promote DLAT expression by delivering PKM2, leading to copper‐mediated neuronal death and accelerating AD progression. This finding offers a new research avenue for the treatment of AD by inhibiting the expression of DLAT through blocking the transfer of PKM2 by microglia‐derived extracellular vesicles, potentially attenuating neuronal copper‐mediated death and slowing down AD progression [68]. Therefore, this study not only holds significant scientific value in deepening our understanding of AD but also possesses crucial clinical implications for the development of novel therapeutic strategies.

This study utilized snRNA‐seq analysis to reveal the potential interaction of microglial cells with neurons in AD. Our snRNA‐seq analysis results also indicate the communication between other cells in the brain tissue of AD mice, such as astrocytes and oligodendrocytes, with neurons. Research suggests that astrocytes and microglial cells are significant sources of neuronal exosomes during neurodegeneration processes [69]. Astrocytic exosomes impact neurodegenerative diseases through metabolic balance and ubiquitin‐dependent protein regulation, whereas exosomes from microglial cells influence neurodegeneration through immune inflammation and oxidative stress [70]. Our findings demonstrate a noticeable increase in the number of microglial cells in AD, along with enhanced interaction with neurons, leading us to hypothesize that microglial exosomes may play a more critical role in AD. Further analysis revealed 20 intersecting genes obtained by intersecting the differentially expressed genes in the sequencing dataset with genes expressed in microglial exosome as retrieved from the ExoCarta database. Protein–protein interaction analysis between these 20 intersecting genes and DLAT indicated a high expression of interacting proteins in microglial cells of AD mice. Notably, ATP1A3 and PKM showed more significant differential changes. Subsequently, using the PPA‐Pred website for prediction, we found a strong interaction between PKM and DLAT (Kd < 10‐8 M indicating a strong bond, Table S2). Subsequent in vitro experiments involving the secretion of microglia extracellular vesicles revealed the delivery of PKM2 protein to neurons. Further, Co‐IP experiments confirmed the interaction between PKM2 and DLAT, leading to an upregulation of DLAT protein expression, resulting in hippocampal neuron death and exacerbating cognitive dysfunction in AD mice. This finding underscores the significant role of microglia extracellular vesicles in the pathogenesis of AD, offering a potential avenue for novel therapeutic strategies. While previous research has highlighted the crucial role of neuroinflammation, particularly microglia activation, in AD, prior studies have predominantly focused on the cytokine secretion of microglia. This study, however, unveils for the first time the impact of microglia‐derived extracellular vesicles in AD [71]. Our experimental results demonstrate the pivotal roles of PKM2 and DLAT in copper‐induced neuronal death. It is suggested that PKM2 may influence neuronal copper ion levels and thereby alter neuron survival by regulating the expression of DLAT. This discovery not only illuminates the significant involvement of copper ions in neuronal survival but also presents novel potential drug targets for AD.

Copper is an essential element in cells, serving as an electron acceptor or donor in various reactions. Humans and other mammals primarily acquire copper ions from dietary sources, which are absorbed by intestinal cells before utilization within the body. However, extracellular copper ions exist in a divalent form and cannot be directly utilized by cells [72]. Therefore, divalent copper ions need to be catalyzed by the enzymes DCYTB, Steap 2, Steap 3, and Steap 4 to be reduced to Cu + before binding to Ctr1 (copper transporter 1) for transport into cells [73]. Nonetheless, the excessive accumulation of copper ions within cells can lead to harmful reactions. For instance, in AD, a significant buildup of copper ions has been observed in senile plaques composed of amyloid‐β (Aβ) peptide and neurofibrillary tangles (NFT), indicating a potential contributory factor to AD pathogenesis [74]. By analyzing expression profile data related to AD, this study reveals upregulation of DLAT in AD, and knocking down DLAT can ameliorate cognitive impairments in an AD mouse model. Furthermore, studies suggest that DLAT, as a protein with a lipoic acid group, readily binds to cuprous ions. Therefore, the upregulation of DLAT in AD may be a significant factor contributing to copper metabolism imbalance. Additionally, despite DLAT's involvement in numerous metabolic processes, its role in AD remains unclear [75, 76].

This study provides initial evidence that DLAT may be a key factor mediating neuronal copper death in AD, opening up new avenues for its treatment. Through in vitro and in vivo experiments, we observed that glial exosomes can transfer PKM2 to hippocampal neurons, affecting DLAT expression. Additionally, Co‐IP experiments revealed a direct interaction between PKM2 and DLAT. This interaction suggests a novel therapeutic strategy for AD by modulating the interaction between PKM2 and DLAT to alter neuronal copper levels and thereby influence neuronal survival. In vivo experiments showed that inhibiting glial exosome secretion of PKM2 downregulates DLAT expression, inhibiting neuronal copper death and consequently attenuating AD progression. This finding uncovers a potential link between glial exosomes and neuronal copper death, offering a fresh perspective on AD treatment.

This research unravels the molecular mechanisms underlying neuronal copper death in AD and the critical role played by glial exosomes at both in vivo animal and in vitro cellular levels. However, there are limitations. Clinical sample testing was not conducted due to constraints, highlighting the need to verify copper death and related factor expression changes in clinical samples from AD patients. Moreover, further investigations are required to delve into the mechanisms associated with copper death, aiming to provide potential targets for the diagnosis and treatment of clinical AD patients.

While our study has yielded promising findings, it also has limitations. Primarily reliant on animal models and cell experiments, our study, although capable of simulating human AD conditions, falls short in comparison to the complexity of the human body, potentially underestimating or overestimating the roles of certain mechanisms. Although our results demonstrate that glial exosomes transmit PKM2 and promote DLAT expression, the specific mechanisms of transmission and action require further study. Additionally, the data primarily sourced from the GEO database may impact the stability and reliability of the results. Factors such as environmental and genetic influences on AD progression were not taken into account.

Despite the limitations, our findings undoubtedly offer a new perspective for AD treatment. Future research should further explore the specific role of glial exosomes in AD, potentially through proteomic or RNA sequencing analysis to unveil their role in the AD network. Elucidating how PKM2 regulates DLAT to induce neuronal copper death requires further experimental validation. Ultimately, translating these laboratory research findings into clinical applications to provide more effective treatment for AD patients is crucial. Incorporating human samples and conducting more in‐depth clinical trials and long‐term follow‐up studies in future research will be essential. Overall, this study points toward new directions and potential targets for future AD research and treatment.

## Author Contributions

X.M., Y.S., Y.N., and J.H. designed the study. C.L., M.W., Q.Z., X.Z., and F.W. collated the data, carried out data analyses and produced the initial draft of the manuscript. X.M., Y.S., Y.N., and J.H. contributed to drafting the manuscript. All authors have read and approved the final submitted manuscript.

## Ethics Statement

The Ethics Committee has approved all animal experimental procedures for Animal Experiments of Taiyuan Institute of Technology.

## Conflicts of Interest

The authors declare no conflicts of interest.

## Funding

This study was supported by the Fundamental Research Program of Shanxi Province (Grants 202303021211194 and 20210302124333), Fund for Shanxi “1331” Project, Program for the (Reserver) Discipline Leaders of Taiyuan Institute of Technology.

## Supporting information


**Figure S1.** Aschematic diagram of the grouping scheme for animal experiments.

**Figure S2.** A schematic representation of the grouping scheme for cell experiments.

**Figure S3** Genes related to copper‐induced cell death are involved in the progression of AD. Note: (A) Volcano plot showing differentially expressed genes between normal control blood samples (Normal group, *n* = 10) and AD blood samples (AD group, *n* = 9) in the GSE97760 dataset; (B) heatmap of the top 50 differentially expressed genes between normal control blood samples (Normal group, n = 10) and AD blood samples (AD group, *n* = 9) in the GSE97760 dataset; (C) Venn diagram showing the intersection between differentially expressed genes in the GSE97760 dataset and genes related to copper‐induced cell death; (D) boxplots of the differential expression of 11 intersecting genes related to copper‐induced cell death in the GSE97760 dataset, * represents *p* < 0.05 compared to the Normal group, ** represents *p* < 0.01 compared to the Normal group; Normal group, *n* = 10, AD group, *n* = 9.

**Figure S4** Key gene selection for copper‐induced neuronal death in AD. Note: (A) Lasso regression analysis curve (left), different colored curves represent different genes; cross‐validation error curve for lasso regression (right), the x‐axis represents log(λ) values, the y‐axis represents binomial deviance, the dots above indicate the number of genes retained when calculating at the corresponding log(λ) value, the dashed line represents the log(λ) value and the number of genes retained when achieving the minimum Binomial Deviance; (B) SVM‐RFE analysis results, the lowest point represents the optimal number of genes; (C) Venn diagram showing the intersection of lasso and SVM‐RFE analysis results; (D) Protein–protein interaction network of the 11 intersecting copper‐induced cell death genes (left) and ranking of their core degrees (right), the color gradient from red to blue indicates decreasing core degree; (E) GO enrichment analysis results for the 11 intersecting copper‐induced cell death genes; (F) KEGG pathway enrichment analysis results for the 11 intersecting copper‐induced cell death genes. Circle size in panels (E) and (F) represents the number of enriched genes; larger circles indicate more enriched genes, and color represents significance; the redder, the more significant.

**Figure S5** Principal component analysis and batch correction of snRNA‐seq Data. Note: (A) Distribution of cells in PC1 and PC2 before batch correction, with each point representing a cell; (B) graph depicting the batch correction process using Harmony, with the x‐axis indicating the number of interaction iterations; (C) distribution of cells in PC1 and PC2 after batch correction with Harmony, where each point represents a cell; (D) Distribution of standard deviations of PCs, with important PCs having larger standard deviations.

**Figure S6** Cell communication between various cells in WT group and AD group. Note: The thickness of the lines in the left diagram represents the number of pathways, while the thickness of the lines in the right diagram represents the strength of interaction.

**Figure S7** Identification of primary microglia and hippocampal neurons. Note: (A) Immunofluorescence staining to detect the level of activated microglia in the hippocampal tissues of each group of mice (100 μm), Iba1 is a marker for microglia, CD68 is a marker for lysosomes, white arrows indicate activated microglia; (B) Immunofluorescence staining to identify primary microglia and hippocampal neurons (25 μm), red fluorescence Iba‐1 represents microglia, green fluorescence MAP‐2 represents neuronal dendrites, blue fluorescence DAPI represents cell nuclei; (C) Western blot detection of PKM2 protein expression in microglia of WT and 5xFAD mice groups, * indicates *p* < 0.05 compared to the WT group.

**Figure S8** Identification of microglial exosomes. Note: (A) Transmission electron microscopy analysis of the morphological characteristics of WT and 5xFAD group M‐exos (100 nm); (B) NTA measurement of the particle size of WT and 5xFAD group M‐exos; (C) Western blot detection of the expression of exosomal positive markers CD81, CD9, Alix, and negative markers HSP90, histone H3 in WT and 5xFAD group exosomes, M: microglial cells.

Table S1
Table S2
Table S3
Table S4

## Data Availability

The data underlying this article will be shared on reasonable request to the corresponding author.
