# Supplementary figures and images for: Novel Insights Into DLAT's Role in Alzheimer's Disease‐Related Copper Toxicity Through Microglial Exosome Dynamics
Source: CNS Neurosci Ther. 2024 Oct 20;30(10):e70064. doi: 10.1111/cns.70064 (PMC11491298; doi:10.1111/cns.70064)

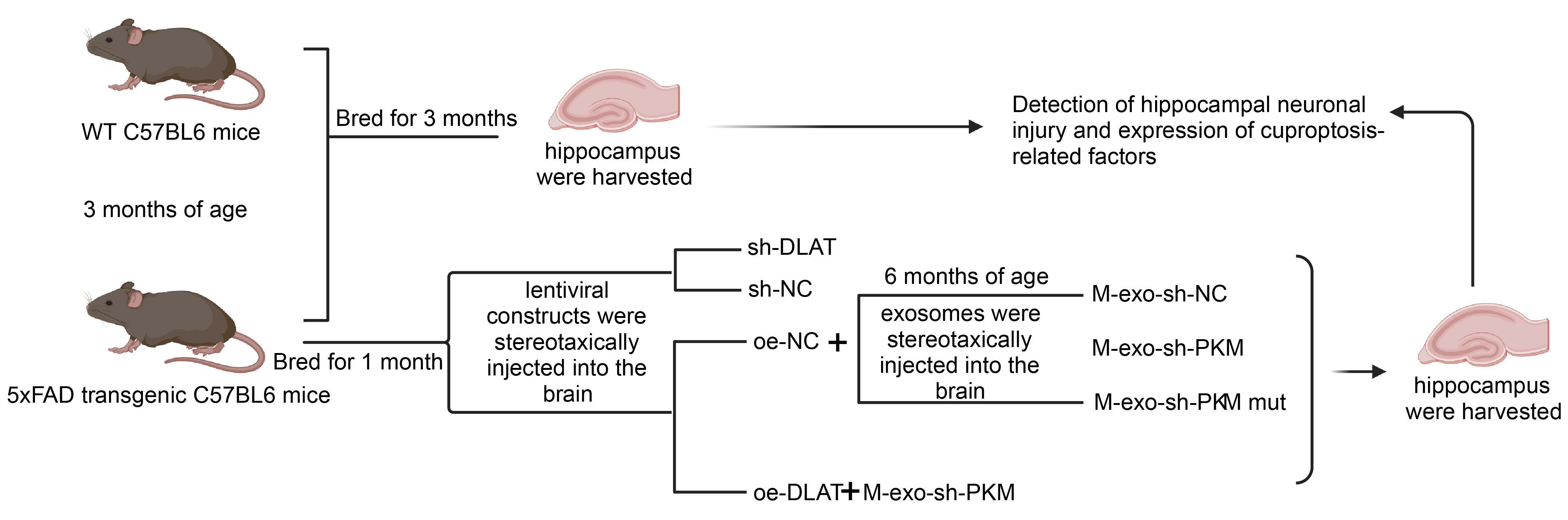

Supplement: Supplementary file 1 — Figure S1. Aschematic diagram of the grouping scheme for animal experiments. [file CNS-30-e70064-s002.jpg]

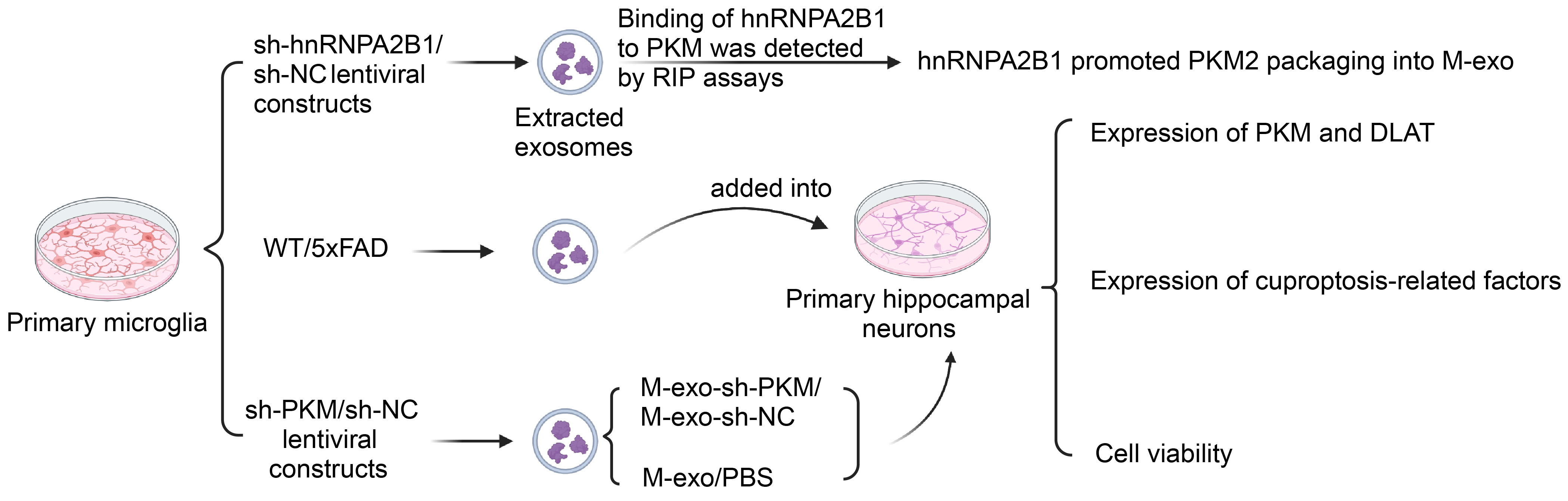

Supplement: Supplementary file 2 — Figure S2. A schematic representation of the grouping scheme for cell experiments. [file CNS-30-e70064-s003.jpg]

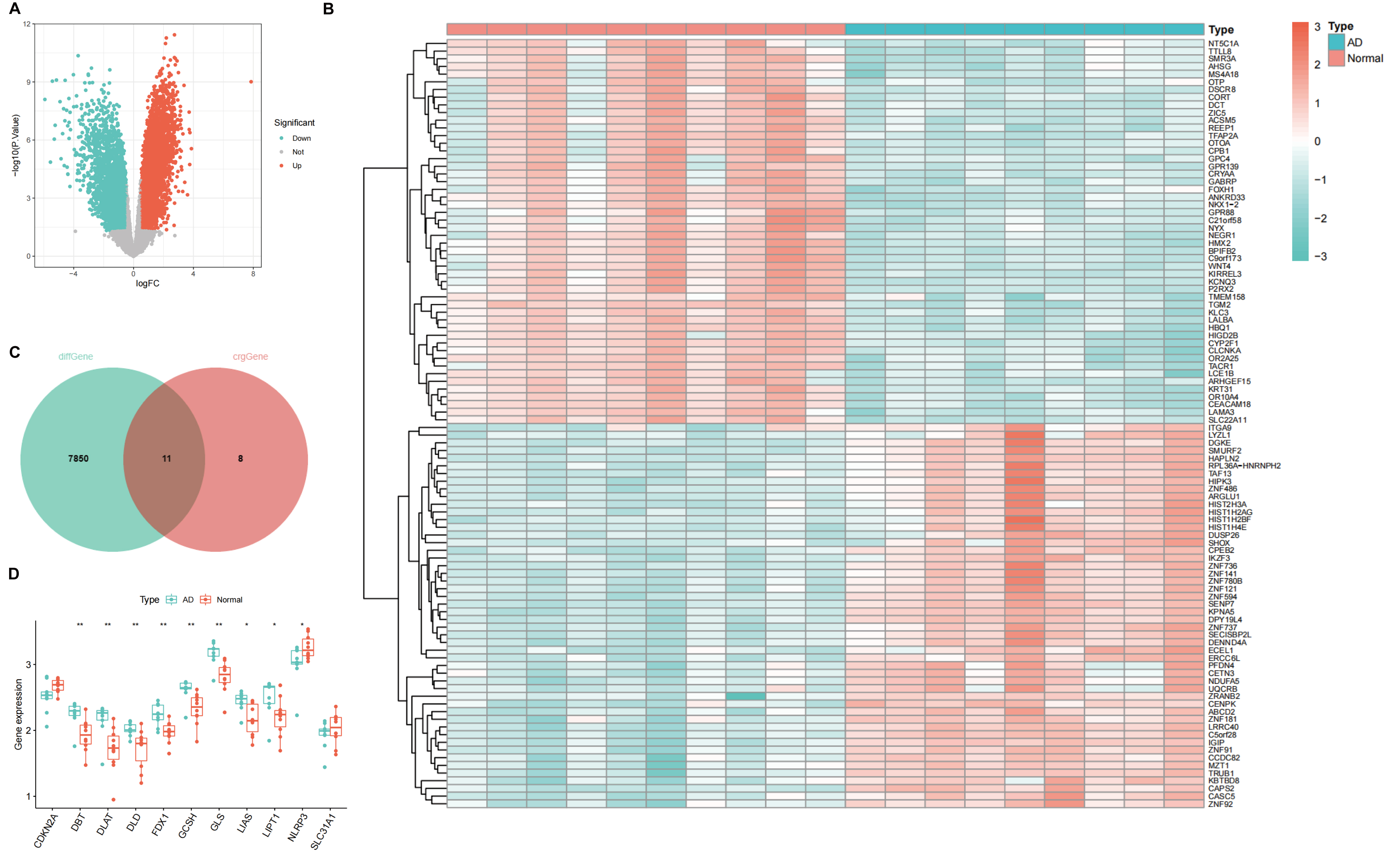

Supplement: Supplementary file 3 — Figure S3 Genes related to copper‐induced cell death are involved in the progression of AD. Note: (A) Volcano plot showing differentially expressed genes between normal control blood samples (Normal group, n = 10) and AD blood samples (AD group, n = 9) in the GSE97760 dataset; (B) heatmap of the top 50 differentially expressed genes between normal control blood samples (Normal group, n = 10) and AD blood samples (AD group, n = 9) in the GSE97760 dataset; (C) Venn diagram showing the intersection between differentially expressed genes in the GSE97760 dataset and genes related to copper‐induced cell death; (D) boxplots of the differential expression of 11 intersecting genes related to copper‐induced cell death in the GSE97760 dataset, * represents p < 0.05 compared to the Normal group, ** represents p < 0.01 compared to the Normal group; Normal group, n = 10, AD group, n = 9. [file CNS-30-e70064-s007.jpg]

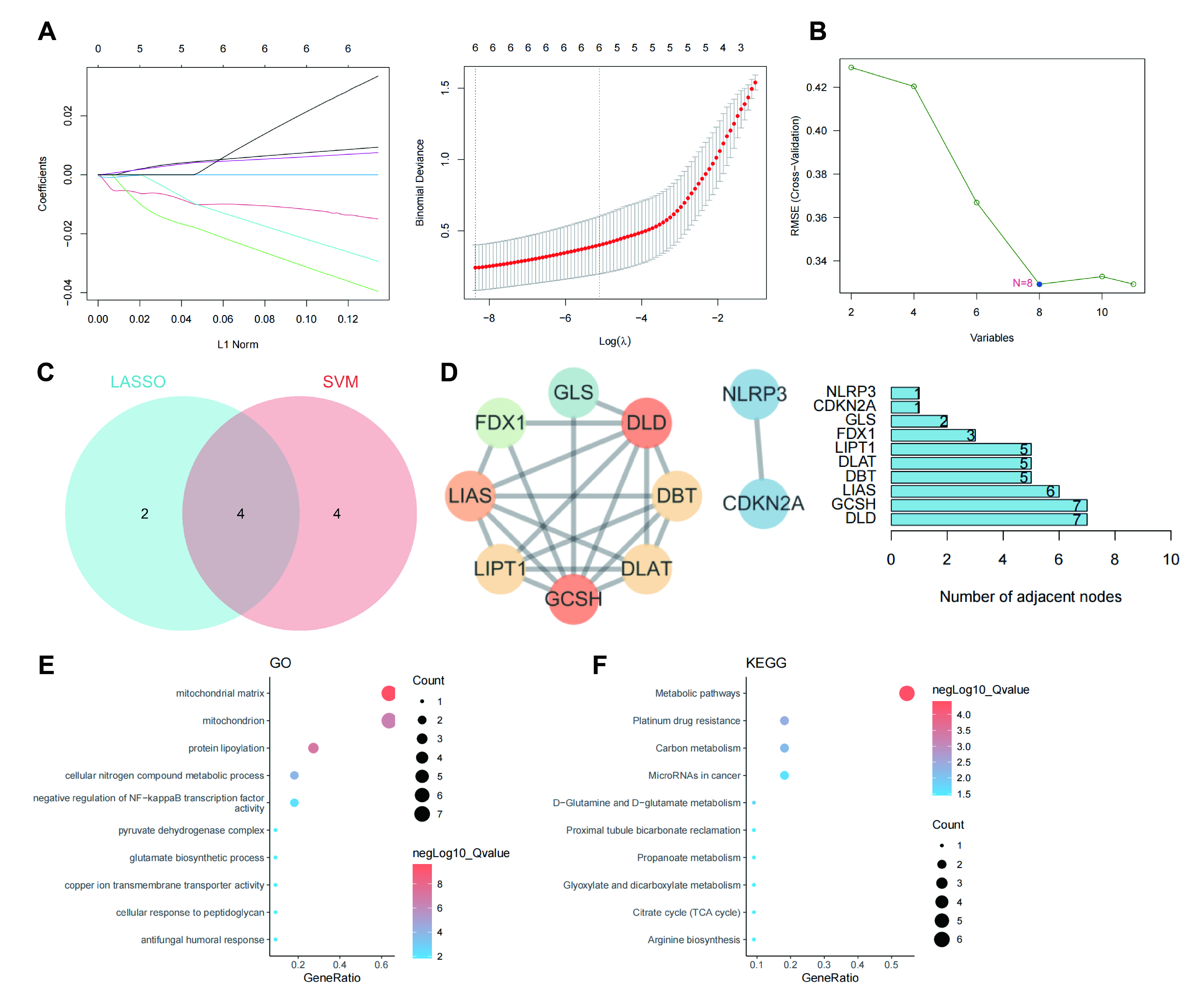

Supplement: Supplementary file 4 — Figure S4 Key gene selection for copper‐induced neuronal death in AD. Note: (A) Lasso regression analysis curve (left), different colored curves represent different genes; cross‐validation error curve for lasso regression (right), the x‐axis represents log(λ) values, the y‐axis represents binomial deviance, the dots above indicate the number of genes retained when calculating at the corresponding log(λ) value, the dashed line represents the log(λ) value and the number of genes retained when achieving the minimum Binomial Deviance; (B) SVM‐RFE analysis results, the lowest point represents the optimal number of genes; (C) Venn diagram showing the intersection of lasso and SVM‐RFE analysis results; (D) Protein–protein interaction network of the 11 intersecting copper‐induced cell death genes (left) and ranking of their core degrees (right), the color gradient from red to blue indicates decreasing core degree; (E) GO enrichment analysis results for the 11 intersecting copper‐induced cell death genes; (F) KEGG pathway enrichment analysis results for the 11 intersecting copper‐induced cell death genes. Circle size in panels (E) and (F) represents the number of enriched genes; larger circles indicate more enriched genes, and color represents significance; the redder, the more significant. [file CNS-30-e70064-s008.jpg]

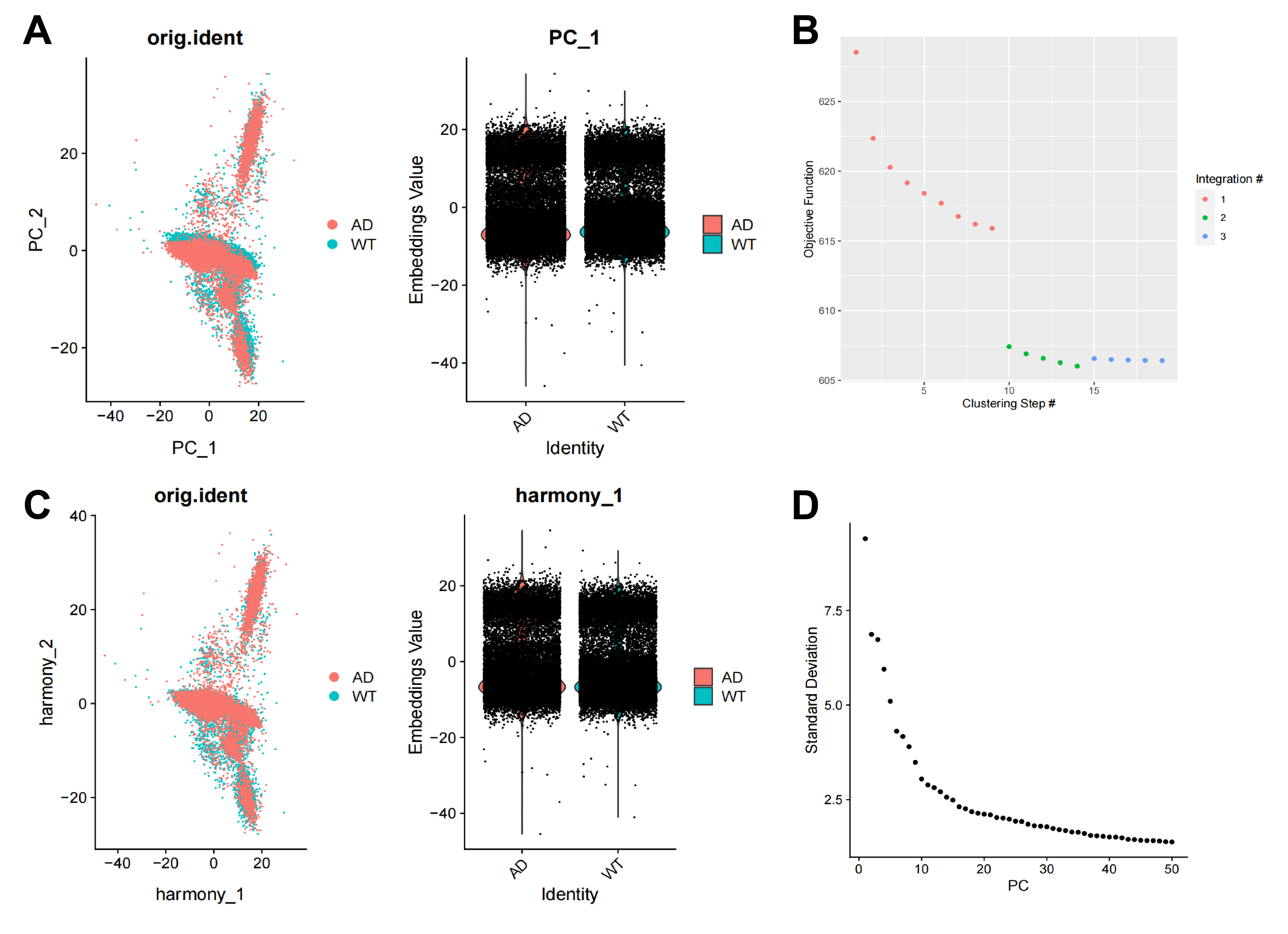

Supplement: Supplementary file 5 — Figure S5 Principal component analysis and batch correction of snRNA‐seq Data. Note: (A) Distribution of cells in PC1 and PC2 before batch correction, with each point representing a cell; (B) graph depicting the batch correction process using Harmony, with the x‐axis indicating the number of interaction iterations; (C) distribution of cells in PC1 and PC2 after batch correction with Harmony, where each point represents a cell; (D) Distribution of standard deviations of PCs, with important PCs having larger standard deviations. [file CNS-30-e70064-s004.jpg]

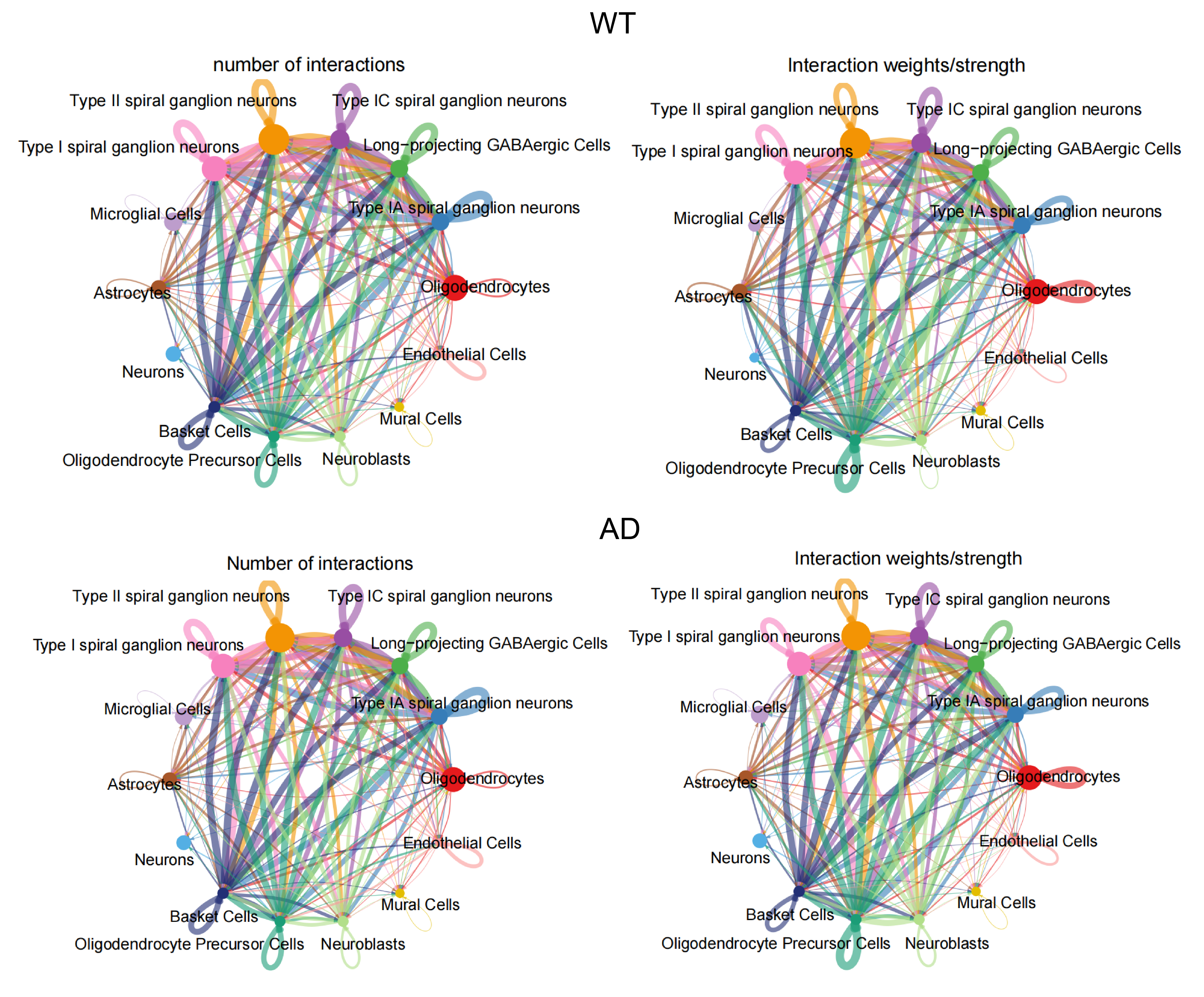

Supplement: Supplementary file 6 — Figure S6 Cell communication between various cells in WT group and AD group. Note: The thickness of the lines in the left diagram represents the number of pathways, while the thickness of the lines in the right diagram represents the strength of interaction. [file CNS-30-e70064-s009.jpg]

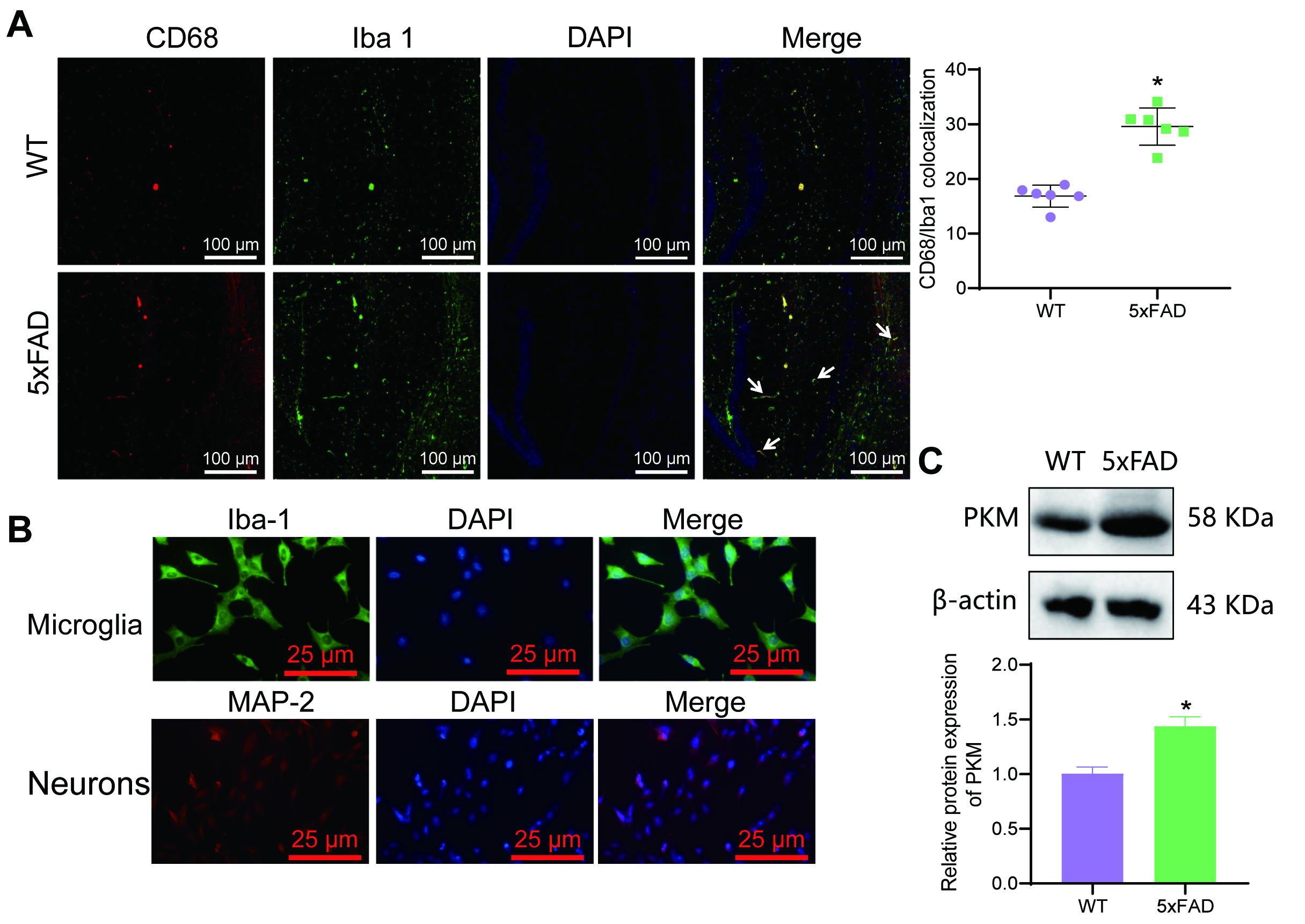

Supplement: Supplementary file 7 — Figure S7 Identification of primary microglia and hippocampal neurons. Note: (A) Immunofluorescence staining to detect the level of activated microglia in the hippocampal tissues of each group of mice (100 μm), Iba1 is a marker for microglia, CD68 is a marker for lysosomes, white arrows indicate activated microglia; (B) Immunofluorescence staining to identify primary microglia and hippocampal neurons (25 μm), red fluorescence Iba‐1 represents microglia, green fluorescence MAP‐2 represents neuronal dendrites, blue fluorescence DAPI represents cell nuclei; (C) Western blot detection of PKM2 protein expression in microglia of WT and 5xFAD mice groups, * indicates p < 0.05 compared to the WT group. [file CNS-30-e70064-s001.jpg]

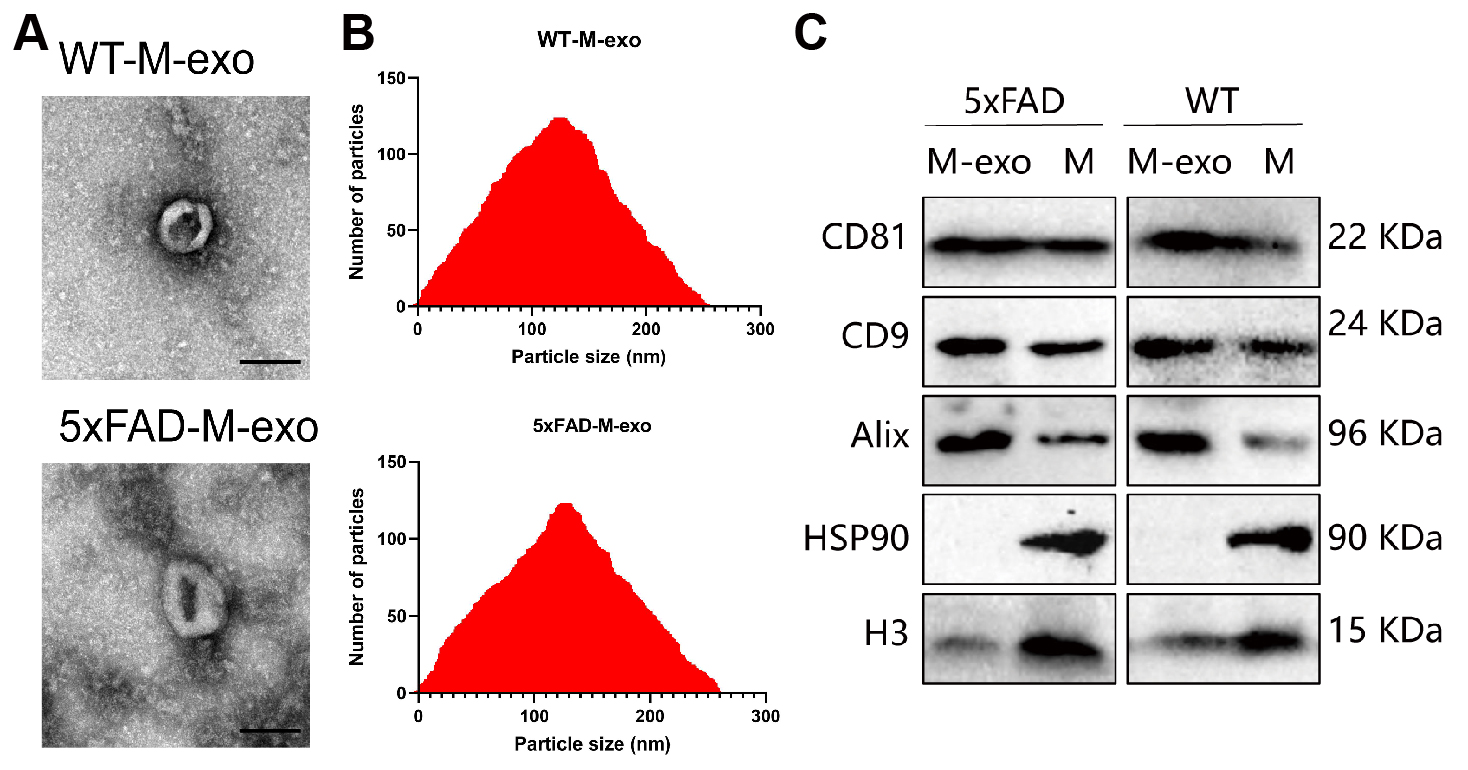

Supplement: Supplementary file 8 — Figure S8 Identification of microglial exosomes. Note: (A) Transmission electron microscopy analysis of the morphological characteristics of WT and 5xFAD group M‐exos (100 nm); (B) NTA measurement of the particle size of WT and 5xFAD group M‐exos; (C) Western blot detection of the expression of exosomal positive markers CD81, CD9, Alix, and negative markers HSP90, histone H3 in WT and 5xFAD group exosomes, M: microglial cells. [file CNS-30-e70064-s006.jpg]
