# Supplementary material for: Novel Insights Into DLAT's Role in Alzheimer's Disease‐Related Copper Toxicity Through Microglial Exosome Dynamics
Source: CNS Neurosci Ther. 2024 Oct 20;30(10):e70064. doi: 10.1111/cns.70064 (PMC11491298; doi:10.1111/cns.70064)
Supplement: Supplementary file 9 — Table S1 Table S2 Table S3 Table S4 [file CNS-30-e70064-s005.docx]

**Table S1. qRT-PCR primer sequences**

| Gene | primer sequences |
| --- | --- |
| PKM | F 5’-CATTACCAGCGACCCCACAG-3’ |
| Verified | R 5’-CTCCTGCCAGACTTGGTGAG-3’ |
| DLAT | F 5’-TGGCAAAAATCCTGGTCCCT-3’ |
| Verified | R 5’-TGGTCACTTCTGTTGGCCTG-3’ |
| hnRNPA2B1 | F 5’-GATACGGAAGTGGACGTGGA-3’ |
| Verified | R 5’-AGGGCTACCTCCAAAATTGCC-3’ |
| β-actin | F 5′-GATATCGCTGCGCTGGTCG-3′ |
| Verified | R 5′-CATTCCCACCATCACACCCT-3′ |
| PKM1 ex9 | F 5′-TTCGCATGCAGCACCTGATA-3′ |
| Verified | R 5′-GCAATGATAGGAGCCCGAGG-3′ |
| PKM2 ex10 | F 5′-GTCACTCCACAGACCTCATGG-3′ |
| Verified | R 5′-GCAATGATAGGAGCCCGAGG-3′ |

NOTE: F: Forward; R: Reverse.

**Table S2. The log2FC values and corresponding p-values of all differentially expressed genes**

| id | logFC | AveExpr | t | P.Value | adj.P.Val | B |
| --- | --- | --- | --- | --- | --- | --- |
| ZNF770 | 2.733995771 | 8.427963333 | 14.79728543 | 3.69E-12 | 4.68E-08 | 17.83109944 |
| ZNF492 | 2.188668475 | 3.000090494 | 14.50153618 | 5.33E-12 | 4.68E-08 | 17.49194291 |
| ARL13B | 2.13338531 | 3.538671755 | 13.97674343 | 1.04E-11 | 6.09E-08 | 16.87212237 |
| TGM2 | -3.702105136 | 8.628519554 | -12.89201136 | 4.41E-11 | 1.55E-07 | 15.51339426 |
| AP1AR | 2.728630384 | 5.298098858 | 12.73241896 | 5.51E-11 | 1.55E-07 | 15.30415555 |
| ZNF12 | 2.033945551 | 9.921474236 | 12.68770217 | 5.86E-11 | 1.55E-07 | 15.24508085 |
| DPY19L4 | 2.792004104 | 5.358040771 | 12.64992532 | 6.18E-11 | 1.55E-07 | 15.19502063 |
| FAM76B | 2.458374378 | 8.407423605 | 12.52320216 | 7.38E-11 | 1.61E-07 | 15.02605721 |
| SENP7 | 2.907692511 | 8.728546901 | 12.44491183 | 8.25E-11 | 1.61E-07 | 14.92086713 |
| KPNA5 | 2.790121524 | 5.892510812 | 12.12625051 | 1.30E-10 | 2.28E-07 | 14.48627844 |
| PCNP | 2.309804843 | 10.31191192 | 12.06172459 | 1.43E-10 | 2.28E-07 | 14.39700121 |
| HBM | -2.813412219 | 15.95214193 | -11.85683738 | 1.93E-10 | 2.75E-07 | 14.11061729 |
| SLC38A2 | 1.927184776 | 10.60174442 | 11.82208733 | 2.03E-10 | 2.75E-07 | 14.06160251 |
| MED25 | -1.58792054 | 12.44539558 | -11.71589971 | 2.37E-10 | 2.98E-07 | 13.91102164 |
| MED13 | 1.821572827 | 10.00277552 | 11.64592217 | 2.64E-10 | 3.03E-07 | 13.81112251 |
| ZNF22 | 1.792691127 | 9.135524446 | 11.61579771 | 2.76E-10 | 3.03E-07 | 13.76795311 |
| C5orf28 | 2.996419425 | 5.734723502 | 11.49647134 | 3.30E-10 | 3.25E-07 | 13.59597721 |
| ZNF280D | 2.39961933 | 6.032772884 | 11.44942338 | 3.54E-10 | 3.25E-07 | 13.52773915 |
| SMARCAD1 | 1.726399764 | 8.80332634 | 11.43374361 | 3.63E-10 | 3.25E-07 | 13.50494278 |
| ARID4B | 1.856990359 | 7.843785905 | 11.40336658 | 3.80E-10 | 3.25E-07 | 13.46070073 |
| IQCJ | -3.025392303 | 3.604788356 | -11.37444651 | 3.97E-10 | 3.25E-07 | 13.41848508 |
| HBQ1 | -4.018090489 | 14.25951825 | -11.33078677 | 4.24E-10 | 3.25E-07 | 13.35457628 |
| BTAF1 | 2.505862514 | 7.396358464 | 11.31218223 | 4.36E-10 | 3.25E-07 | 13.32727816 |
| RAB3IL1 | -3.017368292 | 7.408015558 | -11.28454791 | 4.55E-10 | 3.25E-07 | 13.28665897 |
| YTHDC2 | 1.752604153 | 8.486955299 | 11.27390014 | 4.62E-10 | 3.25E-07 | 13.27098506 |
| RB1 | 2.363271752 | 9.069307015 | 11.18687095 | 5.28E-10 | 3.57E-07 | 13.14239433 |
| PURA | 1.549639564 | 10.45018918 | 11.15487858 | 5.55E-10 | 3.61E-07 | 13.09490762 |
| CUL5 | 2.125462769 | 8.383863744 | 10.96145949 | 7.49E-10 | 4.70E-07 | 12.80531524 |
| CYP2F1 | -5.152843347 | 7.636106231 | -10.92124298 | 7.98E-10 | 4.84E-07 | 12.74455933 |
| CEACAM18 | -4.570320547 | 5.456775407 | -10.89867058 | 8.26E-10 | 4.84E-07 | 12.71037624 |
| REEP5 | 1.889721622 | 11.49628129 | 10.85070195 | 8.91E-10 | 5.02E-07 | 12.63753614 |
| LALBA | -5.424681606 | 7.365038351 | -10.8344625 | 9.14E-10 | 5.02E-07 | 12.61281559 |
| HAPLN2 | 7.852634336 | 8.575715682 | 10.7938617 | 9.75E-10 | 5.02E-07 | 12.55087535 |
| MORC3 | 1.980093664 | 9.62800672 | 10.78230878 | 9.93E-10 | 5.02E-07 | 12.53321486 |
| PLVAP | -2.742205034 | 6.886203771 | -10.72944227 | 1.08E-09 | 5.02E-07 | 12.45219887 |
| ATAD1 | 2.682832517 | 9.852097441 | 10.71704519 | 1.10E-09 | 5.02E-07 | 12.43315293 |
| MRC2 | -3.255507044 | 11.16806078 | -10.71275026 | 1.11E-09 | 5.02E-07 | 12.42655028 |
| SMOX | -2.939513525 | 9.303807699 | -10.70901141 | 1.11E-09 | 5.02E-07 | 12.42080071 |
| F2RL3 | -1.611549227 | 8.696684468 | -10.6969537 | 1.14E-09 | 5.02E-07 | 12.40224717 |
| PREPL | 2.409072514 | 6.731503057 | 10.69373649 | 1.14E-09 | 5.02E-07 | 12.39729384 |
| FNDC3A | 1.974497754 | 8.631319682 | 10.64551286 | 1.23E-09 | 5.29E-07 | 12.32289942 |
| DNAJB14 | 2.49797453 | 9.103540933 | 10.60594084 | 1.31E-09 | 5.34E-07 | 12.26164454 |
| CFL2 | 2.604826111 | 4.342783211 | 10.60266791 | 1.32E-09 | 5.34E-07 | 12.25656987 |
| MTDH | 2.39485816 | 10.23182495 | 10.59545813 | 1.34E-09 | 5.34E-07 | 12.2453866 |
| CHADL | -1.977280779 | 7.692913938 | -10.55614306 | 1.42E-09 | 5.56E-07 | 12.18429427 |
| KIAA1033 | 2.172935401 | 10.28414133 | 10.52697166 | 1.49E-09 | 5.64E-07 | 12.13884424 |
| ZNF594 | 3.371702595 | 4.809548261 | 10.51961218 | 1.51E-09 | 5.64E-07 | 12.12736173 |
| TNFAIP8 | 1.931331876 | 10.64745741 | 10.4892562 | 1.58E-09 | 5.80E-07 | 12.07993015 |
| CPNE3 | 1.425243724 | 7.037153119 | 10.46262721 | 1.65E-09 | 5.83E-07 | 12.03823025 |
| SCAF11 | 1.786225486 | 10.21972956 | 10.45512293 | 1.67E-09 | 5.83E-07 | 12.02646334 |
| CAPS2 | 2.792921903 | 3.781474013 | 10.44924758 | 1.69E-09 | 5.83E-07 | 12.01724585 |
| ALG6 | 1.359673225 | 9.459869025 | 10.42274689 | 1.76E-09 | 5.91E-07 | 11.97561844 |
| PIGA | 1.841885062 | 6.749599359 | 10.41683488 | 1.78E-09 | 5.91E-07 | 11.96632017 |
| NUDCD1 | 2.381926676 | 3.82400575 | 10.3996512 | 1.83E-09 | 5.96E-07 | 11.93926992 |
| BDH2 | 2.153247266 | 3.260183364 | 10.35772498 | 1.96E-09 | 6.19E-07 | 11.87311925 |
| C5orf15 | 1.79772219 | 8.102782485 | 10.34877263 | 1.99E-09 | 6.19E-07 | 11.8589665 |
| PHC3 | 2.144536419 | 11.22675164 | 10.34328767 | 2.01E-09 | 6.19E-07 | 11.85029049 |
| ANGEL2 | 1.977451625 | 7.486728002 | 10.2645823 | 2.28E-09 | 6.92E-07 | 11.72538909 |
| ETNK1 | 1.875146061 | 8.591516425 | 10.23905031 | 2.38E-09 | 7.09E-07 | 11.68470727 |
| TDG | 1.387725404 | 9.670015473 | 10.205793 | 2.51E-09 | 7.27E-07 | 11.63159536 |
| NPRL3 | -2.129744877 | 11.74302117 | -10.20390712 | 2.52E-09 | 7.27E-07 | 11.6285795 |
| CREBZF | 2.62845681 | 4.983802132 | 10.17102948 | 2.66E-09 | 7.28E-07 | 11.5759315 |
| OSBP2 | -3.194467627 | 8.337653421 | -10.16662155 | 2.68E-09 | 7.28E-07 | 11.56886272 |
| TMEM38B | 2.198016376 | 5.131756242 | 10.16556874 | 2.68E-09 | 7.28E-07 | 11.56717403 |
| RALGAPA1 | 1.783938526 | 7.621612947 | 10.16407501 | 2.69E-09 | 7.28E-07 | 11.56477788 |
| ZFR | 1.597685955 | 8.715746704 | 10.14482359 | 2.78E-09 | 7.33E-07 | 11.53387096 |
| TMX1 | 2.014376409 | 10.94947271 | 10.13357674 | 2.83E-09 | 7.33E-07 | 11.51579353 |
| EEA1 | 1.996534016 | 7.967948205 | 10.12773578 | 2.86E-09 | 7.33E-07 | 11.50639893 |
| FAM133B | 1.659345543 | 8.19886071 | 10.12409679 | 2.87E-09 | 7.33E-07 | 11.50054383 |
| ENPP4 | 2.044838349 | 8.391792757 | 10.10605759 | 2.96E-09 | 7.44E-07 | 11.47149453 |
| HACE1 | 2.250548149 | 5.3308247 | 10.09370618 | 3.02E-09 | 7.49E-07 | 11.45158106 |
| ATP8A1 | 2.075507744 | 10.13331006 | 10.05984251 | 3.20E-09 | 7.71E-07 | 11.39688671 |
| PTAR1 | 1.940012326 | 9.1282376 | 10.05940315 | 3.20E-09 | 7.71E-07 | 11.39617614 |
| IGIP | 2.934461695 | 6.120299994 | 10.04275846 | 3.29E-09 | 7.73E-07 | 11.36923913 |
| GUK1 | -1.980136534 | 15.16376887 | -10.0413741 | 3.30E-09 | 7.73E-07 | 11.36699718 |
| SPIN4 | 2.628576281 | 3.494354283 | 10.01073061 | 3.47E-09 | 8.03E-07 | 11.31730877 |
| IMPA1 | 2.299952128 | 7.561484959 | 9.983418581 | 3.63E-09 | 8.19E-07 | 11.27292268 |
| TRAM1 | 1.550979961 | 11.28378598 | 9.983391867 | 3.63E-09 | 8.19E-07 | 11.27287922 |
| RNF208 | -2.020455748 | 7.228315792 | -9.973089096 | 3.69E-09 | 8.21E-07 | 11.25611121 |
| SRSF2 | 1.378722924 | 11.40516442 | 9.966696486 | 3.73E-09 | 8.21E-07 | 11.24570035 |
| BLVRB | -1.681225104 | 15.54014912 | -9.952943589 | 3.82E-09 | 8.29E-07 | 11.22328517 |
| CSNK1G3 | 2.212327697 | 7.234788468 | 9.939799093 | 3.91E-09 | 8.37E-07 | 11.20183923 |
| MMS22L | 1.219791794 | 6.052580517 | 9.924329564 | 4.01E-09 | 8.49E-07 | 11.17657184 |
| GRPEL2 | 2.251164998 | 7.482030188 | 9.88549891 | 4.28E-09 | 8.95E-07 | 11.11301346 |
| UBE2E1 | 2.451849345 | 4.997944819 | 9.870082222 | 4.39E-09 | 9.05E-07 | 11.08772612 |
| ARFGEF1 | 1.408050979 | 9.064519371 | 9.864865539 | 4.43E-09 | 9.05E-07 | 11.07916257 |
| SVIP | 2.524488457 | 3.356523777 | 9.832336993 | 4.68E-09 | 9.33E-07 | 11.02568639 |
| LRRC40 | 2.824895102 | 6.92280976 | 9.829841031 | 4.70E-09 | 9.33E-07 | 11.02157751 |
| UBQLN2 | 1.494614924 | 9.290800403 | 9.826732311 | 4.72E-09 | 9.33E-07 | 11.01645879 |
| MEX3C | 2.651484481 | 9.064055216 | 9.805003825 | 4.90E-09 | 9.57E-07 | 10.98064682 |
| ZBED5 | 1.981836353 | 12.03664851 | 9.791806166 | 5.01E-09 | 9.66E-07 | 10.95886551 |
| TRPC1 | 2.74579059 | 4.996202818 | 9.785471566 | 5.06E-09 | 9.66E-07 | 10.94840301 |
| ATP13A3 | 1.379668509 | 8.887983361 | 9.780182936 | 5.11E-09 | 9.66E-07 | 10.93966414 |
| SLC22A11 | -3.80857407 | 6.222792162 | -9.768674445 | 5.21E-09 | 9.74E-07 | 10.92063525 |
| OGT | 1.583344335 | 12.74226866 | 9.750092488 | 5.38E-09 | 9.87E-07 | 10.88987474 |
| MAT2A | 1.438582889 | 9.272965909 | 9.7486486 | 5.39E-09 | 9.87E-07 | 10.88748268 |
| HECTD1 | 1.413982514 | 10.92687425 | 9.741977452 | 5.45E-09 | 9.88E-07 | 10.87642722 |
| PANK3 | 1.780643951 | 8.192102495 | 9.716626018 | 5.69E-09 | 1.01E-06 | 10.83436246 |
| PUM1 | 1.045895403 | 10.1683519 | 9.715121194 | 5.70E-09 | 1.01E-06 | 10.83186296 |
| SOCS5 | 1.895786534 | 6.878852123 | 9.704270854 | 5.81E-09 | 1.02E-06 | 10.81383196 |
| ANKRD49 | 2.543659252 | 9.636554375 | 9.66881841 | 6.17E-09 | 1.07E-06 | 10.75481147 |
| BAG1 | -1.877971523 | 14.10093225 | -9.647381929 | 6.40E-09 | 1.10E-06 | 10.71904563 |
| MIB1 | 2.339671026 | 7.140596339 | 9.643469691 | 6.44E-09 | 1.10E-06 | 10.71251181 |
| TIA1 | 2.01998398 | 8.663370702 | 9.638059276 | 6.50E-09 | 1.10E-06 | 10.70347261 |
| TMEM209 | 1.522213459 | 8.298170614 | 9.634906301 | 6.54E-09 | 1.10E-06 | 10.69820318 |
| SP4 | 1.582925862 | 8.547496907 | 9.583471876 | 7.14E-09 | 1.18E-06 | 10.61206078 |
| KRT31 | -4.32006424 | 8.515417978 | -9.581543902 | 7.16E-09 | 1.18E-06 | 10.60882512 |
| ZNF100 | 1.837197309 | 6.279743456 | 9.560104913 | 7.43E-09 | 1.21E-06 | 10.5728121 |
| RBBP9 | 1.727016617 | 5.821325339 | 9.541513293 | 7.67E-09 | 1.24E-06 | 10.54153355 |
| EDEM3 | 2.284326275 | 8.79474379 | 9.528229961 | 7.85E-09 | 1.25E-06 | 10.51915803 |
| CLK4 | 2.677079144 | 8.167399438 | 9.52405376 | 7.91E-09 | 1.25E-06 | 10.51211854 |
| OR10A4 | -5.924658856 | 6.84126883 | -9.516746657 | 8.01E-09 | 1.26E-06 | 10.49979604 |
| CEPT1 | 2.351663117 | 6.267220166 | 9.504690917 | 8.18E-09 | 1.27E-06 | 10.4794503 |
| PLS1 | 1.801752603 | 3.0222822 | 9.491539519 | 8.36E-09 | 1.29E-06 | 10.45723379 |
| ZXDB | 1.285363483 | 8.912191147 | 9.485670795 | 8.45E-09 | 1.29E-06 | 10.44731251 |
| ADAM11 | -2.339803601 | 6.479844321 | -9.480580268 | 8.52E-09 | 1.29E-06 | 10.43870314 |
| CPEB2 | 2.954640584 | 4.014495676 | 9.478218225 | 8.56E-09 | 1.29E-06 | 10.43470717 |
| SREK1IP1 | 1.884462316 | 5.184137778 | 9.469995112 | 8.68E-09 | 1.29E-06 | 10.42079007 |
| SLC25A32 | 1.464627943 | 8.150665921 | 9.444150681 | 9.08E-09 | 1.31E-06 | 10.37699222 |
| USP9X | 1.302183203 | 8.114260214 | 9.441904482 | 9.11E-09 | 1.31E-06 | 10.3731815 |
| KLHL28 | 1.659855918 | 9.748497558 | 9.437828427 | 9.18E-09 | 1.31E-06 | 10.3662647 |
| KIR2DL4 | -2.783225214 | 8.550869796 | -9.43764968 | 9.18E-09 | 1.31E-06 | 10.36596133 |
| ARHGAP5 | 2.128083145 | 6.370925278 | 9.436545717 | 9.20E-09 | 1.31E-06 | 10.36408757 |
| CLASP2 | 0.941324654 | 8.674980514 | 9.434232164 | 9.24E-09 | 1.31E-06 | 10.36016026 |
| MBLAC2 | 2.302510734 | 5.801604231 | 9.416833247 | 9.52E-09 | 1.33E-06 | 10.33060262 |
| RNF219 | 2.185794786 | 8.454916663 | 9.41672462 | 9.52E-09 | 1.33E-06 | 10.33041796 |
| PIP5KL1 | -1.997550582 | 6.13839025 | -9.404603513 | 9.72E-09 | 1.35E-06 | 10.30980263 |
| CD164 | 1.591106466 | 10.69097268 | 9.392458501 | 9.93E-09 | 1.36E-06 | 10.28912721 |
| SYCP2 | 2.613628681 | 3.76674256 | 9.379947544 | 1.01E-08 | 1.38E-06 | 10.26780847 |
| LYPLAL1 | 1.585636685 | 7.89185619 | 9.376956791 | 1.02E-08 | 1.38E-06 | 10.26270914 |
| OR2A25 | -4.913009644 | 6.50546636 | -9.363279008 | 1.04E-08 | 1.38E-06 | 10.23937305 |
| RDH14 | 1.929189008 | 8.359119014 | 9.358463506 | 1.05E-08 | 1.38E-06 | 10.23115129 |
| GCOM1 | 1.916724017 | 7.354990798 | 9.354922407 | 1.06E-08 | 1.38E-06 | 10.22510343 |
| PPP3CB | 1.475694092 | 8.810735896 | 9.353808953 | 1.06E-08 | 1.38E-06 | 10.22320141 |
| PAPOLG | 1.950367494 | 7.686160612 | 9.344136868 | 1.08E-08 | 1.38E-06 | 10.20667251 |
| LRRCC1 | 2.701460787 | 3.780541664 | 9.343444404 | 1.08E-08 | 1.38E-06 | 10.20548867 |
| SLTM | 1.543344601 | 9.195533107 | 9.34273514 | 1.08E-08 | 1.38E-06 | 10.20427603 |
| RBM25 | 2.082902049 | 10.56283752 | 9.340312651 | 1.09E-08 | 1.38E-06 | 10.20013378 |
| HNRNPH3 | 1.822766651 | 10.07267943 | 9.337081864 | 1.09E-08 | 1.38E-06 | 10.19460821 |
| ZNF658 | 1.484273997 | 7.218444971 | 9.335750921 | 1.10E-08 | 1.38E-06 | 10.19233151 |
| RFX7 | 1.373907491 | 8.744693015 | 9.326001106 | 1.11E-08 | 1.39E-06 | 10.17564641 |
| ZBTB11 | 1.427591893 | 9.106949591 | 9.322196776 | 1.12E-08 | 1.39E-06 | 10.16913254 |
| BMI1 | 2.34538118 | 6.542998739 | 9.319371541 | 1.13E-08 | 1.39E-06 | 10.16429387 |
| LEMD3 | 2.648684971 | 8.586018087 | 9.308850685 | 1.15E-08 | 1.40E-06 | 10.14626588 |
| SOCS4 | 1.678907651 | 8.80267929 | 9.29544663 | 1.18E-08 | 1.42E-06 | 10.1232761 |
| ATRX | 1.581938768 | 8.132923329 | 9.29024434 | 1.19E-08 | 1.42E-06 | 10.11434704 |
| UFL1 | 2.573458714 | 7.600244409 | 9.290134236 | 1.19E-08 | 1.42E-06 | 10.11415802 |
| RYK | 1.6929094 | 9.913608815 | 9.285772933 | 1.20E-08 | 1.42E-06 | 10.10666956 |
| MAP4K5 | 2.170318701 | 8.052904366 | 9.280141194 | 1.21E-08 | 1.43E-06 | 10.096996 |
| RB1CC1 | 1.431797075 | 9.31816541 | 9.26646972 | 1.24E-08 | 1.44E-06 | 10.07349515 |
| SZT2 | -1.562402984 | 6.733876676 | -9.263795719 | 1.24E-08 | 1.44E-06 | 10.06889571 |
| CPOX | 1.990571533 | 8.4514443 | 9.26132916 | 1.25E-08 | 1.44E-06 | 10.06465225 |
| GFI1B | -2.289076763 | 8.785625803 | -9.259804101 | 1.25E-08 | 1.44E-06 | 10.06202813 |
| NIPSNAP3B | 1.641580969 | 3.103026992 | 9.25641396 | 1.26E-08 | 1.44E-06 | 10.05619372 |
| BIRC3 | 2.05781133 | 11.05487234 | 9.251476333 | 1.27E-08 | 1.44E-06 | 10.04769336 |
| ARHGAP12 | 2.583942371 | 8.864101227 | 9.2484214 | 1.28E-08 | 1.44E-06 | 10.04243252 |
| CTDSPL2 | 1.76960693 | 7.218590868 | 9.238001276 | 1.30E-08 | 1.45E-06 | 10.02447887 |
| LBR | 1.734327646 | 12.73007172 | 9.235844234 | 1.31E-08 | 1.45E-06 | 10.02076053 |
| CEP104 | -3.097325998 | 8.929375269 | -9.22212924 | 1.34E-08 | 1.48E-06 | 9.997103863 |
| TMX3 | 2.323944331 | 9.336215724 | 9.204968798 | 1.38E-08 | 1.51E-06 | 9.96746886 |
| CTTN | -2.656456908 | 8.296382184 | -9.204435805 | 1.38E-08 | 1.51E-06 | 9.966547785 |
| SACS | 2.03807317 | 7.595242426 | 9.166408399 | 1.48E-08 | 1.60E-06 | 9.90073381 |
| FAM180A | -3.461204074 | 7.41109323 | -9.162670713 | 1.49E-08 | 1.60E-06 | 9.894254553 |
| BAIAP2L2 | -1.118614377 | 7.313644087 | -9.160011843 | 1.49E-08 | 1.60E-06 | 9.889644275 |
| ANKRD10 | 1.286381631 | 8.412570608 | 9.149629016 | 1.52E-08 | 1.61E-06 | 9.871632171 |
| KPNA3 | 1.541053433 | 9.003244319 | 9.149447098 | 1.52E-08 | 1.61E-06 | 9.871316451 |
| ZNF681 | 2.065506145 | 3.836910711 | 9.144548334 | 1.53E-08 | 1.61E-06 | 9.862812946 |
| SLC26A2 | 1.950069982 | 8.059715456 | 9.092877107 | 1.68E-08 | 1.76E-06 | 9.772923278 |
| RGS12 | -2.146601942 | 7.34443099 | -9.083442376 | 1.71E-08 | 1.78E-06 | 9.756471406 |
| CLPB | -1.335179127 | 8.769734782 | -9.079483871 | 1.72E-08 | 1.78E-06 | 9.749565167 |
| FAIM2 | -3.008686242 | 6.356559321 | -9.073769899 | 1.74E-08 | 1.79E-06 | 9.739592515 |
| UBE4A | 1.369339608 | 10.90689888 | 9.071251214 | 1.75E-08 | 1.79E-06 | 9.735195231 |
| LAMB2 | -1.663109122 | 7.418364558 | -9.063200179 | 1.77E-08 | 1.80E-06 | 9.721133474 |
| C2orf88 | -2.075119236 | 8.176848024 | -9.052498205 | 1.81E-08 | 1.82E-06 | 9.702428111 |
| RAP1B | 1.953127066 | 11.85694675 | 9.046922706 | 1.82E-08 | 1.82E-06 | 9.692676892 |
| MAMDC4 | -0.965759289 | 7.884325724 | -9.046310548 | 1.83E-08 | 1.82E-06 | 9.69160601 |
| KLC3 | -3.807859597 | 9.335140362 | -9.045152726 | 1.83E-08 | 1.82E-06 | 9.689580426 |
| KLF1 | -3.12431621 | 12.39358271 | -9.037469821 | 1.85E-08 | 1.82E-06 | 9.676134765 |
| ZNF91 | 3.126489344 | 5.041384842 | 9.037137979 | 1.86E-08 | 1.82E-06 | 9.675553837 |
| SPIN1 | 1.775112014 | 6.794909005 | 9.028501176 | 1.88E-08 | 1.84E-06 | 9.660428875 |
| ALG10 | 2.404350395 | 4.698759995 | 9.0265996 | 1.89E-08 | 1.84E-06 | 9.657097439 |
| AK1 | -1.98408593 | 10.28529374 | -9.02289264 | 1.90E-08 | 1.84E-06 | 9.650601682 |
| NKX1-2 | -3.995403506 | 11.4025994 | -9.018118136 | 1.92E-08 | 1.84E-06 | 9.642232515 |
| DMXL1 | 2.430942312 | 6.37477924 | 9.010988936 | 1.94E-08 | 1.86E-06 | 9.629730094 |
| ZNF273 | 2.632862372 | 6.898662849 | 8.96903873 | 2.10E-08 | 1.98E-06 | 9.55602294 |
| TIGD1 | 2.133014473 | 6.047414499 | 8.965465571 | 2.11E-08 | 1.98E-06 | 9.54973382 |
| ZNF699 | 2.697055372 | 5.502536173 | 8.964788748 | 2.11E-08 | 1.98E-06 | 9.548542348 |
| MAP9 | 2.152978742 | 5.565765435 | 8.963364489 | 2.12E-08 | 1.98E-06 | 9.546034894 |
| FBXL3 | 2.011355924 | 9.75033815 | 8.956657544 | 2.14E-08 | 1.98E-06 | 9.534223403 |
| C18orf25 | 0.955450584 | 8.623185687 | 8.956539977 | 2.14E-08 | 1.98E-06 | 9.534016303 |
| PHOSPHO1 | -2.437590409 | 14.61331455 | -8.948986184 | 2.17E-08 | 1.99E-06 | 9.520706009 |
| ZMPSTE24 | 1.240276054 | 11.27763461 | 8.948569395 | 2.17E-08 | 1.99E-06 | 9.519971374 |
| SPARC | -1.850726808 | 7.617135975 | -8.944820618 | 2.19E-08 | 1.99E-06 | 9.513362686 |
| ZBED6 | 2.164879638 | 7.150937656 | 8.937622947 | 2.22E-08 | 2.01E-06 | 9.500668618 |
| TMED5 | 1.646877659 | 9.650862934 | 8.934335243 | 2.23E-08 | 2.01E-06 | 9.494867963 |
| ZNF615 | 1.51674057 | 7.082174502 | 8.922257845 | 2.28E-08 | 2.04E-06 | 9.473546614 |
| LCORL | 2.120408025 | 5.435311993 | 8.91593578 | 2.30E-08 | 2.06E-06 | 9.462377776 |
| CCDC14 | 2.07161385 | 8.067684537 | 8.903218133 | 2.36E-08 | 2.09E-06 | 9.439893742 |
| PLEKHF2 | 2.311738881 | 9.6549228 | 8.902749867 | 2.36E-08 | 2.09E-06 | 9.439065456 |
| P2RX2 | -3.490235521 | 8.411677332 | -8.899873593 | 2.37E-08 | 2.09E-06 | 9.433977139 |
| PSIP1 | 2.013105727 | 9.521390054 | 8.894821945 | 2.39E-08 | 2.09E-06 | 9.425037717 |
| LAMA3 | -4.673651199 | 8.208707802 | -8.888438766 | 2.42E-08 | 2.11E-06 | 9.413737037 |
| PTGER4 | 1.976740336 | 10.44477712 | 8.870512951 | 2.50E-08 | 2.17E-06 | 9.381971751 |
| UBL7 | -1.286048061 | 9.470309368 | -8.853834208 | 2.58E-08 | 2.22E-06 | 9.352376952 |
| ZC3H11A | 1.270591948 | 10.83979116 | 8.850086534 | 2.59E-08 | 2.22E-06 | 9.345721844 |
| ANKRD12 | 2.122375832 | 8.210239702 | 8.850014812 | 2.60E-08 | 2.22E-06 | 9.345594461 |
| KDM5A | 2.736644182 | 3.746754398 | 8.84546516 | 2.62E-08 | 2.22E-06 | 9.33751258 |
| VWF | -2.460203264 | 7.818063199 | -8.839378144 | 2.65E-08 | 2.24E-06 | 9.326695345 |
| INTS2 | 2.193994272 | 3.255850246 | 8.835581591 | 2.66E-08 | 2.24E-06 | 9.319945925 |
| ZFP37 | 1.967216065 | 3.200469615 | 8.828305792 | 2.70E-08 | 2.26E-06 | 9.30700568 |
| VEPH1 | -2.119149055 | 5.345180158 | -8.817592872 | 2.75E-08 | 2.29E-06 | 9.287939224 |
| NUFIP2 | 1.352655176 | 10.91664976 | 8.810241545 | 2.79E-08 | 2.31E-06 | 9.27484652 |
| ZNF287 | 2.484506022 | 3.729114809 | 8.804698933 | 2.82E-08 | 2.32E-06 | 9.264970244 |
| CEP170 | 1.721592005 | 7.070893865 | 8.800383343 | 2.84E-08 | 2.32E-06 | 9.257277461 |
| ZNF765 | 1.427377814 | 7.727539088 | 8.799986254 | 2.84E-08 | 2.32E-06 | 9.256569499 |
| TAS2R19 | 2.115568136 | 4.991465349 | 8.79750018 | 2.85E-08 | 2.32E-06 | 9.252136638 |
| BPIFB2 | -4.471560701 | 6.728279055 | -8.787429869 | 2.91E-08 | 2.36E-06 | 9.234171839 |
| FKBP8 | -2.20054541 | 12.03554197 | -8.778068772 | 2.96E-08 | 2.38E-06 | 9.217459763 |
| C3orf38 | 1.604154609 | 7.900952735 | 8.767461989 | 3.01E-08 | 2.41E-06 | 9.198509282 |
| PDZK1IP1 | -2.923951347 | 11.97149114 | -8.765850806 | 3.02E-08 | 2.41E-06 | 9.195629331 |
| CMTM5 | -2.77590828 | 9.746172208 | -8.764598177 | 3.03E-08 | 2.41E-06 | 9.19339004 |
| GJC2 | -2.097082009 | 7.131484462 | -8.760174562 | 3.05E-08 | 2.42E-06 | 9.185480339 |
| TRPM7 | 2.581338843 | 5.819318062 | 8.758102706 | 3.06E-08 | 2.42E-06 | 9.181774807 |
| SLC5A3 | 2.004031493 | 8.284732086 | 8.756056365 | 3.08E-08 | 2.42E-06 | 9.178114331 |
| NUP160 | 1.152866527 | 8.764985128 | 8.752179655 | 3.10E-08 | 2.42E-06 | 9.171178131 |
| MIER3 | 1.966950467 | 6.540319114 | 8.734164069 | 3.20E-08 | 2.47E-06 | 9.138917598 |
| FAM84B | 2.579859591 | 3.947960652 | 8.733833487 | 3.20E-08 | 2.47E-06 | 9.138325207 |
| SMURF2 | 3.139920348 | 4.10864787 | 8.733113827 | 3.21E-08 | 2.47E-06 | 9.137035552 |
| DCAF10 | -1.72084196 | 8.339728499 | -8.729084762 | 3.23E-08 | 2.48E-06 | 9.129814015 |
| ADRB2 | 1.689260872 | 11.07371451 | 8.708911454 | 3.35E-08 | 2.56E-06 | 9.093622593 |
| PARD6B | 1.815617375 | 3.541852035 | 8.702865365 | 3.39E-08 | 2.58E-06 | 9.08276485 |
| PLEK2 | -2.726365181 | 10.12013038 | -8.694526846 | 3.44E-08 | 2.61E-06 | 9.067782034 |
| SIAE | 1.634394811 | 4.766764917 | 8.681137253 | 3.53E-08 | 2.66E-06 | 9.04370331 |
| TCF23 | -1.763920845 | 5.704417164 | -8.667937019 | 3.61E-08 | 2.70E-06 | 9.01994092 |
| ZNF736 | 3.712469544 | 4.408972119 | 8.667638186 | 3.61E-08 | 2.70E-06 | 9.019402698 |
| PCF11 | 2.025583655 | 9.104732859 | 8.663722001 | 3.64E-08 | 2.71E-06 | 9.012348197 |
| KLHL9 | 1.098288572 | 10.02759405 | 8.662322944 | 3.65E-08 | 2.71E-06 | 9.009827464 |
| ANKRD33 | -4.242441776 | 10.57041712 | -8.652636255 | 3.71E-08 | 2.74E-06 | 8.992367185 |
| HAGH | -1.867171005 | 14.2512795 | -8.640868479 | 3.80E-08 | 2.79E-06 | 8.971138315 |
| SRSF10 | 1.638187989 | 9.573965601 | 8.619058489 | 3.95E-08 | 2.89E-06 | 8.931742872 |
| GEN1 | 2.556595708 | 4.524908944 | 8.609683435 | 4.02E-08 | 2.93E-06 | 8.914788479 |
| LARP4 | 1.268752987 | 8.14478759 | 8.605559618 | 4.05E-08 | 2.94E-06 | 8.907326877 |
| NFE2 | -1.425187125 | 15.39867047 | -8.603786674 | 4.06E-08 | 2.94E-06 | 8.904118205 |
| RCN2 | 1.491387039 | 10.41575394 | 8.591097698 | 4.16E-08 | 2.98E-06 | 8.881140999 |
| GNPDA2 | 1.88854426 | 7.022033314 | 8.588008847 | 4.18E-08 | 2.98E-06 | 8.875544332 |
| PLA2G6 | -1.12205733 | 8.363602468 | -8.587952984 | 4.18E-08 | 2.98E-06 | 8.875443102 |
| KLHDC8A | -3.231152455 | 6.305526521 | -8.584779945 | 4.21E-08 | 2.98E-06 | 8.869692495 |
| HOOK3 | 1.210989591 | 9.160603577 | 8.583210503 | 4.22E-08 | 2.98E-06 | 8.866847626 |
| UBE2Q2 | 1.539679847 | 10.02261886 | 8.582563782 | 4.22E-08 | 2.98E-06 | 8.86567524 |
| ZNF260 | 1.543036267 | 7.237699914 | 8.566874295 | 4.35E-08 | 3.06E-06 | 8.837215332 |
| DNAJC28 | 2.050686509 | 3.309323074 | 8.5622199 | 4.39E-08 | 3.07E-06 | 8.828765942 |
| SEC24A | 1.590129178 | 6.342101207 | 8.56061074 | 4.40E-08 | 3.07E-06 | 8.825844042 |
| GAS2L1 | -1.999317525 | 8.536723968 | -8.555566581 | 4.44E-08 | 3.08E-06 | 8.816682573 |
| SNX4 | 1.710435297 | 7.859238034 | 8.5539092 | 4.45E-08 | 3.08E-06 | 8.813671579 |
| VWCE | -3.241844304 | 11.05747364 | -8.551240127 | 4.48E-08 | 3.09E-06 | 8.808821826 |
| EPB42 | -2.993345225 | 13.03345679 | -8.531399017 | 4.64E-08 | 3.18E-06 | 8.772739189 |
| NEMF | 2.192477189 | 9.602376072 | 8.530295524 | 4.65E-08 | 3.18E-06 | 8.770730797 |
| NEDD1 | 1.480583625 | 10.46661704 | 8.526806917 | 4.68E-08 | 3.19E-06 | 8.764380303 |
| PHTF2 | 2.735862563 | 7.454059715 | 8.517023947 | 4.77E-08 | 3.24E-06 | 8.746562839 |
| ELMOD3 | -2.318788423 | 10.08310764 | -8.503744106 | 4.89E-08 | 3.30E-06 | 8.722355337 |
| DNAJC10 | 2.005609048 | 9.625048758 | 8.493902126 | 4.97E-08 | 3.35E-06 | 8.704398812 |
| TMEM182 | 2.031910316 | 4.435582138 | 8.49271312 | 4.99E-08 | 3.35E-06 | 8.702228578 |
| KLHDC1 | 2.114345367 | 5.378984497 | 8.486482742 | 5.04E-08 | 3.37E-06 | 8.690853367 |
| RAB11FIP2 | 1.858132483 | 7.402309538 | 8.48350047 | 5.07E-08 | 3.38E-06 | 8.685406525 |
| RNF6 | 2.298702723 | 7.576739127 | 8.476236102 | 5.14E-08 | 3.40E-06 | 8.672133655 |
| C6orf132 | -1.411375363 | 4.483373427 | -8.475976494 | 5.14E-08 | 3.40E-06 | 8.671659183 |
| MKL2 | 1.257498814 | 7.097847955 | 8.46931806 | 5.21E-08 | 3.41E-06 | 8.659486736 |
| NUP153 | 1.472528289 | 9.809032255 | 8.46760218 | 5.22E-08 | 3.41E-06 | 8.656348893 |
| DPM2 | -2.367914109 | 10.51358695 | -8.465202644 | 5.25E-08 | 3.41E-06 | 8.651960155 |
| BNIP2 | 1.568909214 | 10.76912658 | 8.463936105 | 5.26E-08 | 3.41E-06 | 8.649643339 |
| ZNF737 | 3.091320214 | 4.445925006 | 8.46282822 | 5.27E-08 | 3.41E-06 | 8.647616558 |
| SLC25A24 | 2.591134237 | 8.272546745 | 8.461573135 | 5.28E-08 | 3.41E-06 | 8.64532028 |
| SECISBP2L | 2.792573067 | 5.555043611 | 8.459126987 | 5.31E-08 | 3.42E-06 | 8.640844227 |
| KIAA1715 | 1.798689712 | 4.906940592 | 8.453472842 | 5.36E-08 | 3.44E-06 | 8.630494874 |
| IDI1 | 1.839401477 | 9.1020303 | 8.450012454 | 5.40E-08 | 3.45E-06 | 8.624158777 |
| PSMA8 | 2.335873745 | 3.250058908 | 8.443709377 | 5.46E-08 | 3.48E-06 | 8.612613326 |
| PDP1 | 2.430481891 | 9.232167498 | 8.439387426 | 5.50E-08 | 3.49E-06 | 8.604693538 |
| CCP110 | 1.967823647 | 7.009772933 | 8.43557773 | 5.54E-08 | 3.51E-06 | 8.597710276 |
| PRAP1 | -1.642582761 | 11.31268041 | -8.427481331 | 5.63E-08 | 3.54E-06 | 8.582862664 |
| YWHAG | 1.117027793 | 11.14110323 | 8.427171813 | 5.63E-08 | 3.54E-06 | 8.58229487 |
| MBTPS2 | 1.975589886 | 5.371514557 | 8.418795423 | 5.72E-08 | 3.58E-06 | 8.566923812 |
| ZNF107 | 2.763569944 | 7.151602517 | 8.400890478 | 5.91E-08 | 3.67E-06 | 8.534034587 |
| PPIP5K2 | 2.17854654 | 8.563092552 | 8.399482348 | 5.93E-08 | 3.67E-06 | 8.531446124 |
| ZMAT1 | 2.039779468 | 7.913285911 | 8.399232602 | 5.93E-08 | 3.67E-06 | 8.530987006 |
| LRRC1 | 1.414783251 | 4.298716716 | 8.395140366 | 5.98E-08 | 3.67E-06 | 8.52346283 |
| TRIM33 | 1.996839925 | 8.979024909 | 8.393805314 | 5.99E-08 | 3.67E-06 | 8.521007636 |
| RTN4RL1 | -3.420276792 | 7.128499385 | -8.393799227 | 5.99E-08 | 3.67E-06 | 8.520996442 |
| CHSY1 | 1.089845794 | 10.80500027 | 8.391984247 | 6.01E-08 | 3.67E-06 | 8.517658247 |
| ZNF644 | 2.540475193 | 8.525097788 | 8.384316961 | 6.10E-08 | 3.71E-06 | 8.503551142 |
| PPP1R15B | 1.223293149 | 10.48114583 | 8.381486433 | 6.13E-08 | 3.71E-06 | 8.498341155 |
| ZNF180 | 2.017804934 | 6.35689633 | 8.380246856 | 6.14E-08 | 3.71E-06 | 8.496059185 |
| SRSF1 | 2.335583518 | 6.198182712 | 8.370210835 | 6.26E-08 | 3.76E-06 | 8.477575704 |
| VPS13C | 1.898631153 | 8.862665372 | 8.365362248 | 6.32E-08 | 3.76E-06 | 8.468640951 |
| KRTAP5-4 | -2.479937736 | 9.347224902 | -8.36451344 | 6.33E-08 | 3.76E-06 | 8.467076467 |
| HDHD2 | 1.073420696 | 10.41191127 | 8.363164029 | 6.34E-08 | 3.76E-06 | 8.46458909 |
| OXR1 | 1.559782131 | 6.503601053 | 8.363076423 | 6.34E-08 | 3.76E-06 | 8.464427596 |
| TH | -1.654406091 | 7.316678996 | -8.361890928 | 6.36E-08 | 3.76E-06 | 8.462242141 |
| PSMF1 | -1.643113237 | 10.37456784 | -8.352951344 | 6.47E-08 | 3.82E-06 | 8.445755726 |
| ZNF726 | 2.279163139 | 5.047146009 | 8.349919688 | 6.50E-08 | 3.82E-06 | 8.440162197 |
| WASL | 1.143997576 | 8.941349933 | 8.343356925 | 6.58E-08 | 3.85E-06 | 8.428049225 |
| CHML | 2.540564782 | 6.24902224 | 8.3420706 | 6.60E-08 | 3.85E-06 | 8.425674334 |
| B3GNT2 | 2.362957411 | 5.735540515 | 8.322941531 | 6.84E-08 | 3.98E-06 | 8.39032974 |
| GDPD1 | 2.14293296 | 3.248522314 | 8.31479304 | 6.94E-08 | 4.01E-06 | 8.375258291 |
| VPS36 | 1.604960825 | 9.929845827 | 8.313958578 | 6.95E-08 | 4.01E-06 | 8.373714346 |
| SLC25A36 | 1.516610431 | 9.604758949 | 8.313933786 | 6.96E-08 | 4.01E-06 | 8.373668472 |
| ZNF518A | 2.171719892 | 7.560994934 | 8.304750107 | 7.08E-08 | 4.07E-06 | 8.356670086 |
| SNX13 | 1.350371069 | 8.870283547 | 8.297113523 | 7.18E-08 | 4.11E-06 | 8.342526269 |
| RAD54B | 1.70721588 | 4.641392218 | 8.284079234 | 7.36E-08 | 4.20E-06 | 8.318366409 |
| PDGFA | -1.424152915 | 6.079228617 | -8.270702705 | 7.54E-08 | 4.28E-06 | 8.293547413 |
| C2orf73 | -2.480122272 | 6.553114324 | -8.268951423 | 7.57E-08 | 4.28E-06 | 8.290296201 |
| RPS6KB1 | 1.738102302 | 6.763847255 | 8.268847623 | 7.57E-08 | 4.28E-06 | 8.290103485 |
| CDK17 | 2.565583219 | 5.303768086 | 8.255422403 | 7.76E-08 | 4.38E-06 | 8.26516541 |
| TAF4B | 1.864191583 | 4.473891584 | 8.252490987 | 7.81E-08 | 4.39E-06 | 8.259716781 |
| TESC | -1.800569908 | 15.75627188 | -8.248616099 | 7.86E-08 | 4.40E-06 | 8.252512667 |
| CNTD1 | 1.916765153 | 3.954186266 | 8.241422609 | 7.97E-08 | 4.44E-06 | 8.239133084 |
| JRKL | 1.940605997 | 4.786172163 | 8.240734732 | 7.98E-08 | 4.44E-06 | 8.237853281 |
| ESAM | -2.099548235 | 6.960322249 | -8.235024029 | 8.07E-08 | 4.47E-06 | 8.227225891 |
| PTCRA | -2.514198336 | 11.39536667 | -8.228275527 | 8.17E-08 | 4.50E-06 | 8.214661296 |
| TNP2 | -1.159487824 | 6.613623189 | -8.227238262 | 8.19E-08 | 4.50E-06 | 8.212729512 |
| RAB12 | 2.251374886 | 7.116919505 | 8.227121334 | 8.19E-08 | 4.50E-06 | 8.212511738 |
| ZNF501 | 2.240853559 | 4.121730212 | 8.222648576 | 8.26E-08 | 4.52E-06 | 8.204179949 |
| GLCCI1 | 2.5068404 | 8.125766089 | 8.209066556 | 8.47E-08 | 4.63E-06 | 8.178862326 |
| TOLLIP | -1.440259774 | 12.52459131 | -8.203541721 | 8.56E-08 | 4.65E-06 | 8.168556315 |
| PPP1R12A | 2.118020328 | 9.02215252 | 8.203474513 | 8.56E-08 | 4.65E-06 | 8.168430919 |
| KIF3A | 1.824934363 | 6.579506056 | 8.197802089 | 8.65E-08 | 4.68E-06 | 8.157845076 |
| ZNF121 | 2.934777878 | 4.24346518 | 8.192426274 | 8.74E-08 | 4.72E-06 | 8.147808586 |
| UCHL5 | 1.653149427 | 9.497744075 | 8.184703348 | 8.87E-08 | 4.75E-06 | 8.133382993 |
| CLDN12 | 1.49688657 | 3.125507623 | 8.184476561 | 8.87E-08 | 4.75E-06 | 8.132959252 |
| CPEB4 | 2.271365448 | 7.748964448 | 8.183894483 | 8.88E-08 | 4.75E-06 | 8.131871634 |
| ZNF578 | -3.412098137 | 8.271221711 | -8.178424476 | 8.98E-08 | 4.76E-06 | 8.121648553 |
| DNAJC24 | 2.089611476 | 6.61306206 | 8.177732595 | 8.99E-08 | 4.76E-06 | 8.120355174 |
| EGFL7 | -1.392061313 | 7.201258092 | -8.176977788 | 9.00E-08 | 4.76E-06 | 8.118944085 |
| KLHL24 | 1.369303992 | 8.743445878 | 8.17590708 | 9.02E-08 | 4.76E-06 | 8.116942291 |
| CAV2 | -2.263800261 | 6.363555594 | -8.174497297 | 9.04E-08 | 4.76E-06 | 8.114306318 |
| TRUB1 | 2.974326137 | 5.922839473 | 8.170656681 | 9.11E-08 | 4.78E-06 | 8.107123821 |
| UBP1 | 1.121170907 | 9.808246736 | 8.166436447 | 9.18E-08 | 4.79E-06 | 8.099228991 |
| ZNF486 | 3.174499723 | 6.419160221 | 8.166320925 | 9.18E-08 | 4.79E-06 | 8.099012848 |
| TAL1 | -1.913024669 | 6.705424216 | -8.158085712 | 9.33E-08 | 4.85E-06 | 8.083599804 |
| TRIM24 | 1.952961501 | 8.86541321 | 8.150823492 | 9.46E-08 | 4.88E-06 | 8.069999894 |
| CAND1 | 1.529487171 | 6.890546237 | 8.150446829 | 9.46E-08 | 4.88E-06 | 8.069294317 |
| SMNDC1 | 1.181416173 | 8.904374635 | 8.150186351 | 9.47E-08 | 4.88E-06 | 8.068806371 |
| MRPS35 | 0.909702277 | 10.63600845 | 8.146125577 | 9.54E-08 | 4.91E-06 | 8.061198185 |
| PI4K2B | 2.176266936 | 7.723780312 | 8.143045865 | 9.60E-08 | 4.92E-06 | 8.055426549 |
| VHLL | 2.056922323 | 3.544555537 | 8.14146879 | 9.63E-08 | 4.92E-06 | 8.052470461 |
| RUNDC3A | -2.988932722 | 8.153689894 | -8.137450848 | 9.70E-08 | 4.94E-06 | 8.044937603 |
| HMX2 | -3.627072388 | 7.999003649 | -8.13644863 | 9.72E-08 | 4.94E-06 | 8.043058285 |
| AP1M2 | -2.703111815 | 5.303939194 | -8.13331864 | 9.78E-08 | 4.95E-06 | 8.037188145 |
| YIPF2 | -1.543785537 | 10.2037025 | -8.130454548 | 9.83E-08 | 4.95E-06 | 8.031815475 |
| EFR3A | 1.533340237 | 10.64862975 | 8.13022901 | 9.83E-08 | 4.95E-06 | 8.031392344 |
| SPATA22 | -2.787772906 | 4.726057093 | -8.128935785 | 9.86E-08 | 4.95E-06 | 8.028966 |
| DGKE | 3.132298287 | 3.787723093 | 8.12328898 | 9.96E-08 | 4.99E-06 | 8.01836872 |
| SYPL1 | 1.290315453 | 10.13271133 | 8.116016743 | 1.01E-07 | 5.04E-06 | 8.004714392 |
| TRIM59 | 1.627684788 | 7.431562767 | 8.115573514 | 1.01E-07 | 5.04E-06 | 8.003881947 |
| KCNQ3 | -4.588700065 | 9.158514114 | -8.113879204 | 1.01E-07 | 5.04E-06 | 8.000699539 |
| UFM1 | 2.312038825 | 7.484735613 | 8.108015551 | 1.03E-07 | 5.08E-06 | 7.98968277 |
| PTBP2 | 2.304618199 | 7.628428975 | 8.102955035 | 1.04E-07 | 5.12E-06 | 7.980171053 |
| RMI1 | 0.945192611 | 8.131353852 | 8.096896328 | 1.05E-07 | 5.16E-06 | 7.968778394 |
| TMEM41B | 1.327226541 | 7.691332362 | 8.088872071 | 1.06E-07 | 5.22E-06 | 7.953681788 |
| SLC6A5 | -2.004919497 | 6.842336921 | -8.086767095 | 1.07E-07 | 5.22E-06 | 7.949720045 |
| ZNF655 | 1.0192033 | 9.777746708 | 8.086276899 | 1.07E-07 | 5.22E-06 | 7.948797363 |
| FAM135A | 1.626128922 | 4.875123899 | 8.082874663 | 1.08E-07 | 5.24E-06 | 7.942392506 |
| EXOC6 | 1.340411008 | 9.625323104 | 8.078974146 | 1.08E-07 | 5.27E-06 | 7.935047606 |
| NIPA2 | 1.074356996 | 11.1658341 | 8.075222671 | 1.09E-07 | 5.27E-06 | 7.927981334 |
| ATF1 | 2.607630009 | 7.560807485 | 8.075214496 | 1.09E-07 | 5.27E-06 | 7.927965932 |
| MATR3 | 2.446005325 | 9.951614419 | 8.072547064 | 1.10E-07 | 5.29E-06 | 7.922940351 |
| ZNF569 | 2.52980209 | 6.654340118 | 8.068805805 | 1.11E-07 | 5.31E-06 | 7.915889932 |
| ZNF280C | 1.553723054 | 6.159999272 | 8.065555228 | 1.11E-07 | 5.33E-06 | 7.9097626 |
| CDKN1B | 1.390417464 | 10.67565675 | 8.057782888 | 1.13E-07 | 5.38E-06 | 7.895105707 |
| ZNF728 | 2.78139171 | 4.746227733 | 8.056495685 | 1.13E-07 | 5.38E-06 | 7.892677508 |
| VSTM4 | -1.432962511 | 3.45358722 | -8.056359879 | 1.13E-07 | 5.38E-06 | 7.892421308 |
| PIK3C2A | 1.577396529 | 8.880796656 | 8.043457701 | 1.16E-07 | 5.50E-06 | 7.868069267 |
| ZNF548 | 2.542170216 | 4.921979004 | 8.032056975 | 1.19E-07 | 5.60E-06 | 7.846531555 |
| COG6 | 1.443471838 | 8.113837311 | 8.014596032 | 1.23E-07 | 5.77E-06 | 7.813509573 |
| TTLL8 | -4.305676259 | 8.186844789 | -8.013851988 | 1.23E-07 | 5.77E-06 | 7.812101487 |
| SDC4 | -1.82644754 | 7.726668311 | -8.011242613 | 1.23E-07 | 5.79E-06 | 7.807162685 |
| AMN1 | 2.059678503 | 5.7729177 | 8.007794047 | 1.24E-07 | 5.81E-06 | 7.800634059 |
| MTBP | 2.147265795 | 3.85405311 | 8.004836213 | 1.25E-07 | 5.82E-06 | 7.795033118 |
| AEBP2 | 1.914630586 | 7.567566306 | 8.002583557 | 1.25E-07 | 5.82E-06 | 7.790766669 |
| ADD3 | 1.869139667 | 11.54259158 | 8.001828568 | 1.26E-07 | 5.82E-06 | 7.789336588 |
| ANKRD13C | 2.108626597 | 5.382658352 | 7.999196803 | 1.26E-07 | 5.82E-06 | 7.78435093 |
| CEP76 | 0.968786071 | 6.132236565 | 7.997919238 | 1.27E-07 | 5.82E-06 | 7.781930339 |
| RC3H1 | 0.915103604 | 10.35604168 | 7.997874124 | 1.27E-07 | 5.82E-06 | 7.781844858 |
| NXT2 | 1.540851792 | 6.186416315 | 7.997222679 | 1.27E-07 | 5.82E-06 | 7.780610477 |
| GABPA | 2.746999944 | 8.003742331 | 7.987335709 | 1.29E-07 | 5.91E-06 | 7.761868941 |
| RICTOR | 1.745209363 | 9.662382655 | 7.986092446 | 1.29E-07 | 5.91E-06 | 7.759511258 |
| ZDHHC17 | 2.141452889 | 8.001760485 | 7.979266384 | 1.31E-07 | 5.97E-06 | 7.74656265 |
| UGDH | 2.097289409 | 6.873442968 | 7.978771054 | 1.31E-07 | 5.97E-06 | 7.745622784 |
| HSF2 | 1.34123079 | 8.578281298 | 7.974385404 | 1.32E-07 | 6.00E-06 | 7.737299699 |
| BTLA | 2.597516397 | 7.577299658 | 7.970593391 | 1.33E-07 | 6.03E-06 | 7.730101025 |
| CEP97 | 1.891582875 | 3.991546443 | 7.96677246 | 1.34E-07 | 6.06E-06 | 7.722845396 |
| ARL15 | 2.614233187 | 3.899832164 | 7.960760632 | 1.36E-07 | 6.10E-06 | 7.711425258 |
| PPP6R2 | -1.05960609 | 10.26117696 | -7.960529328 | 1.36E-07 | 6.10E-06 | 7.710985767 |
| OTUD1 | 1.156093191 | 8.887423613 | 7.952874348 | 1.38E-07 | 6.17E-06 | 7.696436625 |
| ZNF345 | 1.143965862 | 6.313556708 | 7.943645016 | 1.40E-07 | 6.27E-06 | 7.678884228 |
| C5orf24 | 2.130917886 | 6.916585158 | 7.941171951 | 1.41E-07 | 6.28E-06 | 7.674178892 |
| TMEM106B | 1.846224089 | 8.694424907 | 7.937949756 | 1.42E-07 | 6.31E-06 | 7.668046937 |
| MDC1 | 1.609019964 | 8.719154509 | 7.936169008 | 1.42E-07 | 6.31E-06 | 7.664657476 |
| FAM122B | 1.395657654 | 8.95900552 | 7.931307797 | 1.44E-07 | 6.35E-06 | 7.655402408 |
| IBTK | 1.86977938 | 9.144960413 | 7.928376906 | 1.45E-07 | 6.37E-06 | 7.649820783 |
| CBR4 | 1.798790185 | 8.581589822 | 7.917757324 | 1.48E-07 | 6.48E-06 | 7.629586535 |
| ZNF682 | 2.298107383 | 4.930614482 | 7.917136977 | 1.48E-07 | 6.48E-06 | 7.62840405 |
| ARHGEF15 | -3.528726916 | 9.279111057 | -7.914753678 | 1.48E-07 | 6.50E-06 | 7.623860579 |
| APPBP2 | 0.918232644 | 8.539317613 | 7.903539623 | 1.52E-07 | 6.59E-06 | 7.602471538 |
| TRIM36 | 1.520449463 | 3.779938248 | 7.902465282 | 1.52E-07 | 6.59E-06 | 7.600421467 |
| GPBP1 | 1.835211829 | 11.50509591 | 7.90212801 | 1.52E-07 | 6.59E-06 | 7.599777847 |
| FOPNL | 1.578405696 | 9.786748677 | 7.902043667 | 1.52E-07 | 6.59E-06 | 7.599616892 |
| EXTL2 | 1.608369408 | 3.831057286 | 7.896495544 | 1.54E-07 | 6.64E-06 | 7.589026963 |
| LUC7L3 | 2.057486095 | 10.154802 | 7.895770385 | 1.54E-07 | 6.64E-06 | 7.587642501 |
| RNF19A | 1.524784663 | 8.285313075 | 7.887806451 | 1.56E-07 | 6.72E-06 | 7.572432977 |
| SRP9 | 2.185588753 | 8.007979581 | 7.878491968 | 1.59E-07 | 6.83E-06 | 7.554632776 |
| ERCC6L | 3.118898148 | 4.290471523 | 7.877501987 | 1.60E-07 | 6.83E-06 | 7.552740176 |
| EXOC3L4 | -2.556573139 | 5.769638033 | -7.875061037 | 1.60E-07 | 6.84E-06 | 7.548073087 |
| MAP1A | -2.885605375 | 6.756685197 | -7.872763868 | 1.61E-07 | 6.86E-06 | 7.543680136 |
| MYEOV | -3.365927964 | 8.279029196 | -7.870250926 | 1.62E-07 | 6.86E-06 | 7.5388737 |
| GCA | 1.66537392 | 14.21372502 | 7.870066349 | 1.62E-07 | 6.86E-06 | 7.538520629 |
| NMS | -2.058484823 | 4.205051251 | -7.867975726 | 1.63E-07 | 6.87E-06 | 7.534521215 |
| UBR1 | 1.725506103 | 8.835047912 | 7.864565559 | 1.64E-07 | 6.90E-06 | 7.52799615 |
| SCAI | 1.951831952 | 7.093728099 | 7.853862421 | 1.67E-07 | 7.00E-06 | 7.507505903 |
| HDAC9 | 1.581827329 | 3.063100436 | 7.853673135 | 1.67E-07 | 7.00E-06 | 7.507143385 |
| ZMYM1 | 1.709142871 | 6.791537813 | 7.853224524 | 1.67E-07 | 7.00E-06 | 7.506284191 |
| NOC3L | 1.693146227 | 8.608828247 | 7.849872688 | 1.68E-07 | 7.03E-06 | 7.499863746 |
| MYLK | -1.93755747 | 7.668920569 | -7.847960991 | 1.69E-07 | 7.03E-06 | 7.496201175 |
| ATAD2B | 1.941463682 | 7.032276808 | 7.846589728 | 1.69E-07 | 7.03E-06 | 7.493573688 |
| RNF123 | -1.040531148 | 9.752379345 | -7.845360458 | 1.70E-07 | 7.03E-06 | 7.491218049 |
| ZNF268 | 1.627958033 | 8.438261134 | 7.844852301 | 1.70E-07 | 7.03E-06 | 7.490244211 |
| ATAD2 | 1.699795396 | 7.505048135 | 7.839005617 | 1.72E-07 | 7.09E-06 | 7.479036911 |
| ARAP2 | 1.869160722 | 8.665552468 | 7.836879648 | 1.73E-07 | 7.10E-06 | 7.474960516 |
| ZNF781 | 2.672180071 | 4.631689006 | 7.835113788 | 1.73E-07 | 7.10E-06 | 7.471574118 |
| ACSM5 | -5.237151831 | 8.392920007 | -7.834603039 | 1.73E-07 | 7.10E-06 | 7.470594569 |
| ARFGEF2 | 1.729344099 | 7.588840599 | 7.833482303 | 1.74E-07 | 7.10E-06 | 7.468445018 |
| GOLGA8F | 2.767101845 | 4.38286711 | 7.828489459 | 1.75E-07 | 7.13E-06 | 7.458866671 |
| LRCH1 | 1.906965414 | 4.610629113 | 7.828043014 | 1.76E-07 | 7.13E-06 | 7.458010032 |
| RCHY1 | 1.727829527 | 5.681460761 | 7.827695701 | 1.76E-07 | 7.13E-06 | 7.457343587 |
| UBE3A | 2.30763356 | 6.166024635 | 7.825820044 | 1.76E-07 | 7.14E-06 | 7.453744175 |
| REV3L | 1.302229265 | 8.993940925 | 7.817591996 | 1.79E-07 | 7.23E-06 | 7.437948539 |
| SMARCE1 | 1.317297356 | 10.17884476 | 7.817095346 | 1.79E-07 | 7.23E-06 | 7.436994797 |
| CDC42EP3 | 2.152856343 | 5.119952549 | 7.814932828 | 1.80E-07 | 7.25E-06 | 7.432841602 |
| MSMO1 | 1.39779478 | 4.009433059 | 7.811758795 | 1.81E-07 | 7.27E-06 | 7.426744551 |
| DPCD | -2.095015518 | 9.93357941 | -7.810723089 | 1.82E-07 | 7.27E-06 | 7.424754739 |
| PDE2A | -2.22738423 | 8.012279099 | -7.809557973 | 1.82E-07 | 7.27E-06 | 7.42251612 |
| HSPA13 | 2.622480095 | 7.332272671 | 7.807820638 | 1.83E-07 | 7.28E-06 | 7.4191777 |
| PCP2 | -1.095519619 | 9.231660545 | -7.80497859 | 1.84E-07 | 7.30E-06 | 7.413715566 |
| AGL | 2.477304513 | 7.987103452 | 7.803793556 | 1.84E-07 | 7.30E-06 | 7.41143771 |
| CLCNKA | -4.399022016 | 7.380036863 | -7.803132845 | 1.84E-07 | 7.30E-06 | 7.410167615 |
| WDR44 | 1.570794455 | 7.282767029 | 7.801195417 | 1.85E-07 | 7.31E-06 | 7.406442906 |
| MICALL2 | -1.466523621 | 8.372729036 | -7.795143718 | 1.87E-07 | 7.37E-06 | 7.394805083 |
| FOXO4 | -1.788876765 | 7.431645962 | -7.794286096 | 1.87E-07 | 7.37E-06 | 7.393155398 |
| TMTC4 | 1.566693256 | 7.001517181 | 7.793380172 | 1.88E-07 | 7.37E-06 | 7.391412688 |
| CENPK | 2.96052662 | 4.294063672 | 7.789355762 | 1.89E-07 | 7.40E-06 | 7.383669597 |
| GYPC | -2.104284837 | 13.74518855 | -7.78829012 | 1.90E-07 | 7.40E-06 | 7.381618884 |
| U2AF2 | 1.056675323 | 12.38038432 | 7.787830564 | 1.90E-07 | 7.40E-06 | 7.380734467 |
| OTUD4 | 1.505900017 | 6.363681259 | 7.786391897 | 1.90E-07 | 7.41E-06 | 7.377965559 |
| RRAS2 | 2.410261832 | 8.502482184 | 7.778929904 | 1.93E-07 | 7.50E-06 | 7.363599235 |
| ABHD3 | 1.839006453 | 8.92406546 | 7.777487212 | 1.94E-07 | 7.50E-06 | 7.360820759 |
| TWSG1 | 2.153366361 | 3.479358043 | 7.768759935 | 1.97E-07 | 7.61E-06 | 7.344006625 |
| TMEM86B | -1.927559562 | 10.22802531 | -7.765894255 | 1.98E-07 | 7.62E-06 | 7.338483196 |
| SEC23A | 1.696353909 | 8.324669863 | 7.765886417 | 1.98E-07 | 7.62E-06 | 7.338468088 |
| ZFP62 | 1.432828196 | 9.272926601 | 7.763914995 | 1.99E-07 | 7.62E-06 | 7.33466761 |
| COX11 | 1.135175734 | 8.384637049 | 7.763777145 | 1.99E-07 | 7.62E-06 | 7.334401846 |
| PPP1CC | 1.046794346 | 10.59167498 | 7.759563504 | 2.01E-07 | 7.65E-06 | 7.326276923 |
| IKZF3 | 2.948925871 | 6.783483392 | 7.758696786 | 2.01E-07 | 7.65E-06 | 7.324605366 |
| FBXW7 | 1.519286049 | 9.335822099 | 7.758367258 | 2.01E-07 | 7.65E-06 | 7.32396981 |
| HINT3 | 2.029730421 | 8.140467281 | 7.756632236 | 2.02E-07 | 7.66E-06 | 7.32062324 |
| CD47 | 1.108590148 | 12.04646934 | 7.754911377 | 2.02E-07 | 7.67E-06 | 7.317303567 |
| SERPING1 | -2.587024666 | 8.021173959 | -7.750545953 | 2.04E-07 | 7.71E-06 | 7.308880434 |
| SPOPL | 1.726319118 | 8.621289829 | 7.750060161 | 2.04E-07 | 7.71E-06 | 7.307942925 |
| TMEM64 | 1.507081468 | 5.71491047 | 7.742964873 | 2.07E-07 | 7.79E-06 | 7.294246234 |
| ABI1 | 1.289210496 | 8.828739273 | 7.742743233 | 2.07E-07 | 7.79E-06 | 7.293818268 |
| CHD9 | 1.307231647 | 9.741020292 | 7.739666033 | 2.08E-07 | 7.81E-06 | 7.287875747 |
| RBM12 | 1.93867696 | 7.895567062 | 7.739062543 | 2.09E-07 | 7.81E-06 | 7.286710163 |
| ABCD2 | 2.915140019 | 4.536331461 | 7.736562097 | 2.10E-07 | 7.83E-06 | 7.281880234 |
| ZNF621 | 1.627429793 | 7.903411074 | 7.717763102 | 2.18E-07 | 8.11E-06 | 7.245539201 |
| ZFC3H1 | 1.088823328 | 10.04356533 | 7.716273502 | 2.18E-07 | 8.11E-06 | 7.242657457 |
| RBM3 | 1.378789067 | 9.559585086 | 7.710306019 | 2.21E-07 | 8.19E-06 | 7.231109752 |
| ANKRD46 | 1.570023511 | 6.258269485 | 7.7093995 | 2.21E-07 | 8.19E-06 | 7.229355099 |
| DNAJB2 | -1.461701206 | 12.54776109 | -7.707775306 | 2.22E-07 | 8.20E-06 | 7.226211031 |
| CCDC3 | -2.564930823 | 5.151881895 | -7.704903571 | 2.23E-07 | 8.22E-06 | 7.220651091 |
| ECSIT | -1.327980487 | 10.90349487 | -7.703307212 | 2.24E-07 | 8.23E-06 | 7.217559889 |
| POLK | 1.21810382 | 8.649448331 | 7.69845584 | 2.26E-07 | 8.29E-06 | 7.208163437 |
| RNF13 | 1.583680735 | 11.03011195 | 7.697424254 | 2.26E-07 | 8.29E-06 | 7.206164965 |
| ERCC2 | -3.200517221 | 8.850787117 | -7.695948321 | 2.27E-07 | 8.30E-06 | 7.203305403 |
| CTGF | -2.719906315 | 3.885529205 | -7.687252575 | 2.31E-07 | 8.41E-06 | 7.18645147 |
| DCLRE1A | 1.469623247 | 7.668048659 | 7.687051009 | 2.31E-07 | 8.41E-06 | 7.186060672 |
| CAMK2N1 | 1.722917321 | 5.896686234 | 7.683115849 | 2.33E-07 | 8.44E-06 | 7.178429979 |
| MDFIC | 1.629112265 | 8.14193321 | 7.68139859 | 2.34E-07 | 8.44E-06 | 7.175099344 |
| RILP | -2.726055221 | 9.746884672 | -7.680618605 | 2.34E-07 | 8.44E-06 | 7.17358642 |
| DEK | 1.836431836 | 9.643080296 | 7.678745875 | 2.35E-07 | 8.44E-06 | 7.169953563 |
| C2orf69 | 1.709792352 | 6.762888961 | 7.678408427 | 2.35E-07 | 8.44E-06 | 7.169298904 |
| RIF1 | 2.394114141 | 6.855575358 | 7.678116926 | 2.35E-07 | 8.44E-06 | 7.168733371 |
| KBTBD3 | 2.696483292 | 5.6716096 | 7.676724774 | 2.36E-07 | 8.44E-06 | 7.166032328 |
| ZNF566 | 2.561178029 | 4.335105015 | 7.67663468 | 2.36E-07 | 8.44E-06 | 7.165857518 |
| REEP3 | 2.697012784 | 4.391929414 | 7.670623494 | 2.39E-07 | 8.53E-06 | 7.154191425 |
| ZNF684 | 1.962312773 | 4.917454137 | 7.668883021 | 2.39E-07 | 8.54E-06 | 7.150812678 |
| ETAA1 | 2.191357386 | 7.731330686 | 7.665509417 | 2.41E-07 | 8.58E-06 | 7.144262343 |
| RPS3 | 1.076591415 | 14.09912555 | 7.661209275 | 2.43E-07 | 8.62E-06 | 7.135910661 |
| PAQR3 | 1.238148989 | 5.826917611 | 7.661096516 | 2.43E-07 | 8.62E-06 | 7.135691626 |
| CLK1 | 2.346910688 | 10.50570693 | 7.656419747 | 2.45E-07 | 8.68E-06 | 7.126605411 |
| GALNT7 | 1.79985341 | 8.876159205 | 7.653794973 | 2.47E-07 | 8.71E-06 | 7.121504536 |
| DENND4A | 3.193634611 | 5.795618828 | 7.644213818 | 2.51E-07 | 8.85E-06 | 7.102876625 |
| ATP11C | 0.997980525 | 7.308466198 | 7.642132557 | 2.52E-07 | 8.86E-06 | 7.098828467 |
| HSPD1 | 1.349091053 | 12.73651265 | 7.642085255 | 2.52E-07 | 8.86E-06 | 7.098736456 |
| PEX3 | 1.950268743 | 6.853074694 | 7.636520582 | 2.55E-07 | 8.92E-06 | 7.087909828 |
| SPTSSA | 1.299217116 | 8.805064015 | 7.636361616 | 2.55E-07 | 8.92E-06 | 7.08760048 |
| USP1 | 1.743271672 | 9.093450511 | 7.634685294 | 2.56E-07 | 8.93E-06 | 7.084338128 |
| PIGY | 1.194842923 | 10.40278912 | 7.624825807 | 2.61E-07 | 9.09E-06 | 7.065142159 |
| PRKAR1B | -1.799100647 | 7.029497582 | -7.620942575 | 2.63E-07 | 9.12E-06 | 7.057577899 |
| ZNF790 | 1.989401381 | 3.9962298 | 7.620927417 | 2.63E-07 | 9.12E-06 | 7.057548369 |
| HELQ | 1.607619036 | 8.069129899 | 7.619651087 | 2.64E-07 | 9.13E-06 | 7.0550617 |
| ZNF320 | 1.977595041 | 6.642767131 | 7.611827292 | 2.68E-07 | 9.24E-06 | 7.039813574 |
| ROBO3 | -2.48655325 | 8.558999923 | -7.611491264 | 2.68E-07 | 9.24E-06 | 7.039158482 |
| GPR180 | 1.294760649 | 6.583112225 | 7.607559079 | 2.70E-07 | 9.27E-06 | 7.031491413 |
| SENP6 | 2.690779004 | 5.535781172 | 7.606930234 | 2.70E-07 | 9.27E-06 | 7.030265074 |
| LARP1B | 1.796277615 | 6.447492826 | 7.606832378 | 2.70E-07 | 9.27E-06 | 7.030074235 |
| RAB10 | 1.389535147 | 11.18257079 | 7.602384957 | 2.73E-07 | 9.33E-06 | 7.021399449 |
| FASTKD1 | 1.103635751 | 8.937305805 | 7.598775485 | 2.75E-07 | 9.38E-06 | 7.014357035 |
| PRNP | 1.796296098 | 10.52965188 | 7.596158903 | 2.76E-07 | 9.40E-06 | 7.009250688 |
| DMTF1 | 1.418944641 | 9.277680855 | 7.595542394 | 2.76E-07 | 9.40E-06 | 7.008047408 |
| C1GALT1 | 1.598992675 | 7.70257801 | 7.591601653 | 2.79E-07 | 9.45E-06 | 7.000354742 |
| SLC6A8 | -3.025664541 | 8.918645067 | -7.590964883 | 2.79E-07 | 9.45E-06 | 6.999111505 |
| CASC5 | 2.969860222 | 4.968673393 | 7.585962326 | 2.82E-07 | 9.53E-06 | 6.98934246 |
| ZCCHC10 | 2.57456558 | 7.519831985 | 7.580642135 | 2.85E-07 | 9.59E-06 | 6.97894924 |
| N4BP2L2 | 1.29741114 | 11.86744503 | 7.580467143 | 2.85E-07 | 9.59E-06 | 6.978607317 |
| ZNF141 | 3.716007345 | 7.750318578 | 7.578398603 | 2.86E-07 | 9.62E-06 | 6.974565202 |
| CMPK1 | 2.416216324 | 7.401279157 | 7.570207097 | 2.91E-07 | 9.72E-06 | 6.958552294 |
| RPS6KA3 | 1.273363261 | 10.56683663 | 7.569789548 | 2.91E-07 | 9.72E-06 | 6.957735805 |
| EPHB2 | -2.653039653 | 7.678877738 | -7.569048271 | 2.91E-07 | 9.72E-06 | 6.95628623 |
| ACCSL | -3.105341286 | 5.373102659 | -7.568961055 | 2.91E-07 | 9.72E-06 | 6.956115673 |
| TACR1 | -4.21298386 | 5.581091829 | -7.568219592 | 2.92E-07 | 9.72E-06 | 6.954665647 |
| AKAP11 | 1.446332494 | 8.95256813 | 7.566067232 | 2.93E-07 | 9.73E-06 | 6.950455988 |
| RBMS1 | 1.046769691 | 12.08603986 | 7.565480369 | 2.93E-07 | 9.73E-06 | 6.949308068 |
| TMPO | 2.764760346 | 6.203278284 | 7.563932885 | 2.94E-07 | 9.74E-06 | 6.946280912 |
| USP25 | 2.00665623 | 6.176490103 | 7.559420279 | 2.97E-07 | 9.81E-06 | 6.937451506 |
| GMFB | 1.859874376 | 7.769142711 | 7.555442041 | 2.99E-07 | 9.86E-06 | 6.929665256 |
| KLHL5 | 1.077946452 | 6.832975605 | 7.555182894 | 2.99E-07 | 9.86E-06 | 6.929157973 |
| CDNF | 1.620461996 | 3.068427668 | 7.552324038 | 3.01E-07 | 9.88E-06 | 6.923561098 |
| ERBB2IP | 1.534427585 | 10.57504965 | 7.552210775 | 3.01E-07 | 9.88E-06 | 6.923339336 |
| FBXO7 | -1.987175875 | 14.77413466 | -7.547936806 | 3.04E-07 | 9.95E-06 | 6.914969818 |
| CASD1 | 2.102192137 | 6.165850269 | 7.535494368 | 3.11E-07 | 1.02E-05 | 6.890589623 |
| NAA16 | 1.616726145 | 7.745840552 | 7.53459293 | 3.12E-07 | 1.02E-05 | 6.888822458 |
| ANKIB1 | 2.21324342 | 5.472493641 | 7.533708517 | 3.12E-07 | 1.02E-05 | 6.887088557 |
| SMC6 | 2.082541691 | 8.083975409 | 7.531251242 | 3.14E-07 | 1.02E-05 | 6.88227046 |
| MFN1 | 1.386611482 | 8.295846546 | 7.530000783 | 3.15E-07 | 1.02E-05 | 6.879818296 |
| NAA11 | -1.269699391 | 8.845683527 | -7.527177598 | 3.16E-07 | 1.02E-05 | 6.874281184 |
| DICER1 | 1.099149306 | 10.72484567 | 7.524627744 | 3.18E-07 | 1.03E-05 | 6.869279186 |
| MPP6 | 2.385338403 | 5.279018357 | 7.523595088 | 3.19E-07 | 1.03E-05 | 6.867253183 |
| LY6G6F | -2.65229367 | 8.531698092 | -7.523135928 | 3.19E-07 | 1.03E-05 | 6.866352292 |
| ACP2 | -2.263345834 | 7.225557485 | -7.520435468 | 3.21E-07 | 1.03E-05 | 6.861053279 |
| USP37 | 1.045916413 | 7.583640272 | 7.517833883 | 3.22E-07 | 1.03E-05 | 6.855947305 |
| PMS1 | 2.045698944 | 6.941642723 | 7.513573463 | 3.25E-07 | 1.04E-05 | 6.847583565 |
| ZMYND15 | -1.006383523 | 8.945384482 | -7.505140088 | 3.31E-07 | 1.06E-05 | 6.831020204 |
| TMEM161B | 1.577367632 | 8.274465737 | 7.500942953 | 3.33E-07 | 1.06E-05 | 6.822773171 |
| ANKFY1 | -1.372340756 | 8.936502686 | -7.495710687 | 3.37E-07 | 1.07E-05 | 6.812488692 |
| SRRD | -1.574458426 | 9.347935248 | -7.49569406 | 3.37E-07 | 1.07E-05 | 6.812456004 |
| CAPZA1 | 1.163375945 | 11.32134023 | 7.486069797 | 3.43E-07 | 1.09E-05 | 6.793528517 |
| MTMR9 | 1.077905665 | 7.982044386 | 7.484759048 | 3.44E-07 | 1.09E-05 | 6.790949727 |
| ZNF286A | 2.103304868 | 3.155592357 | 7.484549073 | 3.44E-07 | 1.09E-05 | 6.790536595 |
| PARD3 | -1.741315065 | 5.409806422 | -7.478515914 | 3.48E-07 | 1.10E-05 | 6.778663536 |
| MUSK | -1.869647623 | 7.566263958 | -7.472520243 | 3.53E-07 | 1.11E-05 | 6.766859144 |
| HNRNPA1 | 1.146327032 | 12.84034125 | 7.470574322 | 3.54E-07 | 1.11E-05 | 6.763026884 |
| LAPTM4A | 0.910974768 | 12.90227844 | 7.470466254 | 3.54E-07 | 1.11E-05 | 6.762814041 |
| TRIM22 | 1.665706051 | 10.70878243 | 7.467901902 | 3.56E-07 | 1.12E-05 | 6.757762996 |
| EIF1AX | 0.821563794 | 11.55415178 | 7.466832087 | 3.57E-07 | 1.12E-05 | 6.755655488 |
| WSB1 | 0.822536493 | 10.85154143 | 7.465075854 | 3.58E-07 | 1.12E-05 | 6.752195405 |
| CBWD5 | 1.412014877 | 10.42965694 | 7.459568704 | 3.62E-07 | 1.13E-05 | 6.741342531 |
| WDR45 | -1.410432697 | 10.69614048 | -7.459363545 | 3.62E-07 | 1.13E-05 | 6.740938144 |
| GLS | 1.083913752 | 9.944204199 | 7.456187165 | 3.64E-07 | 1.13E-05 | 6.734676445 |
| USP53 | 1.897702 | 6.944364254 | 7.455948456 | 3.64E-07 | 1.13E-05 | 6.734205812 |
| RAPSN | -2.346787608 | 6.528447064 | -7.45239984 | 3.67E-07 | 1.14E-05 | 6.727208503 |
| DCP2 | 1.653555956 | 9.482317403 | 7.446831885 | 3.71E-07 | 1.15E-05 | 6.716225786 |
| IFT80 | 1.602502785 | 6.4841117 | 7.445841146 | 3.72E-07 | 1.15E-05 | 6.714271104 |
| EMILIN3 | -2.308719313 | 7.594480936 | -7.439575879 | 3.76E-07 | 1.16E-05 | 6.701906821 |
| KIAA0408 | 1.921885824 | 5.48398128 | 7.439547442 | 3.77E-07 | 1.16E-05 | 6.701850688 |
| LGALS12 | -1.851002063 | 10.05210376 | -7.439477438 | 3.77E-07 | 1.16E-05 | 6.701712507 |
| ALG10B | 1.349117193 | 7.876680275 | 7.436345321 | 3.79E-07 | 1.16E-05 | 6.695529256 |
| EPHA8 | -1.676625587 | 5.21663565 | -7.434752907 | 3.80E-07 | 1.16E-05 | 6.692385071 |
| HNRNPH1 | 1.347912578 | 9.54020326 | 7.434214438 | 3.81E-07 | 1.16E-05 | 6.691321794 |
| SLC38A5 | -2.674468982 | 10.03764599 | -7.431668098 | 3.82E-07 | 1.17E-05 | 6.68629316 |
| HMGB4 | -3.080436045 | 6.660044303 | -7.427433273 | 3.86E-07 | 1.17E-05 | 6.677927996 |
| SETDB2 | 1.452264193 | 10.23280436 | 7.41324575 | 3.97E-07 | 1.20E-05 | 6.649884504 |
| TAF1A | 1.546232885 | 6.668002523 | 7.41128428 | 3.98E-07 | 1.20E-05 | 6.646005161 |
| CENPQ | 1.925041615 | 6.240552781 | 7.411121706 | 3.98E-07 | 1.20E-05 | 6.645683602 |
| TMEM55A | 1.537984447 | 10.02944971 | 7.410770562 | 3.99E-07 | 1.20E-05 | 6.644989053 |
| RAB28 | 1.406400736 | 7.838504452 | 7.40783618 | 4.01E-07 | 1.20E-05 | 6.639184282 |
| POLL | -2.011163301 | 11.58967582 | -7.407552233 | 4.01E-07 | 1.20E-05 | 6.638622516 |
| ZNF225 | 1.891473958 | 5.29460623 | 7.407509296 | 4.01E-07 | 1.20E-05 | 6.638537568 |
| SEC14L1 | -1.021726189 | 12.31890723 | -7.407291183 | 4.02E-07 | 1.20E-05 | 6.63810604 |
| ARL5A | 2.700817477 | 6.99199152 | 7.406221073 | 4.02E-07 | 1.21E-05 | 6.635988772 |
| NAA15 | 2.387818283 | 4.389205036 | 7.395593823 | 4.11E-07 | 1.23E-05 | 6.614953409 |
| HECA | 1.516473595 | 12.7165133 | 7.394803298 | 4.12E-07 | 1.23E-05 | 6.613388022 |
| SPOCD1 | -3.423698259 | 7.581063277 | -7.392775002 | 4.13E-07 | 1.23E-05 | 6.609371212 |
| ZNF780B | 3.765646913 | 5.258400255 | 7.390824873 | 4.15E-07 | 1.23E-05 | 6.605508654 |
| WDR36 | 1.899700875 | 6.460679967 | 7.388367292 | 4.17E-07 | 1.24E-05 | 6.600640238 |
| KCNE1 | -1.184967026 | 4.676570556 | -7.385753659 | 4.19E-07 | 1.24E-05 | 6.595461748 |
| BRWD1 | 1.862820049 | 8.139532197 | 7.384257247 | 4.20E-07 | 1.24E-05 | 6.592496415 |
| NKTR | 1.126305991 | 11.26920807 | 7.383493427 | 4.21E-07 | 1.24E-05 | 6.590982686 |
| CHM | 1.15448496 | 7.11836798 | 7.379130356 | 4.25E-07 | 1.25E-05 | 6.582334421 |
| FIGNL1 | 2.321450919 | 4.158840926 | 7.376499976 | 4.27E-07 | 1.26E-05 | 6.577119313 |
| RSBN1 | 1.219942731 | 9.376266868 | 7.375891983 | 4.27E-07 | 1.26E-05 | 6.575913739 |
| G3BP2 | 1.054207189 | 10.74943357 | 7.374520628 | 4.29E-07 | 1.26E-05 | 6.573194324 |
| ZDHHC2 | 1.432471262 | 8.511062326 | 7.367946977 | 4.34E-07 | 1.27E-05 | 6.560154992 |
| FAM98A | 1.435604725 | 6.373154306 | 7.366187251 | 4.36E-07 | 1.28E-05 | 6.556663407 |
| C11orf58 | 1.561438824 | 9.981811085 | 7.364743205 | 4.37E-07 | 1.28E-05 | 6.553797856 |
| FHL5 | -2.043483477 | 3.419647918 | -7.363614613 | 4.38E-07 | 1.28E-05 | 6.551558085 |
| HMBS | -1.623037817 | 12.9384487 | -7.357646033 | 4.43E-07 | 1.29E-05 | 6.539710013 |
| EED | 1.33490681 | 10.61384356 | 7.356587251 | 4.44E-07 | 1.29E-05 | 6.537607728 |
| ETV3L | -2.811898654 | 4.003730273 | -7.354230923 | 4.46E-07 | 1.29E-05 | 6.532928506 |
| FAM200A | 1.66926586 | 4.964741777 | 7.353704874 | 4.47E-07 | 1.29E-05 | 6.531883765 |
| NR1D2 | 1.977200245 | 5.231044466 | 7.351735643 | 4.49E-07 | 1.30E-05 | 6.527972495 |
| POLR1D | -1.233457187 | 10.8819607 | -7.34852867 | 4.52E-07 | 1.30E-05 | 6.52160166 |
| MANEA | 1.999920298 | 6.541210797 | 7.348338368 | 4.52E-07 | 1.30E-05 | 6.52122357 |
| SLC25A46 | 1.899338992 | 7.116237327 | 7.345776642 | 4.54E-07 | 1.31E-05 | 6.516133442 |
| GNA13 | 1.298643616 | 10.01458338 | 7.344535162 | 4.55E-07 | 1.31E-05 | 6.5136663 |
| CENPB | -1.02465377 | 8.097644154 | -7.343346423 | 4.56E-07 | 1.31E-05 | 6.511303762 |
| OSTM1 | 1.545632847 | 6.75811075 | 7.337648499 | 4.61E-07 | 1.32E-05 | 6.499976759 |
| FKBP1B | -1.680189237 | 9.283946254 | -7.335703679 | 4.63E-07 | 1.32E-05 | 6.496109566 |
| GPX1 | -1.241508868 | 16.79447978 | -7.332857571 | 4.66E-07 | 1.33E-05 | 6.490449239 |
| NAA50 | 1.488936606 | 11.04813258 | 7.330958496 | 4.68E-07 | 1.33E-05 | 6.486671732 |
| WNT4 | -4.73927681 | 6.71647536 | -7.329737888 | 4.69E-07 | 1.33E-05 | 6.484243514 |
| PIKFYVE | 1.633657333 | 6.983880861 | 7.32690649 | 4.72E-07 | 1.34E-05 | 6.478610061 |
| NKRF | 1.021024184 | 8.98272993 | 7.325794028 | 4.73E-07 | 1.34E-05 | 6.476396355 |
| GP1BB | -2.300099606 | 14.25006506 | -7.321014989 | 4.77E-07 | 1.35E-05 | 6.466884487 |
| U2SURP | 1.865901438 | 6.729126381 | 7.320975112 | 4.77E-07 | 1.35E-05 | 6.466805103 |
| KCNIP4 | 1.323110707 | 2.735702433 | 7.316105921 | 4.82E-07 | 1.36E-05 | 6.457110459 |
| ARGLU1 | 3.297013257 | 8.780291197 | 7.315589236 | 4.82E-07 | 1.36E-05 | 6.456081534 |
| ZNF721 | 2.29378773 | 8.463137412 | 7.313390091 | 4.84E-07 | 1.36E-05 | 6.45170174 |
| MBNL1 | 1.448271631 | 13.74022601 | 7.310561907 | 4.87E-07 | 1.37E-05 | 6.446068157 |
| ARL10 | 1.48012472 | 3.956863406 | 7.308116471 | 4.90E-07 | 1.37E-05 | 6.441196075 |
| KIF18A | 1.572159539 | 3.153784917 | 7.306189962 | 4.92E-07 | 1.38E-05 | 6.437357267 |
| DUT | 0.918491896 | 12.34773561 | 7.305637277 | 4.92E-07 | 1.38E-05 | 6.436255876 |
| SLC4A7 | 2.354124956 | 6.721702132 | 7.304469859 | 4.93E-07 | 1.38E-05 | 6.433929304 |
| ZNF69 | 1.486323307 | 3.871748723 | 7.303739131 | 4.94E-07 | 1.38E-05 | 6.432472924 |
| TMED10 | 1.115261918 | 11.59538128 | 7.299951288 | 4.98E-07 | 1.38E-05 | 6.424922339 |
| ZNF836 | 1.51836511 | 6.300420253 | 7.298108757 | 5.00E-07 | 1.39E-05 | 6.421248756 |
| UBLCP1 | 1.370178452 | 10.33478688 | 7.296118858 | 5.02E-07 | 1.39E-05 | 6.417280815 |
| FAAH | -2.499557201 | 7.034484595 | -7.295705244 | 5.02E-07 | 1.39E-05 | 6.416455982 |
| DUSP13 | -2.205391886 | 7.025431195 | -7.295224362 | 5.02E-07 | 1.39E-05 | 6.415496972 |
| SGTB | 1.688853341 | 8.954572295 | 7.294225733 | 5.04E-07 | 1.39E-05 | 6.41350533 |
| TMEM234 | -0.848307929 | 8.276746589 | -7.287099847 | 5.11E-07 | 1.41E-05 | 6.399289539 |
| KRTAP5-11 | -1.590673869 | 8.982563797 | -7.286737583 | 5.11E-07 | 1.41E-05 | 6.39856665 |
| PRAM1 | -1.037080179 | 13.98688574 | -7.28533677 | 5.13E-07 | 1.41E-05 | 6.395771183 |
| ZNF354B | 2.536787243 | 6.45880705 | 7.281592366 | 5.16E-07 | 1.42E-05 | 6.388297483 |
| IER3IP1 | 1.772036628 | 7.144406689 | 7.279562603 | 5.19E-07 | 1.42E-05 | 6.384245321 |
| C12orf29 | 1.774690762 | 7.548678127 | 7.277534952 | 5.21E-07 | 1.42E-05 | 6.380196795 |
| TMEM200A | 2.287508061 | 4.86439274 | 7.273128113 | 5.25E-07 | 1.43E-05 | 6.371395843 |
| ZNF706 | 2.562430158 | 4.851809605 | 7.269160875 | 5.30E-07 | 1.44E-05 | 6.363470486 |
| SEC14L4 | -2.295728117 | 7.292335684 | -7.266811882 | 5.32E-07 | 1.45E-05 | 6.358776852 |
| SRC | -1.422369651 | 7.584463816 | -7.258867092 | 5.41E-07 | 1.47E-05 | 6.342896233 |
| TADA2A | 1.701365383 | 5.340865504 | 7.258321087 | 5.41E-07 | 1.47E-05 | 6.341804512 |
| SPRED1 | 1.842463677 | 4.498920317 | 7.257594535 | 5.42E-07 | 1.47E-05 | 6.340351728 |
| RNF138 | 1.863884381 | 9.065375802 | 7.254522845 | 5.45E-07 | 1.48E-05 | 6.33420888 |
| KRTAP9-7 | -2.781092839 | 6.638945535 | -7.251986626 | 5.48E-07 | 1.48E-05 | 6.329135879 |
| TUBE1 | 1.809597417 | 6.689450511 | 7.251090006 | 5.49E-07 | 1.48E-05 | 6.327342223 |
| ABCE1 | 1.161521275 | 8.200340187 | 7.251012389 | 5.49E-07 | 1.48E-05 | 6.327186948 |
| GSN | -1.411652422 | 11.73793702 | -7.250991595 | 5.49E-07 | 1.48E-05 | 6.327145348 |
| OTUD6B | 2.314810512 | 5.354088282 | 7.250092496 | 5.50E-07 | 1.48E-05 | 6.325346606 |
| SREK1 | 2.375764982 | 8.487486495 | 7.249973617 | 5.50E-07 | 1.48E-05 | 6.325108769 |
| TMEM9B | 0.76020145 | 13.32755834 | 7.245813193 | 5.55E-07 | 1.49E-05 | 6.316783854 |
| ASPSCR1 | -0.954234372 | 9.729552049 | -7.239995001 | 5.62E-07 | 1.50E-05 | 6.3051377 |
| TRA2B | 1.153628391 | 10.60540092 | 7.236603284 | 5.65E-07 | 1.51E-05 | 6.29834637 |
| USP45 | 1.929425663 | 5.32048938 | 7.234341672 | 5.68E-07 | 1.51E-05 | 6.293816981 |
| ACADSB | 1.935032413 | 8.025259651 | 7.231012995 | 5.72E-07 | 1.52E-05 | 6.287149246 |
| SMAP1 | 1.172722148 | 7.571707597 | 7.228555375 | 5.75E-07 | 1.53E-05 | 6.282225339 |
| VEGFC | -1.589434206 | 3.874134737 | -7.227638795 | 5.76E-07 | 1.53E-05 | 6.280388731 |
| FMR1 | 1.757092801 | 7.744100442 | 7.227551801 | 5.76E-07 | 1.53E-05 | 6.280214409 |
| GHRL | -1.576129615 | 10.11181162 | -7.226597803 | 5.77E-07 | 1.53E-05 | 6.278302686 |
| GDF5 | -3.150103355 | 6.672961572 | -7.224785732 | 5.79E-07 | 1.53E-05 | 6.274671111 |
| CDKN2AIP | 1.439258732 | 7.638195788 | 7.224108019 | 5.80E-07 | 1.53E-05 | 6.273312785 |
| NAA38 | 2.214474801 | 9.823415133 | 7.223771828 | 5.80E-07 | 1.53E-05 | 6.272638942 |
| NOX4 | -1.583322664 | 3.617048319 | -7.2236254 | 5.80E-07 | 1.53E-05 | 6.272345444 |
| UBA5 | 1.759780761 | 6.905555676 | 7.221960611 | 5.82E-07 | 1.53E-05 | 6.269008357 |
| SSX3 | -2.695641851 | 5.558656644 | -7.221897257 | 5.82E-07 | 1.53E-05 | 6.268881356 |
| HCFC2 | 1.633106473 | 7.020555077 | 7.221510034 | 5.83E-07 | 1.53E-05 | 6.268105106 |
| GATA1 | -3.10559057 | 9.156535594 | -7.216348156 | 5.89E-07 | 1.54E-05 | 6.257755265 |
| ASCC2 | -2.103395023 | 10.27195007 | -7.215386456 | 5.90E-07 | 1.54E-05 | 6.255826591 |
| GOLT1B | 1.920978064 | 7.26251665 | 7.214113694 | 5.92E-07 | 1.54E-05 | 6.253273888 |
| ZMYM4 | 0.804302117 | 8.056566813 | 7.214009578 | 5.92E-07 | 1.54E-05 | 6.253065059 |
| TMEM50B | 0.925932811 | 9.366557997 | 7.213172531 | 5.93E-07 | 1.54E-05 | 6.251386106 |
| NDFIP2 | 2.26656633 | 4.062985765 | 7.211132203 | 5.95E-07 | 1.54E-05 | 6.247293197 |
| RAD21 | 1.504024333 | 11.28316643 | 7.210852261 | 5.96E-07 | 1.54E-05 | 6.246731584 |
| TSPO2 | -2.542087272 | 9.075479091 | -7.209183879 | 5.98E-07 | 1.55E-05 | 6.2433843 |
| MORN3 | -1.329708594 | 6.957083151 | -7.207781339 | 5.99E-07 | 1.55E-05 | 6.240570072 |
| GP9 | -2.268129731 | 12.68622396 | -7.20609006 | 6.01E-07 | 1.55E-05 | 6.237176115 |
| IFNG | 2.535799347 | 4.956072537 | 7.204200408 | 6.04E-07 | 1.55E-05 | 6.233383601 |
| ACRBP | -2.074043915 | 12.05584265 | -7.202533654 | 6.06E-07 | 1.56E-05 | 6.230038023 |
| ALOX12 | -2.630580207 | 7.663605273 | -7.199940223 | 6.09E-07 | 1.56E-05 | 6.224831604 |
| SYCP1 | -1.876122098 | 6.00482156 | -7.196857357 | 6.13E-07 | 1.57E-05 | 6.218641396 |
| NDUFV3 | -0.942670445 | 11.7342451 | -7.192727451 | 6.18E-07 | 1.58E-05 | 6.2103467 |
| AVPI1 | -1.431554897 | 7.893584434 | -7.191039243 | 6.20E-07 | 1.58E-05 | 6.206955336 |
| KLF12 | 1.367367755 | 9.893268366 | 7.188825847 | 6.23E-07 | 1.59E-05 | 6.202508336 |
| C4orf46 | 1.173583466 | 6.918573856 | 7.186862928 | 6.25E-07 | 1.59E-05 | 6.198564004 |
| ARID2 | 0.782237919 | 8.503523313 | 7.185888073 | 6.26E-07 | 1.59E-05 | 6.196604906 |
| PLCL1 | 2.070176473 | 4.847052533 | 7.184762832 | 6.28E-07 | 1.60E-05 | 6.194343424 |
| GOLPH3L | 0.897275085 | 9.083063842 | 7.178568669 | 6.36E-07 | 1.61E-05 | 6.181891359 |
| BRD4 | -1.243773384 | 10.06189088 | -7.178050701 | 6.36E-07 | 1.61E-05 | 6.180849849 |
| COL4A3BP | 0.993890975 | 10.02217642 | 7.177736623 | 6.37E-07 | 1.61E-05 | 6.180218295 |
| ADCY4 | -1.56413794 | 9.558603099 | -7.175616542 | 6.40E-07 | 1.61E-05 | 6.175954832 |
| CAPN5 | -1.428225621 | 9.101271902 | -7.175126376 | 6.40E-07 | 1.61E-05 | 6.174969022 |
| AFTPH | 0.823283602 | 9.897360284 | 7.174894949 | 6.41E-07 | 1.61E-05 | 6.174503569 |
| LIMA1 | 1.859730959 | 7.604872597 | 7.173904287 | 6.42E-07 | 1.61E-05 | 6.172511041 |
| TRIOBP | -1.711793619 | 8.664170987 | -7.170046194 | 6.47E-07 | 1.62E-05 | 6.164749905 |
| ZNF605 | 1.489963649 | 6.323927867 | 7.169983034 | 6.47E-07 | 1.62E-05 | 6.164622833 |
| ATG4C | 1.659953638 | 6.163601058 | 7.169961386 | 6.47E-07 | 1.62E-05 | 6.164579278 |
| YY1 | 1.393089057 | 10.76384852 | 7.167183383 | 6.51E-07 | 1.63E-05 | 6.158989579 |
| CBFB | 0.965006554 | 9.594773466 | 7.165547747 | 6.53E-07 | 1.63E-05 | 6.155697962 |
| SPDYE5 | 2.72016132 | 4.749894241 | 7.164711914 | 6.54E-07 | 1.63E-05 | 6.154015755 |
| GPRIN3 | 2.705978357 | 5.051071941 | 7.163288766 | 6.56E-07 | 1.63E-05 | 6.151151285 |
| SLC38A9 | 1.009238722 | 9.330717188 | 7.162656638 | 6.57E-07 | 1.63E-05 | 6.149878866 |
| SLC4A1 | -2.171536605 | 9.293393587 | -7.16116203 | 6.59E-07 | 1.64E-05 | 6.146870125 |
| KIRREL3 | -3.523623157 | 7.349646493 | -7.15585015 | 6.66E-07 | 1.65E-05 | 6.136174438 |
| DOCK11 | 1.525027524 | 10.624138 | 7.154777996 | 6.67E-07 | 1.65E-05 | 6.13401513 |
| ANKRD9 | -2.405076681 | 10.24234943 | -7.15458746 | 6.67E-07 | 1.65E-05 | 6.133631377 |
| DNAJC25 | 2.330933789 | 4.456799943 | 7.150154397 | 6.74E-07 | 1.66E-05 | 6.124701409 |
| HSPH1 | 1.044103382 | 10.12233732 | 7.149708491 | 6.74E-07 | 1.66E-05 | 6.123803022 |
| OPRL1 | -2.524142989 | 7.432790838 | -7.149643251 | 6.74E-07 | 1.66E-05 | 6.123671577 |
| GTPBP8 | 0.989280909 | 10.73423925 | 7.148883835 | 6.75E-07 | 1.66E-05 | 6.122141479 |
| YY1AP1 | -1.126337397 | 12.41525675 | -7.147474214 | 6.77E-07 | 1.66E-05 | 6.119301107 |
| SLC25A39 | -2.656334823 | 15.31372451 | -7.14695588 | 6.78E-07 | 1.66E-05 | 6.118256599 |
| ANKRD26 | 2.088882541 | 5.319985781 | 7.146292015 | 6.79E-07 | 1.66E-05 | 6.116918774 |
| CD2AP | 1.985767525 | 4.635710769 | 7.144164802 | 6.82E-07 | 1.67E-05 | 6.112631583 |
| CD69 | 2.647090802 | 7.39581612 | 7.142476708 | 6.84E-07 | 1.67E-05 | 6.109228942 |
| KATNAL1 | 1.121463096 | 7.283717892 | 7.141497762 | 6.85E-07 | 1.67E-05 | 6.107255526 |
| DDX3X | 2.31456759 | 10.69850266 | 7.138486079 | 6.90E-07 | 1.68E-05 | 6.10118356 |
| SMAD2 | 1.307908049 | 4.277299535 | 7.123868526 | 7.10E-07 | 1.72E-05 | 6.071694513 |
| TMCC2 | -2.606176562 | 9.160714391 | -7.123692271 | 7.11E-07 | 1.72E-05 | 6.071338759 |
| TMEM170B | 2.20320651 | 7.5897608 | 7.123369965 | 7.11E-07 | 1.72E-05 | 6.070688204 |
| C9orf173 | -3.669402006 | 10.51121251 | -7.120753574 | 7.15E-07 | 1.73E-05 | 6.065406629 |
| RANBP2 | 1.73926424 | 6.449408918 | 7.120084591 | 7.16E-07 | 1.73E-05 | 6.064056033 |
| LGR6 | -2.924855761 | 9.184448428 | -7.119144202 | 7.17E-07 | 1.73E-05 | 6.062157394 |
| AVIL | -1.678926437 | 7.438272795 | -7.118825488 | 7.18E-07 | 1.73E-05 | 6.061513885 |
| MTX3 | 2.100371742 | 4.463368441 | 7.117124452 | 7.20E-07 | 1.74E-05 | 6.058079116 |
| C17orf99 | -2.984090519 | 7.762097177 | -7.116123533 | 7.22E-07 | 1.74E-05 | 6.056057851 |
| MDM1 | 2.22778922 | 5.588507627 | 7.115397182 | 7.23E-07 | 1.74E-05 | 6.054590962 |
| CLPS | -2.643299781 | 7.203538176 | -7.114513508 | 7.24E-07 | 1.74E-05 | 6.052806255 |
| RTN2 | -1.587797006 | 7.824842465 | -7.114400369 | 7.24E-07 | 1.74E-05 | 6.052577747 |
| TYW3 | 2.573925412 | 5.019138061 | 7.11049259 | 7.30E-07 | 1.75E-05 | 6.044684051 |
| PTPN2 | 1.591612296 | 7.988549565 | 7.109647412 | 7.31E-07 | 1.75E-05 | 6.042976514 |
| TMEM123 | 1.329622241 | 12.33991066 | 7.107236368 | 7.35E-07 | 1.75E-05 | 6.038104864 |
| NAF1 | 2.156240909 | 4.330926292 | 7.104334682 | 7.39E-07 | 1.76E-05 | 6.032240769 |
| ZNF624 | 2.14947569 | 5.589101543 | 7.101957199 | 7.43E-07 | 1.77E-05 | 6.027435168 |
| ARL5B | 2.321294716 | 4.556522629 | 7.100291242 | 7.45E-07 | 1.77E-05 | 6.024067305 |
| NT5M | -2.324684479 | 11.23985292 | -7.098507654 | 7.48E-07 | 1.77E-05 | 6.02046121 |
| PRPF40A | 1.264991433 | 11.45781802 | 7.097930002 | 7.49E-07 | 1.77E-05 | 6.019293206 |
| DYRK2 | 1.766344814 | 9.638120941 | 7.094683266 | 7.54E-07 | 1.78E-05 | 6.012727481 |
| TMEM65 | 1.534751614 | 6.660344186 | 7.093999238 | 7.55E-07 | 1.78E-05 | 6.011344015 |
| CEP70 | 1.883576585 | 4.908821405 | 7.083583008 | 7.71E-07 | 1.82E-05 | 5.990268812 |
| PRR5 | -2.270174912 | 10.73552585 | -7.082198879 | 7.73E-07 | 1.82E-05 | 5.987467157 |
| MYEF2 | 2.348887986 | 4.173146572 | 7.081264221 | 7.75E-07 | 1.82E-05 | 5.985575137 |
| ZNF738 | 2.75377478 | 8.981170771 | 7.076070399 | 7.83E-07 | 1.84E-05 | 5.975059105 |
| E2F2 | -2.078487572 | 12.49304769 | -7.075310046 | 7.84E-07 | 1.84E-05 | 5.973519288 |
| BRD1 | 1.113388227 | 10.44701251 | 7.07496293 | 7.85E-07 | 1.84E-05 | 5.972816304 |
| DNAJB9 | 1.37838116 | 9.434097918 | 7.073846128 | 7.87E-07 | 1.84E-05 | 5.97055443 |
| RAB39B | 1.533573918 | 7.775758944 | 7.070303762 | 7.92E-07 | 1.85E-05 | 5.96337887 |
| PSMG1 | 1.408884432 | 9.277244238 | 7.069696868 | 7.93E-07 | 1.85E-05 | 5.962149344 |
| MTMR6 | 1.147277875 | 7.719173291 | 7.068470784 | 7.95E-07 | 1.85E-05 | 5.959665227 |
| KIAA1468 | 1.540739658 | 8.786385491 | 7.067676556 | 7.97E-07 | 1.86E-05 | 5.958055965 |
| ATL2 | 1.174321884 | 7.890013863 | 7.066773641 | 7.98E-07 | 1.86E-05 | 5.956226372 |
| CGRRF1 | 1.317127315 | 6.781321859 | 7.06472864 | 8.01E-07 | 1.86E-05 | 5.952082129 |
| MCAT | -1.595854419 | 9.229121627 | -7.061162615 | 8.07E-07 | 1.87E-05 | 5.9448541 |
| JMY | 1.65225382 | 7.196140199 | 7.060685793 | 8.08E-07 | 1.87E-05 | 5.943887487 |
| METTL4 | 1.957172748 | 6.622128827 | 7.06008902 | 8.09E-07 | 1.87E-05 | 5.942677668 |
| MBNL2 | 2.019058372 | 7.09487815 | 7.057618622 | 8.13E-07 | 1.88E-05 | 5.93766897 |
| ABCA5 | 2.352171208 | 7.829691302 | 7.056375988 | 8.15E-07 | 1.88E-05 | 5.935149224 |
| ZNF429 | 2.61142691 | 11.05442819 | 7.054500875 | 8.18E-07 | 1.89E-05 | 5.931346565 |
| GGT5 | -1.865550656 | 6.628480771 | -7.052952438 | 8.21E-07 | 1.89E-05 | 5.928206023 |
| THAP1 | 1.506324087 | 8.315713324 | 7.049599141 | 8.27E-07 | 1.90E-05 | 5.921403714 |
| SMR3A | -4.308328315 | 8.850534489 | -7.048030608 | 8.29E-07 | 1.90E-05 | 5.918221339 |
| NAA30 | 0.86276632 | 7.321598229 | 7.046950672 | 8.31E-07 | 1.91E-05 | 5.916030071 |
| ZNF117 | 1.877865756 | 7.144862883 | 7.045690934 | 8.33E-07 | 1.91E-05 | 5.913473768 |
| CALML4 | -2.015842778 | 8.936466016 | -7.041106324 | 8.41E-07 | 1.92E-05 | 5.90416865 |
| RNF111 | 1.100384075 | 9.068239797 | 7.040356995 | 8.42E-07 | 1.92E-05 | 5.902647501 |
| ZNF532 | 1.387844092 | 5.687125308 | 7.038018621 | 8.46E-07 | 1.93E-05 | 5.897900067 |
| ARL6IP6 | 1.26039476 | 8.726533131 | 7.037994394 | 8.46E-07 | 1.93E-05 | 5.897850875 |
| KEL | -2.642221814 | 5.325912388 | -7.036894763 | 8.48E-07 | 1.93E-05 | 5.895618105 |
| GP6 | -2.006568303 | 7.212886833 | -7.034034595 | 8.53E-07 | 1.94E-05 | 5.889809825 |
| ZNF382 | 1.919784681 | 4.939535318 | 7.033375155 | 8.54E-07 | 1.94E-05 | 5.888470508 |
| EIF4E | 1.666575187 | 7.261812213 | 7.028194961 | 8.63E-07 | 1.96E-05 | 5.87794746 |
| GPR146 | -2.271904652 | 8.625640221 | -7.027140214 | 8.65E-07 | 1.96E-05 | 5.875804387 |
| DTWD2 | 2.241645741 | 4.279325175 | 7.025831382 | 8.68E-07 | 1.96E-05 | 5.873144843 |
| SRPK3 | -2.377962672 | 6.317938395 | -7.023337337 | 8.72E-07 | 1.97E-05 | 5.868076285 |
| USP38 | 1.182023962 | 9.097887734 | 7.023083562 | 8.73E-07 | 1.97E-05 | 5.867560499 |
| NIPA1 | 1.83433511 | 7.981275033 | 7.020168659 | 8.78E-07 | 1.98E-05 | 5.861635445 |
| SYNJ1 | 1.597467863 | 6.370336592 | 7.01845343 | 8.81E-07 | 1.98E-05 | 5.858148387 |
| MAP1LC3A | -3.175159738 | 8.583780508 | -7.018153118 | 8.81E-07 | 1.98E-05 | 5.857537812 |
| ORMDL1 | 1.405436217 | 11.60920645 | 7.01193824 | 8.93E-07 | 2.00E-05 | 5.844899284 |
| HIGD2B | -5.29581477 | 7.645777689 | -7.008076449 | 9.00E-07 | 2.02E-05 | 5.837043269 |
| ZNF727 | 2.544428548 | 12.30578672 | 7.007064418 | 9.02E-07 | 2.02E-05 | 5.834984158 |
| GOPC | 1.759218676 | 9.249495109 | 7.005757161 | 9.04E-07 | 2.02E-05 | 5.832324158 |
| SLC17A5 | 1.046988288 | 8.07251256 | 7.005373325 | 9.05E-07 | 2.02E-05 | 5.831543086 |
| PDLIM7 | -1.47286792 | 10.73582439 | -7.005285778 | 9.05E-07 | 2.02E-05 | 5.831364934 |
| DOCK10 | 1.393181265 | 10.48762018 | 7.003502096 | 9.08E-07 | 2.02E-05 | 5.827735008 |
| MAGT1 | 1.401312695 | 8.905089893 | 7.000153298 | 9.15E-07 | 2.03E-05 | 5.82091876 |
| ABCC3 | -1.718772614 | 8.319477309 | -6.998118533 | 9.18E-07 | 2.04E-05 | 5.816776374 |
| TTC25 | -2.642933793 | 7.67915771 | -6.996107022 | 9.22E-07 | 2.04E-05 | 5.812680762 |
| KIF26B | -2.943381525 | 7.320215124 | -6.993926117 | 9.26E-07 | 2.05E-05 | 5.808239613 |
| KATNB1 | -1.284891152 | 7.729723365 | -6.993695163 | 9.27E-07 | 2.05E-05 | 5.807769266 |
| PRKAA1 | 1.784505884 | 6.699559002 | 6.990003335 | 9.34E-07 | 2.06E-05 | 5.800249682 |
| PTPN11 | 1.259964938 | 9.082057165 | 6.98373836 | 9.46E-07 | 2.09E-05 | 5.787484729 |
| ZDHHC11B | -1.910898571 | 4.108437057 | -6.981082892 | 9.51E-07 | 2.09E-05 | 5.782072539 |
| VKORC1L1 | 1.190105888 | 6.530019654 | 6.980830114 | 9.51E-07 | 2.09E-05 | 5.781557294 |
| PRR3 | 1.386828374 | 5.575121562 | 6.978945938 | 9.55E-07 | 2.10E-05 | 5.777716438 |
| TAF1D | 1.541409067 | 9.197480084 | 6.976382629 | 9.60E-07 | 2.11E-05 | 5.772490391 |
| VCPIP1 | 0.960243448 | 9.485806139 | 6.974694024 | 9.64E-07 | 2.11E-05 | 5.769047184 |
| APOOL | 1.773386732 | 5.896790672 | 6.974588849 | 9.64E-07 | 2.11E-05 | 5.76883271 |
| FAM126B | 1.790265393 | 9.540579178 | 6.969141057 | 9.75E-07 | 2.13E-05 | 5.757721424 |
| ISOC1 | 1.26258097 | 9.213271415 | 6.964696917 | 9.84E-07 | 2.15E-05 | 5.74865413 |
| CABIN1 | -0.949820647 | 10.30023773 | -6.962614792 | 9.88E-07 | 2.16E-05 | 5.744405068 |
| PARVB | -1.091108518 | 7.332036905 | -6.957978251 | 9.97E-07 | 2.17E-05 | 5.734940961 |
| SLC6A9 | -2.62623173 | 7.325637466 | -6.957182357 | 9.99E-07 | 2.17E-05 | 5.733316084 |
| TMEM135 | 1.942413905 | 5.12725758 | 6.955415574 | 1.00E-06 | 2.18E-05 | 5.729708747 |
| RGPD5 | 1.277040369 | 9.599794185 | 6.953628608 | 1.01E-06 | 2.18E-05 | 5.726059763 |
| NRGN | -1.833637003 | 13.06013889 | -6.95246582 | 1.01E-06 | 2.19E-05 | 5.723685111 |
| CSN1S1 | -3.002285129 | 6.437479411 | -6.950930207 | 1.01E-06 | 2.19E-05 | 5.720548788 |
| HAUS6 | 1.647559415 | 6.49265017 | 6.949356339 | 1.02E-06 | 2.20E-05 | 5.717333994 |
| MTR | 1.720234167 | 6.846074239 | 6.948151169 | 1.02E-06 | 2.20E-05 | 5.714872074 |
| ZNF138 | 1.57346611 | 6.675253462 | 6.946198789 | 1.02E-06 | 2.20E-05 | 5.710883324 |
| CHUK | 1.120758261 | 10.19256799 | 6.944103362 | 1.03E-06 | 2.21E-05 | 5.706601741 |
| SCAMP1 | 0.8842829 | 6.902554289 | 6.944052625 | 1.03E-06 | 2.21E-05 | 5.706498063 |
| CCAR1 | 1.877042295 | 8.40282744 | 6.939185866 | 1.04E-06 | 2.23E-05 | 5.696551438 |
| PTP4A3 | -1.518384903 | 8.746084137 | -6.937956361 | 1.04E-06 | 2.23E-05 | 5.694038071 |
| SRFBP1 | 1.415740427 | 7.060792822 | 6.93414336 | 1.05E-06 | 2.24E-05 | 5.686242163 |
| SLC35F5 | 1.406342188 | 7.153296756 | 6.933664935 | 1.05E-06 | 2.24E-05 | 5.685263852 |
| BCL2L1 | -2.543776979 | 9.458300351 | -6.933152598 | 1.05E-06 | 2.24E-05 | 5.684216161 |
| THEG | -2.484483436 | 5.445879909 | -6.933023295 | 1.05E-06 | 2.24E-05 | 5.683951741 |
| ZZZ3 | 1.53429958 | 7.549100523 | 6.932278216 | 1.05E-06 | 2.24E-05 | 5.682428032 |
| ATF2 | 1.78350946 | 6.681987655 | 6.928435404 | 1.06E-06 | 2.26E-05 | 5.674568146 |
| UBXN2A | 1.797546004 | 5.513381689 | 6.925486109 | 1.07E-06 | 2.27E-05 | 5.668534427 |
| TNS1 | -2.471170646 | 10.38122325 | -6.923726029 | 1.07E-06 | 2.28E-05 | 5.664933052 |
| SLC35A1 | 1.46048004 | 7.937771692 | 6.921679046 | 1.07E-06 | 2.28E-05 | 5.660744093 |
| EXOC8 | 1.851338301 | 7.534915962 | 6.919526649 | 1.08E-06 | 2.29E-05 | 5.656338788 |
| NUS1 | 1.72129738 | 8.187250552 | 6.918319905 | 1.08E-06 | 2.29E-05 | 5.653868667 |
| ZDHHC20 | 2.396123496 | 6.856903834 | 6.917074093 | 1.08E-06 | 2.29E-05 | 5.651318368 |
| FAR1 | 2.429645275 | 7.71058193 | 6.916824851 | 1.09E-06 | 2.29E-05 | 5.650808118 |
| C10orf88 | 1.846835608 | 7.463756579 | 6.913125495 | 1.09E-06 | 2.31E-05 | 5.64323378 |
| CEP44 | 2.149288082 | 5.661050209 | 6.910655854 | 1.10E-06 | 2.32E-05 | 5.638176198 |
| CHIC1 | 1.688782668 | 5.607397639 | 6.910572199 | 1.10E-06 | 2.32E-05 | 5.638004867 |
| PRPS2 | 2.06983343 | 4.660245903 | 6.909086961 | 1.10E-06 | 2.32E-05 | 5.634962822 |
| SLC12A2 | 1.459324917 | 5.576289685 | 6.90854694 | 1.10E-06 | 2.32E-05 | 5.633856684 |
| MCOLN1 | -1.523569163 | 8.064008174 | -6.904286318 | 1.11E-06 | 2.33E-05 | 5.625128128 |
| PRPF39 | 2.728844538 | 5.748905463 | 6.904063402 | 1.11E-06 | 2.33E-05 | 5.624671379 |
| ERGIC2 | 1.311255051 | 9.763309179 | 6.904057873 | 1.11E-06 | 2.33E-05 | 5.624660051 |
| EFNA1 | -2.69728336 | 7.597525421 | -6.901511826 | 1.12E-06 | 2.34E-05 | 5.619442794 |
| UBE2W | 1.451276863 | 8.747516613 | 6.901040764 | 1.12E-06 | 2.34E-05 | 5.618477414 |
| TMEM30A | 1.314034132 | 10.11503937 | 6.897750911 | 1.13E-06 | 2.35E-05 | 5.611734443 |
| CIZ1 | -0.856754664 | 9.656473143 | -6.896814589 | 1.13E-06 | 2.36E-05 | 5.609815057 |
| NARS | 1.224503869 | 10.25792036 | 6.896069691 | 1.13E-06 | 2.36E-05 | 5.60828799 |
| ROCK1 | 2.506440438 | 8.637908105 | 6.894400703 | 1.14E-06 | 2.36E-05 | 5.604866228 |
| SGIP1 | -1.551481464 | 3.402931028 | -6.894356179 | 1.14E-06 | 2.36E-05 | 5.604774941 |
| SPAG8 | -1.627566575 | 7.09264118 | -6.893579399 | 1.14E-06 | 2.36E-05 | 5.603182249 |
| TMEM168 | 1.602908308 | 7.119023821 | 6.89277277 | 1.14E-06 | 2.36E-05 | 5.601528267 |
| HPS1 | -1.385313986 | 10.31666549 | -6.89173172 | 1.14E-06 | 2.36E-05 | 5.599393475 |
| TMBIM4 | 0.898595444 | 11.85382471 | 6.891441209 | 1.14E-06 | 2.36E-05 | 5.598797722 |
| CAV1 | -3.023573268 | 6.728873886 | -6.886774522 | 1.15E-06 | 2.38E-05 | 5.589226127 |
| ZNF197 | 2.01282745 | 5.253569136 | 6.884630414 | 1.16E-06 | 2.39E-05 | 5.584827453 |
| EMB | 1.480568166 | 11.29842586 | 6.88383219 | 1.16E-06 | 2.39E-05 | 5.58318972 |
| EPM2AIP1 | 1.348972529 | 6.757407285 | 6.883808674 | 1.16E-06 | 2.39E-05 | 5.583141471 |
| MCF2 | 1.708368466 | 3.348608585 | 6.883649354 | 1.16E-06 | 2.39E-05 | 5.582814581 |
| IBA57 | -1.464483743 | 8.132814605 | -6.883037709 | 1.16E-06 | 2.39E-05 | 5.581559582 |
| C8orf44 | 0.78354216 | 7.978294616 | 6.878914888 | 1.17E-06 | 2.40E-05 | 5.573098858 |
| C9orf85 | 1.945188103 | 6.402953662 | 6.878658577 | 1.17E-06 | 2.40E-05 | 5.572572786 |
| PDE12 | 1.468632173 | 8.299214212 | 6.878566203 | 1.17E-06 | 2.40E-05 | 5.57238319 |
| ZNF669 | 2.060201028 | 4.061138681 | 6.87759785 | 1.18E-06 | 2.40E-05 | 5.570395579 |
| AQP1 | -1.710155323 | 7.828456879 | -6.877138234 | 1.18E-06 | 2.40E-05 | 5.569452141 |
| UBE2J1 | 1.049461207 | 9.553440435 | 6.870989188 | 1.19E-06 | 2.43E-05 | 5.556827401 |
| ACBD3 | 2.027816617 | 5.943714591 | 6.868236369 | 1.20E-06 | 2.44E-05 | 5.551173841 |
| TM4SF5 | -2.391259233 | 5.000400102 | -6.867894548 | 1.20E-06 | 2.44E-05 | 5.550471758 |
| TBC1D23 | 1.476710587 | 9.191874064 | 6.867394689 | 1.20E-06 | 2.44E-05 | 5.549445045 |
| KIAA1644 | -2.081008078 | 3.335172645 | -6.86544703 | 1.21E-06 | 2.45E-05 | 5.545444214 |
| LTN1 | 1.982532229 | 6.438474123 | 6.864582833 | 1.21E-06 | 2.45E-05 | 5.543668837 |
| ZNF181 | 3.018861898 | 5.140687233 | 6.864149194 | 1.21E-06 | 2.45E-05 | 5.542777944 |
| IFT172 | -0.93004477 | 9.981327601 | -6.864089177 | 1.21E-06 | 2.45E-05 | 5.54265464 |
| SFPQ | 0.891391966 | 11.75159918 | 6.859928948 | 1.22E-06 | 2.46E-05 | 5.534106275 |
| MITF | -0.936001087 | 6.770543058 | -6.858822531 | 1.22E-06 | 2.46E-05 | 5.531832429 |
| SLC35A3 | 1.787234044 | 4.590037466 | 6.858722366 | 1.22E-06 | 2.46E-05 | 5.531626568 |
| FHL2 | -2.118306855 | 8.175819527 | -6.858478764 | 1.22E-06 | 2.46E-05 | 5.531125905 |
| CTNNB1 | 1.161612411 | 9.99525342 | 6.85786877 | 1.23E-06 | 2.46E-05 | 5.529872179 |
| ZNF675 | 2.600745658 | 9.524300965 | 6.857566189 | 1.23E-06 | 2.46E-05 | 5.529250262 |
| SKIL | 2.783809778 | 5.18663519 | 6.855414342 | 1.23E-06 | 2.47E-05 | 5.524827052 |
| FGF6 | -0.897114791 | 3.462165687 | -6.85188763 | 1.24E-06 | 2.49E-05 | 5.517576371 |
| LNPEP | 1.215455808 | 10.38275613 | 6.851049752 | 1.24E-06 | 2.49E-05 | 5.5158535 |
| TRIM10 | -2.080095381 | 8.772044881 | -6.849732582 | 1.25E-06 | 2.49E-05 | 5.513144898 |
| SOAT1 | 1.878509435 | 6.931808351 | 6.849681135 | 1.25E-06 | 2.49E-05 | 5.513039099 |
| SCOC | 2.74816245 | 5.044900813 | 6.849304004 | 1.25E-06 | 2.49E-05 | 5.512263527 |
| OLFML2A | -2.106939567 | 6.355832574 | -6.848898606 | 1.25E-06 | 2.49E-05 | 5.511429802 |
| PHF6 | 2.099909079 | 7.320752422 | 6.848533666 | 1.25E-06 | 2.49E-05 | 5.510679263 |
| CDC42EP2 | -1.739692252 | 10.68025083 | -6.845213193 | 1.26E-06 | 2.50E-05 | 5.503849501 |
| POLR3G | 1.526600473 | 4.37106433 | 6.844852311 | 1.26E-06 | 2.50E-05 | 5.503107126 |
| NPC1L1 | -3.405709728 | 8.544141129 | -6.844060035 | 1.26E-06 | 2.50E-05 | 5.501477259 |
| PRSS42 | -1.803452673 | 3.623415611 | -6.842227244 | 1.27E-06 | 2.50E-05 | 5.497706517 |
| SGMS1 | 2.201742148 | 6.991403715 | 6.841766475 | 1.27E-06 | 2.50E-05 | 5.496758468 |
| FAM110A | -0.864862421 | 13.61784001 | -6.841690109 | 1.27E-06 | 2.50E-05 | 5.496601341 |
| 1-Mar | 1.895140625 | 6.934864742 | 6.835290273 | 1.28E-06 | 2.54E-05 | 5.483430376 |
| WAC | 0.91533974 | 11.92747472 | 6.833206925 | 1.29E-06 | 2.54E-05 | 5.479141597 |
| RBM39 | 1.351015353 | 11.71649604 | 6.832779719 | 1.29E-06 | 2.54E-05 | 5.478262079 |
| DDHD1 | 2.043256665 | 6.714747431 | 6.829638389 | 1.30E-06 | 2.56E-05 | 5.471794029 |
| GORAB | 2.168474884 | 5.018547512 | 6.828449652 | 1.30E-06 | 2.56E-05 | 5.469346048 |
| CXorf57 | 2.257126042 | 6.306495912 | 6.828384141 | 1.30E-06 | 2.56E-05 | 5.469211135 |
| CEP57L1 | 1.696581712 | 5.478129592 | 6.826491638 | 1.31E-06 | 2.56E-05 | 5.46531346 |
| UBXN6 | -2.5395704 | 11.59409959 | -6.826223184 | 1.31E-06 | 2.56E-05 | 5.46476053 |
| AMD1 | 1.02977747 | 10.68526442 | 6.826007006 | 1.31E-06 | 2.56E-05 | 5.464315266 |
| ING3 | 0.765883323 | 9.612487558 | 6.825554895 | 1.31E-06 | 2.56E-05 | 5.463384024 |
| LMBR1 | 1.9987176 | 6.127200652 | 6.8247637 | 1.31E-06 | 2.56E-05 | 5.461754286 |
| VAV3 | 1.023319252 | 10.70271478 | 6.822464123 | 1.32E-06 | 2.57E-05 | 5.457017022 |
| PPP2R5B | -1.659604185 | 9.361955022 | -6.819874383 | 1.33E-06 | 2.58E-05 | 5.451681137 |
| BUB3 | 1.007377264 | 10.09067488 | 6.819408134 | 1.33E-06 | 2.58E-05 | 5.450720383 |
| ZNF326 | 1.371448929 | 7.075211604 | 6.817370143 | 1.33E-06 | 2.59E-05 | 5.446520542 |
| CALML3 | -0.960873756 | 8.580776562 | -6.811274259 | 1.35E-06 | 2.62E-05 | 5.433954897 |
| SNRNP40 | 1.817247148 | 7.104294444 | 6.809943589 | 1.35E-06 | 2.62E-05 | 5.431211264 |
| IL7 | 2.254841932 | 4.533911206 | 6.809833362 | 1.35E-06 | 2.62E-05 | 5.430983984 |
| TMF1 | 2.661214316 | 5.907951502 | 6.808948668 | 1.36E-06 | 2.62E-05 | 5.429159736 |
| C9orf72 | 2.602932725 | 7.669618311 | 6.803101779 | 1.37E-06 | 2.65E-05 | 5.417100705 |
| DTX2 | -1.06870968 | 8.447665949 | -6.802383728 | 1.38E-06 | 2.65E-05 | 5.415619425 |
| INSIG2 | 1.875031578 | 7.23953728 | 6.801233251 | 1.38E-06 | 2.66E-05 | 5.413245935 |
| IREB2 | 1.304709845 | 9.711533744 | 6.801085977 | 1.38E-06 | 2.66E-05 | 5.412942088 |
| UBXN4 | 1.515879584 | 8.416942798 | 6.798034641 | 1.39E-06 | 2.67E-05 | 5.406646099 |
| MTRF1L | 1.879789493 | 6.442827483 | 6.797591741 | 1.39E-06 | 2.67E-05 | 5.405732134 |
| NETO2 | 1.406183401 | 6.452219245 | 6.797097549 | 1.39E-06 | 2.67E-05 | 5.404712289 |
| ZNF318 | 1.000538672 | 8.269808067 | 6.795986582 | 1.39E-06 | 2.67E-05 | 5.402419512 |
| GCC2 | 1.936608988 | 8.699592936 | 6.791997484 | 1.41E-06 | 2.69E-05 | 5.394185546 |
| ACTR3 | 1.02555851 | 12.87033387 | 6.791017043 | 1.41E-06 | 2.69E-05 | 5.392161467 |
| ACR | -2.248388437 | 6.131046864 | -6.790018193 | 1.41E-06 | 2.70E-05 | 5.390099249 |
| INPP5K | -1.084534349 | 12.32585827 | -6.789370569 | 1.41E-06 | 2.70E-05 | 5.388762095 |
| LGI3 | -2.409128268 | 7.693800891 | -6.78911204 | 1.41E-06 | 2.70E-05 | 5.388228294 |
| SLC34A1 | -3.282119609 | 6.697190318 | -6.786977207 | 1.42E-06 | 2.71E-05 | 5.38382001 |
| SMC2 | 1.741300726 | 5.769298347 | 6.785559728 | 1.42E-06 | 2.71E-05 | 5.380892671 |
| WDR3 | 1.427665643 | 6.265795429 | 6.78413955 | 1.43E-06 | 2.72E-05 | 5.377959481 |
| DHX36 | 1.304514464 | 10.36960765 | 6.7826311 | 1.43E-06 | 2.72E-05 | 5.374843674 |
| DSTYK | -0.698554469 | 9.294359311 | -6.779292761 | 1.44E-06 | 2.74E-05 | 5.367947003 |
| NDUFA5 | 2.794391205 | 8.353309122 | 6.778247237 | 1.45E-06 | 2.74E-05 | 5.365786743 |
| POLR3F | 1.13182883 | 6.228813963 | 6.777969323 | 1.45E-06 | 2.74E-05 | 5.365212492 |
| GYPB | -2.654980617 | 8.627593155 | -6.776773936 | 1.45E-06 | 2.74E-05 | 5.362742357 |
| ADD2 | -1.669943847 | 5.836179732 | -6.776226418 | 1.45E-06 | 2.74E-05 | 5.361610907 |
| ZEB1 | 1.111926315 | 8.070564816 | 6.774800312 | 1.46E-06 | 2.75E-05 | 5.358663653 |
| PHF20L1 | 0.946704865 | 9.865323045 | 6.774198713 | 1.46E-06 | 2.75E-05 | 5.35742028 |
| C7orf60 | 1.237249239 | 6.660004778 | 6.773638837 | 1.46E-06 | 2.75E-05 | 5.356263094 |
| SELENBP1 | -2.908336236 | 14.26121999 | -6.772171817 | 1.46E-06 | 2.75E-05 | 5.353230763 |
| SS18 | 1.516167109 | 7.152984121 | 6.771459177 | 1.47E-06 | 2.76E-05 | 5.35175763 |
| C3orf33 | 2.16821189 | 3.565245203 | 6.767191014 | 1.48E-06 | 2.78E-05 | 5.342933252 |
| CRELD1 | -0.740098161 | 9.235694905 | -6.763445518 | 1.49E-06 | 2.79E-05 | 5.335187434 |
| MTF2 | 0.982721498 | 9.886039655 | 6.763046808 | 1.49E-06 | 2.79E-05 | 5.334362774 |
| ZNF385A | -1.102108637 | 11.60953629 | -6.761021818 | 1.50E-06 | 2.80E-05 | 5.330174116 |
| G2E3 | 1.664854538 | 6.796119362 | 6.760594388 | 1.50E-06 | 2.80E-05 | 5.329289912 |
| BICD1 | 2.048592658 | 3.594550086 | 6.759958449 | 1.50E-06 | 2.80E-05 | 5.32797433 |
| CDK2 | 1.648510777 | 5.066481647 | 6.75888125 | 1.51E-06 | 2.80E-05 | 5.325745778 |
| PMAIP1 | 2.041932608 | 8.145367138 | 6.758655665 | 1.51E-06 | 2.80E-05 | 5.32527906 |
| SLC35A5 | 1.63981438 | 7.71922867 | 6.758317894 | 1.51E-06 | 2.80E-05 | 5.324580221 |
| HP1BP3 | 1.184665625 | 11.90536318 | 6.758242061 | 1.51E-06 | 2.80E-05 | 5.324423323 |
| SLC36A4 | 1.041090141 | 8.453445772 | 6.754685935 | 1.52E-06 | 2.82E-05 | 5.317064834 |
| CA6 | -1.655498655 | 6.4770211 | -6.75431453 | 1.52E-06 | 2.82E-05 | 5.316296207 |
| S100PBP | 1.120870956 | 9.680364187 | 6.753924809 | 1.52E-06 | 2.82E-05 | 5.315489656 |
| ZNF254 | 2.617429683 | 10.58430581 | 6.749928419 | 1.53E-06 | 2.84E-05 | 5.307217681 |
| THAP5 | 2.682585744 | 5.640223441 | 6.746806258 | 1.54E-06 | 2.85E-05 | 5.300753725 |
| ATR | 1.29646762 | 9.103635585 | 6.746480614 | 1.54E-06 | 2.85E-05 | 5.300079453 |
| BDP1 | 2.200229427 | 6.655212152 | 6.745048644 | 1.55E-06 | 2.86E-05 | 5.297114271 |
| MOB4 | 1.83413007 | 8.175775135 | 6.743003541 | 1.56E-06 | 2.87E-05 | 5.292878988 |
| CCNG2 | 1.702590951 | 6.391175808 | 6.739461741 | 1.57E-06 | 2.88E-05 | 5.285542793 |
| CTDSPL | -1.947438222 | 6.880475757 | -6.739302629 | 1.57E-06 | 2.88E-05 | 5.285213183 |
| LIN54 | 1.787532039 | 7.047688528 | 6.736988848 | 1.58E-06 | 2.90E-05 | 5.280419633 |
| ZFP30 | 1.521336305 | 6.846475188 | 6.734548432 | 1.58E-06 | 2.91E-05 | 5.275362939 |
| ZC3H14 | 1.050273215 | 8.849154717 | 6.733086934 | 1.59E-06 | 2.91E-05 | 5.272334237 |
| PTBP3 | 1.053365256 | 11.12401837 | 6.730942619 | 1.60E-06 | 2.92E-05 | 5.267889989 |
| C9 | -2.790095108 | 3.997121976 | -6.729809876 | 1.60E-06 | 2.93E-05 | 5.265542045 |
| ZBTB38 | 1.458558524 | 5.463731728 | 6.728288866 | 1.60E-06 | 2.93E-05 | 5.262389027 |
| ITGAV | 1.983979362 | 7.450994187 | 6.727460378 | 1.61E-06 | 2.93E-05 | 5.26067146 |
| ZNF253 | 2.741342453 | 9.622217183 | 6.725912944 | 1.61E-06 | 2.94E-05 | 5.25746317 |
| PKMYT1 | -1.033871777 | 8.74097804 | -6.723215233 | 1.62E-06 | 2.95E-05 | 5.251869234 |
| TMEM40 | -1.855579919 | 8.825239701 | -6.722840712 | 1.62E-06 | 2.95E-05 | 5.251092556 |
| FAM3C | 2.540279238 | 7.760918304 | 6.721938338 | 1.63E-06 | 2.95E-05 | 5.249221139 |
| PF4 | -1.703973586 | 14.67397091 | -6.721808582 | 1.63E-06 | 2.95E-05 | 5.248952032 |
| CCNG1 | 1.393336751 | 8.295103626 | 6.721741562 | 1.63E-06 | 2.95E-05 | 5.248813034 |
| KRT25 | -1.748850834 | 3.563868882 | -6.717831396 | 1.64E-06 | 2.97E-05 | 5.240702432 |
| XRN1 | 1.065110181 | 8.593878059 | 6.713529297 | 1.65E-06 | 3.00E-05 | 5.231776471 |
| TCEB2 | -0.893602231 | 9.750267002 | -6.713217862 | 1.66E-06 | 3.00E-05 | 5.23113021 |
| WNK2 | -1.951053798 | 4.034510406 | -6.709097129 | 1.67E-06 | 3.02E-05 | 5.222578016 |
| CCNC | 1.842019356 | 6.576409418 | 6.708229212 | 1.67E-06 | 3.02E-05 | 5.220776441 |
| CCDC90B | 1.325373319 | 9.559773122 | 6.707500772 | 1.68E-06 | 3.02E-05 | 5.219264306 |
| TOMM20 | 0.97760252 | 10.7546764 | 6.706020725 | 1.68E-06 | 3.03E-05 | 5.216191725 |
| ZNF85 | 2.650163237 | 8.714780613 | 6.70106738 | 1.70E-06 | 3.06E-05 | 5.205906406 |
| MYSM1 | 2.582553848 | 5.497809775 | 6.699067372 | 1.71E-06 | 3.07E-05 | 5.20175257 |
| PJA2 | 1.296199855 | 9.917797366 | 6.694149748 | 1.72E-06 | 3.09E-05 | 5.1915368 |
| AP1G1 | 0.77318514 | 9.951802221 | 6.693045726 | 1.73E-06 | 3.10E-05 | 5.189242877 |
| LMBRD2 | 1.962428001 | 4.108790658 | 6.691769523 | 1.73E-06 | 3.10E-05 | 5.186590993 |
| PLXNB3 | -2.424798022 | 3.483713825 | -6.690048208 | 1.74E-06 | 3.11E-05 | 5.18301384 |
| RANBP6 | 2.029524891 | 5.218939661 | 6.687730343 | 1.75E-06 | 3.12E-05 | 5.178196332 |
| DENND5B | 1.897007287 | 5.42567498 | 6.687042978 | 1.75E-06 | 3.13E-05 | 5.176767555 |
| MZT1 | 2.881522564 | 6.170369221 | 6.685300292 | 1.76E-06 | 3.13E-05 | 5.173144874 |
| UBE2V2 | 1.491713589 | 6.030505281 | 6.683409784 | 1.76E-06 | 3.14E-05 | 5.169214435 |
| ADO | 1.902338721 | 6.420228414 | 6.682451908 | 1.77E-06 | 3.15E-05 | 5.16722279 |
| OTOR | -3.075290183 | 5.204092589 | -6.681565491 | 1.77E-06 | 3.15E-05 | 5.165379613 |
| PVALB | -3.129896773 | 8.29514307 | -6.680751529 | 1.77E-06 | 3.15E-05 | 5.163687002 |
| HS2ST1 | 1.227883635 | 5.736852933 | 6.680071141 | 1.77E-06 | 3.15E-05 | 5.162272087 |
| SCRN3 | 1.718913849 | 5.868937189 | 6.674450262 | 1.80E-06 | 3.19E-05 | 5.15058067 |
| FCHO2 | 1.922346818 | 7.142475534 | 6.67368202 | 1.80E-06 | 3.19E-05 | 5.148982396 |
| DLEU7 | -3.008890805 | 5.941393989 | -6.673532853 | 1.80E-06 | 3.19E-05 | 5.148672057 |
| MFAP3 | 0.757041 | 7.316591538 | 6.671415453 | 1.81E-06 | 3.20E-05 | 5.144266499 |
| SCFD2 | -1.01773776 | 9.457527875 | -6.669001177 | 1.82E-06 | 3.21E-05 | 5.139242508 |
| C4orf32 | 1.489136684 | 7.000684991 | 6.668182126 | 1.82E-06 | 3.21E-05 | 5.137537924 |
| VPS54 | 1.493999876 | 7.840296265 | 6.668159562 | 1.82E-06 | 3.21E-05 | 5.137490964 |
| TIFA | 1.51722905 | 2.807925943 | 6.668153127 | 1.82E-06 | 3.21E-05 | 5.137477571 |
| WDTC1 | -1.20447785 | 8.336665858 | -6.667299425 | 1.82E-06 | 3.21E-05 | 5.135700772 |
| DNAJB4 | 2.524146943 | 4.353573655 | 6.666311753 | 1.83E-06 | 3.21E-05 | 5.133645022 |
| ZBTB1 | 2.320077846 | 7.088009893 | 6.664163861 | 1.83E-06 | 3.22E-05 | 5.12917392 |
| CEP120 | 1.664884488 | 7.175823114 | 6.664140416 | 1.83E-06 | 3.22E-05 | 5.129125113 |
| MAOB | -2.305002419 | 3.891766432 | -6.663782034 | 1.84E-06 | 3.22E-05 | 5.128379035 |
| VIL1 | -2.349414298 | 4.978056515 | -6.661192275 | 1.85E-06 | 3.23E-05 | 5.122987174 |
| GUCY1A3 | -1.351205965 | 6.826085096 | -6.658817325 | 1.86E-06 | 3.25E-05 | 5.118041748 |
| MYBL1 | 2.486084812 | 8.895906909 | 6.657761135 | 1.86E-06 | 3.25E-05 | 5.115842167 |
| NCKAP5L | -0.922971817 | 9.884200337 | -6.657637169 | 1.86E-06 | 3.25E-05 | 5.115583992 |
| BMPR2 | 2.261799202 | 4.508607989 | 6.656144989 | 1.87E-06 | 3.25E-05 | 5.112476155 |
| STXBP3 | 1.640566998 | 10.33203665 | 6.654629495 | 1.87E-06 | 3.26E-05 | 5.109319454 |
| PPP3CC | 0.96991393 | 10.89891282 | 6.654356492 | 1.87E-06 | 3.26E-05 | 5.108750768 |
| GALNT1 | 1.255273507 | 8.419683284 | 6.647869643 | 1.90E-06 | 3.30E-05 | 5.095235237 |
| GLO1 | 1.494785442 | 6.720454019 | 6.645912046 | 1.91E-06 | 3.31E-05 | 5.091155414 |
| TMEM30B | 2.244156425 | 4.58923404 | 6.644051142 | 1.91E-06 | 3.32E-05 | 5.08727663 |
| B3GALNT2 | 1.299996483 | 6.552293513 | 6.643973596 | 1.91E-06 | 3.32E-05 | 5.087114986 |
| ZNF688 | -0.799294072 | 11.5587267 | -6.643590611 | 1.92E-06 | 3.32E-05 | 5.086316648 |
| SNX25 | 1.51188058 | 6.204165412 | 6.642246431 | 1.92E-06 | 3.32E-05 | 5.083514527 |
| TACR2 | -2.281071128 | 7.611475928 | -6.639588337 | 1.93E-06 | 3.34E-05 | 5.077972663 |
| MRPL42 | 1.281781524 | 8.344698091 | 6.63378529 | 1.96E-06 | 3.37E-05 | 5.065870575 |
| GCNT4 | 1.710764869 | 3.750592197 | 6.633731436 | 1.96E-06 | 3.37E-05 | 5.065758242 |
| KIAA1109 | 1.518132137 | 9.092440353 | 6.633637939 | 1.96E-06 | 3.37E-05 | 5.065563219 |
| FLCN | -2.590064322 | 9.084995012 | -6.63314485 | 1.96E-06 | 3.37E-05 | 5.064534678 |
| ZNF25 | 1.374232294 | 6.923656446 | 6.632792467 | 1.96E-06 | 3.37E-05 | 5.063799617 |
| SPPL2A | 0.796866752 | 10.10119336 | 6.632350132 | 1.96E-06 | 3.37E-05 | 5.062876896 |
| UQCRFS1 | 0.64671106 | 12.71052444 | 6.630960524 | 1.97E-06 | 3.38E-05 | 5.059977967 |
| ATAD5 | 2.112215793 | 4.173024097 | 6.629264919 | 1.97E-06 | 3.39E-05 | 5.056440331 |
| TFE3 | -1.524186546 | 10.46646773 | -6.627400804 | 1.98E-06 | 3.40E-05 | 5.052550679 |
| PTP4A1 | 0.824556517 | 8.436345739 | 6.626162506 | 1.99E-06 | 3.40E-05 | 5.049966595 |
| RBM27 | 1.565816564 | 9.666010192 | 6.624133759 | 2.00E-06 | 3.41E-05 | 5.045732553 |
| ZGLP1 | -0.944746782 | 9.688081604 | -6.62035942 | 2.01E-06 | 3.44E-05 | 5.037853948 |
| GDI2 | 1.398722653 | 8.844801212 | 6.617483607 | 2.02E-06 | 3.45E-05 | 5.031849656 |
| ABLIM3 | -1.525851723 | 6.865481671 | -6.617222217 | 2.02E-06 | 3.45E-05 | 5.031303855 |
| PEX10 | -0.945879756 | 6.701313817 | -6.616160417 | 2.03E-06 | 3.46E-05 | 5.029086647 |
| HIPK3 | 2.860751853 | 6.472392432 | 6.615894818 | 2.03E-06 | 3.46E-05 | 5.02853201 |
| RAD18 | 1.412128144 | 5.232875156 | 6.613783094 | 2.04E-06 | 3.46E-05 | 5.024121871 |
| PPA2 | 1.40230978 | 10.68116338 | 6.613694668 | 2.04E-06 | 3.46E-05 | 5.023937186 |
| KIF5B | 1.46325552 | 9.26374459 | 6.612129473 | 2.05E-06 | 3.47E-05 | 5.020668018 |
| OTUD5 | -0.774555943 | 11.73846753 | -6.61194182 | 2.05E-06 | 3.47E-05 | 5.020276052 |
| BCLAF1 | 1.356870507 | 11.00182856 | 6.611421195 | 2.05E-06 | 3.47E-05 | 5.019188553 |
| TMX4 | 0.889931382 | 11.57073923 | 6.611187411 | 2.05E-06 | 3.47E-05 | 5.018700206 |
| SUZ12 | 0.928380855 | 10.84687663 | 6.60682053 | 2.07E-06 | 3.50E-05 | 5.00957696 |
| DR1 | 1.168781194 | 9.141967703 | 6.601663663 | 2.09E-06 | 3.53E-05 | 4.998799988 |
| ZNF626 | 2.207292836 | 9.079045014 | 6.600992418 | 2.09E-06 | 3.53E-05 | 4.997396939 |
| ERCC8 | 1.358431953 | 5.316882677 | 6.599383758 | 2.10E-06 | 3.54E-05 | 4.994034239 |
| RNF38 | 0.803362445 | 9.804621244 | 6.598868188 | 2.10E-06 | 3.54E-05 | 4.992956434 |
| WDR76 | 1.658152021 | 3.451781695 | 6.597704196 | 2.11E-06 | 3.55E-05 | 4.990522961 |
| VAMP4 | 1.561323775 | 6.826660621 | 6.594427662 | 2.12E-06 | 3.57E-05 | 4.983671976 |
| RBAK | 2.465785301 | 4.526166766 | 6.593692834 | 2.13E-06 | 3.57E-05 | 4.982135307 |
| PTCD2 | 1.217507283 | 2.87738725 | 6.592805178 | 2.13E-06 | 3.58E-05 | 4.98027895 |
| 15-Sep | 1.083790701 | 12.51606853 | 6.587867294 | 2.15E-06 | 3.61E-05 | 4.969950422 |
| USP15 | 1.88714869 | 11.57094184 | 6.586912969 | 2.16E-06 | 3.61E-05 | 4.967953891 |
| BRCA2 | 1.250556999 | 3.260565876 | 6.583784818 | 2.17E-06 | 3.63E-05 | 4.961408678 |
| TAB2 | 1.377954403 | 10.74463855 | 6.581211904 | 2.18E-06 | 3.65E-05 | 4.956024238 |
| HNRNPR | 1.226016272 | 10.41058492 | 6.581180345 | 2.18E-06 | 3.65E-05 | 4.955958187 |
| APC | 1.768593874 | 5.603990028 | 6.579163402 | 2.19E-06 | 3.66E-05 | 4.951736624 |
| ZNF567 | 1.632531462 | 6.879921906 | 6.578100739 | 2.20E-06 | 3.66E-05 | 4.949512198 |
| STRADB | -2.398220547 | 13.40666733 | -6.576376054 | 2.21E-06 | 3.67E-05 | 4.945901669 |
| EPT1 | 1.350567397 | 6.126191558 | 6.570488618 | 2.23E-06 | 3.72E-05 | 4.933573665 |
| MYNN | 1.450743964 | 6.828293958 | 6.566058474 | 2.25E-06 | 3.74E-05 | 4.92429411 |
| DPY19L1 | 2.17329205 | 4.606311934 | 6.56596822 | 2.25E-06 | 3.74E-05 | 4.924105034 |
| PCDHB1 | -1.802362063 | 4.059989728 | -6.564529909 | 2.26E-06 | 3.75E-05 | 4.92109171 |
| EML4 | 0.993798255 | 12.41260411 | 6.564124513 | 2.26E-06 | 3.75E-05 | 4.920242337 |
| MAP3K8 | 0.920315911 | 8.748135795 | 6.562667207 | 2.27E-06 | 3.76E-05 | 4.917188859 |
| RSRC2 | 1.111240394 | 11.14176725 | 6.560287909 | 2.28E-06 | 3.77E-05 | 4.91220293 |
| UHMK1 | 1.753700063 | 7.639096476 | 6.557448206 | 2.30E-06 | 3.79E-05 | 4.906251214 |
| HEATR5B | 1.70972868 | 6.345678017 | 6.555058912 | 2.31E-06 | 3.81E-05 | 4.901242674 |
| MAP2K3 | -1.811852074 | 13.65002291 | -6.550421218 | 2.33E-06 | 3.84E-05 | 4.891518775 |
| DCUN1D4 | 1.883834984 | 6.377070762 | 6.548738682 | 2.34E-06 | 3.85E-05 | 4.887990276 |
| ERLEC1 | 1.307578165 | 8.866047177 | 6.546466055 | 2.35E-06 | 3.87E-05 | 4.883223682 |
| HMGN5 | 1.835364604 | 3.801512363 | 6.545326711 | 2.36E-06 | 3.87E-05 | 4.880833769 |
| SMC5 | 2.341767272 | 3.842593171 | 6.543840572 | 2.36E-06 | 3.88E-05 | 4.877716153 |
| NHSL2 | -2.517066822 | 8.79748717 | -6.542548758 | 2.37E-06 | 3.89E-05 | 4.875005953 |
| PRPF4B | 1.437185014 | 8.842811551 | 6.542195229 | 2.37E-06 | 3.89E-05 | 4.874264217 |
| RAG1 | 1.541526711 | 3.090681128 | 6.538425125 | 2.39E-06 | 3.92E-05 | 4.866353166 |
| POLD4 | -0.950583234 | 14.83191399 | -6.536749833 | 2.40E-06 | 3.93E-05 | 4.862837188 |
| CNTNAP1 | -2.962959108 | 8.582460774 | -6.534838712 | 2.41E-06 | 3.94E-05 | 4.858825814 |
| HARBI1 | -0.78564307 | 7.990011393 | -6.533437858 | 2.41E-06 | 3.95E-05 | 4.855885164 |
| ZNF468 | 1.539124501 | 7.159659643 | 6.532195775 | 2.42E-06 | 3.95E-05 | 4.853277585 |
| MAPK6 | 1.947450092 | 6.70212415 | 6.531362687 | 2.43E-06 | 3.96E-05 | 4.851528518 |
| ZNF484 | 2.055139703 | 5.973889708 | 6.529583049 | 2.43E-06 | 3.97E-05 | 4.847791865 |
| THTPA | -1.070760628 | 8.722568662 | -6.527066863 | 2.45E-06 | 3.99E-05 | 4.842507985 |
| ERAL1 | -0.946990166 | 8.480632664 | -6.525256843 | 2.46E-06 | 4.00E-05 | 4.838706503 |
| ZNF208 | 2.582669245 | 3.458067957 | 6.523966286 | 2.46E-06 | 4.00E-05 | 4.835995755 |
| ZNF430 | 1.697209465 | 5.894539489 | 6.522494487 | 2.47E-06 | 4.01E-05 | 4.832904048 |
| RET | -2.192539141 | 6.581966024 | -6.519825384 | 2.49E-06 | 4.03E-05 | 4.827296512 |
| CCDC121 | 1.348613381 | 4.676356576 | 6.519812216 | 2.49E-06 | 4.03E-05 | 4.827268845 |
| ADAM21 | -3.41049581 | 5.917959198 | -6.519629057 | 2.49E-06 | 4.03E-05 | 4.82688401 |
| PER3 | 1.327771142 | 3.569769122 | 6.518254864 | 2.49E-06 | 4.03E-05 | 4.823996555 |
| AASDHPPT | 1.524599297 | 8.870433223 | 6.517506162 | 2.50E-06 | 4.04E-05 | 4.822423277 |
| NGLY1 | 0.964707523 | 12.26028586 | 6.515988963 | 2.51E-06 | 4.04E-05 | 4.819234896 |
| TXNRD1 | 2.299273025 | 5.913289142 | 6.515735746 | 2.51E-06 | 4.04E-05 | 4.818702733 |
| RECK | 1.870774392 | 6.66666025 | 6.512761073 | 2.52E-06 | 4.07E-05 | 4.8124505 |
| KRTAP5-10 | -1.716523963 | 7.730288061 | -6.510288319 | 2.54E-06 | 4.08E-05 | 4.807252317 |
| SPTB | -1.850170664 | 6.054871511 | -6.510012972 | 2.54E-06 | 4.08E-05 | 4.806673437 |
| CTSE | -1.700349804 | 6.449161781 | -6.50909083 | 2.54E-06 | 4.09E-05 | 4.804734686 |
| FGF7 | 1.70383932 | 3.831221522 | 6.508626451 | 2.54E-06 | 4.09E-05 | 4.803758312 |
| PRSS33 | -2.102330006 | 6.557518364 | -6.504482406 | 2.57E-06 | 4.12E-05 | 4.795044046 |
| BCKDHA | -1.000225013 | 9.996403733 | -6.503291214 | 2.57E-06 | 4.12E-05 | 4.792538739 |
| ZNF443 | 1.552011648 | 4.797314657 | 6.503074571 | 2.57E-06 | 4.12E-05 | 4.792083075 |
| NKIRAS2 | -1.871874283 | 9.624021032 | -6.501173297 | 2.59E-06 | 4.13E-05 | 4.788083886 |
| ZRANB3 | 1.599490739 | 4.369587177 | 6.501017621 | 2.59E-06 | 4.13E-05 | 4.787756411 |
| ZBTB3 | -0.940703735 | 9.14714596 | -6.500136547 | 2.59E-06 | 4.14E-05 | 4.785902956 |
| HELZ | 0.848009704 | 10.10607704 | 6.498759945 | 2.60E-06 | 4.14E-05 | 4.783006888 |
| TAF7 | 1.465731969 | 11.8355938 | 6.498730443 | 2.60E-06 | 4.14E-05 | 4.78294482 |
| ZCCHC6 | 1.20928978 | 8.158962436 | 6.49802829 | 2.60E-06 | 4.15E-05 | 4.781467545 |
| RAP2C | 2.01209581 | 6.912651013 | 6.493936546 | 2.63E-06 | 4.18E-05 | 4.772857539 |
| NT5DC1 | 0.73601763 | 8.747995794 | 6.493241418 | 2.63E-06 | 4.18E-05 | 4.771394604 |
| ZNF217 | 1.548731988 | 9.496349254 | 6.492453005 | 2.63E-06 | 4.18E-05 | 4.769735268 |
| ADRB3 | -3.307975963 | 7.086704141 | -6.487994112 | 2.66E-06 | 4.22E-05 | 4.760349303 |
| CARM1 | -1.924411643 | 12.32463684 | -6.487687 | 2.66E-06 | 4.22E-05 | 4.759702736 |
| PGGT1B | 2.222182908 | 4.885112821 | 6.485414326 | 2.67E-06 | 4.23E-05 | 4.754917659 |
| PRRT1 | -2.017005312 | 6.850020613 | -6.485332844 | 2.67E-06 | 4.23E-05 | 4.754746087 |
| GK5 | 2.185663892 | 4.210092086 | 6.484547807 | 2.68E-06 | 4.23E-05 | 4.753093039 |
| JAK2 | 1.536464371 | 9.047342468 | 6.482476846 | 2.69E-06 | 4.25E-05 | 4.748731834 |
| ZNF99 | 2.318241779 | 6.246421254 | 6.481582065 | 2.69E-06 | 4.25E-05 | 4.746847352 |
| SLC9A6 | 1.020806422 | 9.454287622 | 6.481041098 | 2.70E-06 | 4.25E-05 | 4.745707982 |
| QPRT | -1.033222292 | 8.009214521 | -6.476369318 | 2.72E-06 | 4.29E-05 | 4.735866794 |
| MORF4L1 | 0.811420903 | 12.05587181 | 6.474024115 | 2.74E-06 | 4.31E-05 | 4.730925497 |
| MEF2B | -0.953089778 | 11.0353885 | -6.473291927 | 2.74E-06 | 4.31E-05 | 4.729382644 |
| LEF1 | 1.287498728 | 12.29650411 | 6.4732875 | 2.74E-06 | 4.31E-05 | 4.729373315 |
| ANK1 | -2.583277927 | 8.712026469 | -6.473169347 | 2.74E-06 | 4.31E-05 | 4.729124337 |
| MCM9 | 1.496162328 | 5.898941308 | 6.471452166 | 2.75E-06 | 4.32E-05 | 4.72550562 |
| HEXIM2 | -1.337949078 | 10.65362832 | -6.467391528 | 2.78E-06 | 4.35E-05 | 4.716946849 |
| TXNDC15 | 1.182779766 | 5.943665819 | 6.467067654 | 2.78E-06 | 4.35E-05 | 4.716264112 |
| ZNF714 | 2.339267482 | 10.9355161 | 6.465193451 | 2.79E-06 | 4.36E-05 | 4.712312967 |
| TAF13 | 3.865735863 | 4.957821618 | 6.463000411 | 2.80E-06 | 4.38E-05 | 4.707689071 |
| ZNF718 | 2.656388479 | 5.111768741 | 6.462956255 | 2.80E-06 | 4.38E-05 | 4.707595966 |
| BTG2 | 0.951196562 | 12.78444482 | 6.458359216 | 2.83E-06 | 4.42E-05 | 4.697901301 |
| DCAF17 | 0.959600826 | 8.27979102 | 6.457883398 | 2.83E-06 | 4.42E-05 | 4.696897694 |
| ZBTB26 | 1.656903544 | 3.788538119 | 6.456378236 | 2.84E-06 | 4.43E-05 | 4.693722773 |
| ZNF813 | 1.885110785 | 6.693948152 | 6.454106545 | 2.86E-06 | 4.44E-05 | 4.688930404 |
| ZFP1 | 1.952812961 | 5.988981406 | 6.454064569 | 2.86E-06 | 4.44E-05 | 4.688841844 |
| SIKE1 | 1.222927377 | 8.260512751 | 6.453378835 | 2.86E-06 | 4.44E-05 | 4.68739508 |
| NOLC1 | 1.73830423 | 8.37879059 | 6.453267897 | 2.86E-06 | 4.44E-05 | 4.687161015 |
| DTL | 1.652821258 | 4.489987426 | 6.45232658 | 2.87E-06 | 4.45E-05 | 4.685174907 |
| FBXO30 | 0.999732757 | 9.043726175 | 6.451436684 | 2.87E-06 | 4.45E-05 | 4.683297187 |
| SCN8A | 1.138783439 | 2.838277261 | 6.449034092 | 2.89E-06 | 4.47E-05 | 4.678227089 |
| ZNF283 | 2.03716837 | 6.54441836 | 6.445672303 | 2.91E-06 | 4.49E-05 | 4.671131564 |
| KRTAP12-3 | -2.902764822 | 5.144035076 | -6.445561526 | 2.91E-06 | 4.49E-05 | 4.670897729 |
| AGRN | -1.780911558 | 6.521832621 | -6.445492997 | 2.91E-06 | 4.49E-05 | 4.670753073 |
| TYW5 | 1.216629871 | 6.39522858 | 6.444929836 | 2.91E-06 | 4.49E-05 | 4.669564285 |
| THAP9 | 1.850941918 | 4.632064269 | 6.443696578 | 2.92E-06 | 4.50E-05 | 4.666960829 |
| METTL22 | -0.890362628 | 9.994683514 | -6.442882331 | 2.93E-06 | 4.50E-05 | 4.665241813 |
| SLC23A3 | -1.682717612 | 7.090522915 | -6.442090754 | 2.93E-06 | 4.51E-05 | 4.663570575 |
| ZBTB6 | 1.997722624 | 5.105858811 | 6.440250691 | 2.94E-06 | 4.52E-05 | 4.659685373 |
| CST11 | -1.276821413 | 3.870065035 | -6.440088664 | 2.94E-06 | 4.52E-05 | 4.659343242 |
| MAPK8 | 2.125084883 | 5.523585516 | 6.439728536 | 2.94E-06 | 4.52E-05 | 4.658582789 |
| RBM12B | 1.330093104 | 7.464385216 | 6.437811364 | 2.96E-06 | 4.53E-05 | 4.654534173 |
| SLC33A1 | 2.010441156 | 5.505726767 | 6.437637928 | 2.96E-06 | 4.53E-05 | 4.654167895 |
| VPS13A | 1.261733707 | 8.418014839 | 6.437540162 | 2.96E-06 | 4.53E-05 | 4.653961421 |
| NRIP1 | 0.839772093 | 8.656949339 | 6.435933142 | 2.97E-06 | 4.54E-05 | 4.650567344 |
| ZNF654 | 1.471352187 | 7.054262964 | 6.435733967 | 2.97E-06 | 4.54E-05 | 4.650146656 |
| SH3BP1 | -0.928786166 | 7.475125974 | -6.435227309 | 2.97E-06 | 4.54E-05 | 4.649076497 |
| ACSL5 | 0.886183883 | 11.37668185 | 6.433649985 | 2.98E-06 | 4.55E-05 | 4.645744665 |
| SLFN12L | 2.289967547 | 4.591425337 | 6.432810213 | 2.99E-06 | 4.55E-05 | 4.643970656 |
| TUBA8 | -1.805181725 | 10.61662231 | -6.43226781 | 2.99E-06 | 4.56E-05 | 4.642824787 |
| MAVS | -1.166668537 | 9.967266331 | -6.429648307 | 3.01E-06 | 4.58E-05 | 4.637290333 |
| PTPN22 | 2.055788094 | 8.1128568 | 6.428876076 | 3.01E-06 | 4.58E-05 | 4.635658604 |
| MED4 | 0.849460174 | 10.61882794 | 6.428035383 | 3.02E-06 | 4.58E-05 | 4.633882123 |
| MIS18BP1 | 1.92665526 | 7.283864563 | 6.427021491 | 3.03E-06 | 4.59E-05 | 4.63173953 |
| SLC44A1 | 1.043492088 | 9.464818992 | 6.424224261 | 3.04E-06 | 4.61E-05 | 4.625827629 |
| OFD1 | 1.046869969 | 10.07426842 | 6.423910533 | 3.05E-06 | 4.61E-05 | 4.625164505 |
| NYX | -3.519366611 | 10.61570263 | -6.423883929 | 3.05E-06 | 4.61E-05 | 4.625108272 |
| MED23 | 1.219970751 | 6.445077032 | 6.422607593 | 3.05E-06 | 4.62E-05 | 4.622410359 |
| PTCH1 | 1.884868551 | 4.856488035 | 6.418902184 | 3.08E-06 | 4.65E-05 | 4.614576678 |
| PQBP1 | -0.737518234 | 11.21010469 | -6.418505811 | 3.08E-06 | 4.65E-05 | 4.613738592 |
| NAA25 | 1.476574822 | 6.654199555 | 6.418430166 | 3.08E-06 | 4.65E-05 | 4.613578648 |
| ZNF92 | 3.094301742 | 7.549708192 | 6.417504407 | 3.09E-06 | 4.65E-05 | 4.611621144 |
| DNHD1 | -1.360579912 | 7.690109476 | -6.416224869 | 3.10E-06 | 4.66E-05 | 4.608915397 |
| TSPAN5 | -1.643363746 | 10.60446797 | -6.415800436 | 3.10E-06 | 4.66E-05 | 4.608017831 |
| ZNF441 | 2.023806725 | 6.432834551 | 6.414797582 | 3.11E-06 | 4.67E-05 | 4.605896963 |
| RNFT1 | 2.092204807 | 7.642563644 | 6.414562099 | 3.11E-06 | 4.67E-05 | 4.605398936 |
| CRYAA | -3.958991791 | 8.963868691 | -6.414124933 | 3.11E-06 | 4.67E-05 | 4.604474349 |
| DYNLT3 | 1.843764645 | 7.37170195 | 6.413676525 | 3.11E-06 | 4.67E-05 | 4.60352596 |
| NHLRC3 | 0.966598176 | 8.928520076 | 6.411388941 | 3.13E-06 | 4.68E-05 | 4.598687278 |
| SACM1L | 1.44553949 | 10.46157849 | 6.410966203 | 3.13E-06 | 4.68E-05 | 4.59779303 |
| RLN1 | 2.246856925 | 4.553390207 | 6.410788171 | 3.13E-06 | 4.68E-05 | 4.597416419 |
| OR5D13 | -2.540699813 | 4.398334458 | -6.410308293 | 3.13E-06 | 4.68E-05 | 4.596401261 |
| TSNAX | 2.125540116 | 7.599679587 | 6.407077307 | 3.16E-06 | 4.71E-05 | 4.589565481 |
| TNNC2 | -1.640080543 | 8.093200839 | -6.405585433 | 3.17E-06 | 4.72E-05 | 4.586408673 |
| ZNF729 | 2.234113065 | 7.362432674 | 6.405291063 | 3.17E-06 | 4.72E-05 | 4.585785753 |
| MED22 | -0.887026391 | 9.513793499 | -6.402689573 | 3.19E-06 | 4.74E-05 | 4.580280202 |
| PRKACB | 2.069209702 | 8.479801745 | 6.399955751 | 3.20E-06 | 4.77E-05 | 4.574493646 |
| LIN37 | -0.734030583 | 10.88732138 | -6.398611646 | 3.21E-06 | 4.78E-05 | 4.571648284 |
| CD200R1 | 1.885621895 | 5.671403367 | 6.398127605 | 3.22E-06 | 4.78E-05 | 4.570623549 |
| ZER1 | -1.51445014 | 13.6610441 | -6.397842723 | 3.22E-06 | 4.78E-05 | 4.57002043 |
| HNRNPD | 0.746667942 | 12.3363763 | 6.395902175 | 3.23E-06 | 4.79E-05 | 4.565911836 |
| TBC1D17 | -1.165205166 | 8.588200495 | -6.395769204 | 3.23E-06 | 4.79E-05 | 4.565630289 |
| TOR1AIP2 | 2.219322985 | 5.79498149 | 6.394847111 | 3.24E-06 | 4.80E-05 | 4.563677814 |
| CHORDC1 | 2.102318626 | 7.115870323 | 6.394319853 | 3.24E-06 | 4.80E-05 | 4.56256133 |
| C16orf59 | -1.707676553 | 7.215959166 | -6.393647374 | 3.25E-06 | 4.80E-05 | 4.561137281 |
| UBR3 | 2.47551183 | 4.65072671 | 6.393593238 | 3.25E-06 | 4.80E-05 | 4.561022641 |
| ZNF480 | 1.7272706 | 7.099427553 | 6.393207601 | 3.25E-06 | 4.80E-05 | 4.560205983 |
| WEE1 | 2.030135084 | 6.305120546 | 6.390886012 | 3.27E-06 | 4.81E-05 | 4.55528918 |
| ATP6V0C | -1.108308629 | 17.11820662 | -6.390651572 | 3.27E-06 | 4.81E-05 | 4.554792631 |
| FAM91A1 | 1.608950287 | 6.40412758 | 6.389633735 | 3.28E-06 | 4.82E-05 | 4.552636742 |
| CCDC34 | 1.415523632 | 4.63620735 | 6.389537767 | 3.28E-06 | 4.82E-05 | 4.552433464 |
| MORF4L2 | 0.988691757 | 8.46665944 | 6.389170295 | 3.28E-06 | 4.82E-05 | 4.551655082 |
| HMGN2 | 0.632440119 | 14.07285927 | 6.383723978 | 3.32E-06 | 4.87E-05 | 4.540116578 |
| AP2S1 | -0.848913068 | 15.49407894 | -6.382650824 | 3.33E-06 | 4.88E-05 | 4.537842552 |
| HIF1A | 1.393811053 | 7.741597845 | 6.381704937 | 3.33E-06 | 4.88E-05 | 4.535838083 |
| CXCR4 | 0.985341048 | 14.38768058 | 6.377518283 | 3.36E-06 | 4.92E-05 | 4.526964572 |
| RBM11 | 1.739606349 | 3.631176602 | 6.377200939 | 3.36E-06 | 4.92E-05 | 4.526291878 |
| CPXM1 | -2.938395381 | 5.793225577 | -6.37690866 | 3.37E-06 | 4.92E-05 | 4.525672303 |
| CHD6 | 1.171872866 | 6.962066006 | 6.375401216 | 3.38E-06 | 4.93E-05 | 4.522476637 |
| PIGN | 1.461012267 | 4.347549743 | 6.375057606 | 3.38E-06 | 4.93E-05 | 4.521748168 |
| HIC1 | -1.23321771 | 6.163414062 | -6.373885986 | 3.39E-06 | 4.94E-05 | 4.519264168 |
| ZNF614 | 1.415221212 | 6.545350227 | 6.37130819 | 3.41E-06 | 4.96E-05 | 4.51379825 |
| DAPP1 | 1.272882884 | 10.00898527 | 6.371149284 | 3.41E-06 | 4.96E-05 | 4.513461279 |
| DDN | -2.472503767 | 6.659608387 | -6.370764019 | 3.41E-06 | 4.96E-05 | 4.512644288 |
| EIF4A2 | 1.439537134 | 12.77585627 | 6.369937748 | 3.42E-06 | 4.97E-05 | 4.510892034 |
| TAPT1 | 0.945523544 | 7.414554494 | 6.368891586 | 3.42E-06 | 4.97E-05 | 4.508673337 |
| TES | 1.181347694 | 7.057857438 | 6.368394339 | 3.43E-06 | 4.97E-05 | 4.507618729 |
| SRSF6 | 1.173978937 | 11.15263999 | 6.366083444 | 3.44E-06 | 4.99E-05 | 4.502717138 |
| WBP2 | -1.390048553 | 11.21906935 | -6.364186068 | 3.46E-06 | 5.01E-05 | 4.498692139 |
| PNN | 1.803667859 | 5.289330648 | 6.362842077 | 3.47E-06 | 5.02E-05 | 4.495840781 |
| CCDC82 | 2.888418317 | 7.356051301 | 6.362510707 | 3.47E-06 | 5.02E-05 | 4.495137723 |
| UEVLD | 1.565858373 | 5.490495732 | 6.359746054 | 3.49E-06 | 5.05E-05 | 4.489271493 |
| LEPROTL1 | 1.182930823 | 10.80455782 | 6.3585627 | 3.50E-06 | 5.05E-05 | 4.486760267 |
| GMPR | -2.853119169 | 10.85473981 | -6.358381981 | 3.50E-06 | 5.05E-05 | 4.486376744 |
| PHC2 | -1.375288728 | 12.94276819 | -6.356316606 | 3.52E-06 | 5.07E-05 | 4.481993279 |
| BAG6 | -1.229191867 | 14.56918171 | -6.355702848 | 3.52E-06 | 5.07E-05 | 4.48069056 |
| CHMP2B | 1.064068648 | 7.460549656 | 6.353485121 | 3.54E-06 | 5.09E-05 | 4.475982961 |
| SHISA7 | -1.696227351 | 7.616643695 | -6.352345621 | 3.55E-06 | 5.10E-05 | 4.473563883 |
| ALDH3B1 | -1.470670906 | 8.136283863 | -6.351777288 | 3.55E-06 | 5.10E-05 | 4.47235729 |
| AP2A1 | -1.709480727 | 8.368867974 | -6.350085497 | 3.56E-06 | 5.12E-05 | 4.468765302 |
| PAPD4 | 1.311403102 | 9.100057896 | 6.349156128 | 3.57E-06 | 5.12E-05 | 4.466791921 |
| ASNA1 | -0.917550417 | 9.900671249 | -6.347244826 | 3.59E-06 | 5.14E-05 | 4.462733198 |
| PTGIR | -1.356579722 | 10.04006153 | -6.346302823 | 3.59E-06 | 5.15E-05 | 4.460732645 |
| TAS2R14 | 1.656431306 | 5.853442392 | 6.345484494 | 3.60E-06 | 5.15E-05 | 4.458994648 |
| ANKRD34B | 2.31786132 | 3.263629458 | 6.34467739 | 3.61E-06 | 5.15E-05 | 4.457280406 |
| STX5 | -0.861901095 | 11.32392499 | -6.344316089 | 3.61E-06 | 5.15E-05 | 4.456512999 |
| CGGBP1 | 1.652125877 | 8.778270606 | 6.342646579 | 3.62E-06 | 5.17E-05 | 4.452966713 |
| DYNLRB1 | -0.631379928 | 11.18024505 | -6.340385812 | 3.64E-06 | 5.19E-05 | 4.44816394 |
| INSIG1 | 1.053310956 | 9.717371435 | 6.339953217 | 3.64E-06 | 5.19E-05 | 4.44724486 |
| MAP4K3 | 1.481100808 | 2.759953762 | 6.338227576 | 3.66E-06 | 5.21E-05 | 4.443578368 |
| FAM98B | 1.650688467 | 3.723353262 | 6.337439406 | 3.66E-06 | 5.21E-05 | 4.441903606 |
| DPRX | -2.711221485 | 5.620755825 | -6.337203839 | 3.66E-06 | 5.21E-05 | 4.44140304 |
| HSPA14 | 1.338333533 | 9.880809721 | 6.33553126 | 3.68E-06 | 5.22E-05 | 4.437848705 |
| GSK3A | -0.805061243 | 13.57923243 | -6.333154572 | 3.70E-06 | 5.25E-05 | 4.432797472 |
| MINA | 1.345673537 | 5.704776629 | 6.332466207 | 3.70E-06 | 5.25E-05 | 4.431334339 |
| CCR4 | 2.640452803 | 5.479909938 | 6.330872698 | 3.71E-06 | 5.26E-05 | 4.427947067 |
| AGER | -0.767177988 | 9.752342108 | -6.329638078 | 3.72E-06 | 5.27E-05 | 4.425322451 |
| HIST1H2AG | 3.187065571 | 6.325488306 | 6.328931494 | 3.73E-06 | 5.28E-05 | 4.423820271 |
| UBTFL1 | -1.674295651 | 3.348272954 | -6.328472427 | 3.73E-06 | 5.28E-05 | 4.422844272 |
| FNTA | 0.94757869 | 11.95937371 | 6.327332661 | 3.74E-06 | 5.29E-05 | 4.420420957 |
| OR4K14 | 2.195559038 | 3.147824869 | 6.326547485 | 3.75E-06 | 5.29E-05 | 4.418751456 |
| SFRP2 | -2.257667625 | 5.701822691 | -6.326424803 | 3.75E-06 | 5.29E-05 | 4.418490593 |
| CXorf23 | 1.762605871 | 5.836609619 | 6.323549507 | 3.77E-06 | 5.32E-05 | 4.412376199 |
| BACH1 | 1.195438888 | 10.15528977 | 6.320718639 | 3.80E-06 | 5.34E-05 | 4.406355246 |
| BLZF1 | 1.362851153 | 6.864631076 | 6.320429826 | 3.80E-06 | 5.34E-05 | 4.405740913 |
| ESRP2 | -1.739027848 | 7.792695056 | -6.319511709 | 3.80E-06 | 5.35E-05 | 4.403787921 |
| KIF27 | 2.079425798 | 3.954266748 | 6.317562553 | 3.82E-06 | 5.37E-05 | 4.399641375 |
| ZBTB41 | 2.16113797 | 5.815586735 | 6.316927345 | 3.83E-06 | 5.37E-05 | 4.398289957 |
| CCDC9 | 1.172633382 | 12.81246747 | 6.316458769 | 3.83E-06 | 5.37E-05 | 4.397293019 |
| COL19A1 | 2.206811837 | 4.855670019 | 6.316338674 | 3.83E-06 | 5.37E-05 | 4.397037503 |
| KRIT1 | 1.144888172 | 2.591297431 | 6.313864397 | 3.85E-06 | 5.39E-05 | 4.391772746 |
| OR56B4 | -1.747845039 | 4.751840113 | -6.313425165 | 3.85E-06 | 5.39E-05 | 4.390838069 |
| RARA | -1.247873003 | 11.77016729 | -6.311990294 | 3.87E-06 | 5.41E-05 | 4.387784517 |
| XPA | 1.074122648 | 7.896040129 | 6.308378876 | 3.90E-06 | 5.44E-05 | 4.380097883 |
| DIS3 | 2.205786938 | 4.558220357 | 6.307040471 | 3.91E-06 | 5.45E-05 | 4.377248762 |
| SEPN1 | -0.910819492 | 8.210370941 | -6.306089564 | 3.92E-06 | 5.46E-05 | 4.375224386 |
| MAFF | -1.145536469 | 7.120552846 | -6.30478133 | 3.93E-06 | 5.47E-05 | 4.372439112 |
| EIF3E | 1.600354004 | 12.39565982 | 6.303719547 | 3.94E-06 | 5.48E-05 | 4.37017838 |
| SDSL | -1.777208058 | 9.897861868 | -6.302164968 | 3.95E-06 | 5.49E-05 | 4.366868133 |
| USP46 | 2.022844923 | 3.888248555 | 6.299276237 | 3.97E-06 | 5.52E-05 | 4.360716185 |
| TTC7B | -1.324171149 | 8.029149443 | -6.299117248 | 3.97E-06 | 5.52E-05 | 4.360377566 |
| BAG4 | 1.605974798 | 5.553058165 | 6.29885065 | 3.98E-06 | 5.52E-05 | 4.359809749 |
| C1orf198 | -1.305642105 | 10.15021491 | -6.297087398 | 3.99E-06 | 5.53E-05 | 4.356054042 |
| PDE5A | -1.55643984 | 8.706108263 | -6.29694143 | 3.99E-06 | 5.53E-05 | 4.355743113 |
| PCDH1 | -1.664868091 | 7.915037484 | -6.293275961 | 4.02E-06 | 5.57E-05 | 4.347934366 |
| KRR1 | 1.388690713 | 5.586267844 | 6.291565063 | 4.04E-06 | 5.59E-05 | 4.344288961 |
| OR10G9 | -2.246597365 | 5.150300027 | -6.288129636 | 4.07E-06 | 5.63E-05 | 4.336967976 |
| FAM188A | 1.46987957 | 7.605007075 | 6.287538907 | 4.07E-06 | 5.63E-05 | 4.335708966 |
| PLEKHA2 | 1.158701018 | 12.62001712 | 6.286596194 | 4.08E-06 | 5.64E-05 | 4.333699685 |
| KLHL35 | -0.958661535 | 5.994570732 | -6.285762938 | 4.09E-06 | 5.64E-05 | 4.331923606 |
| SSFA2 | 1.096979233 | 9.190083089 | 6.281064212 | 4.13E-06 | 5.69E-05 | 4.321906643 |
| C12orf4 | 1.360839769 | 8.104743756 | 6.278134945 | 4.16E-06 | 5.73E-05 | 4.315660474 |
| CARHSP1 | -0.960192894 | 10.9402217 | -6.277448949 | 4.16E-06 | 5.73E-05 | 4.314197545 |
| KLF3 | 1.392497228 | 10.84528345 | 6.276761311 | 4.17E-06 | 5.73E-05 | 4.312731052 |
| HEATR3 | 1.833498896 | 6.020129444 | 6.276429819 | 4.17E-06 | 5.73E-05 | 4.312024074 |
| NUP35 | 1.014812094 | 7.818111101 | 6.275747723 | 4.18E-06 | 5.74E-05 | 4.310569315 |
| OSBPL8 | 2.395688043 | 6.607632933 | 6.274028379 | 4.19E-06 | 5.75E-05 | 4.306902071 |
| SSR1 | 0.886658942 | 11.31771115 | 6.270727608 | 4.22E-06 | 5.79E-05 | 4.299860695 |
| BABAM1 | -1.106996277 | 12.11483861 | -6.27050273 | 4.23E-06 | 5.79E-05 | 4.299380923 |
| C6orf120 | 1.586200208 | 7.806105782 | 6.267457285 | 4.25E-06 | 5.82E-05 | 4.292882909 |
| NXF3 | -2.648853159 | 7.997290238 | -6.262232203 | 4.30E-06 | 5.88E-05 | 4.281731492 |
| SRRM3 | -2.404970902 | 6.226002045 | -6.26143924 | 4.31E-06 | 5.89E-05 | 4.280038841 |
| ECEL1 | 2.892780437 | 4.118818044 | 6.25809726 | 4.34E-06 | 5.92E-05 | 4.272904204 |
| PAEP | -1.688633162 | 6.42550929 | -6.257863364 | 4.34E-06 | 5.92E-05 | 4.272404818 |
| ASNSD1 | 1.345266421 | 10.88808643 | 6.257699685 | 4.34E-06 | 5.92E-05 | 4.272055345 |
| PELI1 | 1.219310711 | 11.23427417 | 6.256525533 | 4.35E-06 | 5.93E-05 | 4.269548309 |
| FSTL3 | -0.904985387 | 7.22303924 | -6.254624514 | 4.37E-06 | 5.95E-05 | 4.265488902 |
| FMO5 | 1.421261677 | 6.384415532 | 6.253424283 | 4.38E-06 | 5.96E-05 | 4.26292571 |
| NOBOX | -1.457548864 | 9.335149765 | -6.251209299 | 4.40E-06 | 5.99E-05 | 4.258194952 |
| ANP32E | 2.182259223 | 7.380045827 | 6.249334847 | 4.42E-06 | 6.01E-05 | 4.254191016 |
| BCAP29 | 0.836234273 | 7.863486853 | 6.248445534 | 4.43E-06 | 6.01E-05 | 4.252291238 |
| TMEM156 | 1.767974796 | 5.724417391 | 6.247748023 | 4.44E-06 | 6.02E-05 | 4.250801122 |
| GIN1 | 1.193122109 | 6.760745223 | 6.245039898 | 4.46E-06 | 6.04E-05 | 4.245015085 |
| NAE1 | 1.298058535 | 9.663134243 | 6.244879747 | 4.47E-06 | 6.04E-05 | 4.244672886 |
| GTF2H3 | 1.909058821 | 5.557707367 | 6.244369857 | 4.47E-06 | 6.05E-05 | 4.243583366 |
| ZNF84 | 1.797569731 | 7.316322492 | 6.243549494 | 4.48E-06 | 6.05E-05 | 4.241830369 |
| LPAR6 | 1.868900506 | 10.15882099 | 6.243419852 | 4.48E-06 | 6.05E-05 | 4.241553334 |
| MYO7B | -1.508007022 | 7.124574975 | -6.241667549 | 4.50E-06 | 6.06E-05 | 4.237808603 |
| ARSK | 1.436904692 | 4.951037104 | 6.241652684 | 4.50E-06 | 6.06E-05 | 4.237776835 |
| MS4A18 | -3.830179304 | 6.796991739 | -6.240834626 | 4.50E-06 | 6.07E-05 | 4.236028482 |
| HNRNPK | 1.319416722 | 10.5989531 | 6.240339894 | 4.51E-06 | 6.07E-05 | 4.2349711 |
| SNX14 | 1.27219546 | 10.59613034 | 6.23992116 | 4.51E-06 | 6.07E-05 | 4.234076124 |
| CDC42SE2 | 1.000723056 | 12.48545106 | 6.238935595 | 4.52E-06 | 6.08E-05 | 4.231969549 |
| BGLAP | -0.850574573 | 8.348661799 | -6.237699481 | 4.53E-06 | 6.09E-05 | 4.229327271 |
| THUMPD2 | 1.066140604 | 6.454910824 | 6.235794142 | 4.55E-06 | 6.11E-05 | 4.225254101 |
| ANKRD36 | 1.66958102 | 8.759302128 | 6.235228485 | 4.56E-06 | 6.11E-05 | 4.224044769 |
| C12orf60 | 1.320483779 | 4.84085483 | 6.234248205 | 4.57E-06 | 6.12E-05 | 4.221948909 |
| SET | 0.99137415 | 10.73198074 | 6.233067165 | 4.58E-06 | 6.13E-05 | 4.219423661 |
| COX7A2L | 0.883834783 | 10.38063812 | 6.230015666 | 4.61E-06 | 6.17E-05 | 4.212898263 |
| SCAF8 | 1.231972514 | 8.034780235 | 6.22933593 | 4.62E-06 | 6.17E-05 | 4.211444539 |
| BRMS1L | 2.095711123 | 4.288878801 | 6.228146711 | 4.63E-06 | 6.19E-05 | 4.208901066 |
| FOXF1 | -1.074061477 | 5.059371298 | -6.227183071 | 4.64E-06 | 6.19E-05 | 4.206839922 |
| C15orf39 | -1.150715948 | 12.42410017 | -6.225659175 | 4.65E-06 | 6.21E-05 | 4.203580203 |
| CCDC154 | -1.189835449 | 9.506246075 | -6.225078797 | 4.66E-06 | 6.21E-05 | 4.202338658 |
| ETNK2 | -1.413101701 | 6.379749916 | -6.22271325 | 4.68E-06 | 6.24E-05 | 4.197277836 |
| TMC5 | -2.194272258 | 5.385368634 | -6.222323013 | 4.69E-06 | 6.24E-05 | 4.1964429 |
| DCAF12L2 | -1.583054288 | 8.61285997 | -6.222080411 | 4.69E-06 | 6.24E-05 | 4.19592383 |
| ZNF583 | 2.453917701 | 4.717386025 | 6.221103396 | 4.70E-06 | 6.25E-05 | 4.193833332 |
| SNX16 | 2.218065639 | 4.841273506 | 6.218650858 | 4.72E-06 | 6.27E-05 | 4.188585163 |
| PLEKHA3 | 1.490560644 | 7.357125457 | 6.217678497 | 4.73E-06 | 6.28E-05 | 4.186504203 |
| PLEKHG5 | -1.421005338 | 9.724443196 | -6.217072982 | 4.74E-06 | 6.29E-05 | 4.185208274 |
| WDR89 | 2.648233623 | 5.289744208 | 6.216180891 | 4.75E-06 | 6.29E-05 | 4.183298931 |
| RSU1 | -1.267420278 | 8.771786507 | -6.21527054 | 4.76E-06 | 6.30E-05 | 4.181350402 |
| HOOK1 | 1.548945382 | 5.378255092 | 6.21424558 | 4.77E-06 | 6.31E-05 | 4.179156437 |
| TTC37 | 0.981464209 | 10.01069463 | 6.214002413 | 4.77E-06 | 6.31E-05 | 4.17863591 |
| ICAM4 | -1.65219126 | 10.23460603 | -6.213331244 | 4.78E-06 | 6.31E-05 | 4.177199156 |
| FZD6 | 2.062957423 | 3.511101564 | 6.212464832 | 4.79E-06 | 6.32E-05 | 4.175344366 |
| FAM107B | 1.017548063 | 11.60842005 | 6.211953407 | 4.79E-06 | 6.32E-05 | 4.174249477 |
| DNM1L | 1.000821843 | 7.537398699 | 6.209730423 | 4.82E-06 | 6.35E-05 | 4.169490004 |
| RPL36A-HNRNPH2 | 2.79857656 | 7.234144603 | 6.208474834 | 4.83E-06 | 6.36E-05 | 4.166801477 |
| KIAA1143 | 2.038633327 | 9.306161794 | 6.208078317 | 4.83E-06 | 6.36E-05 | 4.165952394 |
| ZNF638 | 2.122558996 | 7.106765932 | 6.207772734 | 4.84E-06 | 6.36E-05 | 4.16529802 |
| ACTR2 | 1.985926037 | 11.03154372 | 6.207061814 | 4.84E-06 | 6.37E-05 | 4.163775614 |
| ACSL3 | 1.571588187 | 7.222993696 | 6.205478882 | 4.86E-06 | 6.38E-05 | 4.160385602 |
| COL7A1 | -1.643849741 | 7.266997394 | -6.204771214 | 4.87E-06 | 6.39E-05 | 4.158869957 |
| ZFAND6 | 1.062808473 | 10.41945712 | 6.203702827 | 4.88E-06 | 6.39E-05 | 4.156581624 |
| PPP1R8 | 0.914051328 | 9.964888174 | 6.203655987 | 4.88E-06 | 6.39E-05 | 4.156481295 |
| SCYL2 | 0.989794645 | 8.758607252 | 6.201029079 | 4.91E-06 | 6.42E-05 | 4.150854208 |
| SP100 | 1.112755058 | 11.69553852 | 6.200896135 | 4.91E-06 | 6.42E-05 | 4.150569404 |
| PDIK1L | 1.633463793 | 6.407555697 | 6.199598526 | 4.92E-06 | 6.43E-05 | 4.147789463 |
| GPR65 | 1.412102827 | 10.20658517 | 6.199345638 | 4.92E-06 | 6.43E-05 | 4.14724766 |
| RDM1 | 1.197322812 | 5.006828316 | 6.197502199 | 4.94E-06 | 6.45E-05 | 4.143297933 |
| FIS1 | -1.338907496 | 12.38954258 | -6.197293348 | 4.95E-06 | 6.45E-05 | 4.142850426 |
| PCGF6 | 1.552229671 | 5.237595561 | 6.197268387 | 4.95E-06 | 6.45E-05 | 4.14279694 |
| ZFP36 | 0.725512925 | 13.8478335 | 6.195231851 | 4.97E-06 | 6.47E-05 | 4.13843293 |
| TMEM158 | -3.78474779 | 7.002336939 | -6.193750767 | 4.98E-06 | 6.49E-05 | 4.135258851 |
| RGPD6 | 2.682797471 | 6.056660822 | 6.193544476 | 4.99E-06 | 6.49E-05 | 4.134816731 |
| RNF141 | 1.584664303 | 9.750540905 | 6.190587219 | 5.02E-06 | 6.52E-05 | 4.128478199 |
| ZHX1 | 0.843628001 | 7.1293914 | 6.189703392 | 5.03E-06 | 6.53E-05 | 4.126583607 |
| SRSF3 | 1.176790506 | 11.99838239 | 6.187733965 | 5.05E-06 | 6.55E-05 | 4.122361546 |
| YTHDF3 | 1.049057276 | 10.68134332 | 6.187503247 | 5.05E-06 | 6.55E-05 | 4.121866902 |
| RASGRP1 | 1.684742071 | 11.05042936 | 6.186366859 | 5.06E-06 | 6.56E-05 | 4.119430459 |
| SLC39A8 | 1.591414481 | 8.156579427 | 6.186145967 | 5.07E-06 | 6.56E-05 | 4.118956841 |
| TMOD1 | -2.372395042 | 8.524363662 | -6.186136706 | 5.07E-06 | 6.56E-05 | 4.118936985 |
| RNASEH2A | -0.675259767 | 10.45880081 | -6.185290321 | 5.08E-06 | 6.56E-05 | 4.117122186 |
| UBAP1 | -1.54577694 | 10.28252162 | -6.184136624 | 5.09E-06 | 6.57E-05 | 4.114648311 |
| ESRRB | -1.862460945 | 5.837470673 | -6.183963755 | 5.09E-06 | 6.57E-05 | 4.114277614 |
| PNRC2 | 1.626444725 | 9.654170147 | 6.183211171 | 5.10E-06 | 6.58E-05 | 4.112663741 |
| ARL6IP5 | 1.170400478 | 11.2298467 | 6.180201359 | 5.13E-06 | 6.62E-05 | 4.106208668 |
| PURB | 0.876333163 | 10.29563742 | 6.176129638 | 5.18E-06 | 6.67E-05 | 4.097474343 |
| FYB | 1.144816655 | 12.20911364 | 6.175987961 | 5.18E-06 | 6.67E-05 | 4.097170393 |
| FAM172A | 1.104042818 | 9.135988274 | 6.1738775 | 5.20E-06 | 6.69E-05 | 4.092642353 |
| SLC6A4 | -1.268479101 | 6.012377386 | -6.173226305 | 5.21E-06 | 6.70E-05 | 4.091245088 |
| SUN1 | 1.269722298 | 7.751132484 | 6.171618056 | 5.23E-06 | 6.72E-05 | 4.087794052 |
| CD80 | -2.064349266 | 6.067210988 | -6.168879511 | 5.26E-06 | 6.75E-05 | 4.081916848 |
| ZNF432 | 2.116430841 | 5.297044802 | 6.166868144 | 5.28E-06 | 6.77E-05 | 4.077599647 |
| ARHGEF3 | 1.209225756 | 9.939769534 | 6.166713034 | 5.28E-06 | 6.77E-05 | 4.0772667 |
| B3GNT5 | 1.644171887 | 7.176349047 | 6.164716133 | 5.31E-06 | 6.79E-05 | 4.072980014 |
| TMCC3 | -1.577017806 | 10.46816359 | -6.164293111 | 5.31E-06 | 6.79E-05 | 4.072071861 |
| ZNF660 | 1.767074918 | 3.178044402 | 6.163762067 | 5.32E-06 | 6.79E-05 | 4.070931775 |
| CA13 | 1.212423458 | 5.395052851 | 6.163599033 | 5.32E-06 | 6.79E-05 | 4.070581752 |
| CLU | -2.486080897 | 10.02370867 | -6.16343302 | 5.32E-06 | 6.79E-05 | 4.070225333 |
| AAAS | -1.140915494 | 9.810911887 | -6.163415439 | 5.32E-06 | 6.79E-05 | 4.070187586 |
| FCF1 | 1.299103084 | 5.640920244 | 6.159271772 | 5.37E-06 | 6.85E-05 | 4.061290256 |
| TMEM59L | -2.180081073 | 7.50370459 | -6.158737622 | 5.38E-06 | 6.85E-05 | 4.060143167 |
| CAPRIN1 | 1.308211115 | 5.4270714 | 6.15845867 | 5.38E-06 | 6.85E-05 | 4.059544102 |
| AGPAT5 | 1.848731741 | 7.499445845 | 6.157344614 | 5.39E-06 | 6.86E-05 | 4.05715151 |
| USP16 | 1.675572229 | 9.516728484 | 6.156332196 | 5.40E-06 | 6.87E-05 | 4.054977069 |
| SCML1 | 2.277920557 | 4.069765699 | 6.156251337 | 5.40E-06 | 6.87E-05 | 4.054803396 |
| C19orf45 | -2.192332651 | 4.211377924 | -6.151827788 | 5.46E-06 | 6.93E-05 | 4.045301087 |
| CTAGE5 | 1.965359145 | 5.984419448 | 6.149678234 | 5.48E-06 | 6.96E-05 | 4.040682713 |
| GADD45G | -1.151799209 | 8.060101158 | -6.14916344 | 5.49E-06 | 6.96E-05 | 4.03957658 |
| FRS2 | 1.14274349 | 6.78815441 | 6.148656503 | 5.49E-06 | 6.96E-05 | 4.038487296 |
| RAB30 | 1.736648203 | 5.498247998 | 6.148055156 | 5.50E-06 | 6.96E-05 | 4.037195109 |
| RSBN1L | 1.133932194 | 9.263832644 | 6.147328394 | 5.51E-06 | 6.97E-05 | 4.035633366 |
| TRIM4 | 0.882551044 | 10.35829232 | 6.147083598 | 5.51E-06 | 6.97E-05 | 4.035107308 |
| PTPRC | 1.342728384 | 12.6638878 | 6.146231287 | 5.52E-06 | 6.98E-05 | 4.033275663 |
| HNRNPA0 | 1.205866222 | 7.149373416 | 6.144339666 | 5.55E-06 | 7.00E-05 | 4.029210183 |
| CAPN7 | 2.413554298 | 5.736886004 | 6.14210497 | 5.57E-06 | 7.03E-05 | 4.024406797 |
| RASA1 | 1.283186708 | 8.611924427 | 6.141796818 | 5.58E-06 | 7.03E-05 | 4.023744389 |
| SYNPO | -1.584214706 | 8.232162307 | -6.14014623 | 5.60E-06 | 7.05E-05 | 4.020196059 |
| TRAPPC9 | -0.780341177 | 10.18351514 | -6.139039479 | 5.61E-06 | 7.06E-05 | 4.017816646 |
| PIBF1 | 1.603692407 | 6.32546832 | 6.137606609 | 5.63E-06 | 7.08E-05 | 4.014735883 |
| DIMT1 | 0.776528712 | 9.808055906 | 6.136850713 | 5.64E-06 | 7.08E-05 | 4.013110555 |
| CXorf56 | 1.785993202 | 5.588981702 | 6.13613831 | 5.64E-06 | 7.09E-05 | 4.011578681 |
| SERINC1 | 1.393667188 | 10.98870206 | 6.136079791 | 5.65E-06 | 7.09E-05 | 4.011452846 |
| ITGB5 | -2.372546326 | 7.65918614 | -6.135699399 | 5.65E-06 | 7.09E-05 | 4.010634864 |
| ADAT2 | 1.207671353 | 11.23415059 | 6.134173199 | 5.67E-06 | 7.10E-05 | 4.007352797 |
| PLAC1 | -1.411462769 | 2.953685329 | -6.133803734 | 5.67E-06 | 7.10E-05 | 4.006558226 |
| FBXO28 | 0.991077034 | 8.171735995 | 6.133287401 | 5.68E-06 | 7.10E-05 | 4.005447772 |
| SERP1 | 1.089421813 | 9.421719375 | 6.133226321 | 5.68E-06 | 7.10E-05 | 4.005316408 |
| SNAPC3 | 1.284959037 | 6.617849747 | 6.126998 | 5.76E-06 | 7.19E-05 | 3.991918803 |
| C16orf52 | 0.96008201 | 8.497551118 | 6.126826411 | 5.76E-06 | 7.19E-05 | 3.991549634 |
| STX7 | 1.463806348 | 8.446838468 | 6.12527166 | 5.78E-06 | 7.21E-05 | 3.988204464 |
| CCDC112 | 2.166511847 | 5.572009188 | 6.123582172 | 5.80E-06 | 7.23E-05 | 3.984569059 |
| RPL22L1 | 2.463146426 | 11.25682173 | 6.122294878 | 5.82E-06 | 7.25E-05 | 3.98179885 |
| ZNF45 | 1.490586704 | 6.351444483 | 6.121532399 | 5.83E-06 | 7.25E-05 | 3.980157929 |
| DNAJC5G | -3.316161254 | 5.44611557 | -6.121393482 | 5.83E-06 | 7.25E-05 | 3.97985896 |
| PPP1R15A | -1.649556906 | 8.870796636 | -6.118110523 | 5.87E-06 | 7.30E-05 | 3.972792869 |
| EAF2 | 2.059817204 | 7.727470239 | 6.115771856 | 5.90E-06 | 7.33E-05 | 3.967758419 |
| POMP | 1.941464073 | 5.055145272 | 6.114870963 | 5.91E-06 | 7.34E-05 | 3.965818888 |
| ZADH2 | 0.923224217 | 10.62278235 | 6.114262327 | 5.92E-06 | 7.34E-05 | 3.964508499 |
| NMUR2 | -1.166277394 | 5.297248264 | -6.114090703 | 5.92E-06 | 7.34E-05 | 3.964138986 |
| ADIPOQ | -0.847191699 | 3.696672452 | -6.113712517 | 5.93E-06 | 7.34E-05 | 3.963324725 |
| GABRP | -3.845673373 | 9.926309537 | -6.113020927 | 5.93E-06 | 7.35E-05 | 3.961835635 |
| SEPSECS | 1.123154546 | 5.243321313 | 6.112688304 | 5.94E-06 | 7.35E-05 | 3.96111943 |
| ZNF711 | 1.700059944 | 3.125181514 | 6.110579085 | 5.97E-06 | 7.38E-05 | 3.956577538 |
| TRPS1 | 1.079150325 | 7.094978043 | 6.108412407 | 5.99E-06 | 7.41E-05 | 3.95191135 |
| LCE1B | -3.60369074 | 6.981828026 | -6.108098961 | 6.00E-06 | 7.41E-05 | 3.951236259 |
| NEGR1 | -3.536229977 | 9.451389977 | -6.106670961 | 6.02E-06 | 7.42E-05 | 3.948160529 |
| AGGF1 | 1.335577452 | 7.961547322 | 6.103439427 | 6.06E-06 | 7.47E-05 | 3.941199289 |
| WDR35 | 1.613474703 | 6.209473878 | 6.103233662 | 6.06E-06 | 7.47E-05 | 3.940755995 |
| ST6GALNAC4 | -1.996547053 | 9.222155859 | -6.102935084 | 6.07E-06 | 7.47E-05 | 3.940112738 |
| AHSG | -3.891924419 | 5.979257519 | -6.101867911 | 6.08E-06 | 7.48E-05 | 3.937813531 |
| KCTD12 | 1.257620185 | 11.61824976 | 6.098454769 | 6.12E-06 | 7.53E-05 | 3.930459032 |
| TMEM219 | -1.428337311 | 10.87102948 | -6.09842037 | 6.12E-06 | 7.53E-05 | 3.930384904 |
| MAG | -1.818374264 | 7.009599019 | -6.097296716 | 6.14E-06 | 7.54E-05 | 3.927963384 |
| ZNF540 | 1.75042597 | 6.606175709 | 6.09532557 | 6.17E-06 | 7.57E-05 | 3.92371511 |
| KCNC3 | -2.596076535 | 8.1491203 | -6.094699177 | 6.17E-06 | 7.57E-05 | 3.92236499 |
| SLC1A5 | -1.836384709 | 7.604943837 | -6.093363917 | 6.19E-06 | 7.59E-05 | 3.919486826 |
| STRC | -1.442790489 | 6.472736595 | -6.091612161 | 6.22E-06 | 7.61E-05 | 3.915710569 |
| NAT14 | -2.533570226 | 9.449446493 | -6.090591137 | 6.23E-06 | 7.62E-05 | 3.913509377 |
| WIPF2 | -1.1134119 | 11.35627858 | -6.088547884 | 6.26E-06 | 7.65E-05 | 3.909104013 |
| FAM131A | -1.882474202 | 6.546852617 | -6.087879945 | 6.27E-06 | 7.66E-05 | 3.907663789 |
| SLAIN1 | 1.27826728 | 5.641447085 | 6.087609831 | 6.27E-06 | 7.66E-05 | 3.90708135 |
| QSER1 | 1.049826029 | 8.205462321 | 6.083440426 | 6.33E-06 | 7.72E-05 | 3.898089829 |
| ENOPH1 | 1.092690679 | 8.686759888 | 6.082822984 | 6.34E-06 | 7.73E-05 | 3.896758105 |
| TNFRSF8 | -1.137370874 | 10.94040924 | -6.082273972 | 6.34E-06 | 7.73E-05 | 3.895573935 |
| CDC73 | 2.620351816 | 6.311300381 | 6.081641853 | 6.35E-06 | 7.74E-05 | 3.894210465 |
| LPP | 2.453338935 | 5.409342777 | 6.080610197 | 6.37E-06 | 7.75E-05 | 3.891985096 |
| THOC1 | 1.702101657 | 6.634801444 | 6.080180665 | 6.37E-06 | 7.75E-05 | 3.891058521 |
| ARMCX5 | 1.141114792 | 7.733395842 | 6.079719556 | 6.38E-06 | 7.75E-05 | 3.890063803 |
| RFXAP | 1.75621332 | 6.938843931 | 6.077747724 | 6.41E-06 | 7.78E-05 | 3.885809821 |
| ZNF292 | 1.72230861 | 7.86048733 | 6.077167478 | 6.41E-06 | 7.78E-05 | 3.884557922 |
| FNIP1 | 2.210938001 | 7.367629509 | 6.076576576 | 6.42E-06 | 7.79E-05 | 3.883282991 |
| TARDBP | 0.584421149 | 10.69212761 | 6.074421577 | 6.45E-06 | 7.82E-05 | 3.878633 |
| SLC25A40 | 1.704905396 | 6.551110253 | 6.071728604 | 6.49E-06 | 7.86E-05 | 3.872821391 |
| ZNF493 | 1.33863273 | 7.91728523 | 6.066580408 | 6.56E-06 | 7.94E-05 | 3.861708799 |
| STK38L | 1.871941053 | 5.058114375 | 6.063267291 | 6.61E-06 | 7.99E-05 | 3.8545556 |
| RAD51B | -1.938028642 | 6.623701345 | -6.061462436 | 6.64E-06 | 8.02E-05 | 3.850658259 |
| PGBD2 | 0.980849849 | 9.203334202 | 6.059478645 | 6.66E-06 | 8.05E-05 | 3.846374074 |
| PIM1 | -1.175083501 | 13.74745987 | -6.058768996 | 6.68E-06 | 8.06E-05 | 3.844841402 |
| DDX17 | 1.522024939 | 11.54436006 | 6.058191997 | 6.68E-06 | 8.06E-05 | 3.843595181 |
| CXCL12 | -1.926175016 | 6.043916278 | -6.057620057 | 6.69E-06 | 8.07E-05 | 3.842359843 |
| TUBA3C | -1.735397937 | 9.213138604 | -6.056823468 | 6.70E-06 | 8.07E-05 | 3.84063922 |
| HEPACAM2 | -2.451041151 | 6.825696503 | -6.0563826 | 6.71E-06 | 8.08E-05 | 3.839686919 |
| POLI | 1.877559227 | 7.355697842 | 6.054609195 | 6.74E-06 | 8.10E-05 | 3.835856012 |
| CEP135 | 1.201447333 | 8.496465888 | 6.054058923 | 6.74E-06 | 8.11E-05 | 3.83466724 |
| PTGS1 | -1.493422469 | 8.828076305 | -6.051777506 | 6.78E-06 | 8.14E-05 | 3.829738215 |
| CLEC2D | 1.247103016 | 10.5457994 | 6.051167946 | 6.79E-06 | 8.15E-05 | 3.828421149 |
| ZNF280B | 1.198561202 | 4.812462515 | 6.04939444 | 6.81E-06 | 8.17E-05 | 3.824588907 |
| PRDX6 | -1.478416562 | 14.18755617 | -6.046964159 | 6.85E-06 | 8.21E-05 | 3.819336869 |
| STYX | 1.049348638 | 9.541497447 | 6.046847504 | 6.85E-06 | 8.21E-05 | 3.81908475 |
| FBXO3 | 1.409706684 | 6.360040555 | 6.045881337 | 6.86E-06 | 8.22E-05 | 3.816996572 |
| GDAP1 | 1.383394013 | 6.47739968 | 6.044741564 | 6.88E-06 | 8.23E-05 | 3.814533033 |
| POU2F2 | -2.847485326 | 8.627026385 | -6.041214932 | 6.93E-06 | 8.29E-05 | 3.806909476 |
| FCHSD2 | 1.02575826 | 7.69988221 | 6.040481512 | 6.95E-06 | 8.30E-05 | 3.805323846 |
| HSF5 | 1.993912217 | 4.571545797 | 6.039894551 | 6.95E-06 | 8.30E-05 | 3.804054808 |
| FER | 1.481248927 | 4.631399729 | 6.038700164 | 6.97E-06 | 8.32E-05 | 3.801472359 |
| IFT57 | 2.032601407 | 3.426377158 | 6.037174238 | 7.00E-06 | 8.34E-05 | 3.798172821 |
| GPR160 | 0.89324099 | 8.95810192 | 6.036291992 | 7.01E-06 | 8.35E-05 | 3.796264996 |
| BPIFB4 | -2.008948315 | 4.054050204 | -6.035107294 | 7.03E-06 | 8.36E-05 | 3.793702982 |
| AHSP | -2.803971664 | 13.00216843 | -6.034973705 | 7.03E-06 | 8.36E-05 | 3.793414074 |
| KIAA1919 | 1.074868445 | 5.755450107 | 6.033542555 | 7.05E-06 | 8.38E-05 | 3.790318837 |
| PCYT2 | -1.424810703 | 9.17617664 | -6.030597144 | 7.10E-06 | 8.43E-05 | 3.783947841 |
| FAM208B | 1.713407652 | 7.868409551 | 6.029776809 | 7.11E-06 | 8.44E-05 | 3.78217325 |
| RABGGTB | 1.75763222 | 7.269359096 | 6.029019634 | 7.12E-06 | 8.45E-05 | 3.780535219 |
| RP2 | 1.390651563 | 9.189674876 | 6.028892977 | 7.12E-06 | 8.45E-05 | 3.780261209 |
| TJP3 | -2.98372327 | 6.794972301 | -6.027807954 | 7.14E-06 | 8.46E-05 | 3.77791379 |
| AHI1 | 1.662565639 | 4.712823571 | 6.026856942 | 7.15E-06 | 8.47E-05 | 3.775856187 |
| CYP20A1 | 0.821235033 | 8.698171229 | 6.025845255 | 7.17E-06 | 8.48E-05 | 3.773667188 |
| ZNF436 | 0.990289456 | 6.251875424 | 6.02540884 | 7.18E-06 | 8.48E-05 | 3.772722871 |
| TMEM107 | 0.707245692 | 10.51759834 | 6.025306166 | 7.18E-06 | 8.48E-05 | 3.772500703 |
| ODF2L | 1.242373119 | 13.93335713 | 6.022259516 | 7.23E-06 | 8.53E-05 | 3.765907688 |
| EDIL3 | -3.362032271 | 3.782214616 | -6.017531112 | 7.30E-06 | 8.61E-05 | 3.755673117 |
| DDX3Y | 2.544994763 | 3.7586791 | 6.01741584 | 7.30E-06 | 8.61E-05 | 3.75542358 |
| FANCM | 1.919845596 | 7.216514038 | 6.015999291 | 7.33E-06 | 8.63E-05 | 3.752356935 |
| USP6NL | 2.33586276 | 4.901136839 | 6.014759595 | 7.35E-06 | 8.65E-05 | 3.749672957 |
| ZNF546 | 2.283543517 | 4.640640528 | 6.014259627 | 7.35E-06 | 8.66E-05 | 3.74859046 |
| THAP2 | 2.024523617 | 5.338418409 | 6.010222732 | 7.42E-06 | 8.73E-05 | 3.73984895 |
| C14orf28 | 2.019360626 | 5.388347623 | 6.009259174 | 7.43E-06 | 8.74E-05 | 3.73776217 |
| ASF1B | -1.566334429 | 8.601052944 | -6.007670136 | 7.46E-06 | 8.76E-05 | 3.73432054 |
| HNRNPCL1 | 2.634336179 | 3.883235156 | 6.004739023 | 7.51E-06 | 8.81E-05 | 3.72797138 |
| PIGK | 1.372366025 | 6.957066882 | 6.002603288 | 7.54E-06 | 8.85E-05 | 3.723344463 |
| NFYB | 2.035188867 | 6.356537855 | 6.001739882 | 7.56E-06 | 8.86E-05 | 3.721473801 |
| NHLRC2 | 1.755322353 | 4.962657772 | 6.001489244 | 7.56E-06 | 8.86E-05 | 3.72093075 |
| MARK3 | -0.828574601 | 12.59731902 | -6.000718453 | 7.57E-06 | 8.87E-05 | 3.719260649 |
| ZNF446 | -0.958363839 | 11.35182796 | -5.999476628 | 7.59E-06 | 8.89E-05 | 3.716569794 |
| ZNF189 | 1.021006573 | 8.804098619 | 5.996931231 | 7.64E-06 | 8.93E-05 | 3.711053712 |
| ZFP82 | 1.603972484 | 5.759198161 | 5.996693446 | 7.64E-06 | 8.93E-05 | 3.710538374 |
| SLC30A9 | 2.140927827 | 5.41185976 | 5.994503459 | 7.68E-06 | 8.96E-05 | 3.705791813 |
| HPRT1 | 1.071201655 | 9.084554869 | 5.994110237 | 7.68E-06 | 8.97E-05 | 3.704939486 |
| SYNE2 | 1.048210819 | 11.10863975 | 5.990055579 | 7.75E-06 | 9.04E-05 | 3.696149758 |
| ZNF98 | 1.750057849 | 10.24086012 | 5.987787175 | 7.79E-06 | 9.08E-05 | 3.691231436 |
| PEX2 | 1.62565678 | 5.182429414 | 5.987047949 | 7.80E-06 | 9.09E-05 | 3.689628523 |
| ZNF596 | 1.491816947 | 4.236330063 | 5.986175854 | 7.82E-06 | 9.10E-05 | 3.687737419 |
| HAAO | -1.02858515 | 7.366040517 | -5.985568214 | 7.83E-06 | 9.10E-05 | 3.686419724 |
| HIST1H4E | 3.109473201 | 10.28826614 | 5.984491599 | 7.85E-06 | 9.12E-05 | 3.684084923 |
| MRM1 | -0.951920091 | 8.858077159 | -5.983766543 | 7.86E-06 | 9.13E-05 | 3.682512455 |
| NEIL1 | -1.033399817 | 10.35771797 | -5.982702107 | 7.88E-06 | 9.14E-05 | 3.68020384 |
| ZNF750 | -1.770152508 | 4.214378259 | -5.980731033 | 7.91E-06 | 9.18E-05 | 3.675928498 |
| LCE3D | -2.78187148 | 5.925578038 | -5.979203848 | 7.94E-06 | 9.20E-05 | 3.672615652 |
| MIA | -0.76639064 | 7.965046893 | -5.978724987 | 7.95E-06 | 9.20E-05 | 3.671576826 |
| FAM69B | -1.734795612 | 8.006213514 | -5.978236257 | 7.95E-06 | 9.21E-05 | 3.670516562 |
| MSI2 | -1.116447679 | 9.011914413 | -5.972405094 | 8.06E-06 | 9.32E-05 | 3.657864103 |
| SLC22A10 | -3.136406306 | 6.711364085 | -5.971083472 | 8.08E-06 | 9.34E-05 | 3.654995888 |
| NCOA1 | -1.409976879 | 10.56334746 | -5.970370168 | 8.09E-06 | 9.35E-05 | 3.653447774 |
| VRK2 | 1.703267964 | 7.480415771 | 5.965635405 | 8.18E-06 | 9.44E-05 | 3.643170184 |
| FUBP3 | 1.535322276 | 6.859615684 | 5.96479414 | 8.19E-06 | 9.45E-05 | 3.641343802 |
| TSHB | -1.229079469 | 6.620075487 | -5.96365912 | 8.21E-06 | 9.47E-05 | 3.638879545 |
| PPP1CB | 0.910609846 | 10.51799386 | 5.96336267 | 8.22E-06 | 9.47E-05 | 3.638235893 |
| ZBTB7B | -0.803503984 | 8.598897074 | -5.962249327 | 8.24E-06 | 9.49E-05 | 3.635818516 |
| TLR3 | 1.525117244 | 5.44379101 | 5.959547432 | 8.29E-06 | 9.54E-05 | 3.629951338 |
| TMED7-TICAM2 | 0.868053835 | 7.610397644 | 5.958065626 | 8.31E-06 | 9.56E-05 | 3.626733223 |
| DBF4B | -2.247480418 | 7.286956353 | -5.953336603 | 8.40E-06 | 9.65E-05 | 3.61646123 |
| CABP5 | -3.079769386 | 9.138773194 | -5.951255885 | 8.44E-06 | 9.69E-05 | 3.611940832 |
| C1orf95 | 1.28129404 | 4.127625373 | 5.949559327 | 8.47E-06 | 9.72E-05 | 3.608254651 |
| FLI1 | 1.070370469 | 11.39474045 | 5.943538652 | 8.58E-06 | 9.84E-05 | 3.595170559 |
| NME4 | -1.48206499 | 8.672683397 | -5.940224608 | 8.64E-06 | 9.91E-05 | 3.587966683 |
| TMEM145 | -3.186861672 | 9.135713738 | -5.939190236 | 8.66E-06 | 9.92E-05 | 3.585717963 |
| MAPK1IP1L | 0.80356072 | 10.63806849 | 5.939112838 | 8.66E-06 | 9.92E-05 | 3.585549696 |
| MBIP | 1.047789649 | 7.880413745 | 5.937004985 | 8.70E-06 | 9.96E-05 | 3.580966824 |
| COBLL1 | 2.206522987 | 7.396957965 | 5.936015765 | 8.72E-06 | 9.97E-05 | 3.578815893 |
| C17orf58 | 1.579584463 | 3.29913402 | 5.936001033 | 8.72E-06 | 9.97E-05 | 3.578783858 |
| ZNF571 | 1.688432513 | 5.784587134 | 5.935466585 | 8.73E-06 | 9.97E-05 | 3.577621722 |
| CLIP4 | 1.257297733 | 7.472467056 | 5.934706229 | 8.75E-06 | 9.98E-05 | 3.5759683 |
| ICA1 | -1.444281183 | 8.274785147 | -5.933620106 | 8.77E-06 | 1.00E-04 | 3.573606367 |
| SPEF2 | 1.770293348 | 4.575825651 | 5.932997793 | 8.78E-06 | 0.000100067 | 3.572252993 |
| STEAP3 | -1.347079347 | 7.14301424 | -5.93109209 | 8.82E-06 | 0.00010042 | 3.568108293 |
| ALCAM | 1.871745272 | 6.833138757 | 5.928854978 | 8.86E-06 | 0.000100826 | 3.56324227 |
| ABCB10 | 1.84891117 | 6.680872285 | 5.928654062 | 8.86E-06 | 0.000100826 | 3.562805223 |
| RFK | 1.622031224 | 7.314668814 | 5.926008258 | 8.92E-06 | 0.000101333 | 3.557049425 |
| TTC14 | 1.314855559 | 9.262986967 | 5.925770108 | 8.92E-06 | 0.000101333 | 3.556531302 |
| EFCAB7 | 2.193601997 | 6.417527635 | 5.923942079 | 8.96E-06 | 0.000101645 | 3.552553999 |
| PCM1 | 0.989690977 | 10.543164 | 5.923775631 | 8.96E-06 | 0.000101645 | 3.552191833 |
| SLC5A6 | -1.424070231 | 8.35407642 | -5.921157863 | 9.01E-06 | 0.000102163 | 3.546495539 |
| PLEKHG1 | 1.96080379 | 4.641583123 | 5.917725106 | 9.08E-06 | 0.000102867 | 3.539024609 |
| LRPPRC | 1.174626591 | 7.949245099 | 5.916200405 | 9.11E-06 | 0.000103145 | 3.535705864 |
| P2RY10 | 1.588648318 | 7.163991312 | 5.913464545 | 9.16E-06 | 0.000103698 | 3.529750169 |
| TREML1 | -2.412495731 | 9.338697589 | -5.910103658 | 9.23E-06 | 0.000104397 | 3.522432661 |
| LINGO3 | -0.981426417 | 5.709393339 | -5.907690776 | 9.28E-06 | 0.000104882 | 3.517178392 |
| REEP1 | -4.845017302 | 6.880473606 | -5.907105117 | 9.29E-06 | 0.00010493 | 3.515902964 |
| PKN2 | 1.581982025 | 8.443752544 | 5.906895863 | 9.30E-06 | 0.00010493 | 3.515447249 |
| ZNF674 | 1.631870972 | 3.247250859 | 5.905536032 | 9.32E-06 | 0.000105121 | 3.51248567 |
| BTG3 | 1.201494148 | 8.429752177 | 5.905481837 | 9.33E-06 | 0.000105121 | 3.512367634 |
| PPIL4 | 1.660172493 | 6.681053926 | 5.904087657 | 9.35E-06 | 0.000105375 | 3.509331016 |
| MRPL19 | 0.795677913 | 9.316831912 | 5.903135432 | 9.37E-06 | 0.000105527 | 3.507256877 |
| AZI2 | 1.455598567 | 7.576388183 | 5.902644014 | 9.38E-06 | 0.000105573 | 3.506186427 |
| VWA1 | -2.024549117 | 8.713350185 | -5.900884072 | 9.42E-06 | 0.000105914 | 3.502352539 |
| SUMO2 | 0.831792268 | 12.51835926 | 5.898606498 | 9.47E-06 | 0.000106375 | 3.497390499 |
| BAZ2B | 1.856892766 | 7.495001946 | 5.895760411 | 9.53E-06 | 0.000106877 | 3.491189023 |
| BSG | -1.954723888 | 11.95725711 | -5.895706795 | 9.53E-06 | 0.000106877 | 3.491072188 |
| ZNF852 | 2.406260341 | 4.885871277 | 5.895587908 | 9.53E-06 | 0.000106877 | 3.490813117 |
| ZNF550 | 1.99286529 | 5.556563211 | 5.893882397 | 9.57E-06 | 0.000107209 | 3.487096415 |
| HECTD3 | -0.755794912 | 8.210286922 | -5.893417575 | 9.58E-06 | 0.00010725 | 3.486083404 |
| HERC4 | 1.362410776 | 8.305441717 | 5.890282459 | 9.64E-06 | 0.00010792 | 3.479250217 |
| TONSL | -1.574857276 | 6.987373169 | -5.886065996 | 9.73E-06 | 0.000108854 | 3.470058376 |
| CLECL1 | 2.482937363 | 6.596678513 | 5.885271329 | 9.75E-06 | 0.000108974 | 3.468325781 |
| WDR43 | 1.469987305 | 7.151700764 | 5.88416236 | 9.77E-06 | 0.00010917 | 3.4659078 |
| CDKL3 | 1.359414042 | 5.005918316 | 5.883173223 | 9.79E-06 | 0.000109338 | 3.463750979 |
| FAM208A | 1.392788266 | 7.99260001 | 5.878901079 | 9.89E-06 | 0.000110297 | 3.454434243 |
| DNAL4 | -3.228111585 | 7.60130328 | -5.877546362 | 9.91E-06 | 0.000110555 | 3.451479422 |
| PPIL2 | -1.107667717 | 7.826824466 | -5.875434064 | 9.96E-06 | 0.000110998 | 3.446871797 |
| TRIM16 | 1.607273654 | 5.108668679 | 5.874897596 | 9.97E-06 | 0.000111059 | 3.4457015 |
| TLN1 | -0.900360526 | 9.186536556 | -5.873807917 | 1.00E-05 | 0.000111254 | 3.443324279 |
| FAM179B | 1.454806242 | 6.761349746 | 5.872762407 | 1.00E-05 | 0.000111388 | 3.441043287 |
| TGM1 | -1.10326505 | 7.609650856 | -5.87256725 | 1.00E-05 | 0.000111388 | 3.440617501 |
| ELMOD2 | 1.560594809 | 8.888014398 | 5.872394065 | 1.00E-05 | 0.000111388 | 3.440239647 |
| NPM1 | 1.326975465 | 13.58003425 | 5.870906896 | 1.01E-05 | 0.000111656 | 3.436994809 |
| GFPT1 | 1.187385723 | 6.33829295 | 5.870580256 | 1.01E-05 | 0.000111656 | 3.436282081 |
| ZFP36L1 | 1.107053567 | 13.25372123 | 5.87043676 | 1.01E-05 | 0.000111656 | 3.435968971 |
| RNMT | 1.673466266 | 7.322599101 | 5.870020365 | 1.01E-05 | 0.000111688 | 3.435060375 |
| RAB18 | 1.686592834 | 7.52448751 | 5.869284387 | 1.01E-05 | 0.000111798 | 3.433454385 |
| CHRNE | -1.789177615 | 6.878092744 | -5.868087548 | 1.01E-05 | 0.000112022 | 3.430842608 |
| NACC1 | -0.842370421 | 10.60853033 | -5.867030022 | 1.01E-05 | 0.000112211 | 3.428534707 |
| KLHL2 | 2.109038097 | 7.269417699 | 5.865619247 | 1.02E-05 | 0.000112489 | 3.425455691 |
| SHPRH | 1.163947095 | 7.442193352 | 5.863708352 | 1.02E-05 | 0.000112868 | 3.421284801 |
| AP4S1 | 1.283827429 | 5.282964207 | 5.863512544 | 1.02E-05 | 0.000112868 | 3.42085739 |
| OR4D10 | -1.841306323 | 6.694873133 | -5.860998859 | 1.03E-05 | 0.000113422 | 3.415370102 |
| ELOVL7 | -1.564493714 | 6.259219421 | -5.859219838 | 1.03E-05 | 0.000113795 | 3.411486125 |
| MAP3K11 | -1.01177808 | 14.49926367 | -5.858908678 | 1.03E-05 | 0.000113801 | 3.410806761 |
| CGREF1 | -2.10881924 | 7.360109995 | -5.857496466 | 1.04E-05 | 0.000114083 | 3.407723297 |
| PALM3 | -2.032996237 | 6.966402181 | -5.856539765 | 1.04E-05 | 0.000114203 | 3.405634281 |
| TADA1 | 0.92506861 | 7.357009148 | 5.856448772 | 1.04E-05 | 0.000114203 | 3.405435585 |
| IQSEC2 | -2.558164219 | 12.62719817 | -5.855785434 | 1.04E-05 | 0.000114298 | 3.403987077 |
| ATG2B | 1.044641648 | 8.773668452 | 5.853826725 | 1.04E-05 | 0.000114688 | 3.399709617 |
| SAMD14 | -1.636775869 | 5.244562522 | -5.853664404 | 1.04E-05 | 0.000114688 | 3.399355117 |
| GOLPH3 | 1.537737688 | 8.429840684 | 5.853242649 | 1.05E-05 | 0.000114723 | 3.398434018 |
| NAPB | 1.214961016 | 8.366825512 | 5.851711712 | 1.05E-05 | 0.000115038 | 3.395090326 |
| PDE4B | 0.80535068 | 10.92780806 | 5.851055276 | 1.05E-05 | 0.000115132 | 3.393656535 |
| RNPC3 | 1.181059432 | 11.04215719 | 5.848404194 | 1.06E-05 | 0.000115733 | 3.387865526 |
| FAM161A | 1.396352238 | 4.383787974 | 5.847940081 | 1.06E-05 | 0.00011576 | 3.386851637 |
| ZMYM5 | 1.215115559 | 6.14015593 | 5.847731113 | 1.06E-05 | 0.00011576 | 3.386395124 |
| MIER1 | 1.40820893 | 9.001615987 | 5.844633158 | 1.07E-05 | 0.000116478 | 3.379626716 |
| ST3GAL3 | -1.09470851 | 7.314135219 | -5.843150447 | 1.07E-05 | 0.000116786 | 3.376386906 |
| KRTAP5-2 | -1.887569521 | 8.807353679 | -5.842464035 | 1.07E-05 | 0.000116889 | 3.374886973 |
| SYNC | 1.939575641 | 5.352791973 | 5.840074706 | 1.08E-05 | 0.000117432 | 3.369665435 |
| ITGA4 | 1.647836766 | 9.854861677 | 5.839289036 | 1.08E-05 | 0.000117562 | 3.367948325 |
| THEM5 | -3.158261237 | 8.374826661 | -5.83892128 | 1.08E-05 | 0.000117584 | 3.367144558 |
| ESF1 | 2.299217788 | 6.715461093 | 5.838467915 | 1.08E-05 | 0.000117629 | 3.366153661 |
| NCF4 | -1.252121271 | 12.54514954 | -5.836367697 | 1.09E-05 | 0.0001181 | 3.361563017 |
| SPG20 | 1.600586455 | 5.122632646 | 5.835741289 | 1.09E-05 | 0.00011819 | 3.360193721 |
| PTPDC1 | 0.845043669 | 6.999153334 | 5.835019672 | 1.09E-05 | 0.000118249 | 3.35861625 |
| KIF21A | 2.181544766 | 3.671553945 | 5.834951868 | 1.09E-05 | 0.000118249 | 3.358468025 |
| RNF139 | 1.093353693 | 9.5851303 | 5.832916303 | 1.09E-05 | 0.000118663 | 3.354017895 |
| MAN1A2 | 1.256690909 | 8.605293722 | 5.832798656 | 1.09E-05 | 0.000118663 | 3.353760684 |
| MGEA5 | 0.846749719 | 13.7952222 | 5.829658691 | 1.10E-05 | 0.000119385 | 3.346895168 |
| ARL1 | 0.718738088 | 9.147719635 | 5.829480535 | 1.10E-05 | 0.000119385 | 3.346505596 |
| GOLGB1 | 2.051550772 | 7.575161943 | 5.825823103 | 1.11E-05 | 0.000120276 | 3.33850716 |
| UBB | -1.091735671 | 17.05934651 | -5.824831217 | 1.11E-05 | 0.000120464 | 3.336337746 |
| LY75 | 1.25231573 | 8.550655371 | 5.824120167 | 1.11E-05 | 0.000120579 | 3.334782496 |
| EXOC1 | 1.398792305 | 8.219149313 | 5.821052813 | 1.12E-05 | 0.000121321 | 3.328072748 |
| GNE | 0.747686971 | 9.14811024 | 5.818302331 | 1.13E-05 | 0.000121982 | 3.322055247 |
| PHLPP2 | 1.022736456 | 6.448122298 | 5.817996414 | 1.13E-05 | 0.00012199 | 3.321385909 |
| PTS | 1.570623957 | 7.954626051 | 5.81680223 | 1.13E-05 | 0.000122236 | 3.318772968 |
| KTN1 | 1.943854612 | 10.47648291 | 5.814757225 | 1.14E-05 | 0.000122712 | 3.314298009 |
| TWISTNB | 0.99259236 | 7.013534805 | 5.812931346 | 1.14E-05 | 0.000123131 | 3.310302154 |
| EDNRA | -1.825687467 | 5.81555091 | -5.812280462 | 1.14E-05 | 0.000123232 | 3.308877632 |
| VEZT | 1.509960113 | 7.109681742 | 5.811616298 | 1.15E-05 | 0.000123336 | 3.307423996 |
| FASTKD3 | 1.011681883 | 8.247918466 | 5.810540826 | 1.15E-05 | 0.000123539 | 3.30507004 |
| KRTAP10-8 | -2.218866103 | 8.550297792 | -5.81031515 | 1.15E-05 | 0.000123539 | 3.304576071 |
| WFDC3 | -1.258096173 | 8.285509059 | -5.808627221 | 1.15E-05 | 0.000123868 | 3.300881282 |
| CLCN1 | -2.482462205 | 7.487212042 | -5.80855131 | 1.15E-05 | 0.000123868 | 3.30071511 |
| AGTPBP1 | 1.333158857 | 9.312044608 | 5.805741669 | 1.16E-05 | 0.000124438 | 3.294564222 |
| TFEC | 1.662538976 | 7.60136737 | 5.805653336 | 1.16E-05 | 0.000124438 | 3.294370828 |
| ZNF185 | -1.225753005 | 8.983822823 | -5.805637696 | 1.16E-05 | 0.000124438 | 3.294336586 |
| KCNK16 | -2.565048766 | 4.862129995 | -5.804147913 | 1.17E-05 | 0.000124771 | 3.291074764 |
| FRYL | 1.387518729 | 8.929295867 | 5.803105158 | 1.17E-05 | 0.000124982 | 3.288791544 |
| LMNA | -1.713526081 | 10.28352918 | -5.801072789 | 1.17E-05 | 0.000125466 | 3.284341112 |
| PARP15 | 1.920805925 | 7.442708062 | 5.796953509 | 1.18E-05 | 0.000126533 | 3.275319398 |
| FAM169A | 1.565374736 | 7.293613163 | 5.796384507 | 1.19E-05 | 0.000126615 | 3.274073067 |
| GPR174 | 2.504316168 | 7.319469842 | 5.793155613 | 1.19E-05 | 0.000127442 | 3.26699987 |
| AMIGO2 | 1.624596761 | 7.742546656 | 5.791273351 | 1.20E-05 | 0.000127829 | 3.262876062 |
| BTBD7 | 1.181012008 | 6.891721348 | 5.791227785 | 1.20E-05 | 0.000127829 | 3.262776228 |
| SYNCRIP | 1.50217871 | 8.838947954 | 5.788271661 | 1.21E-05 | 0.000128587 | 3.256298904 |
| C1QTNF6 | -1.101508427 | 8.198713214 | -5.78769476 | 1.21E-05 | 0.000128672 | 3.255034712 |
| SV2C | -2.170471773 | 6.350708064 | -5.78720889 | 1.21E-05 | 0.000128732 | 3.253969972 |
| TRMT11 | 2.185696122 | 6.33202191 | 5.783677904 | 1.22E-05 | 0.00012966 | 3.246231344 |
| PAN3 | 1.485635818 | 6.833181937 | 5.782883335 | 1.22E-05 | 0.000129809 | 3.244489747 |
| NAPEPLD | 1.090268345 | 8.285931371 | 5.781504508 | 1.22E-05 | 0.000130055 | 3.241467366 |
| DBF4 | 1.628965472 | 5.640248209 | 5.781475446 | 1.22E-05 | 0.000130055 | 3.24140366 |
| YES1 | 1.605287225 | 5.324863514 | 5.780011694 | 1.23E-05 | 0.000130397 | 3.238194885 |
| DOCK4 | 2.217311895 | 5.190742955 | 5.779662686 | 1.23E-05 | 0.000130419 | 3.23742977 |
| RRP12 | -1.05002551 | 8.83945533 | -5.779359042 | 1.23E-05 | 0.000130427 | 3.236764093 |
| GALNT4 | 2.109717949 | 4.133254725 | 5.777664551 | 1.24E-05 | 0.000130837 | 3.233049084 |
| SRGAP2 | -0.841789809 | 10.54110746 | -5.777095447 | 1.24E-05 | 0.000130922 | 3.231801305 |
| STK17A | 1.739078895 | 5.701123552 | 5.773423604 | 1.25E-05 | 0.000131908 | 3.223749816 |
| IPO8 | 0.960426596 | 9.086720864 | 5.772850626 | 1.25E-05 | 0.000131995 | 3.222493275 |
| TLK1 | 2.54684149 | 4.980521771 | 5.771863608 | 1.25E-05 | 0.000132203 | 3.220328659 |
| ARPP19 | 0.936072179 | 9.125312795 | 5.768755071 | 1.26E-05 | 0.000132887 | 3.213510661 |
| METTL10 | 1.293133561 | 6.639351746 | 5.768711747 | 1.26E-05 | 0.000132887 | 3.21341563 |
| SFN | -1.098482277 | 7.356128032 | -5.768711585 | 1.26E-05 | 0.000132887 | 3.213415274 |
| ARID5B | 1.173924702 | 9.197860193 | 5.767427162 | 1.26E-05 | 0.000133184 | 3.210597815 |
| ACADM | 1.672662499 | 7.034061562 | 5.761935134 | 1.28E-05 | 0.000134727 | 3.198548655 |
| LSM11 | 1.521763803 | 3.394124746 | 5.761115544 | 1.28E-05 | 0.000134849 | 3.196750242 |
| SLC10A7 | 0.8403461 | 5.741936596 | 5.760983645 | 1.28E-05 | 0.000134849 | 3.196460809 |
| TBC1D19 | 1.457115711 | 5.637583933 | 5.756812965 | 1.29E-05 | 0.000136015 | 3.187307943 |
| SH3BP4 | -3.449931884 | 4.974985504 | -5.756100044 | 1.30E-05 | 0.000136148 | 3.185743194 |
| HMGCS1 | 1.47687912 | 6.53911665 | 5.754842467 | 1.30E-05 | 0.000136437 | 3.182982873 |
| VPS13B | 0.764606539 | 8.924658852 | 5.75459944 | 1.30E-05 | 0.000136437 | 3.182449421 |
| MTHFD2L | 0.854330149 | 2.629195391 | 5.752629493 | 1.31E-05 | 0.00013695 | 3.17812508 |
| SLC35D1 | 1.820280727 | 4.232168188 | 5.750427215 | 1.31E-05 | 0.000137536 | 3.17329023 |
| DCK | 2.299659916 | 5.391637912 | 5.749396861 | 1.31E-05 | 0.000137767 | 3.171028022 |
| KBTBD8 | 2.804120062 | 5.112893002 | 5.747015306 | 1.32E-05 | 0.000138411 | 3.165798722 |
| RBM38 | -2.268056995 | 12.10510832 | -5.743886989 | 1.33E-05 | 0.000139247 | 3.158928773 |
| DCAF6 | -0.852405864 | 8.537417554 | -5.743750612 | 1.33E-05 | 0.000139247 | 3.158629259 |
| TALDO1 | -1.124115535 | 15.53906442 | -5.743188604 | 1.33E-05 | 0.000139338 | 3.157394937 |
| ADAD1 | -1.826198661 | 4.251860601 | -5.742071475 | 1.34E-05 | 0.000139536 | 3.154941317 |
| GPR162 | -1.621357065 | 10.91937686 | -5.74200537 | 1.34E-05 | 0.000139536 | 3.154796122 |
| GLI4 | -2.003203105 | 14.32641371 | -5.740993869 | 1.34E-05 | 0.000139766 | 3.152574376 |
| RWDD3 | 1.638080055 | 8.041168029 | 5.739513042 | 1.34E-05 | 0.000140096 | 3.149321558 |
| NOC4L | -1.872433122 | 11.51244291 | -5.739387665 | 1.34E-05 | 0.000140096 | 3.149046142 |
| PPTC7 | 1.11813091 | 9.432398774 | 5.738770524 | 1.35E-05 | 0.000140205 | 3.147690438 |
| CCDC91 | 2.691451198 | 6.080649164 | 5.737518821 | 1.35E-05 | 0.00014051 | 3.14494063 |
| SLC25A30 | 0.948421782 | 7.364675231 | 5.736389158 | 1.35E-05 | 0.000140778 | 3.142458779 |
| SPRR2D | -1.639754147 | 8.63433997 | -5.734490262 | 1.36E-05 | 0.000141286 | 3.138286625 |
| ADAMTSL4 | -1.245562129 | 7.194710855 | -5.733200211 | 1.36E-05 | 0.000141606 | 3.135451966 |
| BCL9 | 2.381483226 | 4.22135642 | 5.732402176 | 1.36E-05 | 0.000141772 | 3.133698335 |
| ANAPC4 | 1.080551312 | 10.78264808 | 5.730634486 | 1.37E-05 | 0.00014208 | 3.129813701 |
| HGD | -1.822120853 | 8.497013629 | -5.73030917 | 1.37E-05 | 0.00014208 | 3.129098758 |
| C14orf166 | 1.104256408 | 12.97494861 | 5.730112938 | 1.37E-05 | 0.00014208 | 3.128667495 |
| ZNF568 | 1.058709042 | 4.771593865 | 5.729781265 | 1.37E-05 | 0.00014208 | 3.127938561 |
| CNFN | -1.919102051 | 9.202404818 | -5.729723287 | 1.37E-05 | 0.00014208 | 3.12781114 |
| CRBN | 1.333595648 | 10.54676274 | 5.729547098 | 1.37E-05 | 0.00014208 | 3.127423914 |
| ZDHHC5 | -0.677246454 | 11.63318803 | -5.72929742 | 1.37E-05 | 0.00014208 | 3.126875171 |
| CLDN5 | -2.465974998 | 8.956418599 | -5.729288711 | 1.37E-05 | 0.00014208 | 3.12685603 |
| ANKRD18B | 1.558378345 | 3.343052356 | 5.728484851 | 1.38E-05 | 0.000142249 | 3.125089257 |
| CTIF | -2.122832375 | 8.679184532 | -5.726958813 | 1.38E-05 | 0.000142647 | 3.12173504 |
| TFAP2A | -5.55300137 | 6.106302872 | -5.726680453 | 1.38E-05 | 0.000142651 | 3.12112318 |
| CCDC138 | 1.480842646 | 3.794281984 | 5.725970761 | 1.38E-05 | 0.000142791 | 3.119563177 |
| ZNHIT2 | -2.060681455 | 9.314853345 | -5.724989638 | 1.39E-05 | 0.000143017 | 3.117406438 |
| MED31 | 0.932653749 | 5.172563208 | 5.722772176 | 1.39E-05 | 0.000143636 | 3.112531553 |
| CSGALNACT2 | 1.353686537 | 8.258153419 | 5.721609444 | 1.40E-05 | 0.000143922 | 3.109975179 |
| RHBDD3 | -0.617420975 | 9.229078792 | -5.717780179 | 1.41E-05 | 0.000145015 | 3.101555152 |
| FAM35A | 1.291408505 | 7.983641398 | 5.717545307 | 1.41E-05 | 0.000145015 | 3.10103865 |
| PFKFB1 | -1.719424471 | 4.476108645 | -5.717395415 | 1.41E-05 | 0.000145015 | 3.100709022 |
| DIO2 | -1.943355986 | 4.959139394 | -5.715796914 | 1.42E-05 | 0.000145444 | 3.097193599 |
| PACRGL | 0.973441353 | 5.986953168 | 5.71420557 | 1.42E-05 | 0.000145822 | 3.093693644 |
| C1orf27 | 2.637618938 | 6.798072474 | 5.714097313 | 1.42E-05 | 0.000145822 | 3.093455536 |
| RNF24 | -1.173586152 | 9.954940864 | -5.713719589 | 1.42E-05 | 0.000145848 | 3.092624735 |
| FAM13B | 0.942726054 | 9.151909602 | 5.713490022 | 1.42E-05 | 0.000145848 | 3.092119799 |
| ZNF846 | 1.952447146 | 4.412677146 | 5.712520539 | 1.43E-05 | 0.000146076 | 3.089987336 |
| PRSS16 | 1.547177502 | 4.788571129 | 5.711564351 | 1.43E-05 | 0.0001463 | 3.087884016 |
| PPIG | 2.235264318 | 9.334467803 | 5.710218176 | 1.43E-05 | 0.000146651 | 3.084922677 |
| RECQL | 1.655087107 | 8.75786681 | 5.70966173 | 1.44E-05 | 0.000146747 | 3.083698542 |
| MEIS1 | -1.6806405 | 5.705222591 | -5.709284924 | 1.44E-05 | 0.000146784 | 3.08286958 |
| STK17B | 1.136716898 | 10.51163117 | 5.708044582 | 1.44E-05 | 0.000147102 | 3.080140758 |
| TP53INP1 | 1.627071653 | 8.91078931 | 5.707211619 | 1.44E-05 | 0.000147288 | 3.078308098 |
| MIOS | 0.797305591 | 9.731273009 | 5.706713667 | 1.44E-05 | 0.000147365 | 3.077212483 |
| PCP4 | -1.743791634 | 5.329580802 | -5.703819331 | 1.45E-05 | 0.000148226 | 3.070843717 |
| FZR1 | -0.920533133 | 9.360495724 | -5.702889676 | 1.46E-05 | 0.000148446 | 3.06879789 |
| NANOS3 | -1.053695277 | 6.060439817 | -5.700383716 | 1.47E-05 | 0.000149185 | 3.063282734 |
| RBBP5 | 1.859235434 | 4.776463976 | 5.699787996 | 1.47E-05 | 0.000149296 | 3.061971564 |
| NUP43 | 0.611008454 | 9.814963484 | 5.698910507 | 1.47E-05 | 0.0001495 | 3.060040157 |
| ZNF354A | 1.453720396 | 7.140534935 | 5.697427625 | 1.47E-05 | 0.000149905 | 3.056776054 |
| CHD7 | 0.979507524 | 7.505283714 | 5.694164848 | 1.49E-05 | 0.000150905 | 3.049593232 |
| LST1 | -0.958351082 | 14.61611292 | -5.692259293 | 1.49E-05 | 0.000151456 | 3.045397731 |
| SETD5 | -0.853374938 | 8.015261761 | -5.690983966 | 1.50E-05 | 0.000151797 | 3.042589597 |
| EXOC5 | 2.006515783 | 7.354471966 | 5.690228043 | 1.50E-05 | 0.000151964 | 3.040925055 |
| FOXN2 | 1.476441746 | 11.16563026 | 5.689457698 | 1.50E-05 | 0.000152135 | 3.03922869 |
| G3BP1 | 0.962424724 | 10.23937571 | 5.688342976 | 1.50E-05 | 0.000152424 | 3.036773868 |
| BORA | 1.49478438 | 7.094216586 | 5.687183288 | 1.51E-05 | 0.000152728 | 3.034219879 |
| WDR75 | 1.296342291 | 8.79922038 | 5.686182732 | 1.51E-05 | 0.000152979 | 3.032016233 |
| PRKRA | 0.945118917 | 8.464992533 | 5.682812663 | 1.52E-05 | 0.000154037 | 3.024593132 |
| ATP10D | 1.447881549 | 4.394118084 | 5.68085767 | 1.53E-05 | 0.000154618 | 3.0202864 |
| ATXN3 | 0.813478588 | 9.617880135 | 5.680398393 | 1.53E-05 | 0.000154686 | 3.01927458 |
| RNF149 | 0.844418155 | 13.23170988 | 5.680011248 | 1.53E-05 | 0.00015473 | 3.018421656 |
| RSL24D1 | 2.783816642 | 7.844576597 | 5.679454003 | 1.53E-05 | 0.000154816 | 3.017193953 |
| TAS2R30 | 1.86172857 | 7.785158681 | 5.679243376 | 1.54E-05 | 0.000154816 | 3.016729897 |
| MOV10L1 | -2.641118938 | 4.184591656 | -5.678610994 | 1.54E-05 | 0.000154944 | 3.015336603 |
| PLA2G12B | -2.213833643 | 3.53570299 | -5.677014743 | 1.54E-05 | 0.000155375 | 3.01181947 |
| TMEM117 | 1.30490614 | 5.558476091 | 5.676841286 | 1.54E-05 | 0.000155375 | 3.011437263 |
| CSTF2T | 0.685653072 | 8.843357139 | 5.675895665 | 1.55E-05 | 0.000155612 | 3.009353566 |
| RIN1 | -1.236774266 | 10.48750998 | -5.675371225 | 1.55E-05 | 0.000155681 | 3.008197909 |
| ZNF582 | 1.55941244 | 3.905369779 | 5.67498553 | 1.55E-05 | 0.000155681 | 3.007347972 |
| RAD1 | 0.80780933 | 6.510396494 | 5.674922582 | 1.55E-05 | 0.000155681 | 3.007209255 |
| RNF10 | -1.678966366 | 13.45508454 | -5.674283632 | 1.55E-05 | 0.000155813 | 3.005801193 |
| TANK | 0.661561369 | 11.19255171 | 5.672672495 | 1.56E-05 | 0.000156269 | 3.002250518 |
| TST | -1.262811759 | 13.43440559 | -5.672148641 | 1.56E-05 | 0.000156269 | 3.001095971 |
| RHCE | -2.450284374 | 6.540489305 | -5.671942058 | 1.56E-05 | 0.000156269 | 3.000640665 |
| SDCBP | 1.178971811 | 12.09557492 | 5.671938637 | 1.56E-05 | 0.000156269 | 3.000633125 |
| MAPK9 | 1.849597158 | 6.277493225 | 5.671642432 | 1.56E-05 | 0.000156283 | 2.999980287 |
| ANO10 | -0.997643134 | 9.929108643 | -5.667051871 | 1.58E-05 | 0.000157792 | 2.989861445 |
| ZNF592 | 0.628678798 | 7.700576051 | 5.664853017 | 1.59E-05 | 0.000158474 | 2.985013782 |
| SERPINI1 | 1.807010207 | 4.241941697 | 5.66393534 | 1.59E-05 | 0.000158706 | 2.982990489 |
| OXER1 | -1.249375826 | 11.71822064 | -5.661731265 | 1.60E-05 | 0.000159358 | 2.978130587 |
| ZNF449 | 1.752372855 | 6.278035573 | 5.661576419 | 1.60E-05 | 0.000159358 | 2.977789138 |
| NCOA2 | 1.558175015 | 5.115647646 | 5.660326172 | 1.60E-05 | 0.00015971 | 2.975032139 |
| UBA52 | -0.55389583 | 17.41121258 | -5.658446768 | 1.61E-05 | 0.000160287 | 2.970887438 |
| WTAP | 1.174212291 | 8.512108267 | 5.657811738 | 1.61E-05 | 0.000160422 | 2.969486905 |
| STT3B | 1.920156704 | 8.101642306 | 5.654519815 | 1.62E-05 | 0.000161507 | 2.962226013 |
| C7orf43 | -0.895251646 | 8.972983163 | -5.654247489 | 1.62E-05 | 0.000161513 | 2.961625301 |
| PRPF38B | 1.068113171 | 8.130551368 | 5.653874512 | 1.62E-05 | 0.000161556 | 2.960802554 |
| ILK | -0.703302948 | 11.5151772 | -5.650214701 | 1.64E-05 | 0.000162782 | 2.952728631 |
| PVRL1 | -1.191551827 | 5.449173055 | -5.649214579 | 1.64E-05 | 0.000163051 | 2.950522014 |
| FAM69A | 1.25682162 | 8.321429121 | 5.648401533 | 1.64E-05 | 0.000163197 | 2.948728071 |
| TSPAN9 | -1.337410517 | 3.445121117 | -5.648302772 | 1.64E-05 | 0.000163197 | 2.948510156 |
| 7-Sep | 1.686039585 | 9.637667945 | 5.64713942 | 1.65E-05 | 0.000163527 | 2.945943152 |
| SLC43A1 | -0.920143569 | 8.711297937 | -5.645930164 | 1.65E-05 | 0.000163874 | 2.943274704 |
| CDKN2AIPNL | 0.823449915 | 6.956825619 | 5.645228686 | 1.66E-05 | 0.000164037 | 2.941726695 |
| TAC3 | -1.664108615 | 5.518240489 | -5.644533907 | 1.66E-05 | 0.000164198 | 2.940193415 |
| THOC2 | 1.187868707 | 8.711808691 | 5.643938223 | 1.66E-05 | 0.000164222 | 2.938878784 |
| RAB14 | 0.922499603 | 6.637005971 | 5.643832397 | 1.66E-05 | 0.000164222 | 2.93864523 |
| PGM3 | 0.908668591 | 7.484273986 | 5.643709717 | 1.66E-05 | 0.000164222 | 2.938374479 |
| PRMT6 | 0.973584714 | 8.404577569 | 5.643279457 | 1.66E-05 | 0.000164286 | 2.937424893 |
| TMEM33 | 1.570499556 | 3.699917342 | 5.642948581 | 1.66E-05 | 0.000164315 | 2.936694634 |
| WSB2 | 0.769323782 | 10.72978492 | 5.642689421 | 1.67E-05 | 0.000164317 | 2.93612265 |
| FRMD3 | -0.874272524 | 7.304947271 | -5.639660454 | 1.68E-05 | 0.000165334 | 2.929436969 |
| VBP1 | 1.763129159 | 7.313172219 | 5.637305684 | 1.69E-05 | 0.000166108 | 2.924238743 |
| NDFIP1 | 0.857641539 | 10.81470819 | 5.636407286 | 1.69E-05 | 0.000166286 | 2.922255348 |
| DNAJC3 | 1.619963581 | 8.57668148 | 5.636318096 | 1.69E-05 | 0.000166286 | 2.922058439 |
| IVNS1ABP | 1.572354669 | 9.072725513 | 5.635689058 | 1.69E-05 | 0.000166426 | 2.920669655 |
| WNK4 | -2.541806457 | 7.604267913 | -5.635067377 | 1.69E-05 | 0.000166562 | 2.919297075 |
| HRH3 | -2.785853207 | 6.420152998 | -5.633860789 | 1.70E-05 | 0.000166916 | 2.916632985 |
| MFSD8 | 1.377416184 | 6.898100834 | 5.632621669 | 1.70E-05 | 0.000167283 | 2.91389691 |
| MXI1 | -1.61498874 | 10.9756684 | -5.632232961 | 1.70E-05 | 0.000167334 | 2.91303858 |
| FASN | -0.757472306 | 8.792284688 | -5.6315802 | 1.71E-05 | 0.000167484 | 2.91159714 |
| UBE2D4 | -0.934826047 | 9.979586836 | -5.631181508 | 1.71E-05 | 0.000167538 | 2.910716718 |
| ZNF41 | 1.719228762 | 5.192789174 | 5.630913998 | 1.71E-05 | 0.000167545 | 2.910125975 |
| PPIL1 | 0.907388245 | 9.264422406 | 5.629655399 | 1.71E-05 | 0.00016792 | 2.9073465 |
| CDC34 | -2.275941961 | 12.27344256 | -5.629091307 | 1.72E-05 | 0.000168037 | 2.906100712 |
| ZFYVE16 | 1.692989874 | 6.720653764 | 5.627318006 | 1.72E-05 | 0.000168573 | 2.902184189 |
| SPHKAP | -0.853481419 | 2.789166448 | -5.626738527 | 1.73E-05 | 0.000168573 | 2.900904277 |
| C3 | -1.065266662 | 6.346568802 | -5.626669819 | 1.73E-05 | 0.000168573 | 2.900752517 |
| FAM63A | -1.06925358 | 12.07801858 | -5.626659517 | 1.73E-05 | 0.000168573 | 2.900729763 |
| TNRC18 | -2.234996366 | 13.23102829 | -5.624489697 | 1.73E-05 | 0.000169294 | 2.89593689 |
| AHNAK | 1.037115361 | 11.62977468 | 5.623513467 | 1.74E-05 | 0.000169568 | 2.893780356 |
| CEP152 | 1.182750929 | 6.096649514 | 5.621221274 | 1.75E-05 | 0.000170339 | 2.88871641 |
| RASA2 | 1.632170439 | 6.998224306 | 5.62025648 | 1.75E-05 | 0.000170611 | 2.886584809 |
| CCDC66 | 1.438309601 | 6.099577121 | 5.619800272 | 1.75E-05 | 0.000170683 | 2.885576837 |
| ZCCHC7 | 1.24956286 | 7.010411834 | 5.619566711 | 1.75E-05 | 0.000170683 | 2.885060787 |
| C16orf86 | -0.866609122 | 7.141885426 | -5.61704704 | 1.76E-05 | 0.000171426 | 2.879493229 |
| ATP6V1C2 | 1.959587138 | 3.336700403 | 5.616868037 | 1.76E-05 | 0.000171426 | 2.879097672 |
| ANKRD13B | -2.760628221 | 7.88846526 | -5.616648059 | 1.76E-05 | 0.000171426 | 2.878611565 |
| CLIP2 | -0.968858749 | 6.952845167 | -5.616532515 | 1.76E-05 | 0.000171426 | 2.878356234 |
| C5orf34 | 1.511356307 | 3.401297357 | 5.616317687 | 1.77E-05 | 0.000171426 | 2.877881501 |
| THEMIS | 2.186607052 | 8.411977687 | 5.616122267 | 1.77E-05 | 0.000171426 | 2.877449652 |
| YWHAZ | 1.1619217 | 12.02052523 | 5.615569013 | 1.77E-05 | 0.000171543 | 2.876227019 |
| ITSN1 | -1.381694591 | 5.909029158 | -5.615020057 | 1.77E-05 | 0.000171658 | 2.875013854 |
| KIFC2 | -1.043543783 | 8.157373933 | -5.613737643 | 1.78E-05 | 0.000172053 | 2.872179663 |
| ZNF720 | 1.251136282 | 6.628865769 | 5.61162669 | 1.78E-05 | 0.000172768 | 2.86751399 |
| TBCD | -0.887460202 | 9.489793024 | -5.611211464 | 1.79E-05 | 0.000172832 | 2.866596196 |
| BCL2 | 1.196150625 | 9.374415032 | 5.610516945 | 1.79E-05 | 0.000173004 | 2.865061024 |
| LSM14A | 0.905906811 | 9.572477111 | 5.609464322 | 1.79E-05 | 0.000173314 | 2.862734203 |
| MDH1 | 0.879418541 | 12.62836186 | 5.608473754 | 1.80E-05 | 0.000173601 | 2.860544448 |
| HBS1L | 1.462693733 | 6.465821928 | 5.607809005 | 1.80E-05 | 0.000173763 | 2.859074893 |
| FUZ | -1.00118071 | 8.38211143 | -5.6052064 | 1.81E-05 | 0.000174654 | 2.853320899 |
| SMN2 | 2.084912504 | 4.054211644 | 5.605017283 | 1.81E-05 | 0.000174654 | 2.852902762 |
| PTPN12 | 1.420622371 | 10.56844458 | 5.604607789 | 1.81E-05 | 0.000174717 | 2.851997358 |
| FBRS | -0.994261799 | 13.20232967 | -5.602790724 | 1.82E-05 | 0.000175329 | 2.847979554 |
| BRIP1 | 1.734241011 | 2.938715142 | 5.601761414 | 1.82E-05 | 0.000175635 | 2.845703445 |
| C16orf87 | 1.685855858 | 7.687108948 | 5.601163183 | 1.83E-05 | 0.000175773 | 2.84438053 |
| LRTOMT | -0.81059443 | 5.754877781 | -5.598235351 | 1.84E-05 | 0.00017674 | 2.837905448 |
| ADCY7 | 1.326178481 | 9.846943679 | 5.598206305 | 1.84E-05 | 0.00017674 | 2.837841205 |
| PRR5-ARHGAP8 | -1.696637309 | 7.234077354 | -5.597478639 | 1.84E-05 | 0.000176874 | 2.836231788 |
| HIST2H3A | 3.751807014 | 7.098890409 | 5.597373977 | 1.84E-05 | 0.000176874 | 2.836000298 |
| PDE1B | -0.811762417 | 7.915070087 | -5.596164529 | 1.85E-05 | 0.000177254 | 2.833325164 |
| ARF4 | 0.841953822 | 10.58917976 | 5.593696315 | 1.86E-05 | 0.000178134 | 2.827865344 |
| DCAF5 | 0.745134981 | 7.799155066 | 5.590189587 | 1.87E-05 | 0.000179433 | 2.820107201 |
| ZNF816 | 1.54986082 | 8.201073302 | 5.586797439 | 1.89E-05 | 0.000180695 | 2.812601348 |
| C1QTNF5 | -2.016941259 | 6.855552079 | -5.586197431 | 1.89E-05 | 0.000180838 | 2.811273581 |
| GALT | -0.680474908 | 9.009545612 | -5.580507931 | 1.91E-05 | 0.000183019 | 2.798681355 |
| NFKBIA | 0.677901765 | 13.24892577 | 5.580277571 | 1.91E-05 | 0.000183019 | 2.798171445 |
| FOXH1 | -3.712907719 | 8.484546944 | -5.580083291 | 1.91E-05 | 0.000183019 | 2.797741393 |
| DDX49 | -0.823469444 | 9.367241457 | -5.57746009 | 1.93E-05 | 0.000183902 | 2.791934394 |
| IQCF5 | -1.647394444 | 3.721918137 | -5.577434835 | 1.93E-05 | 0.000183902 | 2.791878483 |
| DLG1 | 2.12407602 | 7.707008275 | 5.577157578 | 1.93E-05 | 0.000183916 | 2.791264676 |
| AP3D1 | -1.020288363 | 9.798443144 | -5.574607159 | 1.94E-05 | 0.000184864 | 2.785618044 |
| UTF1 | -1.10877658 | 10.09532492 | -5.573432187 | 1.94E-05 | 0.000185248 | 2.78301643 |
| ZNF439 | 1.92577246 | 6.780780832 | 5.572192512 | 1.95E-05 | 0.00018566 | 2.780271401 |
| FAM73B | -1.074171855 | 8.739396544 | -5.570506856 | 1.96E-05 | 0.000186258 | 2.776538578 |
| NT5C1A | -3.554777515 | 5.054421936 | -5.570196122 | 1.96E-05 | 0.000186286 | 2.775850438 |
| SEC23IP | 0.889040684 | 9.706559609 | 5.569150434 | 1.96E-05 | 0.00018662 | 2.773534622 |
| UMOD | -1.444126363 | 7.439984363 | -5.567294031 | 1.97E-05 | 0.000187292 | 2.769423096 |
| ATCAY | -1.470718701 | 2.99709795 | -5.565558734 | 1.98E-05 | 0.000187917 | 2.765579479 |
| OR2T1 | -0.987320286 | 4.373401505 | -5.564469084 | 1.98E-05 | 0.000188272 | 2.763165788 |
| NAPG | 1.03920661 | 9.318511023 | 5.562407526 | 1.99E-05 | 0.000188966 | 2.758598891 |
| TTC9 | 1.203423011 | 2.885315562 | 5.562334369 | 1.99E-05 | 0.000188966 | 2.758436821 |
| SFR1 | 1.221883024 | 7.276533304 | 5.560167365 | 2.00E-05 | 0.000189779 | 2.75363585 |
| DNAJC15 | 1.235200151 | 7.897194533 | 5.558343913 | 2.01E-05 | 0.000190404 | 2.74959565 |
| OLFML2B | -3.29427453 | 8.017514817 | -5.558211164 | 2.01E-05 | 0.000190404 | 2.749301506 |
| MAGEB5 | -1.947200867 | 5.111020924 | -5.557963416 | 2.01E-05 | 0.000190406 | 2.748752545 |
| RHOT1 | 1.332920734 | 6.693465368 | 5.556111194 | 2.02E-05 | 0.000191092 | 2.744648183 |
| PNMA5 | -1.73637151 | 9.41502414 | -5.554753624 | 2.03E-05 | 0.000191568 | 2.741639706 |
| MUSTN1 | -0.888988656 | 8.597029526 | -5.553232123 | 2.03E-05 | 0.000192116 | 2.738267727 |
| MRPL2 | -0.822179903 | 7.619452905 | -5.5512973 | 2.04E-05 | 0.000192844 | 2.733979398 |
| C9orf9 | -0.902661613 | 5.740039945 | -5.550846734 | 2.04E-05 | 0.000192934 | 2.732980711 |
| CAMK2D | 1.392428009 | 8.056079284 | 5.550444555 | 2.04E-05 | 0.000193003 | 2.732089261 |
| ACSL4 | 1.471807201 | 7.951589777 | 5.550004554 | 2.05E-05 | 0.000193089 | 2.731113955 |
| CRYBG3 | 1.854488137 | 6.458438965 | 5.549623736 | 2.05E-05 | 0.00019315 | 2.730269818 |
| CRLS1 | 1.046745261 | 9.489844859 | 5.548006485 | 2.06E-05 | 0.000193744 | 2.726684792 |
| ENO3 | -0.90716597 | 10.30516863 | -5.54490779 | 2.07E-05 | 0.000194984 | 2.719815055 |
| CCDC57 | -0.788555488 | 8.569598483 | -5.543996356 | 2.07E-05 | 0.000195277 | 2.717794244 |
| RNF180 | -1.718046715 | 9.031012304 | -5.541668615 | 2.09E-05 | 0.000196189 | 2.712632848 |
| MYOM1 | -0.999786599 | 8.208628302 | -5.541161742 | 2.09E-05 | 0.000196306 | 2.711508867 |
| SPHK2 | -0.696008983 | 10.75194403 | -5.540757253 | 2.09E-05 | 0.000196379 | 2.7106119 |
| OTOA | -4.630326459 | 7.778063988 | -5.539512086 | 2.10E-05 | 0.000196773 | 2.707850605 |
| DDX46 | 1.117093184 | 7.331507073 | 5.539182453 | 2.10E-05 | 0.000196773 | 2.707119581 |
| RIC3 | 2.102645351 | 4.867735819 | 5.539139635 | 2.10E-05 | 0.000196773 | 2.707024623 |
| ZNF236 | 1.220833638 | 5.480937936 | 5.537370664 | 2.11E-05 | 0.000197447 | 2.703101402 |
| BRCC3 | 1.007923617 | 8.726386966 | 5.537017693 | 2.11E-05 | 0.000197497 | 2.702318545 |
| PDE8A | 1.034227972 | 7.73631678 | 5.535071808 | 2.12E-05 | 0.000198251 | 2.698002534 |
| ITGA2B | -1.530303894 | 7.839596883 | -5.534815895 | 2.12E-05 | 0.000198259 | 2.697434885 |
| ZNF701 | 1.683836518 | 8.127887207 | 5.534514156 | 2.12E-05 | 0.000198287 | 2.696765581 |
| RNF2 | 1.37336393 | 6.68073148 | 5.532750306 | 2.13E-05 | 0.000198964 | 2.692852903 |
| COX6C | 2.08040355 | 11.8975225 | 5.531612393 | 2.13E-05 | 0.00019925 | 2.690328552 |
| MS4A7 | 1.306847194 | 10.67405149 | 5.531491137 | 2.13E-05 | 0.00019925 | 2.690059548 |
| DMXL2 | 1.893083514 | 6.275081076 | 5.531392746 | 2.13E-05 | 0.00019925 | 2.689841271 |
| NAB1 | 1.260064841 | 8.052207062 | 5.531109552 | 2.13E-05 | 0.00019927 | 2.689213005 |
| PGAP2 | -0.909347238 | 9.012300296 | -5.529563377 | 2.14E-05 | 0.000199854 | 2.685782677 |
| PIGF | 1.115687235 | 8.754371728 | 5.528801955 | 2.15E-05 | 0.000200088 | 2.684093308 |
| UHRF2 | 1.487349651 | 8.717190658 | 5.526345864 | 2.16E-05 | 0.000201048 | 2.678643578 |
| NFE2L3 | 1.195474762 | 7.644372489 | 5.52618367 | 2.16E-05 | 0.000201048 | 2.678283669 |
| PLAG1 | 1.63320614 | 7.693007037 | 5.525719167 | 2.16E-05 | 0.00020115 | 2.677252926 |
| FPGT | 1.149828795 | 7.120859086 | 5.524938407 | 2.16E-05 | 0.000201354 | 2.675520351 |
| CHCHD7 | 0.861771193 | 10.0524939 | 5.524792686 | 2.17E-05 | 0.000201354 | 2.675196978 |
| GRB10 | -0.994915323 | 8.246995302 | -5.523443033 | 2.17E-05 | 0.000201855 | 2.672201819 |
| ZNF267 | 2.376019133 | 9.06366875 | 5.521098666 | 2.18E-05 | 0.000202709 | 2.666998752 |
| SNX15 | -0.934594466 | 7.931135038 | -5.521080753 | 2.18E-05 | 0.000202709 | 2.666958995 |
| TGFB1I1 | -1.472656073 | 8.946218332 | -5.518912563 | 2.19E-05 | 0.000203586 | 2.662146447 |
| ANKRD42 | 1.100876007 | 12.66323296 | 5.51740164 | 2.20E-05 | 0.000204167 | 2.658792505 |
| NUDT21 | 0.817506731 | 8.749524602 | 5.516926687 | 2.20E-05 | 0.000204276 | 2.657738159 |
| ZNF570 | 1.4093237 | 6.093082727 | 5.515566249 | 2.21E-05 | 0.000204574 | 2.654718004 |
| CEBPZ | 1.630278252 | 9.146199114 | 5.515546217 | 2.21E-05 | 0.000204574 | 2.654673532 |
| TSPAN1 | -2.151571248 | 7.652154406 | -5.515348712 | 2.21E-05 | 0.000204574 | 2.654235059 |
| CYP4V2 | 1.960639726 | 5.091199168 | 5.515333298 | 2.21E-05 | 0.000204574 | 2.654200838 |
| NUP205 | 0.691173996 | 11.35263195 | 5.514481096 | 2.22E-05 | 0.000204856 | 2.652308849 |
| KBTBD2 | 1.254498081 | 7.874485371 | 5.513232071 | 2.22E-05 | 0.000205263 | 2.649535735 |
| OSBPL11 | 1.17456107 | 9.45771158 | 5.513123284 | 2.22E-05 | 0.000205263 | 2.649294196 |
| FRA10AC1 | 1.296295508 | 6.346878721 | 5.512552926 | 2.23E-05 | 0.000205417 | 2.648027818 |
| CASK | 1.010896146 | 6.66437807 | 5.511643108 | 2.23E-05 | 0.000205727 | 2.646007662 |
| CACNB4 | 1.944296337 | 3.554404958 | 5.508424112 | 2.25E-05 | 0.000207104 | 2.63885956 |
| POGLUT1 | 0.871412896 | 8.390515024 | 5.507902555 | 2.25E-05 | 0.000207232 | 2.637701295 |
| ZNF653 | -0.733480632 | 7.054932162 | -5.50768032 | 2.25E-05 | 0.000207232 | 2.637207752 |
| B3GALT2 | 1.957602909 | 3.785859762 | 5.506879292 | 2.25E-05 | 0.000207494 | 2.635428772 |
| TGFBR1 | 1.144358114 | 6.864588302 | 5.506324373 | 2.26E-05 | 0.000207627 | 2.634196332 |
| MS4A1 | 1.449270091 | 9.932317931 | 5.506124388 | 2.26E-05 | 0.000207627 | 2.63375217 |
| VASH1 | -1.330144988 | 11.17566232 | -5.504614012 | 2.27E-05 | 0.000208221 | 2.630397537 |
| LRRC58 | 1.662762723 | 6.205656866 | 5.503938971 | 2.27E-05 | 0.000208427 | 2.628898159 |
| ZNF383 | 1.383153865 | 6.542355417 | 5.50233339 | 2.28E-05 | 0.000208977 | 2.625331717 |
| TPPP3 | -1.858335939 | 8.021493384 | -5.502293858 | 2.28E-05 | 0.000208977 | 2.625243904 |
| MGAT2 | 1.059540534 | 9.220925559 | 5.501900218 | 2.28E-05 | 0.000209051 | 2.624369479 |
| RNF170 | 1.080105772 | 7.403665954 | 5.500824946 | 2.28E-05 | 0.000209431 | 2.621980816 |
| GXYLT1 | 1.635552213 | 4.734917498 | 5.50062269 | 2.29E-05 | 0.000209431 | 2.6215315 |
| MYO6 | 1.303812066 | 5.477914843 | 5.500168868 | 2.29E-05 | 0.000209474 | 2.620523314 |
| DCUN1D5 | 1.272338944 | 9.181675371 | 5.500064567 | 2.29E-05 | 0.000209474 | 2.620291601 |
| GPN2 | -0.659721631 | 10.30779817 | -5.496029841 | 2.31E-05 | 0.000211209 | 2.611327343 |
| SERPINA6 | -2.376158867 | 7.51121392 | -5.495912503 | 2.31E-05 | 0.000211209 | 2.611066622 |
| FBXL19 | -1.755434564 | 8.800247527 | -5.49453996 | 2.32E-05 | 0.000211749 | 2.608016759 |
| SH3RF1 | 1.573717533 | 5.430975119 | 5.493472162 | 2.32E-05 | 0.000212145 | 2.605643928 |
| ITGB1 | 1.826561546 | 8.384668166 | 5.491882426 | 2.33E-05 | 0.000212696 | 2.602111056 |
| RBM33 | 1.733917058 | 6.570420818 | 5.491848536 | 2.33E-05 | 0.000212696 | 2.60203574 |
| VAMP1 | 1.659019064 | 5.178039338 | 5.491256904 | 2.33E-05 | 0.000212777 | 2.600720891 |
| TTC32 | 0.996398103 | 9.024804498 | 5.491214607 | 2.33E-05 | 0.000212777 | 2.600626889 |
| ARHGAP15 | 0.958588325 | 10.89628062 | 5.490666573 | 2.34E-05 | 0.000212928 | 2.599408899 |
| MRPS31 | 1.110772235 | 10.62014714 | 5.489589854 | 2.34E-05 | 0.000213331 | 2.597015837 |
| RBM41 | 0.7697985 | 8.007279128 | 5.488697859 | 2.35E-05 | 0.000213548 | 2.595033249 |
| PLA2G2C | -1.628531873 | 6.235593988 | -5.488672261 | 2.35E-05 | 0.000213548 | 2.594976353 |
| GPD2 | 1.059751622 | 6.92473892 | 5.486840735 | 2.36E-05 | 0.000214315 | 2.590905271 |
| SPTLC1 | 1.250134805 | 7.791153451 | 5.484424387 | 2.37E-05 | 0.000215365 | 2.585533761 |
| TWF1 | 1.332153196 | 9.008490648 | 5.481823764 | 2.38E-05 | 0.000216412 | 2.579751975 |
| RNF40 | -0.989124718 | 8.180839718 | -5.481797951 | 2.38E-05 | 0.000216412 | 2.579694586 |
| DUSP26 | 3.19448746 | 5.162177995 | 5.479171382 | 2.40E-05 | 0.000217576 | 2.573854444 |
| LTV1 | 1.23960318 | 9.237639989 | 5.478312498 | 2.40E-05 | 0.000217784 | 2.571944581 |
| RAB11A | 1.398095647 | 7.11410913 | 5.478283499 | 2.40E-05 | 0.000217784 | 2.571880095 |
| AASDH | 1.26291393 | 6.612943581 | 5.477794971 | 2.41E-05 | 0.00021786 | 2.570793745 |
| SLC48A1 | -1.202671194 | 10.70324876 | -5.477667397 | 2.41E-05 | 0.00021786 | 2.570510051 |
| FOLR2 | -1.211044618 | 8.513278814 | -5.475876417 | 2.42E-05 | 0.000218623 | 2.566527187 |
| OR7A10 | -1.359213134 | 3.692835904 | -5.475641236 | 2.42E-05 | 0.000218626 | 2.566004157 |
| TXNDC9 | 1.583303129 | 8.653517904 | 5.473249083 | 2.43E-05 | 0.000219687 | 2.560683839 |
| ADIPOR1 | -1.966745598 | 12.97078694 | -5.472959246 | 2.43E-05 | 0.000219717 | 2.560039184 |
| GINS1 | 1.055960749 | 4.602187378 | 5.47105036 | 2.44E-05 | 0.000220545 | 2.555793235 |
| CASC4 | 1.202356644 | 8.098437406 | 5.468894522 | 2.45E-05 | 0.000221365 | 2.550997568 |
| IFT81 | 1.515825836 | 3.234772568 | 5.468850362 | 2.45E-05 | 0.000221365 | 2.550899329 |
| SLC37A3 | 1.081339863 | 9.590444273 | 5.468706135 | 2.45E-05 | 0.000221365 | 2.550578478 |
| SEC14L3 | -2.14296167 | 8.550925404 | -5.468164008 | 2.46E-05 | 0.00022152 | 2.549372436 |
| SGPP1 | 1.760747452 | 6.617878658 | 5.467798754 | 2.46E-05 | 0.000221588 | 2.548559857 |
| SGCB | 1.308337764 | 6.150599223 | 5.467483127 | 2.46E-05 | 0.000221631 | 2.547857672 |
| DCAF12 | -2.07387109 | 12.50510268 | -5.46641952 | 2.47E-05 | 0.000222046 | 2.545491366 |
| CCL16 | -1.161017518 | 8.94122595 | -5.465670265 | 2.47E-05 | 0.000222305 | 2.543824364 |
| NBEA | 1.759732248 | 4.207492039 | 5.464971885 | 2.48E-05 | 0.00022254 | 2.542270502 |
| CAPN2 | 0.712186635 | 13.8950382 | 5.463166807 | 2.49E-05 | 0.000223327 | 2.538254076 |
| MEF2C | 1.06470224 | 10.3589535 | 5.461391938 | 2.50E-05 | 0.000224103 | 2.534304562 |
| YRDC | 0.719152295 | 10.3184689 | 5.460731476 | 2.50E-05 | 0.00022432 | 2.532834798 |
| UTP15 | 1.187762817 | 5.95866456 | 5.460134823 | 2.50E-05 | 0.000224506 | 2.531506995 |
| MFSD2B | -1.310120822 | 5.967269264 | -5.459618187 | 2.51E-05 | 0.000224651 | 2.530357234 |
| DCT | -4.435878746 | 8.039121221 | -5.458367108 | 2.51E-05 | 0.000225167 | 2.527572886 |
| MYH11 | 1.256999301 | 3.075091693 | 5.458109258 | 2.51E-05 | 0.000225182 | 2.526999008 |
| ARFRP1 | -0.920328648 | 10.37189265 | -5.457132369 | 2.52E-05 | 0.000225561 | 2.524824757 |
| CLINT1 | 0.77267125 | 10.64871516 | 5.452919497 | 2.54E-05 | 0.000227585 | 2.515447168 |
| ZNF160 | 1.203602148 | 6.976758439 | 5.452276337 | 2.55E-05 | 0.000227798 | 2.514015386 |
| SLC8A3 | -1.674560378 | 4.652332784 | -5.451868597 | 2.55E-05 | 0.000227852 | 2.513107669 |
| TSPAN4 | -1.149814439 | 9.436174987 | -5.451716083 | 2.55E-05 | 0.000227852 | 2.512768134 |
| NPAT | 1.469219205 | 6.508862492 | 5.450879267 | 2.55E-05 | 0.000228164 | 2.510905136 |
| UGCG | 1.20666731 | 9.124701213 | 5.448831572 | 2.57E-05 | 0.000229098 | 2.506346086 |
| CHFR | 0.742894281 | 8.283035205 | 5.447735061 | 2.57E-05 | 0.000229545 | 2.503904618 |
| ARMC10 | 0.626842732 | 8.884515782 | 5.447018749 | 2.58E-05 | 0.000229798 | 2.502309629 |
| RABEP1 | 0.941485702 | 7.997230247 | 5.445337632 | 2.59E-05 | 0.000230492 | 2.498566151 |
| RAB5A | 0.938154632 | 9.689876907 | 5.445220308 | 2.59E-05 | 0.000230492 | 2.498304887 |
| ZFP14 | 1.23198489 | 5.775477435 | 5.444403281 | 2.59E-05 | 0.000230798 | 2.49648544 |
| TRAF3 | 1.308787303 | 6.099586026 | 5.443857176 | 2.60E-05 | 0.000230964 | 2.495269279 |
| ZNHIT6 | 0.784728603 | 8.281117526 | 5.443411937 | 2.60E-05 | 0.00023098 | 2.494277721 |
| YWHAQ | 0.834074277 | 11.66115815 | 5.443376113 | 2.60E-05 | 0.00023098 | 2.494197941 |
| SLC25A45 | -1.079844243 | 8.148837565 | -5.442561862 | 2.60E-05 | 0.000231285 | 2.492384534 |
| ZNF732 | 2.577857772 | 6.796567487 | 5.442182588 | 2.61E-05 | 0.000231365 | 2.491539837 |
| CNOT3 | -0.695797285 | 11.3257111 | -5.441781827 | 2.61E-05 | 0.000231456 | 2.490647271 |
| PIGM | 2.123248943 | 5.083800337 | 5.439993512 | 2.62E-05 | 0.000232269 | 2.48666419 |
| ALG13 | 1.176634493 | 9.389500191 | 5.4396803 | 2.62E-05 | 0.000232315 | 2.485966547 |
| MRPL12 | 0.934832769 | 8.763799892 | 5.439249078 | 2.62E-05 | 0.000232353 | 2.485006034 |
| PVRL3 | 1.250014249 | 4.600475741 | 5.438965477 | 2.62E-05 | 0.000232353 | 2.484374328 |
| TCAP | -0.782676503 | 8.933882249 | -5.438933204 | 2.62E-05 | 0.000232353 | 2.484302441 |
| SLC13A2 | -1.246395777 | 5.953151712 | -5.438682091 | 2.63E-05 | 0.000232367 | 2.483743092 |
| SLC6A18 | -2.290266111 | 6.652761075 | -5.436425508 | 2.64E-05 | 0.000233429 | 2.478716332 |
| GTPBP10 | 1.185958477 | 4.727143822 | 5.43592786 | 2.64E-05 | 0.000233572 | 2.477607708 |
| RANBP10 | -1.209936002 | 11.20797865 | -5.435534706 | 2.64E-05 | 0.000233661 | 2.47673185 |
| GNB1 | 1.147398481 | 11.27622362 | 5.434452527 | 2.65E-05 | 0.000233994 | 2.474320929 |
| RAB1A | 0.821911407 | 10.2885004 | 5.434451427 | 2.65E-05 | 0.000233994 | 2.474318478 |
| GAB1 | 2.00788455 | 4.195876895 | 5.432541382 | 2.66E-05 | 0.000234792 | 2.470062934 |
| CLTCL1 | -1.504968653 | 7.076604947 | -5.432486507 | 2.66E-05 | 0.000234792 | 2.469940669 |
| ENTPD5 | -1.124093875 | 5.333199561 | -5.429795422 | 2.68E-05 | 0.000236096 | 2.463944385 |
| NUDT12 | 1.952114448 | 3.679703724 | 5.427826372 | 2.69E-05 | 0.000237023 | 2.459556509 |
| ADCY2 | -2.771153769 | 4.155725758 | -5.426837968 | 2.70E-05 | 0.00023743 | 2.457353789 |
| ZNF17 | 1.067593583 | 7.577386835 | 5.426592185 | 2.70E-05 | 0.000237436 | 2.456806031 |
| KCTD6 | 0.749661271 | 8.169081327 | 5.426380868 | 2.70E-05 | 0.000237436 | 2.456335082 |
| ZNF146 | 2.108254434 | 7.099164127 | 5.426089746 | 2.70E-05 | 0.000237473 | 2.455686269 |
| MMP19 | -2.133311575 | 7.78685378 | -5.425841855 | 2.70E-05 | 0.000237486 | 2.455133797 |
| DOCK7 | 1.504762599 | 5.730952727 | 5.425111597 | 2.71E-05 | 0.000237654 | 2.453506245 |
| KLHDC10 | 1.517567454 | 5.486889704 | 5.425081758 | 2.71E-05 | 0.000237654 | 2.453439741 |
| RRM2B | 1.434099185 | 6.795105626 | 5.424391386 | 2.71E-05 | 0.000237904 | 2.451901035 |
| CUX1 | -0.882189851 | 9.451077986 | -5.423307903 | 2.72E-05 | 0.000238364 | 2.449486073 |
| TIGD3 | -1.617034591 | 8.386849906 | -5.422045634 | 2.73E-05 | 0.000238922 | 2.446672475 |
| EFNA2 | -1.680719833 | 5.873477839 | -5.421774641 | 2.73E-05 | 0.000238948 | 2.446068413 |
| TPR | 1.103244873 | 9.892290073 | 5.421070395 | 2.73E-05 | 0.000239207 | 2.444498566 |
| SFT2D3 | 1.233345517 | 7.420912094 | 5.417934875 | 2.75E-05 | 0.000240777 | 2.437508565 |
| TNNT3 | -1.390005884 | 6.659763781 | -5.417668216 | 2.75E-05 | 0.000240801 | 2.436914061 |
| CCNL1 | 1.310032242 | 10.54600295 | 5.416535222 | 2.76E-05 | 0.000241295 | 2.434388028 |
| COL11A2 | -2.590794248 | 7.978632403 | -5.414560255 | 2.77E-05 | 0.000242223 | 2.429984512 |
| RAB5C | -0.791185695 | 8.31083193 | -5.414351399 | 2.77E-05 | 0.000242223 | 2.429518811 |
| ZNF780A | 1.214165389 | 6.424406563 | 5.4140218 | 2.78E-05 | 0.000242223 | 2.428783874 |
| C19orf33 | -1.822619975 | 9.619379622 | -5.413878009 | 2.78E-05 | 0.000242223 | 2.428463246 |
| SYF2 | -0.810055593 | 7.754433525 | -5.413719669 | 2.78E-05 | 0.000242223 | 2.428110176 |
| PRTFDC1 | -1.111179263 | 4.967184432 | -5.412468842 | 2.78E-05 | 0.000242748 | 2.425320964 |
| SMC4 | 1.523607801 | 8.267134158 | 5.412313946 | 2.79E-05 | 0.000242748 | 2.424975552 |
| JMJD1C | 1.607193899 | 8.368003697 | 5.411637671 | 2.79E-05 | 0.000242996 | 2.42346746 |
| TEAD3 | -1.551621586 | 6.153724657 | -5.410480054 | 2.80E-05 | 0.000243508 | 2.420885876 |
| AADAC | -0.973743501 | 2.621282992 | -5.410136498 | 2.80E-05 | 0.000243576 | 2.420119693 |
| ZNF346 | -0.833587711 | 9.777605122 | -5.408789065 | 2.81E-05 | 0.000244193 | 2.417114604 |
| PLEC | -0.952095395 | 9.540141264 | -5.408265747 | 2.81E-05 | 0.00024436 | 2.415947438 |
| MUC5AC | -1.56396152 | 5.94440864 | -5.407390995 | 2.82E-05 | 0.000244719 | 2.413996405 |
| MAT2B | 1.318281321 | 9.135996099 | 5.406110373 | 2.82E-05 | 0.000245303 | 2.411139997 |
| ATOX1 | -0.648171115 | 11.34054498 | -5.402932115 | 2.85E-05 | 0.000246939 | 2.404050284 |
| MRFAP1L1 | 1.412773934 | 10.37864919 | 5.402669398 | 2.85E-05 | 0.000246963 | 2.403464203 |
| TSTD2 | 0.871991186 | 6.183574475 | 5.40183825 | 2.85E-05 | 0.000247303 | 2.401609992 |
| PLTP | -1.493743894 | 6.889128068 | -5.400237292 | 2.86E-05 | 0.000248072 | 2.398038233 |
| SORCS2 | -1.249436806 | 4.287732839 | -5.39929404 | 2.87E-05 | 0.000248476 | 2.395933713 |
| MAP7D2 | -1.59617927 | 4.962652676 | -5.398696585 | 2.87E-05 | 0.000248687 | 2.394600671 |
| ZNF749 | 2.340243665 | 3.478812941 | 5.398359722 | 2.87E-05 | 0.000248702 | 2.393849047 |
| METTL2B | 1.229895618 | 8.283010081 | 5.398232331 | 2.88E-05 | 0.000248702 | 2.393564803 |
| ZNF418 | -3.344121269 | 9.868878967 | -5.397649908 | 2.88E-05 | 0.000248905 | 2.392265242 |
| RIMS1 | -1.333848482 | 4.174283522 | -5.397246075 | 2.88E-05 | 0.000249009 | 2.391364151 |
| TLR7 | 0.80108186 | 9.525358725 | 5.396481992 | 2.89E-05 | 0.000249314 | 2.389659177 |
| LRRK2 | 1.156307349 | 9.400394992 | 5.396082995 | 2.89E-05 | 0.000249415 | 2.388768835 |
| HADH | 0.802313967 | 10.51200938 | 5.39582673 | 2.89E-05 | 0.000249437 | 2.388196982 |
| IKBKAP | 1.068326735 | 5.858342896 | 5.395436986 | 2.89E-05 | 0.000249533 | 2.387327263 |
| TPM1 | -1.222976374 | 7.871530691 | -5.393176468 | 2.91E-05 | 0.000250681 | 2.382282612 |
| ERBB3 | -1.917673714 | 6.359944073 | -5.392891258 | 2.91E-05 | 0.000250719 | 2.381646095 |
| WDHD1 | 1.352613789 | 5.678051558 | 5.391292907 | 2.92E-05 | 0.000251498 | 2.378078832 |
| APOL1 | -1.311284498 | 11.5097602 | -5.390848259 | 2.92E-05 | 0.000251596 | 2.377086407 |
| RPL17 | 2.256862388 | 13.19435425 | 5.390683609 | 2.92E-05 | 0.000251596 | 2.376718915 |
| C16orf13 | -0.938107078 | 12.24669256 | -5.390466733 | 2.93E-05 | 0.000251596 | 2.376234852 |
| ZNF354C | 1.91593644 | 3.034663422 | 5.389832117 | 2.93E-05 | 0.000251832 | 2.374818378 |
| PBX1 | -1.712510787 | 5.654430384 | -5.38895237 | 2.94E-05 | 0.000252208 | 2.372854706 |
| SYT15 | -2.389480343 | 7.322108974 | -5.388532232 | 2.94E-05 | 0.000252323 | 2.371916895 |
| TRA2A | 2.168827062 | 5.656286899 | 5.387505951 | 2.95E-05 | 0.000252782 | 2.369626019 |
| GPR78 | -3.450720418 | 6.050956084 | -5.386886079 | 2.95E-05 | 0.000253011 | 2.368242283 |
| CORO2B | -1.281282059 | 6.573912312 | -5.386366196 | 2.95E-05 | 0.000253184 | 2.367081728 |
| SRPRB | 0.643942876 | 10.11726707 | 5.386017414 | 2.96E-05 | 0.000253204 | 2.366303113 |
| CYLD | 0.952858067 | 9.32822383 | 5.385897434 | 2.96E-05 | 0.000253204 | 2.36603527 |
| NUP62CL | 1.719212423 | 3.398058571 | 5.384658582 | 2.96E-05 | 0.000253786 | 2.363269578 |
| LCE2B | -1.952673622 | 4.156188071 | -5.381476662 | 2.99E-05 | 0.000255485 | 2.356165409 |
| ESPN | -1.709843683 | 6.910568304 | -5.380613604 | 2.99E-05 | 0.000255857 | 2.354238328 |
| SEC61A2 | 1.079568684 | 5.504404591 | 5.379981831 | 3.00E-05 | 0.000256096 | 2.352827629 |
| SLU7 | 1.056732525 | 9.973439857 | 5.379148036 | 3.00E-05 | 0.000256452 | 2.350965777 |
| MTM1 | 1.406578595 | 4.93283553 | 5.377361234 | 3.01E-05 | 0.00025736 | 2.346975658 |
| FKBP14 | 0.978913896 | 6.488873028 | 5.376057178 | 3.02E-05 | 0.000257991 | 2.344063377 |
| SLC39A10 | 1.532747729 | 6.37475119 | 5.375167515 | 3.03E-05 | 0.000258382 | 2.342076452 |
| TAF9B | 1.484636531 | 6.278665591 | 5.374536591 | 3.03E-05 | 0.000258624 | 2.340667335 |
| KIF20B | 2.504363727 | 5.681298367 | 5.370588654 | 3.06E-05 | 0.000260805 | 2.331849125 |
| CARD8 | 0.837429629 | 10.19837964 | 5.370116323 | 3.06E-05 | 0.000260849 | 2.33079402 |
| GSTCD | 1.031107194 | 4.597176508 | 5.370082452 | 3.06E-05 | 0.000260849 | 2.330718358 |
| FOXJ2 | -1.212925853 | 7.537693733 | -5.367203315 | 3.08E-05 | 0.000262418 | 2.324286424 |
| AGK | 1.255309675 | 7.008573699 | 5.366180645 | 3.09E-05 | 0.000262895 | 2.322001618 |
| SAMSN1 | 1.412715843 | 8.846660574 | 5.365610497 | 3.09E-05 | 0.000263105 | 2.320727776 |
| TMEM39A | 1.034659537 | 7.898408814 | 5.363921413 | 3.11E-05 | 0.00026398 | 2.316953804 |
| OR2W3 | -2.264586213 | 7.873184685 | -5.362843929 | 3.11E-05 | 0.000264493 | 2.314546212 |
| GAL3ST1 | -1.231216352 | 4.959133385 | -5.362109674 | 3.12E-05 | 0.000264677 | 2.312905493 |
| TTC33 | 1.351351291 | 5.413777557 | 5.362106369 | 3.12E-05 | 0.000264677 | 2.312898106 |
| GGCT | 0.82499458 | 8.200092009 | 5.360630722 | 3.13E-05 | 0.000265429 | 2.309600569 |
| USP47 | 1.249816096 | 7.660908619 | 5.35984231 | 3.13E-05 | 0.000265773 | 2.307838674 |
| ZNF148 | 0.520325224 | 8.731423867 | 5.358709161 | 3.14E-05 | 0.000266323 | 2.305306284 |
| ZDBF2 | 2.173346331 | 3.425201427 | 5.357345514 | 3.15E-05 | 0.000267013 | 2.302258614 |
| GLIPR1 | 1.21284055 | 9.088917196 | 5.35628363 | 3.16E-05 | 0.000267524 | 2.299885254 |
| RC3H2 | 1.152709704 | 8.595381727 | 5.355603273 | 3.16E-05 | 0.000267805 | 2.29836457 |
| GLMN | 1.318318134 | 5.999916463 | 5.352941797 | 3.18E-05 | 0.000269285 | 2.292415435 |
| NUP54 | 1.205882593 | 8.884178318 | 5.352088933 | 3.19E-05 | 0.000269673 | 2.290508915 |
| SIGLEC14 | -1.084598623 | 10.80836851 | -5.350825833 | 3.20E-05 | 0.000270311 | 2.287685218 |
| ZNF782 | 1.245990546 | 7.330309912 | 5.34995262 | 3.21E-05 | 0.000270497 | 2.28573304 |
| MDN1 | 1.659988001 | 4.708932357 | 5.349813825 | 3.21E-05 | 0.000270497 | 2.285422742 |
| 8-Mar | -1.426398588 | 7.821379553 | -5.349768126 | 3.21E-05 | 0.000270497 | 2.285320574 |
| USP51 | 1.267269119 | 2.8679047 | 5.349667834 | 3.21E-05 | 0.000270497 | 2.285096353 |
| PARG | 1.872026027 | 5.473869824 | 5.348793709 | 3.21E-05 | 0.000270858 | 2.283142046 |
| BZW1 | 1.161257816 | 9.978856741 | 5.348650755 | 3.21E-05 | 0.000270858 | 2.282822434 |
| NET1 | 1.661442237 | 6.279744352 | 5.347524649 | 3.22E-05 | 0.000271162 | 2.280304652 |
| ANXA11 | -0.821344276 | 12.90492025 | -5.347520604 | 3.22E-05 | 0.000271162 | 2.280295606 |
| SMCHD1 | 0.992537267 | 10.86571367 | 5.347515328 | 3.22E-05 | 0.000271162 | 2.28028381 |
| CTRC | -1.258616229 | 6.54170074 | -5.347168108 | 3.23E-05 | 0.000271244 | 2.279507462 |
| LRRC28 | -0.811126659 | 6.837811836 | -5.345620337 | 3.24E-05 | 0.000272014 | 2.276046676 |
| IRAK4 | 1.016497192 | 6.571495688 | 5.345485271 | 3.24E-05 | 0.000272014 | 2.27574466 |
| PXMP4 | -0.789648391 | 8.46373426 | -5.34481606 | 3.24E-05 | 0.000272295 | 2.274248243 |
| SPATA5 | 1.334972426 | 3.710371633 | 5.343778829 | 3.25E-05 | 0.000272678 | 2.271928817 |
| RTEL1 | -0.785156623 | 9.400427473 | -5.343767572 | 3.25E-05 | 0.000272678 | 2.271903644 |
| DHDH | -1.022527105 | 6.729754121 | -5.339638102 | 3.28E-05 | 0.000275096 | 2.262668498 |
| SLC38A1 | 1.043876355 | 11.48348197 | 5.338342922 | 3.29E-05 | 0.000275768 | 2.259771645 |
| LMAN1 | 2.344450813 | 3.792256378 | 5.338108929 | 3.29E-05 | 0.000275782 | 2.259248273 |
| TRPM3 | -0.913077764 | 4.447543814 | -5.337212275 | 3.30E-05 | 0.000276196 | 2.257242673 |
| TMED2 | 0.822295858 | 11.98234329 | 5.337021794 | 3.30E-05 | 0.000276196 | 2.256816602 |
| PHF14 | 1.295947032 | 8.318696681 | 5.33199082 | 3.34E-05 | 0.000279101 | 2.245562118 |
| DYRK1A | 1.417640426 | 8.169655439 | 5.331956572 | 3.34E-05 | 0.000279101 | 2.245485497 |
| UBE2N | 1.008989651 | 7.45581901 | 5.331129868 | 3.34E-05 | 0.00027943 | 2.24363591 |
| TTC31 | -0.833508367 | 7.944662319 | -5.33101218 | 3.34E-05 | 0.00027943 | 2.243372603 |
| FAM199X | 0.962041725 | 8.17765152 | 5.330151442 | 3.35E-05 | 0.000279792 | 2.241446799 |
| REST | 2.434669628 | 7.245724943 | 5.330017396 | 3.35E-05 | 0.000279792 | 2.241146881 |
| CHMP1A | -0.82478554 | 10.45953613 | -5.329405311 | 3.36E-05 | 0.000279948 | 2.239777363 |
| XPNPEP1 | -1.353395984 | 8.225579208 | -5.329210613 | 3.36E-05 | 0.000279948 | 2.239341727 |
| MDK | -2.057533637 | 8.034587758 | -5.329081861 | 3.36E-05 | 0.000279948 | 2.239053642 |
| SNX21 | -0.868395722 | 7.569324229 | -5.328729489 | 3.36E-05 | 0.000279948 | 2.238265198 |
| GTF2E1 | 1.270794505 | 8.72718671 | 5.328717948 | 3.36E-05 | 0.000279948 | 2.238239375 |
| ZNF678 | 1.018323955 | 7.41103644 | 5.328144438 | 3.37E-05 | 0.000280178 | 2.236956102 |
| CYP2D6 | -1.25537556 | 7.545980047 | -5.327821327 | 3.37E-05 | 0.000280249 | 2.236233106 |
| TSPYL4 | 0.776043688 | 8.726948662 | 5.326106684 | 3.38E-05 | 0.000281202 | 2.23239624 |
| ARG2 | -1.305138702 | 5.610860129 | -5.323934558 | 3.40E-05 | 0.0002824 | 2.22753529 |
| APLN | -1.213092364 | 6.187420451 | -5.323802084 | 3.40E-05 | 0.0002824 | 2.227238816 |
| ATOH8 | -1.97060354 | 5.843145054 | -5.323155285 | 3.40E-05 | 0.000282587 | 2.225791271 |
| NMD3 | 2.06386041 | 6.268015025 | 5.323089625 | 3.41E-05 | 0.000282587 | 2.225644323 |
| TTC39A | -1.306952007 | 4.222168831 | -5.320763734 | 3.42E-05 | 0.000283937 | 2.220438632 |
| UBE2K | 0.651970253 | 12.00905863 | 5.320488232 | 3.43E-05 | 0.000283937 | 2.219821986 |
| KDM2B | 1.604371375 | 6.156029346 | 5.32034904 | 3.43E-05 | 0.000283937 | 2.219510436 |
| AP1S2 | 1.113793471 | 9.483152805 | 5.319921406 | 3.43E-05 | 0.000283993 | 2.218553261 |
| MAGEB1 | -1.784790832 | 4.853866586 | -5.319842738 | 3.43E-05 | 0.000283993 | 2.218377175 |
| PNKP | -1.033703475 | 10.17606032 | -5.318268191 | 3.44E-05 | 0.00028487 | 2.214852709 |
| UTP3 | 0.734045792 | 10.99409147 | 5.317833917 | 3.45E-05 | 0.000285015 | 2.213880593 |
| CETN3 | 3.218172668 | 5.392201072 | 5.317451513 | 3.45E-05 | 0.000285089 | 2.213024572 |
| CALR3 | -1.792692697 | 4.503335758 | -5.317301679 | 3.45E-05 | 0.000285089 | 2.212689161 |
| ATP6V0D1 | -0.977530352 | 11.9172715 | -5.317082124 | 3.45E-05 | 0.000285096 | 2.212197674 |
| H2AFJ | -0.903912915 | 12.26892911 | -5.315965933 | 3.46E-05 | 0.000285681 | 2.209698944 |
| PDHX | 0.999894083 | 8.036994861 | 5.315226137 | 3.47E-05 | 0.000286023 | 2.208042761 |
| CDKL5 | -1.067364028 | 9.056694678 | -5.313641183 | 3.48E-05 | 0.000286913 | 2.204494359 |
| FAM173B | 1.365117381 | 6.706554011 | 5.31153698 | 3.50E-05 | 0.000288143 | 2.19978312 |
| PRLH | -1.564074823 | 6.535729572 | -5.309554352 | 3.51E-05 | 0.0002893 | 2.195343732 |
| OR2G6 | -1.752937884 | 3.177810152 | -5.308146779 | 3.52E-05 | 0.000290084 | 2.192191767 |
| CDSN | -1.49906483 | 3.323299218 | -5.30708011 | 3.53E-05 | 0.000290647 | 2.189803069 |
| EEF1A1 | 0.873730261 | 15.38820817 | 5.304687169 | 3.55E-05 | 0.000292084 | 2.184443961 |
| CYP1B1 | 1.388901245 | 9.825315051 | 5.304307051 | 3.55E-05 | 0.000292198 | 2.183592621 |
| ASAH1 | 1.015294222 | 12.63804403 | 5.302415371 | 3.57E-05 | 0.000293311 | 2.179355698 |
| ATP1B3 | 0.7282523 | 12.54484587 | 5.301761713 | 3.57E-05 | 0.000293607 | 2.177891583 |
| NSF | 1.29819578 | 9.697354453 | 5.299079265 | 3.59E-05 | 0.000295252 | 2.171882841 |
| TFF3 | -1.874085843 | 7.604314384 | -5.298667811 | 3.60E-05 | 0.000295363 | 2.17096112 |
| DSCR8 | -3.705969482 | 4.177129689 | -5.298499065 | 3.60E-05 | 0.000295363 | 2.170583097 |
| FBXO48 | 0.951432502 | 5.408229189 | 5.29794411 | 3.60E-05 | 0.000295595 | 2.169339881 |
| FSTL1 | -1.689154112 | 7.610242089 | -5.296099808 | 3.62E-05 | 0.00029669 | 2.165208061 |
| ASPM | 1.755128383 | 5.047061114 | 5.29383126 | 3.64E-05 | 0.000298075 | 2.160125396 |
| CTNND2 | -1.20996556 | 5.674622377 | -5.293054381 | 3.64E-05 | 0.000298459 | 2.158384702 |
| PES1 | -1.0057152 | 7.719722868 | -5.292748579 | 3.65E-05 | 0.000298498 | 2.157699499 |
| FTL | -1.166109865 | 15.44582665 | -5.292583748 | 3.65E-05 | 0.000298498 | 2.157330165 |
| ALOXE3 | -2.585549041 | 6.765523799 | -5.28984035 | 3.67E-05 | 0.000300213 | 2.151182714 |
| SNX1 | 0.844939807 | 10.07763488 | 5.286145699 | 3.70E-05 | 0.000302518 | 2.142902662 |
| DNAH2 | -1.864937184 | 5.258278045 | -5.286042051 | 3.70E-05 | 0.000302518 | 2.142670361 |
| CEP55 | 1.649926886 | 3.731872772 | 5.285573952 | 3.71E-05 | 0.000302698 | 2.141621219 |
| MEF2A | 0.634583631 | 11.23676601 | 5.284706853 | 3.71E-05 | 0.00030315 | 2.139677759 |
| ANKRD20A1 | -1.535594459 | 8.236532662 | -5.283856262 | 3.72E-05 | 0.000303543 | 2.137771237 |
| DOCK8 | 0.772049119 | 10.76684605 | 5.283669888 | 3.72E-05 | 0.000303543 | 2.137353487 |
| UBOX5 | -0.810214738 | 9.000605829 | -5.283500051 | 3.72E-05 | 0.000303543 | 2.136972803 |
| SALL3 | -1.999854043 | 9.995057104 | -5.28331257 | 3.73E-05 | 0.000303543 | 2.136552567 |
| GKAP1 | 1.199434476 | 7.754008639 | 5.281703986 | 3.74E-05 | 0.000304507 | 2.132946827 |
| BBS10 | 2.314087031 | 6.550562094 | 5.280665994 | 3.75E-05 | 0.00030508 | 2.130619989 |
| MALT1 | 1.315583839 | 5.861002695 | 5.280460783 | 3.75E-05 | 0.000305081 | 2.130159962 |
| ANKRD65 | -1.034198182 | 2.743219085 | -5.280023684 | 3.75E-05 | 0.000305241 | 2.129180092 |
| MFSD7 | -1.077953395 | 9.696572233 | -5.279535837 | 3.76E-05 | 0.000305436 | 2.12808644 |
| RPL37A | 0.628785412 | 15.91088715 | 5.279195948 | 3.76E-05 | 0.00030553 | 2.127324467 |
| EXD3 | -1.008841732 | 7.100370449 | -5.277766153 | 3.77E-05 | 0.000306308 | 2.124119003 |
| GGTLC2 | -1.02175339 | 8.884773337 | -5.277660701 | 3.77E-05 | 0.000306308 | 2.123882584 |
| ATP6V1G2 | -2.329283879 | 8.052924055 | -5.277314136 | 3.78E-05 | 0.000306407 | 2.123105588 |
| TMPRSS9 | -2.557344147 | 6.095498118 | -5.276165358 | 3.79E-05 | 0.000307061 | 2.120529968 |
| CPNE9 | -1.202692852 | 4.846717717 | -5.274897402 | 3.80E-05 | 0.0003078 | 2.117687014 |
| EIF4G2 | 0.775113597 | 13.06566234 | 5.273959677 | 3.80E-05 | 0.000308217 | 2.115584402 |
| CD46 | 2.013195903 | 8.993735349 | 5.273890346 | 3.81E-05 | 0.000308217 | 2.115428942 |
| KIF14 | 1.751588249 | 3.513966352 | 5.273160946 | 3.81E-05 | 0.000308584 | 2.113793389 |
| CAST | 0.904235743 | 11.98254153 | 5.271331756 | 3.83E-05 | 0.000309578 | 2.109691552 |
| GNAI3 | 0.956121661 | 9.41343205 | 5.27133076 | 3.83E-05 | 0.000309578 | 2.109689318 |
| FKBP3 | 1.383584029 | 10.52850849 | 5.270548615 | 3.83E-05 | 0.000309983 | 2.107935325 |
| POLDIP3 | -0.563537744 | 10.96269421 | -5.270325414 | 3.84E-05 | 0.000309997 | 2.107434777 |
| PHGDH | -1.16896885 | 6.657489186 | -5.269358335 | 3.84E-05 | 0.000310458 | 2.105265969 |
| TM2D1 | 0.694385496 | 9.758529969 | 5.269261515 | 3.85E-05 | 0.000310458 | 2.105048834 |
| ATE1 | 2.050836944 | 5.335820245 | 5.268458128 | 3.85E-05 | 0.000310754 | 2.103247062 |
| CAB39L | 0.83417358 | 5.998956802 | 5.268432598 | 3.85E-05 | 0.000310754 | 2.103189804 |
| DDX59 | 1.307201201 | 7.898723512 | 5.26767149 | 3.86E-05 | 0.000311147 | 2.101482801 |
| GPRASP1 | 0.98622079 | 8.272792191 | 5.266852858 | 3.87E-05 | 0.00031158 | 2.099646728 |
| HRH1 | -1.125686831 | 6.123439108 | -5.265331113 | 3.88E-05 | 0.000312511 | 2.096233523 |
| SYDE1 | -1.987349984 | 6.485641408 | -5.264545511 | 3.89E-05 | 0.000312923 | 2.094471377 |
| CDK15 | -2.079473081 | 3.626575469 | -5.264292454 | 3.89E-05 | 0.000312959 | 2.093903749 |
| FBXO17 | -1.974965618 | 6.538231182 | -5.263605331 | 3.89E-05 | 0.000313302 | 2.092362438 |
| C11orf68 | -1.187940092 | 11.08699718 | -5.262847416 | 3.90E-05 | 0.000313696 | 2.090662287 |
| ALS2CL | -1.462141552 | 7.401792578 | -5.262131944 | 3.91E-05 | 0.00031406 | 2.089057301 |
| RFX2 | -1.275663178 | 9.40305676 | -5.261535222 | 3.91E-05 | 0.000314211 | 2.087718668 |
| ILVBL | -0.815275205 | 10.02788043 | -5.261451141 | 3.91E-05 | 0.000314211 | 2.087530045 |
| C1GALT1C1 | 1.049484268 | 9.994095504 | 5.261313752 | 3.91E-05 | 0.000314211 | 2.087221833 |
| ARRB2 | -0.833306747 | 14.41740874 | -5.260976519 | 3.92E-05 | 0.000314307 | 2.086465294 |
| ST3GAL5 | 1.25976258 | 8.859080855 | 5.260559647 | 3.92E-05 | 0.000314312 | 2.085530084 |
| MYLK4 | 1.719006534 | 4.406881995 | 5.260498383 | 3.92E-05 | 0.000314312 | 2.085392642 |
| AHCTF1 | 1.045656428 | 7.904277052 | 5.260364213 | 3.92E-05 | 0.000314312 | 2.085091641 |
| COPE | -0.875655627 | 8.621291321 | -5.257862458 | 3.95E-05 | 0.000315951 | 2.079478854 |
| SESN3 | -1.360124867 | 9.379213251 | -5.25724092 | 3.95E-05 | 0.000316252 | 2.078084326 |
| CCDC129 | -2.838597495 | 7.266086062 | -5.255802169 | 3.96E-05 | 0.000317138 | 2.074856119 |
| PRKD3 | 1.052618877 | 8.553615821 | 5.255361754 | 3.97E-05 | 0.000317308 | 2.073867899 |
| MBD2 | 0.590206035 | 11.72100752 | 5.255162991 | 3.97E-05 | 0.000317308 | 2.073421903 |
| UBA6 | 1.497337867 | 5.593476301 | 5.254387215 | 3.98E-05 | 0.000317721 | 2.071681135 |
| ANKRA2 | 1.124649045 | 8.087061814 | 5.25289075 | 3.99E-05 | 0.00031854 | 2.068323073 |
| IL18R1 | 1.508356542 | 7.544383407 | 5.252847608 | 3.99E-05 | 0.00031854 | 2.068226258 |
| NAP1L3 | 1.87563297 | 4.765577895 | 5.25238539 | 3.99E-05 | 0.000318728 | 2.067189003 |
| ZBTB22 | -0.719469628 | 6.755655032 | -5.250920414 | 4.01E-05 | 0.000319591 | 2.063901356 |
| NANP | 1.340890791 | 3.968168125 | 5.250789183 | 4.01E-05 | 0.000319591 | 2.063606844 |
| SPCS3 | 1.409906328 | 10.84038503 | 5.248885183 | 4.03E-05 | 0.000320825 | 2.059333667 |
| CDK13 | 0.843488717 | 8.656878399 | 5.247084223 | 4.04E-05 | 0.000321989 | 2.055291468 |
| ZACN | -1.934321734 | 5.559454361 | -5.246809452 | 4.05E-05 | 0.000322016 | 2.054674729 |
| TBX18 | -1.317041849 | 3.136164501 | -5.246646683 | 4.05E-05 | 0.000322016 | 2.054309381 |
| B4GALT4 | 1.406179619 | 6.541725426 | 5.246282719 | 4.05E-05 | 0.000322136 | 2.053492431 |
| NT5C2 | 1.621327544 | 9.318458037 | 5.245582376 | 4.06E-05 | 0.000322501 | 2.051920413 |
| POLR1C | -1.668978854 | 9.62854639 | -5.24315529 | 4.08E-05 | 0.00032413 | 2.046472164 |
| STK19 | -0.745631809 | 10.52956994 | -5.242911795 | 4.08E-05 | 0.000324162 | 2.045925548 |
| SCGB1C1 | -2.387970644 | 6.519137146 | -5.241506291 | 4.09E-05 | 0.000324913 | 2.042770263 |
| RHOBTB3 | 1.803832006 | 4.386593592 | 5.241490209 | 4.09E-05 | 0.000324913 | 2.042734158 |
| RAB21 | 0.824950421 | 9.583786131 | 5.239372741 | 4.11E-05 | 0.000326213 | 2.037980243 |
| NAP1L5 | 1.803693698 | 4.959775319 | 5.239327792 | 4.11E-05 | 0.000326213 | 2.037879323 |
| MDM4 | 1.398493318 | 9.602634973 | 5.238432342 | 4.12E-05 | 0.000326727 | 2.035868837 |
| NOL8 | 1.030007736 | 10.90042601 | 5.237888008 | 4.13E-05 | 0.000326795 | 2.034646655 |
| EPC2 | 0.950700913 | 9.782851667 | 5.237745093 | 4.13E-05 | 0.000326795 | 2.034325765 |
| VPS41 | 1.337456794 | 7.702509276 | 5.237743203 | 4.13E-05 | 0.000326795 | 2.034321522 |
| CIDEC | -0.928384248 | 8.516903751 | -5.235679247 | 4.15E-05 | 0.000328118 | 2.029687111 |
| DNAJC21 | 1.261662229 | 7.384091583 | 5.235561552 | 4.15E-05 | 0.000328118 | 2.029422828 |
| SRP72 | 0.92891896 | 10.08370019 | 5.235147394 | 4.15E-05 | 0.000328278 | 2.028492831 |
| TNIP1 | -0.885301477 | 13.34071749 | -5.234777851 | 4.16E-05 | 0.000328405 | 2.027663003 |
| HAT1 | 1.771344122 | 9.912726119 | 5.228157057 | 4.22E-05 | 0.000333215 | 2.012793806 |
| GOLGA6C | 2.24490403 | 4.213805198 | 5.22757123 | 4.23E-05 | 0.000333507 | 2.011477963 |
| NDUFB5 | 0.647631701 | 10.10285715 | 5.226857092 | 4.23E-05 | 0.000333897 | 2.00987388 |
| HIST1H2BF | 2.963693184 | 5.913475056 | 5.226279296 | 4.24E-05 | 0.000334184 | 2.008576014 |
| ZFP2 | 1.936533717 | 4.126609964 | 5.225772668 | 4.24E-05 | 0.000334418 | 2.007437987 |
| SLC16A9 | -1.555739763 | 5.75426701 | -5.2248833 | 4.25E-05 | 0.000334861 | 2.005440168 |
| DLGAP3 | -0.797233977 | 8.42048311 | -5.224791958 | 4.25E-05 | 0.000334861 | 2.005234978 |
| LRRC16A | 1.277725071 | 2.741088736 | 5.223109531 | 4.27E-05 | 0.000335989 | 2.001455495 |
| TBCCD1 | 1.100665644 | 5.13778725 | 5.222105348 | 4.28E-05 | 0.000336524 | 1.999199539 |
| NCAM2 | -2.114029628 | 6.319539062 | -5.221874803 | 4.28E-05 | 0.000336524 | 1.998681596 |
| ZFAND1 | 1.285684859 | 8.555807145 | 5.221813883 | 4.28E-05 | 0.000336524 | 1.998544732 |
| RAB3IP | 1.208463577 | 8.368406302 | 5.221419561 | 4.28E-05 | 0.000336675 | 1.997658833 |
| CRABP2 | -1.869839637 | 6.311956561 | -5.220890598 | 4.29E-05 | 0.000336928 | 1.996470425 |
| DBR1 | 1.053506743 | 7.256596682 | 5.220047441 | 4.30E-05 | 0.000337421 | 1.994576076 |
| YIPF3 | -0.839104695 | 11.74146496 | -5.219674793 | 4.30E-05 | 0.000337555 | 1.993738819 |
| FAM60A | 1.213760539 | 10.69888035 | 5.218775524 | 4.31E-05 | 0.000338064 | 1.991718312 |
| CNN1 | -2.379161613 | 5.918816466 | -5.218616116 | 4.31E-05 | 0.000338064 | 1.991360142 |
| BTG1 | 0.918368909 | 13.2862515 | 5.217342969 | 4.32E-05 | 0.000338889 | 1.988499465 |
| GPSM3 | -0.890636923 | 13.5880829 | -5.216689561 | 4.33E-05 | 0.00033924 | 1.987031249 |
| UBA2 | 0.909597638 | 9.067319908 | 5.216380213 | 4.33E-05 | 0.000339327 | 1.986336128 |
| ZNF234 | 1.188491284 | 8.810180426 | 5.215136538 | 4.35E-05 | 0.000340132 | 1.98354145 |
| ZNF595 | 1.107576383 | 7.143169323 | 5.212961707 | 4.37E-05 | 0.00034166 | 1.978654055 |
| ARNTL2 | 2.272461958 | 3.835097065 | 5.212411941 | 4.37E-05 | 0.000341934 | 1.977418533 |
| HECTD2 | 1.372657889 | 4.660884152 | 5.21175781 | 4.38E-05 | 0.000342289 | 1.975948431 |
| DAB2 | -0.914182066 | 8.089806055 | -5.210939233 | 4.39E-05 | 0.000342671 | 1.974108704 |
| IDUA | -0.56027836 | 9.160319082 | -5.210849609 | 4.39E-05 | 0.000342671 | 1.973907273 |
| YPEL5 | 0.838054821 | 11.38330853 | 5.210632106 | 4.39E-05 | 0.000342671 | 1.973418432 |
| PDCD6IP | 1.263984385 | 7.361505315 | 5.210481708 | 4.39E-05 | 0.000342671 | 1.973080407 |
| TCEB1 | 0.761552473 | 12.29761401 | 5.209971549 | 4.40E-05 | 0.000342915 | 1.971933795 |
| ATXN2 | -0.882862842 | 10.73059731 | -5.208990049 | 4.41E-05 | 0.000343526 | 1.969727753 |
| TIGD7 | 1.856563979 | 5.816404727 | 5.208047987 | 4.42E-05 | 0.000344108 | 1.967610282 |
| DYRK1B | -1.638733891 | 8.921260508 | -5.206823045 | 4.43E-05 | 0.000344912 | 1.964856875 |
| DYNC1LI2 | 1.241697728 | 6.916869359 | 5.20637966 | 4.43E-05 | 0.000345106 | 1.963860213 |
| CECR2 | -2.13087868 | 7.335257927 | -5.204939222 | 4.45E-05 | 0.000346081 | 1.96062221 |
| NAP1L2 | 2.105163415 | 5.436004278 | 5.203595002 | 4.46E-05 | 0.000346983 | 1.95760035 |
| MAGIX | -0.805835991 | 5.53109507 | -5.20300556 | 4.47E-05 | 0.000347294 | 1.956275215 |
| SKIV2L2 | 1.175729297 | 10.16247039 | 5.20106246 | 4.49E-05 | 0.000348672 | 1.951906702 |
| KIAA0907 | 1.122962996 | 7.863418917 | 5.199100098 | 4.51E-05 | 0.000350072 | 1.947494576 |
| STAM2 | 1.591547796 | 6.466192052 | 5.198066671 | 4.52E-05 | 0.000350738 | 1.94517092 |
| NEUROD2 | -1.802729051 | 3.391638454 | -5.195814339 | 4.54E-05 | 0.000352378 | 1.940106269 |
| ZNF14 | 1.121119731 | 8.335000616 | 5.195390226 | 4.55E-05 | 0.000352562 | 1.939152551 |
| PQLC1 | -1.232415722 | 10.97572758 | -5.195066673 | 4.55E-05 | 0.000352665 | 1.938424956 |
| ZNF525 | 2.107588468 | 12.37414666 | 5.194223956 | 4.56E-05 | 0.000352969 | 1.936529848 |
| SASS6 | 1.651311208 | 3.746000134 | 5.194110503 | 4.56E-05 | 0.000352969 | 1.93627471 |
| RAD51C | 1.349839207 | 8.612763733 | 5.194103593 | 4.56E-05 | 0.000352969 | 1.93625917 |
| BLOC1S2 | 1.375488616 | 10.20194522 | 5.19352994 | 4.56E-05 | 0.000353273 | 1.934969096 |
| DCAF11 | -1.360651583 | 10.236174 | -5.193334229 | 4.57E-05 | 0.000353274 | 1.934528961 |
| CCDC69 | -0.780201492 | 8.655412816 | -5.190341528 | 4.60E-05 | 0.000355523 | 1.927798282 |
| SMOC1 | -1.999010229 | 6.586260887 | -5.189385213 | 4.61E-05 | 0.000356138 | 1.92564735 |
| VAPA | 0.59290371 | 8.779507189 | 5.187881951 | 4.62E-05 | 0.000357198 | 1.922266083 |
| ZNF574 | -1.229804119 | 10.01433596 | -5.185638763 | 4.65E-05 | 0.000358862 | 1.917220179 |
| WDR5B | 1.369051304 | 5.068089739 | 5.18279199 | 4.68E-05 | 0.000361028 | 1.91081598 |
| SLC20A1 | 0.76413799 | 12.4051898 | 5.182166508 | 4.68E-05 | 0.000361382 | 1.90940879 |
| AXIN2 | 1.584683753 | 7.465494719 | 5.18158095 | 4.69E-05 | 0.000361608 | 1.908091389 |
| BEND4 | 2.069034422 | 5.456539167 | 5.181473901 | 4.69E-05 | 0.000361608 | 1.907850547 |
| ATG5 | 1.075220126 | 7.903427574 | 5.181311799 | 4.69E-05 | 0.000361608 | 1.90748584 |
| TRIM61 | 1.533346894 | 5.531261681 | 5.180129122 | 4.71E-05 | 0.00036242 | 1.904824922 |
| LYRM5 | 1.288143464 | 6.513157636 | 5.179051484 | 4.72E-05 | 0.000363134 | 1.902400239 |
| ZMYND8 | -1.018241075 | 10.3820292 | -5.178875972 | 4.72E-05 | 0.000363134 | 1.902005327 |
| ZNF451 | 1.419339824 | 7.780094833 | 5.177449788 | 4.73E-05 | 0.000364152 | 1.898796257 |
| SKA2 | 1.414287696 | 5.388031082 | 5.176756827 | 4.74E-05 | 0.000364565 | 1.89723696 |
| TRMT1L | 2.438255807 | 6.227603258 | 5.174864003 | 4.76E-05 | 0.000365974 | 1.892977547 |
| SHD | -2.131820603 | 8.487942649 | -5.174502445 | 4.77E-05 | 0.000366115 | 1.892163903 |
| HSDL1 | 1.278658203 | 5.527907046 | 5.174185027 | 4.77E-05 | 0.000366218 | 1.891449582 |
| ANO2 | -1.875644842 | 4.500116554 | -5.173581196 | 4.78E-05 | 0.00036656 | 1.890090694 |
| MTX2 | 1.178177119 | 7.717156749 | 5.173307083 | 4.78E-05 | 0.000366629 | 1.889473808 |
| SBDS | 1.848887969 | 8.022195033 | 5.172925169 | 4.78E-05 | 0.000366786 | 1.888614308 |
| ZFX | 2.287155658 | 5.476268341 | 5.171771462 | 4.80E-05 | 0.000367588 | 1.886017814 |
| C16orf72 | 0.762093419 | 10.57431389 | 5.169111754 | 4.82E-05 | 0.000369581 | 1.880031573 |
| NAA10 | -0.766664717 | 10.07124585 | -5.169005173 | 4.83E-05 | 0.000369581 | 1.879791676 |
| COL5A1 | -1.314621132 | 5.782802293 | -5.168755505 | 4.83E-05 | 0.000369629 | 1.879229716 |
| SLC14A1 | -1.388871785 | 8.236058629 | -5.168206732 | 4.83E-05 | 0.000369929 | 1.877994501 |
| SSBP3 | -1.947547804 | 6.95451926 | -5.167805163 | 4.84E-05 | 0.000370105 | 1.877090608 |
| MRPS25 | 1.178047026 | 8.393259593 | 5.166967032 | 4.85E-05 | 0.000370649 | 1.875204015 |
| DNAJC4 | -1.350879885 | 10.1712455 | -5.16592917 | 4.86E-05 | 0.000371361 | 1.872867762 |
| KRTAP4-12 | -2.492085734 | 6.530585662 | -5.165356756 | 4.87E-05 | 0.000371683 | 1.871579206 |
| PPP2R5E | 0.854072667 | 10.39792492 | 5.164517538 | 4.87E-05 | 0.000371891 | 1.869690008 |
| URM1 | -0.529794257 | 11.71835563 | -5.164391418 | 4.88E-05 | 0.000371891 | 1.869406089 |
| ARMC8 | 0.543568018 | 9.300541886 | 5.164386215 | 4.88E-05 | 0.000371891 | 1.869394376 |
| ACOT12 | -3.058418932 | 5.872084157 | -5.16417801 | 4.88E-05 | 0.000371891 | 1.868925667 |
| DPY19L3 | 1.853150145 | 5.021520854 | 5.164154572 | 4.88E-05 | 0.000371891 | 1.868872902 |
| IL1F10 | -2.108081925 | 6.318447892 | -5.162847524 | 4.89E-05 | 0.000372763 | 1.865930408 |
| MAP3K1 | 0.808463131 | 12.9786259 | 5.162740336 | 4.89E-05 | 0.000372763 | 1.865689094 |
| INO80C | -1.014271558 | 8.329193553 | -5.161138464 | 4.91E-05 | 0.00037396 | 1.862082681 |
| CCDC126 | 1.093238273 | 5.972342082 | 5.16031748 | 4.92E-05 | 0.000374495 | 1.860234262 |
| CNIH2 | -1.956613896 | 5.674689136 | -5.15941457 | 4.93E-05 | 0.000375101 | 1.85820133 |
| SFXN3 | -0.799723977 | 8.816790213 | -5.158690699 | 4.94E-05 | 0.000375414 | 1.856571463 |
| SNX5 | 0.689159009 | 11.92913895 | 5.158666579 | 4.94E-05 | 0.000375414 | 1.856517155 |
| GPR88 | -3.620502938 | 9.017345421 | -5.158164128 | 4.95E-05 | 0.00037568 | 1.855385812 |
| ARID4A | 1.550337452 | 7.067678314 | 5.156758192 | 4.96E-05 | 0.000376719 | 1.852220038 |
| APEX2 | -0.685087873 | 9.887876188 | -5.155834312 | 4.97E-05 | 0.000377347 | 1.850139637 |
| RAB35 | -0.711706746 | 11.42242285 | -5.15517591 | 4.98E-05 | 0.000377749 | 1.848657002 |
| TMEM108 | -0.929896467 | 4.878850134 | -5.152949746 | 5.00E-05 | 0.000379362 | 1.843643722 |
| MED21 | 1.10827111 | 7.845233488 | 5.152919676 | 5.00E-05 | 0.000379362 | 1.843576003 |
| MRPL1 | 1.933903565 | 4.969656614 | 5.151025837 | 5.03E-05 | 0.000380833 | 1.839310809 |
| AGPS | 1.211903677 | 8.706101749 | 5.150824868 | 5.03E-05 | 0.000380843 | 1.838858183 |
| ACOT7 | -0.887809187 | 7.648088123 | -5.150296374 | 5.03E-05 | 0.000381136 | 1.837667883 |
| CKAP2 | 1.05482135 | 6.213702042 | 5.15007003 | 5.04E-05 | 0.000381168 | 1.837158095 |
| ZCCHC11 | 1.057391491 | 6.87289963 | 5.149760252 | 5.04E-05 | 0.000381272 | 1.836460382 |
| LRFN1 | -0.90160317 | 8.748684042 | -5.149311389 | 5.05E-05 | 0.000381497 | 1.835449394 |
| SP3 | 1.242151941 | 9.270938377 | 5.148790646 | 5.05E-05 | 0.000381785 | 1.834276491 |
| HLTF | 1.490614586 | 7.275696431 | 5.148393175 | 5.06E-05 | 0.000381965 | 1.833381229 |
| RQCD1 | 1.945549867 | 4.102015 | 5.148067843 | 5.06E-05 | 0.000382084 | 1.832648442 |
| UPF3A | 1.490867597 | 6.453442501 | 5.146780313 | 5.08E-05 | 0.000383038 | 1.829748295 |
| PRRG4 | -1.069783456 | 7.044962557 | -5.146415443 | 5.08E-05 | 0.000383115 | 1.828926407 |
| GPKOW | -0.621367119 | 11.15047255 | -5.146314756 | 5.08E-05 | 0.000383115 | 1.828699601 |
| CLEC7A | 1.902379952 | 6.474965084 | 5.143912071 | 5.11E-05 | 0.000385047 | 1.823287158 |
| ANKDD1A | -1.887355561 | 6.323024752 | -5.141406726 | 5.14E-05 | 0.000387057 | 1.817642988 |
| SPRY2 | 1.572725444 | 5.372654096 | 5.141174937 | 5.14E-05 | 0.000387057 | 1.817120777 |
| PRKCI | 1.081139314 | 7.917254183 | 5.141026732 | 5.14E-05 | 0.000387057 | 1.816786874 |
| ZNF845 | 1.026733053 | 8.512792808 | 5.140866285 | 5.14E-05 | 0.000387057 | 1.81642539 |
| ASCC3 | 1.871830665 | 8.337656209 | 5.138585937 | 5.17E-05 | 0.000388901 | 1.811287599 |
| PLEKHM1 | -0.719345819 | 11.9635532 | -5.135295715 | 5.21E-05 | 0.000391526 | 1.803873791 |
| CPNE8 | 1.419101777 | 5.91639066 | 5.135250655 | 5.21E-05 | 0.000391526 | 1.803772253 |
| ZNF415 | 1.872257421 | 4.667414991 | 5.135058226 | 5.21E-05 | 0.000391529 | 1.803338629 |
| AQP8 | -1.938674482 | 6.381925596 | -5.133598033 | 5.23E-05 | 0.000392541 | 1.800048111 |
| DPYD | 1.046198779 | 8.6132459 | 5.133409265 | 5.23E-05 | 0.000392541 | 1.799622714 |
| ALG5 | 0.814167269 | 10.83340669 | 5.133359085 | 5.23E-05 | 0.000392541 | 1.799509631 |
| DZANK1 | 1.114190157 | 6.205031103 | 5.132188312 | 5.25E-05 | 0.00039342 | 1.796871179 |
| PSRC1 | -0.945444569 | 7.977755286 | -5.131949913 | 5.25E-05 | 0.000393465 | 1.79633391 |
| C21orf58 | -3.744449145 | 9.671857186 | -5.131585052 | 5.25E-05 | 0.000393624 | 1.795511632 |
| NOP58 | 1.341216711 | 11.39028259 | 5.130966935 | 5.26E-05 | 0.000394009 | 1.794118576 |
| ORC2 | 1.017347884 | 7.520827807 | 5.130298324 | 5.27E-05 | 0.000394441 | 1.792611685 |
| STRAP | 0.702595921 | 11.82957429 | 5.129331811 | 5.28E-05 | 0.000394858 | 1.79043334 |
| ACYP1 | 0.993351348 | 9.888023842 | 5.12927437 | 5.28E-05 | 0.000394858 | 1.790303875 |
| NSD1 | -0.644614549 | 7.962368835 | -5.129271901 | 5.28E-05 | 0.000394858 | 1.79029831 |
| GLIS1 | -2.029692553 | 7.109649899 | -5.128234521 | 5.29E-05 | 0.000395622 | 1.787960159 |
| SCT | -1.076625549 | 6.556103024 | -5.127777072 | 5.30E-05 | 0.000395865 | 1.786929089 |
| CREB1 | 0.964895719 | 9.134088264 | 5.126189329 | 5.32E-05 | 0.000397128 | 1.783350262 |
| SEL1L3 | 1.288193065 | 10.70176605 | 5.124708968 | 5.34E-05 | 0.000398298 | 1.780013309 |
| NEK4 | 1.394735552 | 6.368234564 | 5.124205077 | 5.34E-05 | 0.000398585 | 1.778877426 |
| JUN | 1.646668591 | 5.299970742 | 5.124016997 | 5.34E-05 | 0.000398587 | 1.778453449 |
| FANCF | 0.916608122 | 9.682448528 | 5.122151251 | 5.37E-05 | 0.000399958 | 1.774247451 |
| CAPN14 | 0.977090854 | 4.051592068 | 5.122133282 | 5.37E-05 | 0.000399958 | 1.774206943 |
| RLIM | 1.458406083 | 7.285050338 | 5.121263368 | 5.38E-05 | 0.000400581 | 1.772245784 |
| HBE1 | -1.57803879 | 3.456367267 | -5.120499643 | 5.39E-05 | 0.000401107 | 1.770523974 |
| SCFD1 | 0.941792679 | 10.38820932 | 5.120144759 | 5.39E-05 | 0.000401261 | 1.769723877 |
| SS18L2 | 0.943894113 | 11.8836277 | 5.118207946 | 5.42E-05 | 0.000402862 | 1.765357109 |
| COPB1 | 0.749715735 | 11.04649799 | 5.115019615 | 5.46E-05 | 0.000405622 | 1.758168045 |
| NF1 | 1.081704313 | 5.356611576 | 5.114621806 | 5.46E-05 | 0.000405818 | 1.757271008 |
| SPAST | 1.134804011 | 7.865210807 | 5.11314475 | 5.48E-05 | 0.000407011 | 1.753940237 |
| PTOV1 | -1.055534455 | 9.8239523 | -5.112959578 | 5.48E-05 | 0.000407011 | 1.753522661 |
| ZNF529 | 0.904903348 | 7.662586337 | 5.112550189 | 5.49E-05 | 0.000407218 | 1.752599451 |
| ENTPD2 | -1.347266855 | 7.26542932 | -5.112178926 | 5.49E-05 | 0.00040739 | 1.75176221 |
| STK40 | -0.753974893 | 15.05976013 | -5.111874795 | 5.49E-05 | 0.0004075 | 1.75107635 |
| ZNF280A | 1.261070603 | 4.620171187 | 5.110810944 | 5.51E-05 | 0.000408316 | 1.748677158 |
| STK39 | 1.463364041 | 8.187551362 | 5.110093471 | 5.52E-05 | 0.00040881 | 1.747059071 |
| ZNF878 | 0.92672155 | 2.631973095 | 5.109377366 | 5.53E-05 | 0.000409177 | 1.745444028 |
| STXBP2 | -0.979065912 | 8.201641481 | -5.109329188 | 5.53E-05 | 0.000409177 | 1.745335371 |
| ATP5F1 | 1.05210052 | 12.13406383 | 5.109056635 | 5.53E-05 | 0.000409258 | 1.744720668 |
| TDP2 | 0.986326067 | 11.30332065 | 5.108708618 | 5.53E-05 | 0.000409388 | 1.743935759 |
| PC | -1.775705634 | 9.04162172 | -5.108548097 | 5.54E-05 | 0.000409388 | 1.743573722 |
| CNR1 | 2.14915412 | 4.831813655 | 5.108126428 | 5.54E-05 | 0.000409608 | 1.742622683 |
| SLC44A5 | -1.891474051 | 3.583563148 | -5.104565992 | 5.59E-05 | 0.000412766 | 1.734591898 |
| PHF21B | -1.804646608 | 8.118983603 | -5.103641124 | 5.60E-05 | 0.000413462 | 1.732505648 |
| CCNT2 | 2.61384281 | 5.770239519 | 5.102445184 | 5.61E-05 | 0.000414415 | 1.729807842 |
| GNG11 | -1.15930186 | 10.63963307 | -5.100326461 | 5.64E-05 | 0.000416243 | 1.725028161 |
| VTA1 | 0.867203409 | 7.056101528 | 5.097133772 | 5.68E-05 | 0.000418937 | 1.717825076 |
| CD44 | 0.60539221 | 13.34058309 | 5.097073215 | 5.68E-05 | 0.000418937 | 1.717688446 |
| YIF1B | -0.96821224 | 7.773487302 | -5.096939804 | 5.68E-05 | 0.000418937 | 1.717387437 |
| ZIC5 | -4.3911146 | 4.936099021 | -5.094257355 | 5.72E-05 | 0.000421326 | 1.711334905 |
| RNF145 | 1.006664631 | 8.594557009 | 5.093875876 | 5.72E-05 | 0.000421515 | 1.710474114 |
| RARS2 | 0.981559255 | 11.1400656 | 5.092030357 | 5.75E-05 | 0.000423113 | 1.706309629 |
| GALC | 0.839982767 | 9.078881405 | 5.091692305 | 5.75E-05 | 0.000423262 | 1.705546776 |
| MCCC2 | 1.02053349 | 6.425434717 | 5.091395166 | 5.76E-05 | 0.000423311 | 1.70487624 |
| PAK1IP1 | 1.235548546 | 7.489977944 | 5.091273797 | 5.76E-05 | 0.000423311 | 1.704602354 |
| PEF1 | -1.745088932 | 10.91815475 | -5.089645491 | 5.78E-05 | 0.00042459 | 1.700927725 |
| GCAT | -1.293413496 | 5.238139473 | -5.089581633 | 5.78E-05 | 0.00042459 | 1.700783613 |
| RFC5 | 0.856183492 | 9.644742523 | 5.089069594 | 5.79E-05 | 0.000424785 | 1.699628043 |
| NBPF6 | -1.474980433 | 7.241864435 | -5.089012702 | 5.79E-05 | 0.000424785 | 1.699499648 |
| ZNF572 | 0.9137366 | 6.832106064 | 5.088609688 | 5.79E-05 | 0.000424998 | 1.69859011 |
| PRR7 | -1.224546104 | 9.554782968 | -5.088037726 | 5.80E-05 | 0.000425374 | 1.697299265 |
| SF3B1 | 2.367795406 | 8.07840231 | 5.086491224 | 5.82E-05 | 0.000426697 | 1.693808891 |
| ZNF142 | -0.665961904 | 8.937171401 | -5.085861078 | 5.83E-05 | 0.000427131 | 1.692386634 |
| N4BP1 | -0.733297215 | 10.31619608 | -5.085253621 | 5.84E-05 | 0.000427545 | 1.69101556 |
| PGPEP1L | -1.128191071 | 5.439043851 | -5.084826464 | 5.84E-05 | 0.000427783 | 1.690051421 |
| LNX2 | 1.162869069 | 7.610195334 | 5.08447711 | 5.85E-05 | 0.000427945 | 1.689262883 |
| SEZ6L2 | -2.327241954 | 6.565694693 | -5.084188653 | 5.85E-05 | 0.000428048 | 1.688611789 |
| NAMPT | 0.892622479 | 13.12799087 | 5.083289337 | 5.86E-05 | 0.000428711 | 1.68658185 |
| C1orf194 | -2.243865537 | 7.68520311 | -5.083143999 | 5.87E-05 | 0.000428711 | 1.686253789 |
| GPR139 | -4.387642566 | 5.015790808 | -5.082645672 | 5.87E-05 | 0.000428983 | 1.685128934 |
| CITED4 | -0.988209761 | 11.97048009 | -5.08250062 | 5.87E-05 | 0.000428983 | 1.684801511 |
| RNF31 | -0.688796247 | 11.42559037 | -5.082217909 | 5.88E-05 | 0.000429081 | 1.684163347 |
| GFOD2 | -1.186471439 | 9.93882171 | -5.081179967 | 5.89E-05 | 0.000429918 | 1.681820352 |
| DEPDC1 | 1.110915414 | 4.769804193 | 5.08078114 | 5.90E-05 | 0.00043013 | 1.68092004 |
| HDLBP | -0.829226804 | 10.35871649 | -5.080213459 | 5.90E-05 | 0.000430508 | 1.679638538 |
| TSPYL1 | 0.781790001 | 10.56546906 | 5.079826608 | 5.91E-05 | 0.000430709 | 1.678765234 |
| PPP1R1A | -1.254246898 | 4.270461265 | -5.079632409 | 5.91E-05 | 0.000430721 | 1.678326832 |
| FBXO11 | 1.52341497 | 7.06671765 | 5.078534311 | 5.93E-05 | 0.000431386 | 1.67584784 |
| PIGQ | -1.545325088 | 7.530980409 | -5.078380053 | 5.93E-05 | 0.000431386 | 1.675499588 |
| DPEP1 | -1.545691938 | 4.19470344 | -5.078315705 | 5.93E-05 | 0.000431386 | 1.675354318 |
| FBXO25 | 0.936075732 | 10.03669938 | 5.07822797 | 5.93E-05 | 0.000431386 | 1.675156247 |
| SAMD12 | 1.186713963 | 4.521837289 | 5.076308101 | 5.96E-05 | 0.000433098 | 1.67082183 |
| KBTBD6 | 1.555242949 | 6.826876758 | 5.075745053 | 5.97E-05 | 0.000433475 | 1.669550607 |
| SEL1L | 0.827120223 | 7.7840562 | 5.073595935 | 5.99E-05 | 0.000435422 | 1.664698225 |
| ELOVL5 | 0.82960531 | 9.472828236 | 5.072737541 | 6.01E-05 | 0.000436094 | 1.662760011 |
| NEK1 | 1.269354622 | 6.066085989 | 5.072412252 | 6.01E-05 | 0.000436237 | 1.66202551 |
| PRSS53 | -0.7437948 | 8.589485848 | -5.071272094 | 6.03E-05 | 0.000437192 | 1.659450976 |
| C20orf196 | -0.61253952 | 8.474180623 | -5.070557827 | 6.04E-05 | 0.000437723 | 1.65783808 |
| CTDP1 | -0.558461243 | 9.25549239 | -5.067886451 | 6.07E-05 | 0.000440214 | 1.651805489 |
| ABCC6 | -1.027165813 | 6.938178886 | -5.064138766 | 6.13E-05 | 0.000443807 | 1.643341492 |
| EVI2A | 2.21916056 | 11.16820078 | 5.062669123 | 6.15E-05 | 0.000445112 | 1.640022092 |
| PKIA | 0.838933981 | 8.774419472 | 5.061593608 | 6.16E-05 | 0.000445787 | 1.637592792 |
| FZD3 | 1.676734246 | 5.476016871 | 5.06155591 | 6.16E-05 | 0.000445787 | 1.637507642 |
| TMOD2 | 1.83984799 | 6.65428479 | 5.061452195 | 6.16E-05 | 0.000445787 | 1.637273373 |
| NUP133 | 0.851763959 | 8.74184576 | 5.061207821 | 6.17E-05 | 0.000445787 | 1.636721383 |
| MTMR10 | 0.771363117 | 9.749129049 | 5.061066455 | 6.17E-05 | 0.000445787 | 1.636402066 |
| RPS27A | 1.574155394 | 14.65264521 | 5.060921717 | 6.17E-05 | 0.000445787 | 1.63607513 |
| RXRB | -0.978716594 | 8.902184426 | -5.060044652 | 6.18E-05 | 0.000446496 | 1.634093972 |
| DPY19L2 | 1.404594588 | 4.339227478 | 5.059758667 | 6.19E-05 | 0.000446604 | 1.633447964 |
| PNRC1 | 0.97496975 | 12.94611706 | 5.059525738 | 6.19E-05 | 0.000446643 | 1.632921801 |
| PDS5B | 0.934812679 | 7.789650613 | 5.059359427 | 6.19E-05 | 0.000446643 | 1.632546116 |
| C9orf50 | -2.095114766 | 6.704470624 | -5.058632645 | 6.20E-05 | 0.000447201 | 1.630904352 |
| GATS | -0.954624132 | 9.373194524 | -5.05822611 | 6.21E-05 | 0.000447432 | 1.629985991 |
| TRIP11 | 0.80972561 | 8.973104632 | 5.056934736 | 6.23E-05 | 0.000448392 | 1.627068712 |
| TAB3 | 1.343149361 | 6.634884684 | 5.056926024 | 6.23E-05 | 0.000448392 | 1.627049031 |
| TC2N | 1.63276146 | 8.31862863 | 5.056224545 | 6.24E-05 | 0.000448926 | 1.625464307 |
| TBC1D24 | 1.499803009 | 4.414300981 | 5.055788401 | 6.24E-05 | 0.000449189 | 1.624478987 |
| DBT | 0.838897538 | 6.874996559 | 5.055544876 | 6.25E-05 | 0.000449226 | 1.623928819 |
| EIF3M | 1.0532531 | 12.20941551 | 5.055392917 | 6.25E-05 | 0.000449226 | 1.623585515 |
| RRM1 | 0.997357706 | 9.608344181 | 5.054366796 | 6.26E-05 | 0.000450094 | 1.621267261 |
| EID2 | 0.795760085 | 8.815537256 | 5.053928327 | 6.27E-05 | 0.00045029 | 1.620276633 |
| PLIN3 | -0.922392741 | 9.411220172 | -5.05365163 | 6.27E-05 | 0.00045029 | 1.619651489 |
| RPL5 | 1.105955854 | 14.81894418 | 5.05363879 | 6.27E-05 | 0.00045029 | 1.619622479 |
| RAMP1 | -1.499038361 | 8.708362141 | -5.053041255 | 6.28E-05 | 0.00045072 | 1.618272441 |
| EFEMP2 | -0.792479656 | 8.805787521 | -5.051138131 | 6.31E-05 | 0.000452331 | 1.613972463 |
| EPC1 | 1.639717221 | 9.360350618 | 5.051118207 | 6.31E-05 | 0.000452331 | 1.613927443 |
| KDELC2 | 1.526474385 | 6.437218569 | 5.050705232 | 6.32E-05 | 0.0004524 | 1.612994321 |
| TAOK2 | -1.576842914 | 8.901575625 | -5.050694247 | 6.32E-05 | 0.0004524 | 1.612969499 |
| NOTO | -2.982694884 | 5.260514074 | -5.048421331 | 6.35E-05 | 0.000454566 | 1.607833603 |
| OGFOD2 | -0.645224688 | 10.26336334 | -5.048240188 | 6.35E-05 | 0.000454568 | 1.607424274 |
| RPAP2 | 1.006349796 | 7.823233958 | 5.047479837 | 6.36E-05 | 0.000455172 | 1.605706092 |
| TOB1 | 1.401281565 | 9.947360615 | 5.046492214 | 6.38E-05 | 0.000456013 | 1.603474276 |
| SUPT6H | -0.683068031 | 10.22058821 | -5.044823837 | 6.40E-05 | 0.000457565 | 1.59970395 |
| PML | -1.077087791 | 11.02949077 | -5.044411748 | 6.41E-05 | 0.000457809 | 1.59877265 |
| CSRP1 | -1.106070945 | 7.706623555 | -5.04391581 | 6.41E-05 | 0.000458141 | 1.597651841 |
| CLDND1 | 1.075195355 | 11.05067647 | 5.043298405 | 6.42E-05 | 0.000458452 | 1.596256495 |
| ORC3 | 0.763906204 | 9.30430253 | 5.04323198 | 6.42E-05 | 0.000458452 | 1.596106373 |
| GATM | 1.71666761 | 5.449636229 | 5.043084426 | 6.43E-05 | 0.000458452 | 1.595772894 |
| ARL6 | 1.58254807 | 3.06077289 | 5.042027684 | 6.44E-05 | 0.000459372 | 1.593384562 |
| COL27A1 | -1.651600291 | 7.558411547 | -5.041634868 | 6.45E-05 | 0.000459389 | 1.592496745 |
| NFKB1 | -0.787445925 | 11.98257349 | -5.041500346 | 6.45E-05 | 0.000459389 | 1.592192704 |
| RLF | 0.852448439 | 9.698993385 | 5.041387028 | 6.45E-05 | 0.000459389 | 1.591936585 |
| GJA4 | -1.666509939 | 5.202031245 | -5.041300429 | 6.45E-05 | 0.000459389 | 1.591740858 |
| NLE1 | -0.980969988 | 8.490313306 | -5.040400592 | 6.47E-05 | 0.000460147 | 1.58970704 |
| MFSD1 | 0.771890586 | 11.70619821 | 5.040132067 | 6.47E-05 | 0.000460243 | 1.589100108 |
| SIAH1 | 1.035763265 | 7.524909943 | 5.038026914 | 6.50E-05 | 0.000462271 | 1.584341775 |
| ZNF709 | 1.175111348 | 7.087355362 | 5.037574636 | 6.51E-05 | 0.000462561 | 1.583319439 |
| AKT3 | 1.259622365 | 8.097904909 | 5.037367771 | 6.51E-05 | 0.000462593 | 1.582851833 |
| TSEN15 | 1.274961627 | 7.077542327 | 5.033480552 | 6.57E-05 | 0.000466525 | 1.574064479 |
| SH2D1A | 1.545012729 | 8.481025006 | 5.031430111 | 6.60E-05 | 0.000468523 | 1.569428888 |
| C2 | -1.297937451 | 7.066189069 | -5.030377992 | 6.62E-05 | 0.000469312 | 1.56705017 |
| ZNF700 | 1.655620166 | 6.439134936 | 5.030338961 | 6.62E-05 | 0.000469312 | 1.566961924 |
| ABCA10 | 1.272474265 | 2.845961694 | 5.030008295 | 6.62E-05 | 0.000469392 | 1.566214312 |
| SLC9B2 | 0.914599209 | 5.117709746 | 5.029911129 | 6.62E-05 | 0.000469392 | 1.565994623 |
| NLRX1 | -0.838131431 | 11.85977385 | -5.028971896 | 6.64E-05 | 0.00047021 | 1.563871036 |
| KIRREL2 | -1.535129467 | 8.733599967 | -5.027982821 | 6.65E-05 | 0.000471082 | 1.561634691 |
| HM13 | -0.85621562 | 9.695628588 | -5.027298837 | 6.66E-05 | 0.000471628 | 1.560088132 |
| PLEKHA1 | 0.969050277 | 9.822496872 | 5.026793071 | 6.67E-05 | 0.000471983 | 1.558944523 |
| NIN | 0.835697775 | 8.583831706 | 5.026353111 | 6.68E-05 | 0.000472267 | 1.557949696 |
| RPRD1A | 0.782983101 | 8.587139452 | 5.025384503 | 6.69E-05 | 0.000473122 | 1.555759457 |
| SLFN11 | 0.834891184 | 10.13855653 | 5.024468713 | 6.71E-05 | 0.000473921 | 1.553688594 |
| ELAVL1 | 1.109038873 | 6.648656106 | 5.023562735 | 6.72E-05 | 0.000474523 | 1.551639863 |
| FAM175A | 1.026194998 | 7.367805889 | 5.023560774 | 6.72E-05 | 0.000474523 | 1.551635431 |
| PNPLA8 | 1.020460974 | 10.10146945 | 5.022140658 | 6.74E-05 | 0.000475873 | 1.548423944 |
| CHST11 | -0.97376075 | 8.384629618 | -5.021158769 | 6.76E-05 | 0.000476749 | 1.546203396 |
| DNAL1 | 1.923757103 | 4.894096703 | 5.019995166 | 6.77E-05 | 0.000477825 | 1.543571819 |
| MARCKS | 0.836594245 | 11.88333038 | 5.019595237 | 6.78E-05 | 0.00047807 | 1.542667326 |
| OTOF | -0.960882581 | 10.05171243 | -5.019260529 | 6.79E-05 | 0.000478243 | 1.541910333 |
| SNX9 | 1.683336552 | 3.824059763 | 5.018529577 | 6.80E-05 | 0.00047885 | 1.540257146 |
| RAP1A | 0.94484232 | 12.55983794 | 5.017862168 | 6.81E-05 | 0.000479388 | 1.538747644 |
| RECQL5 | -0.924762024 | 7.131859725 | -5.017066434 | 6.82E-05 | 0.000480068 | 1.536947865 |
| RPS7 | 1.997677357 | 14.721897 | 5.016804505 | 6.82E-05 | 0.000480163 | 1.536355429 |
| RPS6KC1 | 0.581484161 | 7.528506688 | 5.016204378 | 6.83E-05 | 0.000480629 | 1.534998032 |
| GPR37L1 | -3.31214483 | 5.886367578 | -5.015117599 | 6.85E-05 | 0.0004816 | 1.532539841 |
| CLDN6 | -2.564217519 | 5.484271243 | -5.014970199 | 6.85E-05 | 0.0004816 | 1.53220643 |
| GRB14 | -1.054832777 | 5.50977461 | -5.014695144 | 6.86E-05 | 0.00048171 | 1.531584265 |
| ZAN | -3.044337432 | 8.277468451 | -5.013158166 | 6.88E-05 | 0.000483091 | 1.528107592 |
| SH3BGRL | 0.847388541 | 12.63566817 | 5.013091173 | 6.88E-05 | 0.000483091 | 1.527956049 |
| ENG | -1.031029161 | 7.137230159 | -5.012004894 | 6.90E-05 | 0.000484097 | 1.525498764 |
| C16orf95 | -0.86153658 | 8.046674534 | -5.010368696 | 6.92E-05 | 0.000485716 | 1.521797355 |
| RTBDN | -1.445368497 | 7.519170398 | -5.009263054 | 6.94E-05 | 0.000486674 | 1.519296068 |
| TUBB1 | -1.459667054 | 9.516849435 | -5.009156295 | 6.94E-05 | 0.000486674 | 1.519054544 |
| PCGF1 | -0.69619108 | 8.770043269 | -5.006916047 | 6.98E-05 | 0.000488975 | 1.513986187 |
| MORC4 | 1.042957504 | 7.649786127 | 5.005810138 | 7.00E-05 | 0.000490017 | 1.511484046 |
| FAM122C | 0.907721342 | 5.420108689 | 5.005429602 | 7.00E-05 | 0.000490248 | 1.510623056 |
| WDR7 | 0.714131751 | 9.523515319 | 5.005208425 | 7.01E-05 | 0.0004903 | 1.510122624 |
| TATDN1 | 1.719424344 | 5.567469388 | 5.002710117 | 7.05E-05 | 0.00049291 | 1.504469759 |
| PNISR | 1.862407906 | 7.177053994 | 5.000783756 | 7.08E-05 | 0.000494887 | 1.500110746 |
| RPL10A | 0.643890344 | 16.24071222 | 5.0003763 | 7.08E-05 | 0.000494985 | 1.499188714 |
| N4BP2L1 | 1.09462331 | 7.65870864 | 5.000348373 | 7.08E-05 | 0.000494985 | 1.499125518 |
| SFTPC | -1.185590064 | 6.861126735 | -4.999125027 | 7.10E-05 | 0.000496173 | 1.496357144 |
| FBXO5 | 1.738716931 | 6.077902198 | 4.998692796 | 7.11E-05 | 0.000496467 | 1.495379001 |
| TMEM104 | -0.617640585 | 10.08240219 | -4.997021613 | 7.14E-05 | 0.000498168 | 1.491596986 |
| SQLE | 1.196012543 | 7.207010102 | 4.995111769 | 7.17E-05 | 0.000500018 | 1.487274642 |
| ZNF420 | 0.879615225 | 8.473212144 | 4.994963995 | 7.17E-05 | 0.000500018 | 1.486940192 |
| SNX10 | 1.322311851 | 9.668378394 | 4.994877903 | 7.17E-05 | 0.000500018 | 1.486745343 |
| MAPK3 | -1.245305708 | 8.695972538 | -4.994638647 | 7.18E-05 | 0.000500094 | 1.486203839 |
| OTOP1 | -1.324880977 | 5.56059343 | -4.993550023 | 7.20E-05 | 0.000500974 | 1.483739935 |
| CCZ1 | 0.715032469 | 9.993293069 | 4.993382041 | 7.20E-05 | 0.000500974 | 1.483359732 |
| RPL6 | 1.033420956 | 15.84432342 | 4.993348866 | 7.20E-05 | 0.000500974 | 1.483284643 |
| PBRM1 | 0.75409082 | 9.341504194 | 4.991333653 | 7.23E-05 | 0.000503087 | 1.478723343 |
| ZMYM2 | 0.71320087 | 9.749401672 | 4.991041757 | 7.24E-05 | 0.000503224 | 1.478062635 |
| PAIP1 | 1.000932826 | 7.65400575 | 4.989900472 | 7.26E-05 | 0.000504338 | 1.475479274 |
| ORC4 | 0.797007985 | 8.071962199 | 4.989727703 | 7.26E-05 | 0.000504338 | 1.475088193 |
| SLC24A3 | -1.707259724 | 7.460873848 | -4.988288253 | 7.28E-05 | 0.0005058 | 1.471829786 |
| PYROXD2 | -0.71373801 | 8.131852143 | -4.987633924 | 7.29E-05 | 0.000506357 | 1.470348569 |
| ZNF791 | 0.844702742 | 7.016206485 | 4.987196286 | 7.30E-05 | 0.000506664 | 1.469357867 |
| DDX5 | 0.838493169 | 14.50065315 | 4.986405824 | 7.31E-05 | 0.00050738 | 1.467568426 |
| ZMYM6 | 0.876785027 | 7.373164804 | 4.985229115 | 7.33E-05 | 0.000508546 | 1.464904529 |
| SGOL2 | 1.948603674 | 4.365015736 | 4.983803212 | 7.36E-05 | 0.000509932 | 1.461676375 |
| SRXN1 | -1.221852182 | 10.93481197 | -4.983633621 | 7.36E-05 | 0.000509932 | 1.461292424 |
| GCSH | 0.702460802 | 8.174086299 | 4.983521033 | 7.36E-05 | 0.000509932 | 1.461037524 |
| HPS5 | 0.898461493 | 8.761790581 | 4.982242515 | 7.38E-05 | 0.000511223 | 1.458142905 |
| LYL1 | -2.134339067 | 10.54728516 | -4.981773851 | 7.39E-05 | 0.000511569 | 1.457081804 |
| HSP90AA1 | 1.961892503 | 11.84927043 | 4.980721061 | 7.41E-05 | 0.0005126 | 1.454698136 |
| NKAPL | -1.737711936 | 7.320700992 | -4.980415289 | 7.42E-05 | 0.000512605 | 1.454005809 |
| KDM4A | -0.612883309 | 8.744719114 | -4.980260007 | 7.42E-05 | 0.000512605 | 1.45365422 |
| KRTAP3-1 | -1.404935975 | 6.34887692 | -4.980047632 | 7.42E-05 | 0.000512605 | 1.453173357 |
| UBE2T | 1.051916862 | 7.707933101 | 4.980029118 | 7.42E-05 | 0.000512605 | 1.453131438 |
| TMEM53 | -1.448778877 | 6.211845944 | -4.976735212 | 7.48E-05 | 0.000516277 | 1.445672953 |
| CEP57 | 1.183722214 | 8.41416997 | 4.975929907 | 7.49E-05 | 0.000517025 | 1.443849375 |
| TSHZ1 | 0.755662573 | 8.814305628 | 4.97397388 | 7.53E-05 | 0.000519139 | 1.439419862 |
| ZFAT | -0.779700666 | 9.07780817 | -4.973058586 | 7.54E-05 | 0.000519989 | 1.437347055 |
| YEATS4 | 1.052104638 | 8.665651537 | 4.972915353 | 7.54E-05 | 0.000519989 | 1.43702268 |
| FRMD4A | -0.813896165 | 4.762916888 | -4.972127733 | 7.56E-05 | 0.000520722 | 1.435238957 |
| NOX1 | -1.131299674 | 4.586169611 | -4.971251118 | 7.57E-05 | 0.000521408 | 1.433253642 |
| B9D2 | -0.943389564 | 11.38710867 | -4.971209503 | 7.57E-05 | 0.000521408 | 1.433159393 |
| PACRG | -2.103767133 | 7.559755548 | -4.968632428 | 7.62E-05 | 0.000524145 | 1.427322673 |
| USO1 | 0.869771328 | 10.58018474 | 4.968578274 | 7.62E-05 | 0.000524145 | 1.427200017 |
| RDH10 | 1.710986271 | 5.238352642 | 4.968005311 | 7.63E-05 | 0.000524627 | 1.425902275 |
| RHOQ | 0.78174548 | 10.27304319 | 4.967130631 | 7.64E-05 | 0.000525472 | 1.423921116 |
| TCFL5 | 0.617015613 | 7.719413042 | 4.966886625 | 7.65E-05 | 0.00052556 | 1.423368431 |
| ADORA3 | -0.997587488 | 6.203745058 | -4.965930164 | 7.66E-05 | 0.000526505 | 1.421201969 |
| TM9SF3 | 1.058658406 | 8.269081843 | 4.965757758 | 7.67E-05 | 0.000526507 | 1.420811449 |
| UBXN7 | 1.584639799 | 5.752519595 | 4.965474857 | 7.67E-05 | 0.000526642 | 1.42017064 |
| CPA3 | -1.399266013 | 7.664112322 | -4.96472193 | 7.69E-05 | 0.000527344 | 1.418465137 |
| C12orf49 | 0.679809357 | 7.116690676 | 4.964015172 | 7.70E-05 | 0.000527991 | 1.416864182 |
| EBF4 | -1.083863568 | 7.473229894 | -4.962946243 | 7.72E-05 | 0.000529077 | 1.414442774 |
| RHOU | 1.337486646 | 7.880255435 | 4.962267935 | 7.73E-05 | 0.000529692 | 1.412906189 |
| TMEM170A | 0.603508137 | 8.545219124 | 4.960697956 | 7.76E-05 | 0.000531391 | 1.409349575 |
| TMOD4 | -0.804727697 | 7.714206614 | -4.960202115 | 7.77E-05 | 0.000531787 | 1.408226269 |
| SMARCA5 | 1.586988658 | 6.978012975 | 4.959389697 | 7.78E-05 | 0.000532568 | 1.406385741 |
| ORC6 | 0.945518751 | 4.113010957 | 4.959029949 | 7.79E-05 | 0.00053276 | 1.405570721 |
| UBE2D2 | 0.775628094 | 9.93798115 | 4.958892742 | 7.79E-05 | 0.00053276 | 1.405259872 |
| TOMM7 | 2.00718161 | 14.05287546 | 4.958492327 | 7.80E-05 | 0.00053304 | 1.404352706 |
| C9orf66 | 1.450978269 | 4.164685777 | 4.957537409 | 7.81E-05 | 0.000533999 | 1.40218924 |
| IFNB1 | -1.754371081 | 3.6764978 | -4.957105134 | 7.82E-05 | 0.000534319 | 1.401209857 |
| IL7R | 1.210363644 | 14.14272055 | 4.955951163 | 7.84E-05 | 0.000535524 | 1.398595309 |
| AZIN1 | 1.042800522 | 9.429355888 | 4.955129732 | 7.86E-05 | 0.000536323 | 1.396734146 |
| EGR2 | 2.419109218 | 5.456704685 | 4.954109655 | 7.87E-05 | 0.000537182 | 1.39442284 |
| ZRANB2 | 2.908770219 | 10.53512517 | 4.954091279 | 7.88E-05 | 0.000537182 | 1.394381202 |
| ATPIF1 | 0.959214058 | 12.93141696 | 4.953455792 | 7.89E-05 | 0.000537569 | 1.392941274 |
| TMEM56-RWDD3 | 1.656148407 | 6.207482422 | 4.953437366 | 7.89E-05 | 0.000537569 | 1.392899524 |
| C1orf64 | -3.275350216 | 5.866995264 | -4.950825099 | 7.93E-05 | 0.000540379 | 1.386980212 |
| MOCS2 | 1.396447274 | 6.868210677 | 4.950797866 | 7.93E-05 | 0.000540379 | 1.3869185 |
| ZNF184 | 1.520919638 | 6.571918583 | 4.950651234 | 7.94E-05 | 0.000540379 | 1.386586224 |
| PDK3 | -0.730056147 | 8.16057867 | -4.950120703 | 7.95E-05 | 0.000540826 | 1.385384003 |
| SULT1A2 | -0.801986689 | 12.14755636 | -4.949861543 | 7.95E-05 | 0.000540937 | 1.384796719 |
| NRN1L | -0.754401732 | 7.769772582 | -4.948449334 | 7.98E-05 | 0.000542376 | 1.381596443 |
| LTA | -2.409004814 | 7.784471796 | -4.948362832 | 7.98E-05 | 0.000542376 | 1.381400412 |
| COMMD3 | 1.108652544 | 12.32560405 | 4.948063472 | 7.98E-05 | 0.000542538 | 1.380722001 |
| ZNF93 | 0.950221948 | 6.650168857 | 4.947620962 | 7.99E-05 | 0.000542878 | 1.379719171 |
| RPL39 | 1.935288084 | 14.26378433 | 4.947085417 | 8.00E-05 | 0.000543334 | 1.378505487 |
| TCF12 | 0.948519098 | 8.469938352 | 4.94543681 | 8.03E-05 | 0.000544994 | 1.374769206 |
| DENND1B | 0.721701139 | 7.468842829 | 4.94541499 | 8.03E-05 | 0.000544994 | 1.374719753 |
| IL37 | -2.075044403 | 6.026058106 | -4.944643468 | 8.05E-05 | 0.000545747 | 1.372971176 |
| PYROXD1 | 0.956721067 | 8.752148448 | 4.944378114 | 8.05E-05 | 0.000545836 | 1.372369769 |
| CCIN | -1.352875811 | 7.212857667 | -4.944149539 | 8.06E-05 | 0.000545836 | 1.371851716 |
| MACF1 | 1.208238084 | 9.484057522 | 4.944067503 | 8.06E-05 | 0.000545836 | 1.371665785 |
| POLE2 | 1.221214087 | 4.748465208 | 4.943535896 | 8.07E-05 | 0.00054629 | 1.37046091 |
| ATP1B2 | -1.651534637 | 7.060391165 | -4.94194052 | 8.10E-05 | 0.000548077 | 1.366844927 |
| PPP6C | 0.642098035 | 10.19758798 | 4.941581533 | 8.10E-05 | 0.000548317 | 1.366031247 |
| PARP8 | 1.0515211 | 10.01120483 | 4.939944374 | 8.13E-05 | 0.000549975 | 1.362320366 |
| NAT10 | -2.412174296 | 10.47494692 | -4.93992597 | 8.13E-05 | 0.000549975 | 1.362278648 |
| ADRM1 | -1.077679683 | 10.46351573 | -4.939534419 | 8.14E-05 | 0.000550257 | 1.361391111 |
| JKAMP | 0.908419009 | 9.186556092 | 4.938201834 | 8.17E-05 | 0.000551589 | 1.358370443 |
| VPS4B | 0.854987532 | 10.90575824 | 4.938142352 | 8.17E-05 | 0.000551589 | 1.358235607 |
| NFKBIB | -1.017856553 | 7.670316411 | -4.93795932 | 8.17E-05 | 0.000551609 | 1.357820706 |
| FOXN1 | -2.157461816 | 5.923102353 | -4.937153574 | 8.19E-05 | 0.000552415 | 1.355994198 |
| BET1 | 0.895833978 | 7.147472321 | 4.935590946 | 8.22E-05 | 0.000554182 | 1.352451838 |
| SLC19A1 | -1.062936622 | 9.369038605 | -4.934402853 | 8.24E-05 | 0.000555479 | 1.349758423 |
| MPP1 | -1.361300602 | 13.51178249 | -4.933509219 | 8.26E-05 | 0.000556403 | 1.347732495 |
| DENND1C | -0.662498606 | 12.01347728 | -4.932850643 | 8.27E-05 | 0.000557029 | 1.346239427 |
| PTER | 2.117779202 | 6.617829599 | 4.932441771 | 8.28E-05 | 0.000557338 | 1.345312454 |
| DSEL | 1.569843412 | 4.275954743 | 4.932127355 | 8.28E-05 | 0.000557526 | 1.344599618 |
| FANCB | 1.346507875 | 5.252396648 | 4.931661017 | 8.29E-05 | 0.000557908 | 1.343542342 |
| AQP5 | -2.142699364 | 7.491748492 | -4.931217723 | 8.30E-05 | 0.000558058 | 1.342537297 |
| RPN2 | 0.607268085 | 11.70923054 | 4.931001123 | 8.30E-05 | 0.000558058 | 1.342046212 |
| EIF2S1 | 0.777156396 | 7.969148336 | 4.930886146 | 8.30E-05 | 0.000558058 | 1.341785531 |
| RIMKLB | 1.043445092 | 4.84758019 | 4.930875442 | 8.31E-05 | 0.000558058 | 1.341761261 |
| SMG1 | 1.740433816 | 9.766850795 | 4.930161183 | 8.32E-05 | 0.000558758 | 1.340141841 |
| NUP214 | -1.112851808 | 9.808453883 | -4.928403991 | 8.35E-05 | 0.00056063 | 1.336157675 |
| SH3BGRL2 | -1.421737107 | 6.689275364 | -4.928367126 | 8.35E-05 | 0.00056063 | 1.336074087 |
| RNF223 | -2.743085608 | 7.945834391 | -4.925433238 | 8.41E-05 | 0.000564194 | 1.329421528 |
| SEC11A | 1.048327306 | 12.53484143 | 4.92379609 | 8.44E-05 | 0.000566098 | 1.325709091 |
| DUXA | -2.79684759 | 5.778818783 | -4.923074 | 8.45E-05 | 0.000566819 | 1.324071611 |
| CCDC88C | 0.635678139 | 9.116295695 | 4.920804545 | 8.50E-05 | 0.000569425 | 1.318924981 |
| TTBK2 | 0.847190855 | 9.378537225 | 4.920734958 | 8.50E-05 | 0.000569425 | 1.318767167 |
| LRCH4 | -1.206216569 | 10.81634189 | -4.920572124 | 8.50E-05 | 0.000569425 | 1.318397883 |
| SERF2 | -0.729427499 | 14.55786569 | -4.919809101 | 8.52E-05 | 0.000570204 | 1.316667434 |
| TBC1D22B | -1.421300645 | 7.927152905 | -4.918799312 | 8.54E-05 | 0.000571307 | 1.314377297 |
| MGA | 0.837507328 | 8.987660923 | 4.918542803 | 8.54E-05 | 0.000571426 | 1.31379554 |
| HELLS | 1.680445096 | 5.664431198 | 4.918114357 | 8.55E-05 | 0.000571646 | 1.312823826 |
| OMG | 1.694554271 | 4.151484794 | 4.918042452 | 8.55E-05 | 0.000571646 | 1.312660745 |
| NID2 | -1.246978851 | 5.590732697 | -4.91781268 | 8.56E-05 | 0.00057173 | 1.312139619 |
| ZNF610 | 1.742324324 | 3.520452755 | 4.916871808 | 8.58E-05 | 0.000572746 | 1.310005672 |
| TMEM133 | 1.59890142 | 3.444258168 | 4.91637877 | 8.59E-05 | 0.000573175 | 1.308887415 |
| ZC3H12C | 1.416911699 | 4.264042876 | 4.915985467 | 8.59E-05 | 0.000573474 | 1.307995357 |
| VPS33B | -0.764969821 | 8.026676882 | -4.915197582 | 8.61E-05 | 0.000574293 | 1.306208312 |
| PANK1 | 1.309313215 | 3.967686174 | 4.914891453 | 8.61E-05 | 0.000574478 | 1.305513955 |
| EXTL3 | -0.648746271 | 12.34525557 | -4.908881214 | 8.73E-05 | 0.000582223 | 1.29188051 |
| RNF103 | 0.896905379 | 8.374607711 | 4.908420981 | 8.74E-05 | 0.000582588 | 1.290836446 |
| TTC30B | 1.353899315 | 5.751736273 | 4.908277263 | 8.75E-05 | 0.000582588 | 1.290510409 |
| HCAR2 | -1.717263556 | 8.209997917 | -4.907445603 | 8.76E-05 | 0.000583478 | 1.288623703 |
| C9orf16 | -0.972977618 | 10.03008562 | -4.90709635 | 8.77E-05 | 0.000583724 | 1.287831375 |
| SNRPF | 0.734100262 | 11.75853719 | 4.906931571 | 8.77E-05 | 0.000583724 | 1.287457549 |
| PPM1B | 1.202443683 | 8.812851581 | 4.904894917 | 8.81E-05 | 0.000586233 | 1.282836963 |
| CHMP2A | -0.587264962 | 14.24106536 | -4.904395992 | 8.82E-05 | 0.000586682 | 1.281705008 |
| C1orf116 | -2.324063482 | 5.868958052 | -4.902389301 | 8.87E-05 | 0.000588945 | 1.277152107 |
| CCDC18 | 1.103906882 | 5.965334705 | 4.902386741 | 8.87E-05 | 0.000588945 | 1.277146299 |
| DNAJC2 | 0.990266372 | 9.998538171 | 4.902104979 | 8.87E-05 | 0.000589102 | 1.276507002 |
| IKZF2 | 1.511613064 | 7.443552026 | 4.901247153 | 8.89E-05 | 0.000589957 | 1.27456063 |
| FTH1 | -0.688069177 | 16.58359516 | -4.901142899 | 8.89E-05 | 0.000589957 | 1.274324079 |
| CTLA4 | 1.373637839 | 7.558351251 | 4.900891548 | 8.90E-05 | 0.000590075 | 1.273753766 |
| IFT74 | 1.19873056 | 4.666089752 | 4.900320632 | 8.91E-05 | 0.000590625 | 1.272458348 |
| SLAMF6 | 1.79306798 | 7.677142179 | 4.89923102 | 8.93E-05 | 0.000591878 | 1.269985945 |
| CCDC78 | -2.701775462 | 12.48942406 | -4.897987654 | 8.95E-05 | 0.000593344 | 1.267164582 |
| GABPB1 | 0.635684435 | 7.366478843 | 4.897578999 | 8.96E-05 | 0.000593677 | 1.266237271 |
| ZNF664 | 0.96853116 | 9.267405865 | 4.896735108 | 8.98E-05 | 0.000594602 | 1.264322299 |
| PCDH9 | 1.900051853 | 3.928111998 | 4.896514775 | 8.99E-05 | 0.000594679 | 1.26382231 |
| FXYD4 | -1.8311492 | 4.912843986 | -4.896090118 | 8.99E-05 | 0.000595034 | 1.262858652 |
| ZBTB2 | 1.217337664 | 8.613182254 | 4.895777599 | 9.00E-05 | 0.000595237 | 1.262149458 |
| LRRC25 | -0.910517103 | 12.09804878 | -4.895411012 | 9.01E-05 | 0.000595513 | 1.261317561 |
| SIGLEC5 | -1.486695196 | 10.20293144 | -4.894143199 | 9.03E-05 | 0.000597022 | 1.258440449 |
| NRAS | 1.643295848 | 6.374815625 | 4.893099559 | 9.06E-05 | 0.000598228 | 1.256071995 |
| HIST1H3D | 1.882752163 | 11.59293988 | 4.888864984 | 9.14E-05 | 0.000603838 | 1.246461357 |
| EML5 | 1.226829978 | 3.873685741 | 4.888251222 | 9.16E-05 | 0.000604461 | 1.245068302 |
| PIGW | 1.442004405 | 4.147331572 | 4.88757594 | 9.17E-05 | 0.000605171 | 1.24353559 |
| KCNK3 | -1.894935424 | 5.058745876 | -4.887151968 | 9.18E-05 | 0.000605532 | 1.242573271 |
| NLRP1 | -0.821698429 | 9.90115657 | -4.886412457 | 9.20E-05 | 0.000606116 | 1.240894731 |
| RPL26 | 2.404171039 | 14.18607193 | 4.886404763 | 9.20E-05 | 0.000606116 | 1.240877266 |
| PIK3CA | 1.26911233 | 8.702250875 | 4.885018774 | 9.23E-05 | 0.000607818 | 1.237731267 |
| KRTAP6-3 | -2.779970031 | 7.066974277 | -4.884844997 | 9.23E-05 | 0.000607832 | 1.23733681 |
| MRPL3 | 1.363150833 | 8.889975902 | 4.884239473 | 9.24E-05 | 0.000608449 | 1.235962315 |
| RGS6 | -1.039201782 | 3.642098807 | -4.883827836 | 9.25E-05 | 0.000608796 | 1.235027919 |
| ZNF460 | 1.80421788 | 4.607461226 | 4.883215543 | 9.26E-05 | 0.000609423 | 1.233638025 |
| NOL6 | -0.861893022 | 7.303830474 | -4.879639973 | 9.34E-05 | 0.000614212 | 1.225521135 |
| PTPRA | 0.569077838 | 11.10821251 | 4.877292601 | 9.39E-05 | 0.000617298 | 1.220191987 |
| PINK1 | -1.512506079 | 11.72422073 | -4.875163134 | 9.44E-05 | 0.000620089 | 1.215357279 |
| DEFB4A | -1.816172111 | 7.644408607 | -4.873813727 | 9.47E-05 | 0.000621779 | 1.212293477 |
| ZNF286B | 0.877029789 | 8.352899679 | 4.87168098 | 9.51E-05 | 0.000624596 | 1.207450915 |
| SLC25A12 | 0.817689504 | 9.337921425 | 4.871507869 | 9.52E-05 | 0.00062461 | 1.207057843 |
| ZFP41 | -1.844113891 | 9.399405754 | -4.870489357 | 9.54E-05 | 0.000625838 | 1.204745138 |
| PCDHB7 | -1.50553947 | 2.898211952 | -4.869588938 | 9.56E-05 | 0.000626728 | 1.202700536 |
| RIMS4 | -1.71656715 | 4.987093614 | -4.869544965 | 9.56E-05 | 0.000626728 | 1.202600684 |
| PLEKHO2 | -1.055656551 | 12.95258521 | -4.86910003 | 9.57E-05 | 0.000626914 | 1.201590344 |
| KLHL11 | 1.777574634 | 5.908004524 | 4.869090522 | 9.57E-05 | 0.000626914 | 1.201568752 |
| ROGDI | -1.181999081 | 7.766572368 | -4.868765098 | 9.58E-05 | 0.000627149 | 1.200829785 |
| IFIT1B | -2.726324745 | 10.91825239 | -4.868203339 | 9.59E-05 | 0.000627724 | 1.199554138 |
| CASC3 | -0.966273557 | 12.89126494 | -4.867422171 | 9.61E-05 | 0.000628616 | 1.197780229 |
| SYTL4 | -1.257651143 | 4.532635228 | -4.867103718 | 9.61E-05 | 0.000628715 | 1.197057062 |
| GTF2F2 | 1.200524308 | 8.857954778 | 4.867029864 | 9.61E-05 | 0.000628715 | 1.196889348 |
| VDR | -0.801920426 | 10.1020985 | -4.866092873 | 9.63E-05 | 0.000629834 | 1.194761526 |
| TTLL4 | -1.332824307 | 8.840318288 | -4.865262757 | 9.65E-05 | 0.0006308 | 1.192876364 |
| RAB11FIP4 | -0.770962628 | 10.9157669 | -4.864846175 | 9.66E-05 | 0.000631169 | 1.19193031 |
| C5orf30 | 1.067152792 | 6.907482408 | 4.864395141 | 9.67E-05 | 0.000631588 | 1.190906006 |
| TCTE3 | 1.35002032 | 4.040286545 | 4.863705069 | 9.69E-05 | 0.000632354 | 1.189338818 |
| FAM181A | -2.859847345 | 9.047585266 | -4.863483629 | 9.69E-05 | 0.000632406 | 1.188835914 |
| HYAL3 | -1.037517804 | 8.158093366 | -4.863345925 | 9.70E-05 | 0.000632406 | 1.188523176 |
| SLC6A7 | -1.3145611 | 3.129244805 | -4.861668022 | 9.73E-05 | 0.000634611 | 1.184712439 |
| THRB | -1.281718066 | 2.90338642 | -4.860754569 | 9.75E-05 | 0.000635707 | 1.182637807 |
| NPSR1 | -1.881757693 | 4.45323682 | -4.859824982 | 9.77E-05 | 0.000636829 | 1.180526483 |
| DZIP3 | 1.227348586 | 7.457021105 | 4.858886141 | 9.80E-05 | 0.000637966 | 1.178394094 |
| RCOR3 | 0.819503647 | 8.198004934 | 4.857473805 | 9.83E-05 | 0.000639708 | 1.175186167 |
| ZNF419 | 1.354950465 | 5.843446845 | 4.857375981 | 9.83E-05 | 0.000639708 | 1.174963972 |
| PLSCR5 | -1.415665686 | 3.083560358 | -4.857156764 | 9.83E-05 | 0.000639793 | 1.17446604 |
| MAP3K2 | 1.103713445 | 8.26647777 | 4.856628332 | 9.85E-05 | 0.00064017 | 1.173265742 |
| TRAT1 | 1.743313562 | 9.287286195 | 4.856577942 | 9.85E-05 | 0.00064017 | 1.173151285 |
| EPS15 | 0.775230812 | 11.00656064 | 4.854807264 | 9.89E-05 | 0.00064254 | 1.169129198 |
| UBE2G1 | 0.630506698 | 9.852298431 | 4.854093561 | 9.90E-05 | 0.000643182 | 1.167507981 |
| CADM1 | 1.575938525 | 4.500153309 | 4.854037675 | 9.90E-05 | 0.000643182 | 1.167381031 |
| SLC39A9 | 1.803192482 | 5.741974373 | 4.853753973 | 9.91E-05 | 0.000643182 | 1.166736576 |
| LEFTY1 | -1.829281057 | 6.36846896 | -4.853728548 | 9.91E-05 | 0.000643182 | 1.166678821 |
| FAM45A | 0.75510522 | 9.975574269 | 4.853515676 | 9.92E-05 | 0.000643259 | 1.16619526 |
| EEF1B2 | 1.827990234 | 13.82073679 | 4.852622575 | 9.94E-05 | 0.000644342 | 1.164166461 |
| ATP11A | 0.968617327 | 9.99003043 | 4.851284992 | 9.97E-05 | 0.000646085 | 1.161127884 |
| MTFR1 | 0.938083969 | 4.226869957 | 4.84990836 | 1.00E-04 | 0.000647693 | 1.158000502 |
| PRKAB2 | 0.962237051 | 8.44471487 | 4.849881211 | 1.00E-04 | 0.000647693 | 1.157938823 |
| NSUN4 | -0.83043031 | 9.178253842 | -4.849095599 | 0.00010018 | 0.000648487 | 1.156154053 |
| NCK1 | 1.006396904 | 9.725315279 | 4.849027029 | 0.000100196 | 0.000648487 | 1.155998274 |
| SENP8 | 1.025956044 | 5.453616087 | 4.847667947 | 0.000100509 | 0.000650274 | 1.1529106 |
| MCEE | 0.919936069 | 9.392233958 | 4.846483821 | 0.000100782 | 0.000651805 | 1.150220324 |
| NCEH1 | 1.177764513 | 6.978844548 | 4.846084386 | 0.000100875 | 0.000652163 | 1.149312812 |
| PUM2 | 1.764688899 | 9.351971323 | 4.844005499 | 0.000101358 | 0.000655043 | 1.144589467 |
| SCNN1D | -1.164397044 | 6.764750524 | -4.843724111 | 0.000101423 | 0.000655083 | 1.14395012 |
| E2F3 | 1.212758444 | 8.636092286 | 4.843658967 | 0.000101438 | 0.000655083 | 1.143802106 |
| PHAX | 1.300088065 | 9.381665773 | 4.842923512 | 0.00010161 | 0.00065595 | 1.142131045 |
| PCNXL2 | -0.995880872 | 10.18135282 | -4.841830755 | 0.000101865 | 0.000657357 | 1.139648088 |
| PTGR1 | 1.364820821 | 5.551704185 | 4.841503245 | 0.000101942 | 0.00065761 | 1.13890391 |
| MYO5C | 1.292550822 | 3.518670847 | 4.841270199 | 0.000101996 | 0.000657721 | 1.138374372 |
| WAS | -0.674202678 | 10.56691273 | -4.840307036 | 0.000102222 | 0.000658936 | 1.1361858 |
| INTS6 | 0.941030357 | 7.328412262 | 4.839856129 | 0.000102328 | 0.000659377 | 1.135161196 |
| STRN3 | 1.82867331 | 4.513037877 | 4.839673419 | 0.000102371 | 0.000659412 | 1.134746021 |
| CCDC103 | -0.972602212 | 4.499390235 | -4.839205254 | 0.000102481 | 0.000659558 | 1.133682187 |
| NRIP3 | -1.015473529 | 5.81045233 | -4.839135033 | 0.000102498 | 0.000659558 | 1.133522618 |
| ADNP2 | 1.416745942 | 6.308483318 | 4.839098824 | 0.000102506 | 0.000659558 | 1.133440338 |
| GNB5 | 0.982327035 | 6.594256764 | 4.838833871 | 0.000102569 | 0.000659718 | 1.132838269 |
| BTBD2 | -1.898369459 | 9.486750117 | -4.838588947 | 0.000102626 | 0.000659848 | 1.132281706 |
| SLC16A14 | 1.111709874 | 2.764197127 | 4.837144041 | 0.000102967 | 0.000661578 | 1.128998262 |
| CALM3 | -0.550394266 | 11.51718724 | -4.837130288 | 0.000102971 | 0.000661578 | 1.128967008 |
| PROSER1 | 0.857561078 | 8.447958102 | 4.836934217 | 0.000103017 | 0.000661635 | 1.128521443 |
| HNRNPA2B1 | 1.372289448 | 10.61382032 | 4.836666596 | 0.00010308 | 0.0006618 | 1.12791328 |
| CORO7 | -0.888402379 | 13.74917545 | -4.836260272 | 0.000103177 | 0.000662176 | 1.126989909 |
| PPM1K | 1.52395565 | 8.454781737 | 4.835823988 | 0.00010328 | 0.000662489 | 1.125998443 |
| P2RX3 | -2.387411535 | 6.919618252 | -4.835736742 | 0.000103301 | 0.000662489 | 1.125800173 |
| RCBTB2 | 1.010652849 | 10.38313338 | 4.835549551 | 0.000103345 | 0.000662532 | 1.125374774 |
| 2-Sep | 0.653977393 | 9.421760109 | 4.835120456 | 0.000103447 | 0.000662944 | 1.124399632 |
| CNOT7 | 0.690916569 | 9.181729436 | 4.833791292 | 0.000103763 | 0.000664729 | 1.121378968 |
| PRIM2 | 0.882182739 | 5.959882476 | 4.832923962 | 0.00010397 | 0.000665812 | 1.119407823 |
| FAM126A | 1.203427886 | 4.815509033 | 4.831210704 | 0.00010438 | 0.000668194 | 1.11551406 |
| GPR152 | -1.223657023 | 9.67178466 | -4.827814574 | 0.000105198 | 0.000673183 | 1.107795148 |
| CELA2B | -2.572897558 | 6.259026923 | -4.827576829 | 0.000105256 | 0.000673306 | 1.107254765 |
| ZCCHC4 | 1.490462814 | 5.775230843 | 4.826348668 | 0.000105553 | 0.000674963 | 1.104463174 |
| XK | -2.203455981 | 9.090059933 | -4.825917495 | 0.000105658 | 0.000675387 | 1.103483106 |
| DCTN1 | -0.869429603 | 9.560300596 | -4.825290013 | 0.00010581 | 0.000676115 | 1.102056808 |
| E2F7 | 1.295770068 | 3.596802546 | 4.825072625 | 0.000105863 | 0.000676207 | 1.101562669 |
| C10orf76 | -1.310152392 | 10.58993384 | -4.822998113 | 0.000106369 | 0.000679192 | 1.096847028 |
| GRAMD1C | 1.505803026 | 8.749693911 | 4.82273356 | 0.000106433 | 0.000679358 | 1.09624565 |
| KIRREL | -1.384505616 | 4.61748696 | -4.821610077 | 0.000106709 | 0.000680867 | 1.093691718 |
| TUSC2 | 0.633215643 | 12.18339834 | 4.820759248 | 0.000106917 | 0.000681952 | 1.09175755 |
| IL10RA | 0.67568514 | 14.32432358 | 4.820389112 | 0.000107008 | 0.000682046 | 1.090916118 |
| ARL8B | 0.724706395 | 10.05169709 | 4.820383863 | 0.00010701 | 0.000682046 | 1.090904184 |
| WWP1 | 0.914750864 | 8.979845413 | 4.819504334 | 0.000107226 | 0.000683178 | 1.08890472 |
| ATRNL1 | -2.445650767 | 6.00638025 | -4.81926301 | 0.000107286 | 0.00068331 | 1.088356101 |
| NDOR1 | -0.768045395 | 10.52283212 | -4.817547288 | 0.00010771 | 0.000685761 | 1.084455554 |
| SCAF1 | -0.959395618 | 7.252664685 | -4.814672877 | 0.000108423 | 0.000690057 | 1.0779205 |
| CAMK1 | -1.117541477 | 7.531651664 | -4.814414047 | 0.000108488 | 0.000690218 | 1.077332023 |
| ZNF226 | 1.15903363 | 9.089455981 | 4.811838804 | 0.000109132 | 0.000694064 | 1.071476757 |
| FBXL8 | -1.079638241 | 8.479885773 | -4.81132645 | 0.000109261 | 0.000694631 | 1.07031179 |
| PDZD8 | 1.698317388 | 8.093626989 | 4.811121932 | 0.000109312 | 0.000694707 | 1.069846764 |
| CSF1R | -0.751834655 | 14.2949111 | -4.810059748 | 0.000109579 | 0.000696153 | 1.06743157 |
| ZC3H7A | 0.799903904 | 10.65327158 | 4.808906871 | 0.00010987 | 0.000697376 | 1.064810094 |
| TMOD3 | 1.669468532 | 9.186650141 | 4.808730038 | 0.000109915 | 0.000697376 | 1.064407997 |
| GLUD1 | 1.225749334 | 10.65285643 | 4.808717545 | 0.000109918 | 0.000697376 | 1.064379588 |
| CLDN19 | -1.353825695 | 8.871778402 | -4.80866846 | 0.00010993 | 0.000697376 | 1.064267973 |
| CNTNAP5 | -1.672131152 | 4.359179924 | -4.808104474 | 0.000110073 | 0.000698029 | 1.062985522 |
| MS4A5 | -0.703292898 | 4.867562523 | -4.807255527 | 0.000110288 | 0.00069914 | 1.061055064 |
| PLCD4 | -1.154410368 | 3.927879318 | -4.805769885 | 0.000110665 | 0.000701165 | 1.057676715 |
| VSIG10 | -1.066606213 | 5.326254854 | -4.805684105 | 0.000110687 | 0.000701165 | 1.057481648 |
| NLRP3 | -0.950019363 | 10.38328649 | -4.804709862 | 0.000110935 | 0.000702334 | 1.055266156 |
| ZNF213 | -1.154399024 | 7.398259281 | -4.804646544 | 0.000110952 | 0.000702334 | 1.055122165 |
| CRYBA4 | -2.511346579 | 5.224641961 | -4.803944424 | 0.000111131 | 0.000702998 | 1.053525469 |
| GPN3 | 0.901561727 | 8.684547106 | 4.803922498 | 0.000111136 | 0.000702998 | 1.053475607 |
| BANK1 | 1.165748838 | 9.55996712 | 4.803291905 | 0.000111298 | 0.000703765 | 1.052041549 |
| SLAIN2 | 1.472483023 | 5.501060061 | 4.80179266 | 0.000111682 | 0.000705941 | 1.048631977 |
| TMEM126A | 1.186774257 | 9.426601668 | 4.801585456 | 0.000111735 | 0.000706024 | 1.048160746 |
| N4BP2 | 1.521895324 | 8.345653348 | 4.800697447 | 0.000111964 | 0.000707213 | 1.046141183 |
| OR2T8 | -2.278459736 | 4.149926438 | -4.800386987 | 0.000112044 | 0.000707463 | 1.045435108 |
| GALNT3 | 1.47952942 | 6.033729871 | 4.800193484 | 0.000112093 | 0.000707524 | 1.044995023 |
| FBXL6 | -0.50835897 | 11.02617721 | -4.799790922 | 0.000112197 | 0.000707925 | 1.04407947 |
| UBE2E2 | 1.332406551 | 5.560122742 | 4.799555192 | 0.000112258 | 0.000708055 | 1.043543342 |
| SOCS1 | -1.22492529 | 9.065326043 | -4.79613025 | 0.000113146 | 0.000713398 | 1.035753589 |
| FAM81A | 1.500996441 | 2.706587563 | 4.793856907 | 0.000113739 | 0.000716881 | 1.030582745 |
| ZNF358 | -0.972490371 | 6.684819619 | -4.793173536 | 0.000113918 | 0.000717748 | 1.029028333 |
| AKAP5 | 1.317165564 | 2.879335691 | 4.793019814 | 0.000113958 | 0.000717748 | 1.028678669 |
| ZFAND3 | -0.826318785 | 8.036428483 | -4.790357521 | 0.000114658 | 0.000721898 | 1.022622719 |
| SCN9A | 1.260269083 | 3.521309383 | 4.789915791 | 0.000114775 | 0.000722373 | 1.021617877 |
| MEPCE | -0.800207874 | 8.322286125 | -4.789603056 | 0.000114857 | 0.000722395 | 1.020906467 |
| HSPB6 | -1.884859401 | 6.432800942 | -4.789591697 | 0.00011486 | 0.000722395 | 1.020880628 |
| ZIC1 | -1.188965768 | 5.74060802 | -4.788394553 | 0.000115177 | 0.000723964 | 1.018157318 |
| ATP8B3 | -1.563396824 | 3.81760541 | -4.788337683 | 0.000115192 | 0.000723964 | 1.018027945 |
| ZNF831 | 1.100297802 | 10.51352988 | 4.787575083 | 0.000115394 | 0.000724976 | 1.016293116 |
| B3GNT8 | -0.991346108 | 12.05339982 | -4.787345981 | 0.000115455 | 0.000724985 | 1.015771929 |
| SSR3 | 1.655741905 | 6.172265692 | 4.787259037 | 0.000115478 | 0.000724985 | 1.015574139 |
| SORBS3 | -2.186657043 | 14.44202273 | -4.786812531 | 0.000115597 | 0.000725471 | 1.014558369 |
| C11orf71 | -0.844886843 | 10.14131265 | -4.785307197 | 0.000115998 | 0.000727707 | 1.011133766 |
| EID2B | 1.436497208 | 5.636303129 | 4.785164571 | 0.000116036 | 0.000727707 | 1.010809291 |
| TMEM87B | 1.14614194 | 8.287931355 | 4.78416307 | 0.000116303 | 0.000729125 | 1.008530833 |
| C8orf59 | 1.935019152 | 8.969273519 | 4.783836196 | 0.000116391 | 0.000729414 | 1.007787171 |
| NUP107 | 0.880072509 | 9.844441807 | 4.782302516 | 0.000116802 | 0.000731731 | 1.00429787 |
| E2F5 | 1.66693048 | 6.51031507 | 4.780778376 | 0.000117213 | 0.00073404 | 1.000830166 |
| SNX2 | 1.044504459 | 12.32137708 | 4.78045237 | 0.000117301 | 0.00073433 | 1.000088427 |
| HSD17B14 | -2.221247392 | 7.742426001 | -4.779995958 | 0.000117424 | 0.00073484 | 0.999049974 |
| GTF2IRD2B | 1.113246525 | 5.212103273 | 4.779802911 | 0.000117476 | 0.000734904 | 0.998610742 |
| KCNK7 | -1.476661852 | 7.436991134 | -4.778179627 | 0.000117916 | 0.000737392 | 0.994917275 |
| UGT2B11 | -2.342744957 | 8.07746282 | -4.777161452 | 0.000118192 | 0.000738859 | 0.992600553 |
| RILPL1 | -1.033479151 | 7.795416804 | -4.776087628 | 0.000118485 | 0.000739696 | 0.990157158 |
| ARIH1 | 1.3703453 | 8.478368797 | 4.776070198 | 0.000118489 | 0.000739696 | 0.990117497 |
| OPA1 | 1.142389013 | 7.93356346 | 4.775897267 | 0.000118537 | 0.000739696 | 0.989724002 |
| CBX3 | 1.626845985 | 10.85483796 | 4.775806554 | 0.000118561 | 0.000739696 | 0.989517588 |
| ARX | -1.383230551 | 6.127167895 | -4.775764316 | 0.000118573 | 0.000739696 | 0.989421479 |
| LRRC69 | -0.959348224 | 4.840826759 | -4.775590451 | 0.00011862 | 0.000739696 | 0.989025856 |
| COTL1 | -0.951250926 | 15.88332254 | -4.775589546 | 0.000118621 | 0.000739696 | 0.989023797 |
| CNBP | 0.853166121 | 11.96469001 | 4.775176286 | 0.000118733 | 0.000740077 | 0.988083434 |
| ITPR1 | 0.912379018 | 9.919960983 | 4.775057269 | 0.000118766 | 0.000740077 | 0.987812614 |
| RBMXL1 | 0.58203072 | 9.504606078 | 4.774787749 | 0.00011884 | 0.000740274 | 0.987199323 |
| SLCO2A1 | -1.256658639 | 3.12642676 | -4.774172029 | 0.000119008 | 0.000741061 | 0.985798242 |
| C6orf62 | 0.686129565 | 12.702301 | 4.772697473 | 0.000119413 | 0.000743146 | 0.982442802 |
| CORT | -3.78616521 | 9.063324001 | -4.772643891 | 0.000119427 | 0.000743146 | 0.982320871 |
| ATP13A2 | -0.770827528 | 8.276763379 | -4.770182022 | 0.000120106 | 0.000747104 | 0.97671851 |
| BBS7 | 1.858970962 | 4.087520578 | 4.768609589 | 0.000120541 | 0.000749547 | 0.973140055 |
| ZNF10 | 1.2869536 | 6.746130599 | 4.766987113 | 0.000120992 | 0.000752086 | 0.969447596 |
| GPR35 | -1.006801059 | 7.678499846 | -4.765703609 | 0.00012135 | 0.000754045 | 0.966526491 |
| TINAGL1 | 2.551956161 | 13.13043685 | 4.763793478 | 0.000121885 | 0.000756847 | 0.962179121 |
| EVI5 | 0.967512943 | 5.64958472 | 4.763753525 | 0.000121896 | 0.000756847 | 0.962088188 |
| CDRT15L2 | -1.371913446 | 6.667161791 | -4.763631925 | 0.00012193 | 0.000756847 | 0.961811427 |
| KNDC1 | -1.639961921 | 3.78250454 | -4.762020322 | 0.000122384 | 0.000759392 | 0.958143352 |
| WDR20 | 0.986700893 | 6.607970691 | 4.76177637 | 0.000122452 | 0.00075955 | 0.957588098 |
| SLC10A2 | 1.407632786 | 3.225577323 | 4.759537744 | 0.000123085 | 0.000763206 | 0.952492679 |
| ENDOG | -1.074174021 | 9.724502529 | -4.759365613 | 0.000123134 | 0.000763239 | 0.952100876 |
| SLC25A14 | 0.687872686 | 9.081945496 | 4.75873118 | 0.000123314 | 0.000764085 | 0.950656774 |
| FAM71F2 | -1.351925903 | 6.016574713 | -4.758406915 | 0.000123406 | 0.000764387 | 0.949918674 |
| NFXL1 | 1.403294764 | 7.279158554 | 4.757878127 | 0.000123556 | 0.000765048 | 0.94871502 |
| IL17REL | -3.349978546 | 8.641169344 | -4.757597164 | 0.000123636 | 0.000765261 | 0.948075474 |
| NUDCD2 | 1.048267092 | 9.577494479 | 4.757451335 | 0.000123678 | 0.000765261 | 0.947743527 |
| PGAM5 | 1.057263886 | 4.532943207 | 4.757214291 | 0.000123745 | 0.00076541 | 0.947203947 |
| FAM117B | 0.996984686 | 9.619135571 | 4.756615169 | 0.000123916 | 0.000766197 | 0.945840165 |
| CACNG2 | -1.800889484 | 4.40801179 | -4.754472208 | 0.000124529 | 0.000769716 | 0.940962008 |
| BTRC | 0.607595899 | 8.229122891 | 4.753696685 | 0.000124752 | 0.000770821 | 0.939196587 |
| C15orf52 | -0.947090494 | 9.145994268 | -4.753308963 | 0.000124863 | 0.000771238 | 0.938313956 |
| THBS3 | -0.959157214 | 8.47206304 | -4.75270687 | 0.000125036 | 0.000771975 | 0.936943307 |
| HIST2H2AC | 2.002983309 | 11.13508278 | 4.752589294 | 0.00012507 | 0.000771975 | 0.936675646 |
| NKX2-3 | -1.630797869 | 7.709920219 | -4.751347282 | 0.000125428 | 0.000773915 | 0.93384818 |
| UCN2 | 1.5765234 | 3.09778126 | 4.751038263 | 0.000125518 | 0.000774194 | 0.933144683 |
| MRPL50 | 1.748116383 | 8.951722851 | 4.750268893 | 0.00012574 | 0.000775295 | 0.931393152 |
| ZNF23 | 1.345515229 | 6.373628182 | 4.748686375 | 0.000126199 | 0.000777626 | 0.927790348 |
| CUL3 | 0.812300045 | 10.82745721 | 4.748660658 | 0.000126207 | 0.000777626 | 0.9277318 |
| CPB1 | -3.591622496 | 4.334210969 | -4.748391235 | 0.000126285 | 0.000777659 | 0.927118413 |
| MAPKAPK5 | 0.756184288 | 8.460702181 | 4.7469912 | 0.000126693 | 0.000779802 | 0.923930945 |
| MITD1 | 1.011139909 | 10.68283224 | 4.746823276 | 0.000126742 | 0.000779831 | 0.923548627 |
| FAM183A | -1.299125957 | 3.821096347 | -4.746248555 | 0.00012691 | 0.00078059 | 0.922240128 |
| KAT8 | -0.651625061 | 13.08097496 | -4.745250626 | 0.000127202 | 0.000781918 | 0.919968055 |
| CCDC43 | 1.12381192 | 4.550032318 | 4.745080588 | 0.000127252 | 0.000781918 | 0.919580909 |
| DNAJA4 | -1.118209388 | 10.83274741 | -4.745055523 | 0.000127259 | 0.000781918 | 0.919523841 |
| UBTD1 | -1.202646212 | 10.61647548 | -4.744588021 | 0.000127396 | 0.000782487 | 0.91845942 |
| ZNF215 | 1.467679387 | 3.403475594 | 4.743950946 | 0.000127583 | 0.000783362 | 0.917008892 |
| TBKBP1 | -1.745224177 | 10.16826313 | -4.74347037 | 0.000127724 | 0.000783956 | 0.91591468 |
| APOE | -1.474417886 | 8.119537466 | -4.743170098 | 0.000127813 | 0.000784225 | 0.915230994 |
| ARMC1 | 1.198805558 | 9.101274267 | 4.742629778 | 0.000127972 | 0.000784927 | 0.914000734 |
| ERH | 1.162732313 | 10.29710611 | 4.741120263 | 0.000128418 | 0.000787387 | 0.910563635 |
| LMO7 | 0.979779189 | 4.149515648 | 4.739845923 | 0.000128795 | 0.000789426 | 0.907661946 |
| PIK3R1 | 1.434804654 | 9.631748523 | 4.739210048 | 0.000128984 | 0.000790308 | 0.906214024 |
| TOP2B | 0.895031607 | 11.8112009 | 4.738885409 | 0.00012908 | 0.000790366 | 0.905474796 |
| CYP2U1 | 0.89026899 | 7.06656062 | 4.738818416 | 0.0001291 | 0.000790366 | 0.905322248 |
| NDUFS1 | 0.722671995 | 11.09948927 | 4.738724309 | 0.000129128 | 0.000790366 | 0.905107958 |
| POLR2H | 0.801586666 | 10.29400163 | 4.737960062 | 0.000129356 | 0.000791484 | 0.903367687 |
| DUSP12 | 0.741271081 | 10.47271467 | 4.73599825 | 0.000129942 | 0.000794792 | 0.898900322 |
| PCGF5 | -1.24317911 | 11.02821016 | -4.734622435 | 0.000130354 | 0.000797038 | 0.89576727 |
| ZFAND5 | 0.823354392 | 11.46966349 | 4.734131853 | 0.000130502 | 0.000797617 | 0.894650081 |
| PRRC1 | 0.815290853 | 7.721173133 | 4.733778196 | 0.000130608 | 0.000797617 | 0.893844702 |
| TADA3 | -0.529618123 | 14.01260142 | -4.733688628 | 0.000130635 | 0.000797617 | 0.893640728 |
| MRPL32 | 1.278517802 | 10.7604183 | 4.733614024 | 0.000130658 | 0.000797617 | 0.893470832 |
| ARID3B | -0.660426517 | 11.91950167 | -4.733553422 | 0.000130676 | 0.000797617 | 0.893332823 |
| RNF187 | -1.273748614 | 10.4393524 | -4.73333641 | 0.000130741 | 0.000797739 | 0.892838621 |
| CCDC88A | 1.67839845 | 8.323060425 | 4.731939292 | 0.000131163 | 0.000800033 | 0.889656899 |
| CDHR2 | -1.981919969 | 6.267733552 | -4.730835216 | 0.000131497 | 0.000801793 | 0.887142482 |
| KCNJ16 | 1.949498783 | 3.536293539 | 4.729450383 | 0.000131917 | 0.000804058 | 0.883988594 |
| MOB1A | 1.845817604 | 9.642231514 | 4.729286201 | 0.000131967 | 0.000804058 | 0.883614672 |
| 2-Mar | -1.841284067 | 9.461687446 | -4.729159515 | 0.000132005 | 0.000804058 | 0.883326148 |
| LCLAT1 | 0.984235514 | 7.022929457 | 4.726252011 | 0.000132893 | 0.000809018 | 0.876704164 |
| GPX6 | -1.047091623 | 3.34254408 | -4.726190503 | 0.000132912 | 0.000809018 | 0.87656407 |
| PAIP2 | 0.95101449 | 11.78217258 | 4.725979024 | 0.000132976 | 0.000809132 | 0.876082404 |
| PANX1 | 0.996474079 | 7.896821766 | 4.725316461 | 0.00013318 | 0.000810088 | 0.874573328 |
| BTBD8 | 1.312003874 | 3.360129166 | 4.723963608 | 0.000133595 | 0.000812336 | 0.871491965 |
| TMEM128 | 1.099464729 | 8.036469095 | 4.722704835 | 0.000133984 | 0.000814179 | 0.868624822 |
| ART1 | -1.903439255 | 3.912989238 | -4.722680279 | 0.000133991 | 0.000814179 | 0.86856889 |
| KANK2 | -2.423323736 | 9.006386774 | -4.720985441 | 0.000134516 | 0.000817083 | 0.864708403 |
| CLN6 | -0.947122856 | 8.459459045 | -4.720373224 | 0.000134706 | 0.000817717 | 0.863313873 |
| FSHR | -0.985852998 | 4.672469863 | -4.720349281 | 0.000134713 | 0.000817717 | 0.863259334 |
| 5-Sep | -1.602763353 | 5.005796336 | -4.720074273 | 0.000134798 | 0.000817953 | 0.862632906 |
| RCAN1 | 1.300295047 | 7.111489532 | 4.71968995 | 0.000134918 | 0.000818396 | 0.861757468 |
| TCHP | 0.779971381 | 7.758038605 | 4.719075162 | 0.000135109 | 0.000819179 | 0.860357048 |
| ZCCHC8 | 0.787052609 | 10.52763904 | 4.718975847 | 0.00013514 | 0.000819179 | 0.860130817 |
| KRTAP15-1 | -2.249109598 | 3.522427029 | -4.718749408 | 0.000135211 | 0.000819324 | 0.859615011 |
| YIPF4 | 1.508243336 | 7.214110155 | 4.717748455 | 0.000135523 | 0.000820934 | 0.857334904 |
| SAMD11 | -2.446164512 | 10.30916141 | -4.716178457 | 0.000136014 | 0.000823438 | 0.853758468 |
| TTI1 | -0.989696821 | 10.70050686 | -4.716128378 | 0.00013603 | 0.000823438 | 0.853644386 |
| HS3ST6 | -1.960700173 | 3.447958086 | -4.715802459 | 0.000136132 | 0.000823773 | 0.852901934 |
| ATP6V1A | 0.711992121 | 11.95461403 | 4.71495845 | 0.000136397 | 0.000824853 | 0.850979238 |
| POP5 | 0.722740371 | 11.10135333 | 4.714935464 | 0.000136404 | 0.000824853 | 0.850926873 |
| LRRC72 | 2.003248508 | 3.985481366 | 4.714578229 | 0.000136517 | 0.000825247 | 0.850113065 |
| ALPP | -1.10281366 | 8.511045841 | -4.714430238 | 0.000136563 | 0.000825247 | 0.849775929 |
| UNC45A | -0.843467363 | 8.849735563 | -4.713908134 | 0.000136728 | 0.000825957 | 0.848586524 |
| KIAA1586 | 1.744093554 | 3.476476856 | 4.713736513 | 0.000136782 | 0.000826 | 0.848195552 |
| SEMA3C | 1.222005293 | 3.030606397 | 4.712588792 | 0.000137144 | 0.000827904 | 0.845580883 |
| SLC43A2 | -1.299369343 | 9.930554162 | -4.711696191 | 0.000137427 | 0.000829324 | 0.843547377 |
| TAF9 | 1.189380734 | 8.947446185 | 4.711110724 | 0.000137612 | 0.000829734 | 0.842213561 |
| KRTAP9-6 | -1.048065004 | 9.202513978 | -4.711097798 | 0.000137617 | 0.000829734 | 0.842184111 |
| CLNS1A | 0.951112966 | 12.01957174 | 4.711035603 | 0.000137636 | 0.000829734 | 0.842042418 |
| PYCARD | -0.78339276 | 14.52053296 | -4.710635278 | 0.000137763 | 0.000830215 | 0.841130384 |
| RTF1 | 0.80506643 | 8.05143083 | 4.710355665 | 0.000137852 | 0.000830466 | 0.840493357 |
| ANKRD18A | 2.284635613 | 4.210514948 | 4.709280936 | 0.000138194 | 0.000832241 | 0.83804483 |
| MAU2 | 1.227206843 | 10.66548584 | 4.708939877 | 0.000138303 | 0.000832611 | 0.837267796 |
| DCAF16 | 0.741069989 | 10.54196621 | 4.708330701 | 0.000138497 | 0.0008333 | 0.835879898 |
| RPL7 | 2.00283654 | 13.22473096 | 4.708266178 | 0.000138518 | 0.0008333 | 0.835732893 |
| PPIAL4A | 1.058890145 | 12.26065367 | 4.708135766 | 0.00013856 | 0.0008333 | 0.835435771 |
| ZNF585B | 0.950851172 | 6.888314332 | 4.702710493 | 0.000140304 | 0.000843334 | 0.823074583 |
| ASB7 | 0.647025352 | 7.022239821 | 4.70264803 | 0.000140324 | 0.000843334 | 0.822932257 |
| MPP5 | 0.963119791 | 7.675460091 | 4.701001465 | 0.000140858 | 0.000846253 | 0.819180418 |
| METTL3 | 0.706108228 | 10.57992153 | 4.700796549 | 0.000140924 | 0.000846363 | 0.818713491 |
| CELF5 | -1.371195447 | 5.249390141 | -4.700266811 | 0.000141096 | 0.000847109 | 0.817506412 |
| ZNF808 | 0.786743937 | 8.713121292 | 4.69981856 | 0.000141242 | 0.000847695 | 0.816485004 |
| MUC12 | -0.951819593 | 3.76774731 | -4.698987346 | 0.000141513 | 0.00084897 | 0.814590934 |
| FADS2 | -1.716125949 | 9.174351869 | -4.698870917 | 0.000141551 | 0.00084897 | 0.814325625 |
| GNG2 | 1.438305633 | 8.790942211 | 4.698674332 | 0.000141616 | 0.000849066 | 0.813877667 |
| KLRF1 | 1.740832461 | 8.966502968 | 4.697976369 | 0.000141844 | 0.000850144 | 0.812287204 |
| LYPLA2 | -0.817188367 | 12.05948243 | -4.697411807 | 0.000142028 | 0.000850961 | 0.811000711 |
| RBM7 | 1.016095634 | 7.150001009 | 4.697260018 | 0.000142078 | 0.000850969 | 0.810654821 |
| CRY1 | 1.550978936 | 4.809003578 | 4.695377694 | 0.000142696 | 0.00085438 | 0.806365386 |
| FOXI2 | 0.755221344 | 13.03603911 | 4.695041609 | 0.000142807 | 0.000854752 | 0.805599501 |
| S100A11 | -0.907497399 | 14.20455916 | -4.694480573 | 0.000142992 | 0.000855567 | 0.80432098 |
| ATP5O | 1.302264431 | 13.73709067 | 4.693861291 | 0.000143196 | 0.000856499 | 0.802909712 |
| STARD10 | -1.284416242 | 8.652889882 | -4.693321534 | 0.000143375 | 0.000857274 | 0.801679658 |
| TTF2 | 1.135194452 | 7.373767732 | 4.692840865 | 0.000143534 | 0.000857933 | 0.800584251 |
| HAS1 | -1.224413837 | 5.218328034 | -4.69171196 | 0.000143908 | 0.000859878 | 0.798011534 |
| ARSD | -1.042148594 | 9.267285158 | -4.690264361 | 0.000144389 | 0.000862461 | 0.794712454 |
| ANKRD54 | -0.95480462 | 6.641649852 | -4.689975627 | 0.000144485 | 0.000862528 | 0.79405442 |
| ARRDC5 | -0.915002263 | 7.511492475 | -4.689936292 | 0.000144499 | 0.000862528 | 0.793964774 |
| LRG1 | -1.367693237 | 11.9964038 | -4.689770188 | 0.000144554 | 0.000862565 | 0.793586217 |
| EMP2 | 1.971618198 | 5.527104988 | 4.688603439 | 0.000144943 | 0.000864596 | 0.790927117 |
| QRSL1 | 1.000319843 | 4.116616164 | 4.688293466 | 0.000145047 | 0.00086485 | 0.790220659 |
| PAX7 | -1.585687649 | 4.779741649 | -4.688182434 | 0.000145084 | 0.00086485 | 0.789967604 |
| BIVM | 0.996240349 | 6.172099453 | 4.686829726 | 0.000145538 | 0.000867259 | 0.786884607 |
| RPS18 | 0.931574139 | 16.47489017 | 4.686465192 | 0.00014566 | 0.000867694 | 0.786053774 |
| BET1L | -0.724697098 | 8.338391647 | -4.68567786 | 0.000145925 | 0.000868977 | 0.784259301 |
| LATS1 | 1.896372708 | 5.495236204 | 4.685275459 | 0.00014606 | 0.00086949 | 0.783342145 |
| BCL6 | 1.008110508 | 13.06428601 | 4.682843511 | 0.000146882 | 0.000874086 | 0.777799106 |
| ERP29 | 0.636502736 | 11.78933604 | 4.682513659 | 0.000146994 | 0.000874455 | 0.777047272 |
| THADA | -1.195622298 | 8.686401731 | -4.679991826 | 0.000147852 | 0.000879261 | 0.771299102 |
| HDGF | -1.089134806 | 10.93267239 | -4.679369603 | 0.000148064 | 0.000880226 | 0.769880793 |
| C14orf93 | -0.702494722 | 10.69329876 | -4.678533857 | 0.00014835 | 0.000881627 | 0.767975755 |
| LENG8 | -2.387304427 | 8.641172439 | -4.678290026 | 0.000148433 | 0.000881825 | 0.767419953 |
| 7-Mar | 1.084161502 | 8.767935076 | 4.677796091 | 0.000148603 | 0.000882533 | 0.766294037 |
| ZBTB47 | -0.994500821 | 9.747056327 | -4.676214158 | 0.000149146 | 0.000885461 | 0.762687993 |
| ALG8 | 0.598255642 | 11.18837439 | 4.676043568 | 0.000149205 | 0.00088551 | 0.762299125 |
| SLC25A34 | -0.977176989 | 7.505128058 | -4.675866604 | 0.000149266 | 0.000885512 | 0.761895725 |
| TSNARE1 | -1.966141601 | 6.966702346 | -4.675750338 | 0.000149306 | 0.000885512 | 0.761630692 |
| KIAA0513 | -0.784936317 | 13.02203562 | -4.675539009 | 0.000149379 | 0.000885512 | 0.761148952 |
| NR5A1 | -2.23826253 | 4.755663453 | -4.675311912 | 0.000149457 | 0.000885512 | 0.760631268 |
| NIPAL1 | -2.153228862 | 6.274637335 | -4.674394094 | 0.000149774 | 0.000886521 | 0.758539014 |
| ZNF26 | 1.410376373 | 7.550384765 | 4.674089859 | 0.000149879 | 0.000886521 | 0.757845476 |
| ARHGEF11 | -1.330084807 | 7.809927437 | -4.674070551 | 0.000149886 | 0.000886521 | 0.757801459 |
| DDX52 | 1.287464066 | 4.292333812 | 4.673993727 | 0.000149912 | 0.000886521 | 0.757626331 |
| CASQ1 | -1.588710511 | 6.413073982 | -4.673991112 | 0.000149913 | 0.000886521 | 0.757620369 |
| HNRNPA3 | 0.650760494 | 10.7745361 | 4.673898272 | 0.000149945 | 0.000886521 | 0.757408729 |
| TDRD3 | 1.471857973 | 4.803543997 | 4.673797665 | 0.00014998 | 0.000886521 | 0.757179383 |
| NFAT5 | 0.683515603 | 9.956407966 | 4.673517839 | 0.000150077 | 0.000886796 | 0.75654148 |
| ZNF600 | 1.528062986 | 8.902093833 | 4.673088564 | 0.000150226 | 0.000887376 | 0.755562882 |
| ATP6AP2 | 0.716335388 | 11.29030056 | 4.672650648 | 0.000150378 | 0.000887975 | 0.754564581 |
| TSPAN3 | 0.909774735 | 9.960804084 | 4.671909478 | 0.000150635 | 0.000889123 | 0.752874944 |
| MUC7 | -1.302738953 | 4.492620921 | -4.67180024 | 0.000150673 | 0.000889123 | 0.752625914 |
| JPH2 | -1.525021042 | 5.173543008 | -4.671388693 | 0.000150816 | 0.000889669 | 0.751687705 |
| SEC63 | 1.014763517 | 8.265911379 | 4.671133385 | 0.000150905 | 0.000889895 | 0.751105673 |
| C12orf66 | 0.822898062 | 4.21690385 | 4.670594071 | 0.000151093 | 0.000890677 | 0.749876179 |
| IFI27L1 | 0.903016887 | 10.47047838 | 4.67046192 | 0.000151139 | 0.000890677 | 0.749574909 |
| KLRC4 | 1.573291932 | 9.812262177 | 4.669706729 | 0.000151403 | 0.000891932 | 0.747853248 |
| ESCO1 | 1.960298449 | 5.626132109 | 4.669418654 | 0.000151503 | 0.000892226 | 0.747196501 |
| CLCA4 | -1.005025359 | 2.666334407 | -4.669017806 | 0.000151644 | 0.000892753 | 0.746282649 |
| ST3GAL2 | -0.975275333 | 10.29405894 | -4.668293951 | 0.000151897 | 0.000893947 | 0.744632391 |
| HSPG2 | -2.146771209 | 7.15653655 | -4.668109354 | 0.000151962 | 0.000894028 | 0.744211542 |
| SP7 | -1.389570786 | 6.579755064 | -4.666991558 | 0.000152354 | 0.000896038 | 0.741663128 |
| SHOX | 3.419309669 | 4.498756083 | 4.665777157 | 0.000152782 | 0.000898253 | 0.738894419 |
| TNFRSF10A | 0.924618728 | 6.984982925 | 4.665391919 | 0.000152918 | 0.000898751 | 0.738016105 |
| DTWD1 | 1.065312113 | 6.996500003 | 4.66500236 | 0.000153056 | 0.000899259 | 0.737127934 |
| SLC22A5 | 1.10732476 | 6.469097259 | 4.664423935 | 0.00015326 | 0.00090016 | 0.735809152 |
| DNAJC16 | 0.678767273 | 6.376000271 | 4.664275557 | 0.000153313 | 0.000900167 | 0.735470854 |
| MADCAM1 | -1.742421649 | 6.089188937 | -4.664016253 | 0.000153404 | 0.000900406 | 0.734879649 |
| HIST1H4A | 1.912751614 | 7.113031032 | 4.663599278 | 0.000153552 | 0.000900922 | 0.73392895 |
| ITGB3BP | 1.74023351 | 7.155229406 | 4.663478713 | 0.000153595 | 0.000900922 | 0.733654062 |
| NUF2 | 1.97695222 | 4.995140237 | 4.662016723 | 0.000154114 | 0.000903666 | 0.73032069 |
| SNRNP27 | 1.133977908 | 8.755641015 | 4.661336591 | 0.000154356 | 0.000904784 | 0.728769948 |
| OR7E24 | -1.96023263 | 8.153981738 | -4.661009749 | 0.000154473 | 0.000905165 | 0.728024723 |
| HINT1 | 1.327488762 | 14.09780146 | 4.660846757 | 0.000154531 | 0.000905204 | 0.727653086 |
| RERG | -2.201482766 | 6.593250464 | -4.658716771 | 0.000155292 | 0.000909282 | 0.722796447 |
| ARGFX | -2.006151611 | 6.319481283 | -4.658611093 | 0.00015533 | 0.000909282 | 0.722555485 |
| PDGFC | 1.170437109 | 5.819862649 | 4.658238342 | 0.000155464 | 0.000909762 | 0.721705546 |
| CLIP1 | 0.686604747 | 9.724680128 | 4.656469877 | 0.0001561 | 0.00091318 | 0.717673073 |
| RIPK3 | -0.650399446 | 8.395514305 | -4.656228643 | 0.000156187 | 0.000913385 | 0.717123001 |
| HNF1B | -1.249124924 | 5.828037248 | -4.655902543 | 0.000156305 | 0.000913769 | 0.716379409 |
| NSUN6 | 1.237996987 | 5.845898668 | 4.654881884 | 0.000156673 | 0.00091562 | 0.714052024 |
| CDKAL1 | 1.034304811 | 6.511011911 | 4.654258785 | 0.000156899 | 0.000916634 | 0.712631169 |
| SLC37A4 | -0.792581765 | 9.371396609 | -4.653394599 | 0.000157212 | 0.000918159 | 0.710660537 |
| TM9SF1 | -0.771045643 | 8.768111658 | -4.653060568 | 0.000157334 | 0.000918395 | 0.709898829 |
| HELB | 1.277496373 | 6.829206799 | 4.652856449 | 0.000157408 | 0.000918395 | 0.709433365 |
| MPZL1 | -0.816213375 | 10.37395543 | -4.652736466 | 0.000157451 | 0.000918395 | 0.70915976 |
| NAALADL2 | -2.237647902 | 6.395641541 | -4.652708348 | 0.000157462 | 0.000918395 | 0.70909564 |
| C10orf62 | -1.523876192 | 3.949921938 | -4.651221486 | 0.000158003 | 0.000921237 | 0.70570501 |
| DNAJC19 | 1.001059103 | 7.435871881 | 4.651083069 | 0.000158054 | 0.000921237 | 0.70538936 |
| CPA2 | -1.47225951 | 3.738948988 | -4.650463558 | 0.00015828 | 0.00092225 | 0.703976608 |
| CDH9 | -1.764260922 | 4.626785756 | -4.649158411 | 0.000158758 | 0.000924427 | 0.701000263 |
| PPP1R2 | 1.037000762 | 8.513169725 | 4.649152137 | 0.00015876 | 0.000924427 | 0.700985954 |
| HSD17B12 | 0.894863969 | 7.979061681 | 4.649012042 | 0.000158811 | 0.000924427 | 0.700666469 |
| RASGEF1C | -1.732159791 | 5.46585121 | -4.648154283 | 0.000159126 | 0.000925953 | 0.698710343 |
| TDRD1 | -2.517832853 | 6.374803646 | -4.647481782 | 0.000159373 | 0.000927086 | 0.697176683 |
| CFHR3 | 1.600410396 | 5.086900421 | 4.646935237 | 0.000159575 | 0.00092795 | 0.695930257 |
| TAX1BP3 | -0.929109677 | 7.875542043 | -4.64645282 | 0.000159752 | 0.000928677 | 0.694830072 |
| SH3GL1 | -0.768572277 | 12.11496846 | -4.646036625 | 0.000159906 | 0.000929055 | 0.693880903 |
| ASF1A | 1.566154091 | 4.896891355 | 4.645990203 | 0.000159923 | 0.000929055 | 0.693775034 |
| DUSP4 | -1.813440371 | 7.970311456 | -4.645515942 | 0.000160098 | 0.000929766 | 0.692693433 |
| RHBG | -1.330917184 | 5.648328871 | -4.645020466 | 0.000160282 | 0.000930192 | 0.691563443 |
| HNRNPC | 0.605989292 | 11.89706628 | 4.644964416 | 0.000160302 | 0.000930192 | 0.691435613 |
| TMEM181 | 1.64628369 | 9.38098631 | 4.644888905 | 0.00016033 | 0.000930192 | 0.6912634 |
| TOPORS | 1.240940631 | 8.349169761 | 4.644254363 | 0.000160566 | 0.000931248 | 0.689816239 |
| P2RX7 | -0.87477866 | 8.771924188 | -4.643919509 | 0.00016069 | 0.000931662 | 0.689052552 |
| ACVR2A | 1.599207546 | 5.404515572 | 4.643088885 | 0.000160998 | 0.000933143 | 0.687158169 |
| CACNG6 | -1.603836218 | 8.433338499 | -4.642508298 | 0.000161214 | 0.000934087 | 0.685834023 |
| WDR73 | -0.520076728 | 9.98740061 | -4.641289754 | 0.000161669 | 0.000936404 | 0.683054855 |
| ABCD1 | -0.902224288 | 6.856259976 | -4.641150262 | 0.000161721 | 0.000936404 | 0.682736708 |
| SV2A | -1.900811027 | 6.59614661 | -4.640801969 | 0.000161851 | 0.000936849 | 0.681942337 |
| ZNF775 | -0.724426803 | 10.09583589 | -4.639245449 | 0.000162434 | 0.000939914 | 0.678392242 |
| ADAMTSL2 | -0.962939994 | 5.950383456 | -4.638738875 | 0.000162624 | 0.000940704 | 0.677236836 |
| DOCK3 | -1.744674626 | 7.015503722 | -4.638024275 | 0.000162893 | 0.000941948 | 0.675606947 |
| ZNF33A | 1.495092202 | 8.701152041 | 4.635490228 | 0.000163849 | 0.00094669 | 0.669827052 |
| CASP8AP2 | 1.036427235 | 7.230064993 | 4.635433665 | 0.00016387 | 0.00094669 | 0.669698034 |
| DHRS7B | -1.04216852 | 9.551295076 | -4.635423326 | 0.000163874 | 0.00094669 | 0.669674453 |
| BATF3 | -1.222325953 | 8.942902019 | -4.634849601 | 0.000164091 | 0.000947634 | 0.668365816 |
| PANX3 | -1.728515038 | 6.378785624 | -4.633403371 | 0.00016464 | 0.000950493 | 0.665066995 |
| PSMC6 | 1.524587512 | 9.344834066 | 4.632103052 | 0.000165136 | 0.00095304 | 0.662100936 |
| BCKDHB | 0.745550707 | 6.854673929 | 4.631804672 | 0.00016525 | 0.000953384 | 0.661420318 |
| GPHB5 | -1.31377205 | 7.348490219 | -4.631608861 | 0.000165324 | 0.000953502 | 0.660973662 |
| PROK2 | 1.220353671 | 12.37596518 | 4.630291563 | 0.000165828 | 0.000956095 | 0.657968798 |
| BTN1A1 | -2.083292153 | 5.434055755 | -4.629880662 | 0.000165986 | 0.0009566 | 0.657031489 |
| IL17C | -1.768362821 | 4.926570609 | -4.629779081 | 0.000166025 | 0.0009566 | 0.656799771 |
| STX10 | -0.798309976 | 11.95147361 | -4.629081499 | 0.000166292 | 0.000957829 | 0.655208498 |
| ZNF547 | 1.155597659 | 4.787854808 | 4.628397837 | 0.000166555 | 0.000959029 | 0.653648962 |
| WDR18 | -1.393993681 | 11.8834082 | -4.628186252 | 0.000166637 | 0.000959184 | 0.653166301 |
| OSCP1 | -0.869003821 | 5.14001359 | -4.627200124 | 0.000167017 | 0.000961057 | 0.650916764 |
| WSCD2 | -2.90980628 | 6.572258707 | -4.625588024 | 0.00016764 | 0.000964327 | 0.647239203 |
| RAD50 | 1.24303673 | 7.548015076 | 4.623203971 | 0.000168566 | 0.000969337 | 0.6418005 |
| CLRN3 | -1.522099224 | 2.983498057 | -4.622627595 | 0.000168791 | 0.000970311 | 0.640485596 |
| CALML5 | -1.329584838 | 6.148485731 | -4.621495996 | 0.000169232 | 0.000972533 | 0.637904013 |
| LIF | -1.328656535 | 5.334021181 | -4.621070352 | 0.000169399 | 0.000973172 | 0.636932959 |
| GSTM5 | -1.274849028 | 7.530154974 | -4.620681163 | 0.000169551 | 0.000973669 | 0.636045065 |
| FAM198B | 1.003519936 | 9.669403108 | 4.62050177 | 0.000169622 | 0.000973669 | 0.635635797 |
| HSPA1A | -0.81534034 | 13.51436928 | -4.62042574 | 0.000169651 | 0.000973669 | 0.635462342 |
| NLK | 0.769734205 | 9.084571725 | 4.620114287 | 0.000169774 | 0.000973793 | 0.63475179 |
| GIPC3 | -1.446028195 | 5.645172918 | -4.620088247 | 0.000169784 | 0.000973793 | 0.634692381 |
| NAA35 | 0.671956679 | 6.949100154 | 4.619481898 | 0.000170022 | 0.00097484 | 0.633309042 |
| LRRC39 | 1.450127684 | 3.819822734 | 4.618779877 | 0.000170298 | 0.000976104 | 0.631707417 |
| HTATSF1 | 0.969958642 | 10.31456393 | 4.618585481 | 0.000170374 | 0.000976225 | 0.631263911 |
| ZDHHC6 | 1.397367565 | 7.418497555 | 4.617677628 | 0.000170732 | 0.000977956 | 0.629192667 |
| LYPLA1 | 0.566408772 | 9.644427507 | 4.617321858 | 0.000170873 | 0.000978442 | 0.628380979 |
| ACE | -1.457811192 | 5.079099219 | -4.61703885 | 0.000170984 | 0.000978763 | 0.627735293 |
| ZNF485 | 1.03665732 | 6.068389864 | 4.614981996 | 0.000171799 | 0.000983106 | 0.6230425 |
| RPL36A | 2.0594896 | 14.97061316 | 4.613636754 | 0.000172334 | 0.000985847 | 0.619973206 |
| OR2M2 | -1.698158509 | 4.093756529 | -4.613096267 | 0.000172549 | 0.000986758 | 0.618740021 |
| SLC7A5 | -1.482863519 | 6.787848159 | -4.611699276 | 0.000173107 | 0.000989359 | 0.615552578 |
| PPM1N | -0.870737611 | 9.069822063 | -4.611675849 | 0.000173117 | 0.000989359 | 0.615499125 |
| ACVR2B | 1.392175625 | 5.469520363 | 4.610572666 | 0.000173559 | 0.000991562 | 0.61298201 |
| GOLIM4 | 1.578876152 | 3.851922887 | 4.609417038 | 0.000174023 | 0.000993891 | 0.610345191 |
| TP53RK | 0.771847887 | 6.871024057 | 4.608650454 | 0.000174331 | 0.000995331 | 0.608596039 |
| ZC3H15 | 1.438202777 | 10.65650046 | 4.608395118 | 0.000174434 | 0.000995595 | 0.608013423 |
| RPS29 | 1.133952401 | 16.15014997 | 4.607233551 | 0.000174903 | 0.000997948 | 0.605362979 |
| CLTC | 1.104425174 | 5.122433452 | 4.606376458 | 0.00017525 | 0.000999602 | 0.603407255 |
| ACTR5 | -2.014245508 | 8.691627976 | -4.60601362 | 0.000175397 | 0.001000117 | 0.602579321 |
| COMMD8 | 1.663028459 | 8.667670077 | 4.604638557 | 0.000175956 | 0.001002712 | 0.599441627 |
| ZIM2 | -1.929231806 | 5.546862033 | -4.604521815 | 0.000176003 | 0.001002712 | 0.599175235 |
| SAMD10 | -0.750952654 | 9.397322399 | -4.604394894 | 0.000176055 | 0.001002712 | 0.598885617 |
| BBIP1 | 1.186403632 | 7.529226881 | 4.604331499 | 0.000176081 | 0.001002712 | 0.598740956 |
| BCKDK | -1.014742559 | 10.76377853 | -4.603482384 | 0.000176426 | 0.001004357 | 0.596803364 |
| MYL4 | -1.684871506 | 6.821815683 | -4.603113014 | 0.000176577 | 0.00100489 | 0.595960494 |
| MAST1 | -1.156608898 | 6.615204042 | -4.602712224 | 0.000176741 | 0.001005496 | 0.595045922 |
| SPRY3 | -1.634900048 | 6.74075589 | -4.601863831 | 0.000177088 | 0.001007144 | 0.593109937 |
| HAO1 | -1.868800912 | 4.145132955 | -4.599206506 | 0.000178179 | 0.001013022 | 0.587045943 |
| ACER3 | 1.296894757 | 4.840209757 | 4.598649239 | 0.000178409 | 0.001014 | 0.585774239 |
| TSNAXIP1 | -1.428752672 | 4.290673682 | -4.598034506 | 0.000178662 | 0.001015114 | 0.584371385 |
| KIAA1522 | -1.171319505 | 7.453135367 | -4.597870772 | 0.00017873 | 0.001015171 | 0.583997732 |
| SNCA | -2.108478719 | 14.30407406 | -4.597666837 | 0.000178814 | 0.001015321 | 0.583532338 |
| COX7C | 1.648925589 | 13.44091338 | 4.597499685 | 0.000178883 | 0.001015386 | 0.583150885 |
| SPCS2 | 0.589452133 | 10.79498743 | 4.596687777 | 0.00017922 | 0.001016965 | 0.581298038 |
| MKKS | 0.770761737 | 9.736112265 | 4.596372573 | 0.00017935 | 0.001017378 | 0.58057871 |
| SCGB3A2 | -1.37713168 | 9.110922435 | -4.596014045 | 0.000179499 | 0.001017894 | 0.579760509 |
| SPATA20 | -1.215696714 | 9.104783053 | -4.593038041 | 0.000180738 | 0.001024591 | 0.572968788 |
| TMEM126B | 1.461234852 | 8.386112103 | 4.592790659 | 0.000180842 | 0.001024847 | 0.572404211 |
| NFASC | -1.342986754 | 5.65643955 | -4.591818372 | 0.000181249 | 0.001026823 | 0.570185237 |
| ZFP91 | 0.704778311 | 8.675454048 | 4.590240993 | 0.000181911 | 0.001030243 | 0.566585253 |
| MIEN1 | -0.58034607 | 13.14667875 | -4.59001693 | 0.000182005 | 0.001030445 | 0.566073877 |
| IL1RL2 | -1.851546987 | 5.394287996 | -4.588706295 | 0.000182558 | 0.00103324 | 0.563082611 |
| FAM76A | 0.845654422 | 6.41486371 | 4.588117619 | 0.000182806 | 0.001034314 | 0.56173906 |
| ZWILCH | 1.293375327 | 5.96405094 | 4.586649506 | 0.000183428 | 0.001037498 | 0.558388303 |
| BNIP3L | 1.310760488 | 13.01587139 | 4.585939149 | 0.00018373 | 0.001038869 | 0.556766994 |
| THUMPD1 | 1.533461911 | 7.166931117 | 4.585297066 | 0.000184003 | 0.001040079 | 0.555301501 |
| KLK5 | -2.040594488 | 5.20722638 | -4.584269238 | 0.00018444 | 0.001042219 | 0.55295556 |
| DUSP8 | -1.183162367 | 9.25787371 | -4.583493439 | 0.000184772 | 0.00104355 | 0.551184837 |
| ZNF793 | 1.115107304 | 6.433214352 | 4.583324335 | 0.000184844 | 0.00104355 | 0.550798863 |
| TRNT1 | 1.270821756 | 7.473908655 | 4.581993142 | 0.000185414 | 0.001046374 | 0.547760439 |
| RPS3A | 2.085866833 | 14.25723287 | 4.581696264 | 0.000185541 | 0.001046757 | 0.547082814 |
| RAD23B | 1.255707831 | 7.360583868 | 4.581453302 | 0.000185645 | 0.001047009 | 0.546528249 |
| TRIM46 | -0.91801723 | 7.365258301 | -4.580781604 | 0.000185934 | 0.001048301 | 0.544995083 |
| WFIKKN2 | -1.06666659 | 3.575982548 | -4.579893466 | 0.000186316 | 0.00105012 | 0.542967869 |
| GALNT12 | 1.035594432 | 5.709361782 | 4.579388659 | 0.000186534 | 0.00105101 | 0.541815616 |
| GPSM1 | -1.915830307 | 10.10009524 | -4.578569721 | 0.000186888 | 0.001052665 | 0.539946325 |
| ANKUB1 | -1.63287691 | 4.70676341 | -4.578232196 | 0.000187034 | 0.00105315 | 0.539175892 |
| SPG11 | 0.586927669 | 11.58659345 | 4.577569943 | 0.00018732 | 0.001054427 | 0.537664229 |
| SHOC2 | 1.300445625 | 9.642011503 | 4.575978058 | 0.000188011 | 0.001057977 | 0.534030536 |
| BIRC2 | 1.342581132 | 10.0628676 | 4.575290667 | 0.000188311 | 0.001059322 | 0.532461451 |
| KDM1B | 1.343775726 | 5.617912548 | 4.574928274 | 0.000188468 | 0.001059584 | 0.531634224 |
| UNKL | -1.009803539 | 8.751238979 | -4.574907178 | 0.000188478 | 0.001059584 | 0.531586068 |
| PTCD3 | 1.614202953 | 6.476157359 | 4.574199503 | 0.000188786 | 0.001060981 | 0.529970664 |
| DLC1 | -0.861343384 | 3.960751838 | -4.573968191 | 0.000188887 | 0.001061209 | 0.529442645 |
| ASPG | -1.615591293 | 5.292303706 | -4.573718759 | 0.000188996 | 0.001061483 | 0.528873265 |
| ZNF230 | 1.244545331 | 6.357834863 | 4.572722232 | 0.000189433 | 0.001063593 | 0.526598465 |
| STAG1 | 1.867859787 | 5.789207856 | 4.571276684 | 0.000190067 | 0.001066633 | 0.523298629 |
| GDE1 | -1.164873199 | 10.61722953 | -4.571212216 | 0.000190095 | 0.001066633 | 0.523151461 |
| PABPC4L | -1.917477378 | 4.542732116 | -4.570287101 | 0.000190503 | 0.001068277 | 0.521039617 |
| NPFF | -0.5933993 | 10.92651587 | -4.570270649 | 0.00019051 | 0.001068277 | 0.521002061 |
| HGFAC | -2.660937881 | 8.434629492 | -4.568544483 | 0.000191272 | 0.00107221 | 0.517061528 |
| GLT1D1 | -0.962440979 | 12.48086423 | -4.568224862 | 0.000191414 | 0.001072661 | 0.516331883 |
| SPHAR | 0.640474436 | 7.824851354 | 4.567955378 | 0.000191533 | 0.001072801 | 0.515716688 |
| CETP | -1.36732523 | 4.90355731 | -4.567893171 | 0.000191561 | 0.001072801 | 0.515574679 |
| MCTP2 | 1.370211576 | 8.26070902 | 4.56716411 | 0.000191884 | 0.001073976 | 0.513910326 |
| SNRPG | 1.795911639 | 10.84368778 | 4.567144648 | 0.000191893 | 0.001073976 | 0.513865898 |
| FIBCD1 | -2.198202514 | 7.224294195 | -4.566675938 | 0.000192101 | 0.001074799 | 0.512795886 |
| CYTH4 | -0.659940764 | 13.39897923 | -4.565966096 | 0.000192417 | 0.001076223 | 0.511175387 |
| MRE11A | 1.376979764 | 8.544728502 | 4.565185574 | 0.000192764 | 0.001077825 | 0.509393519 |
| PROCA1 | -0.928191379 | 8.753789898 | -4.562757546 | 0.00019385 | 0.001083553 | 0.503850432 |
| PGLYRP1 | -1.457068025 | 10.6993924 | -4.562446166 | 0.00019399 | 0.001083989 | 0.503139554 |
| IMPA2 | -0.987383873 | 11.85570191 | -4.561829723 | 0.000194267 | 0.001085191 | 0.501732212 |
| PKD2 | 0.945940354 | 7.209538553 | 4.559459806 | 0.000195335 | 0.001090812 | 0.496321599 |
| HEPACAM | -1.34288191 | 4.088609977 | -4.559238106 | 0.000195435 | 0.001091025 | 0.495815443 |
| METTL6 | 0.807581742 | 6.438312345 | 4.557313795 | 0.000196307 | 0.001095546 | 0.491422056 |
| ASPDH | -1.711857419 | 4.700478453 | -4.556537513 | 0.00019666 | 0.001096977 | 0.489649706 |
| CRNN | -0.939019613 | 5.625111894 | -4.556353973 | 0.000196744 | 0.001096977 | 0.489230657 |
| GSTT2B | -1.804942414 | 4.867765058 | -4.556338655 | 0.000196751 | 0.001096977 | 0.489195685 |
| CCDC65 | 2.340652407 | 5.749530946 | 4.555487059 | 0.000197139 | 0.001098792 | 0.487251362 |
| PTMS | -1.809318426 | 15.6241421 | -4.552832295 | 0.000198354 | 0.001105213 | 0.481190023 |
| C10orf131 | 1.039984447 | 2.460664406 | 4.552084461 | 0.000198697 | 0.001106777 | 0.479482544 |
| TNFRSF4 | -1.290622521 | 9.862977478 | -4.551866343 | 0.000198798 | 0.001106985 | 0.478984527 |
| TRAPPC10 | 0.812131594 | 8.358208495 | 4.550160224 | 0.000199584 | 0.001111012 | 0.475089004 |
| ARID1A | -0.718305487 | 8.508740492 | -4.5499444 | 0.000199684 | 0.001111216 | 0.474596215 |
| CMA1 | -3.015288832 | 5.95715783 | -4.549598929 | 0.000199844 | 0.001111753 | 0.473807404 |
| MON1A | -0.601906074 | 10.16163013 | -4.548044835 | 0.000200564 | 0.001115406 | 0.470258915 |
| IL17RE | -1.689424442 | 6.539746619 | -4.547602954 | 0.000200769 | 0.001116194 | 0.46924995 |
| KCNH8 | 1.890054915 | 5.674962466 | 4.546331346 | 0.000201361 | 0.001119041 | 0.466346415 |
| RHAG | -2.120828086 | 4.179228267 | -4.546229452 | 0.000201408 | 0.001119041 | 0.466113752 |
| ZDHHC21 | 0.996954788 | 7.421617697 | 4.545864995 | 0.000201578 | 0.001119631 | 0.46528156 |
| ZNF880 | 0.868891995 | 6.327510171 | 4.545275243 | 0.000201854 | 0.001120806 | 0.463934927 |
| EVI5L | -0.649317635 | 9.453469919 | -4.544671309 | 0.000202136 | 0.00112202 | 0.462555902 |
| SLC38A3 | -2.063150871 | 5.254644881 | -4.541392029 | 0.000203676 | 0.00113016 | 0.455067841 |
| FEM1B | 1.097471999 | 6.723772228 | 4.541275701 | 0.000203731 | 0.00113016 | 0.454802208 |
| LRP1 | -0.964061127 | 6.834112151 | -4.541060712 | 0.000203832 | 0.001130366 | 0.454311284 |
| KLKB1 | -2.838667193 | 7.887392535 | -4.539210227 | 0.000204707 | 0.00113486 | 0.450085682 |
| C10orf105 | -1.42288733 | 9.504642528 | -4.537049356 | 0.000205734 | 0.001140192 | 0.445151217 |
| TRIM13 | 0.676978308 | 9.96300145 | 4.536648062 | 0.000205925 | 0.001140892 | 0.444234831 |
| RAP2A | 1.917881728 | 5.411950802 | 4.535670329 | 0.000206392 | 0.001143117 | 0.442002082 |
| EBF2 | -1.175933141 | 3.463968161 | -4.534553298 | 0.000206926 | 0.001145716 | 0.43945121 |
| ADH5 | 1.340593899 | 7.09380094 | 4.534254203 | 0.000207069 | 0.001145838 | 0.438768186 |
| SLC30A4 | 1.122672176 | 5.954497172 | 4.534235357 | 0.000207078 | 0.001145838 | 0.43872515 |
| UBFD1 | -0.645320357 | 9.192728627 | -4.533705364 | 0.000207333 | 0.001146744 | 0.437514835 |
| ZNF222 | 1.451045058 | 4.70825184 | 4.533622275 | 0.000207373 | 0.001146744 | 0.437325089 |
| SLC22A18 | -0.971881774 | 11.68695572 | -4.533341212 | 0.000207508 | 0.00114713 | 0.436683239 |
| HSPB9 | -1.55867088 | 6.357302945 | -4.533077423 | 0.000207634 | 0.00114747 | 0.436080834 |
| TSPAN33 | -1.348772908 | 10.03663883 | -4.531307852 | 0.000208487 | 0.001151579 | 0.4320397 |
| ROPN1L | -0.957136882 | 10.24227531 | -4.531261954 | 0.000208509 | 0.001151579 | 0.431934884 |
| IGF2 | -2.161100055 | 6.447993861 | -4.530759537 | 0.000208752 | 0.001152558 | 0.430787512 |
| TMEM42 | 0.752795123 | 11.7403315 | 4.529304 | 0.000209456 | 0.001156085 | 0.427463465 |
| MINPP1 | 1.092023412 | 5.83659295 | 4.528870387 | 0.000209667 | 0.001156883 | 0.426473204 |
| FKTN | 1.437137242 | 3.751088501 | 4.525885739 | 0.000211121 | 0.001164541 | 0.419656927 |
| ABHD1 | -0.75655445 | 7.038456177 | -4.525453728 | 0.000211332 | 0.001164977 | 0.418670294 |
| C1QTNF3 | 1.043565644 | 5.180802273 | 4.525452963 | 0.000211332 | 0.001164977 | 0.418668547 |
| TSTA3 | -1.995463474 | 10.56327444 | -4.524785565 | 0.000211659 | 0.001166413 | 0.417144326 |
| ABL2 | 0.751393395 | 6.125472432 | 4.522491922 | 0.000212786 | 0.001171946 | 0.411905979 |
| RAB6A | 1.312295937 | 8.604976818 | 4.522471565 | 0.000212796 | 0.001171946 | 0.411859485 |
| PPP1R1C | -1.793786015 | 3.801657501 | -4.522050704 | 0.000213004 | 0.001172721 | 0.410898289 |
| PAK1 | -0.849568424 | 11.27159093 | -4.520934486 | 0.000213555 | 0.001175388 | 0.40834896 |
| SREBF1 | -1.318735525 | 11.63934907 | -4.52025234 | 0.000213893 | 0.001176878 | 0.406790997 |
| NECAB2 | -2.121638468 | 6.626991771 | -4.520063879 | 0.000213986 | 0.001177023 | 0.406360565 |
| MECOM | 1.06803529 | 2.987692168 | 4.519753705 | 0.00021414 | 0.001177501 | 0.405652151 |
| OBSCN | -1.523089745 | 7.313028321 | -4.519331449 | 0.000214349 | 0.001178284 | 0.404687745 |
| TRAPPC5 | -0.741841916 | 13.57252493 | -4.518707612 | 0.000214659 | 0.001179619 | 0.403262936 |
| NHS | 1.271457684 | 4.293380443 | 4.51785549 | 0.000215083 | 0.00118158 | 0.401316722 |
| BTBD1 | 1.902252917 | 9.579326398 | 4.517263165 | 0.000215378 | 0.001182833 | 0.399963867 |
| PLA2G2A | -2.743305693 | 4.658793784 | -4.51618102 | 0.000215919 | 0.00118543 | 0.397492258 |
| CRLF3 | 1.637875755 | 8.90537272 | 4.515447511 | 0.000216286 | 0.001187055 | 0.395816917 |
| TTC16 | -0.890601892 | 8.760592714 | -4.51532031 | 0.00021635 | 0.001187055 | 0.395526388 |
| CPXM2 | -1.12457637 | 3.374202341 | -4.514691704 | 0.000216665 | 0.001188413 | 0.394090638 |
| CNPPD1 | -1.245507521 | 9.59011508 | -4.514167439 | 0.000216928 | 0.001189486 | 0.392893198 |
| TMEM91 | -1.236390766 | 10.60999103 | -4.513261788 | 0.000217384 | 0.001191612 | 0.390824648 |
| CANX | 1.337982117 | 8.275874195 | 4.512357311 | 0.00021784 | 0.001193739 | 0.388758763 |
| GFM2 | 0.640422675 | 8.723057227 | 4.512216149 | 0.000217911 | 0.001193757 | 0.388436338 |
| ERI2 | 1.009333245 | 4.186627545 | 4.511834264 | 0.000218104 | 0.001194441 | 0.387564083 |
| RPGRIP1 | -1.34448528 | 8.888808724 | -4.511611159 | 0.000218216 | 0.001194687 | 0.387054492 |
| RAB8B | 0.836961997 | 9.90754327 | 4.511475176 | 0.000218285 | 0.001194691 | 0.386743894 |
| ACSS2 | -1.17150273 | 9.190403422 | -4.510116288 | 0.000218973 | 0.001198084 | 0.383640054 |
| STAG2 | 0.927121501 | 9.170765488 | 4.509696349 | 0.000219186 | 0.001198877 | 0.382680864 |
| TMEM184C | 0.968415291 | 5.930532514 | 4.508492937 | 0.000219798 | 0.00120185 | 0.37993211 |
| VPS28 | -0.644017812 | 15.23120773 | -4.507224013 | 0.000220445 | 0.001204736 | 0.377033689 |
| RSL1D1 | 0.932302188 | 9.665264605 | 4.507189165 | 0.000220463 | 0.001204736 | 0.37695409 |
| PDIA5 | -0.715115034 | 7.78492908 | -4.506481909 | 0.000220824 | 0.001206336 | 0.375338593 |
| CCDC157 | -0.884475068 | 6.41582958 | -4.505046027 | 0.00022156 | 0.001209979 | 0.37205876 |
| ENHO | -1.083302813 | 8.006203724 | -4.504894682 | 0.000221638 | 0.001210028 | 0.371713056 |
| UBL4B | -3.370722198 | 6.082989142 | -4.504744357 | 0.000221715 | 0.001210073 | 0.371369683 |
| TCTEX1D4 | -1.026000972 | 6.552870453 | -4.50450777 | 0.000221836 | 0.001210361 | 0.370829267 |
| LYRM7 | 1.341632317 | 4.608297441 | 4.503570812 | 0.000222318 | 0.001212569 | 0.368689043 |
| KCNRG | 1.112147494 | 4.894982355 | 4.503453263 | 0.000222379 | 0.001212569 | 0.368420533 |
| C22orf15 | -1.490023392 | 7.305466826 | -4.503208897 | 0.000222505 | 0.00121288 | 0.367862345 |
| XKR6 | 1.292197913 | 3.705042039 | 4.503070846 | 0.000222576 | 0.001212891 | 0.367547001 |
| MYOF | -0.85365066 | 7.497865896 | -4.502637392 | 0.0002228 | 0.001213425 | 0.366556888 |
| F8 | 1.960708079 | 6.521044059 | 4.502613457 | 0.000222812 | 0.001213425 | 0.366502212 |
| CEP192 | 0.879103212 | 9.213912429 | 4.502348264 | 0.000222949 | 0.001213795 | 0.365896445 |
| SLC22A1 | -1.36876797 | 6.455991339 | -4.501859918 | 0.000223201 | 0.001214793 | 0.364780936 |
| SRRM5 | -1.272803214 | 5.348157318 | -4.499897312 | 0.000224218 | 0.001219951 | 0.360297792 |
| B3GNT6 | -2.948330566 | 4.285189182 | -4.499295238 | 0.000224531 | 0.001221276 | 0.358922473 |
| PIWIL2 | -2.648372953 | 4.82725979 | -4.497574832 | 0.000225428 | 0.001225466 | 0.354992511 |
| SCGN | -2.210375395 | 4.963651563 | -4.497390809 | 0.000225524 | 0.001225466 | 0.354572139 |
| TGDS | 1.074983777 | 8.001834408 | 4.497378686 | 0.000225531 | 0.001225466 | 0.354544446 |
| WDR13 | -1.213586463 | 9.842732214 | -4.497283198 | 0.000225581 | 0.001225466 | 0.354326318 |
| CTSO | 0.540466749 | 10.60451026 | 4.496985133 | 0.000225736 | 0.001225934 | 0.353645434 |
| CXorf21 | 1.319369551 | 9.09227865 | 4.495531623 | 0.000226498 | 0.001229689 | 0.350325094 |
| SUMO1 | 0.82914751 | 8.908866174 | 4.495152523 | 0.000226697 | 0.00123039 | 0.349459087 |
| IYD | -1.363382973 | 4.644168025 | -4.494296803 | 0.000227147 | 0.001232452 | 0.347504294 |
| INPP1 | -0.615758209 | 7.305818518 | -4.493859562 | 0.000227377 | 0.00123332 | 0.346505462 |
| LCNL1 | -1.376131411 | 5.34425798 | -4.493308824 | 0.000227667 | 0.001234514 | 0.345247352 |
| WNT5B | -1.063793713 | 4.926151899 | -4.492830936 | 0.00022792 | 0.001235501 | 0.344155659 |
| BTF3 | 0.868671085 | 12.36969852 | 4.491626749 | 0.000228556 | 0.001238571 | 0.341404781 |
| SLC26A6 | -0.787997213 | 9.148514088 | -4.489585663 | 0.00022964 | 0.001244059 | 0.336742015 |
| CLDN9 | -0.819579974 | 6.17324235 | -4.488280441 | 0.000230335 | 0.001247443 | 0.333760259 |
| ARMCX3 | 1.141211447 | 6.280438315 | 4.487650795 | 0.000230672 | 0.001248827 | 0.332321834 |
| ZC3H13 | 0.994917138 | 5.527168308 | 4.487536262 | 0.000230733 | 0.001248827 | 0.332060184 |
| MED20 | 0.788259109 | 6.720653078 | 4.486966519 | 0.000231038 | 0.001250092 | 0.330758601 |
| FSD1L | 1.091190888 | 4.78294394 | 4.485864512 | 0.000231629 | 0.001252903 | 0.328241044 |
| MED28 | 1.742895231 | 4.402183272 | 4.485450936 | 0.000231851 | 0.001253719 | 0.327296214 |
| IFT88 | 1.180610733 | 6.824259438 | 4.485152158 | 0.000232011 | 0.001254201 | 0.326613645 |
| EPOR | -1.068347748 | 8.181530398 | -4.484789151 | 0.000232206 | 0.001254871 | 0.325784339 |
| CCNA2 | 1.20862367 | 5.015700315 | 4.483791413 | 0.000232744 | 0.001257389 | 0.323504953 |
| SIRPB2 | -0.929582771 | 8.684096186 | -4.483485449 | 0.000232909 | 0.001257895 | 0.322805959 |
| FAHD1 | -0.650809911 | 7.775618893 | -4.483322108 | 0.000232997 | 0.001257985 | 0.322432796 |
| IFI35 | -1.04165493 | 10.22620624 | -4.482787547 | 0.000233286 | 0.001259157 | 0.32121155 |
| FAM89A | 0.623885754 | 8.206856723 | 4.481502954 | 0.000233982 | 0.001262524 | 0.318276786 |
| SUV39H2 | 1.036677347 | 5.913268229 | 4.481197181 | 0.000234147 | 0.001262644 | 0.317578217 |
| ERAP1 | 1.182082504 | 8.330542978 | 4.480884058 | 0.000234317 | 0.001263173 | 0.316862856 |
| ETFDH | 0.820506312 | 9.064955868 | 4.480628951 | 0.000234456 | 0.001263533 | 0.316280036 |
| HS1BP3 | -0.906222394 | 8.675689131 | -4.478412523 | 0.000235663 | 0.001269651 | 0.311216323 |
| MC5R | -1.829967207 | 5.418084038 | -4.47801551 | 0.00023588 | 0.001270431 | 0.31030929 |
| HDAC5 | -1.774284187 | 9.775825369 | -4.477849399 | 0.000235971 | 0.001270531 | 0.309929783 |
| CLDN10 | -2.870873018 | 3.852776264 | -4.477539816 | 0.000236141 | 0.001271053 | 0.309222493 |
| KIAA1324 | -1.272570521 | 9.883914788 | -4.476209503 | 0.00023687 | 0.001274588 | 0.306183173 |
| CCDC124 | -1.293439244 | 10.1081402 | -4.474439182 | 0.000237844 | 0.001279437 | 0.302138539 |
| OCM2 | -0.795248327 | 7.713442818 | -4.473420802 | 0.000238406 | 0.001281814 | 0.299811838 |
| CDR2L | -1.446828282 | 6.841454908 | -4.473374573 | 0.000238431 | 0.001281814 | 0.299706218 |
| IL34 | -1.614786299 | 4.541247013 | -4.472591756 | 0.000238864 | 0.00128375 | 0.297917699 |
| DEGS1 | 0.609595403 | 10.61036327 | 4.472076943 | 0.000239149 | 0.00128489 | 0.296741488 |
| PRLHR | -1.722765762 | 9.09435239 | -4.471451249 | 0.000239497 | 0.001286361 | 0.295311941 |
| CNOT6 | 0.953608908 | 7.968101841 | 4.471099674 | 0.000239692 | 0.001287017 | 0.294508681 |
| CCNJL | -1.655088861 | 9.436813652 | -4.470765926 | 0.000239877 | 0.00128762 | 0.293746149 |
| ATP1B1 | 1.481318288 | 7.851206233 | 4.469718016 | 0.000240461 | 0.001290168 | 0.291351924 |
| RPS27L | 2.28160795 | 10.81554906 | 4.469649605 | 0.000240499 | 0.001290168 | 0.291195621 |
| VMA21 | 0.548529905 | 10.77811067 | 4.467551936 | 0.000241671 | 0.001296062 | 0.286402903 |
| CASP9 | -0.873397189 | 10.23894172 | -4.466709226 | 0.000242144 | 0.0012982 | 0.284477476 |
| MICAL1 | -0.687789811 | 10.26393259 | -4.465317041 | 0.000242926 | 0.001302 | 0.281296585 |
| RNF125 | 0.889185431 | 10.66868073 | 4.463825559 | 0.000243768 | 0.001306111 | 0.277888789 |
| TM6SF2 | -2.380432182 | 6.113631963 | -4.463433186 | 0.00024399 | 0.001306902 | 0.276992277 |
| DIABLO | 1.527979298 | 14.13248749 | 4.461245095 | 0.00024523 | 0.001313148 | 0.271992778 |
| ZNF124 | 1.097345801 | 5.849000251 | 4.46110871 | 0.000245308 | 0.001313163 | 0.271681154 |
| ABT1 | 0.854037619 | 7.302628412 | 4.460910151 | 0.000245421 | 0.001313368 | 0.271227471 |
| MRPL34 | 0.607116581 | 12.73119823 | 4.460252952 | 0.000245795 | 0.001314971 | 0.269725843 |
| ALAS2 | -1.668916497 | 17.2233671 | -4.459343806 | 0.000246314 | 0.001317344 | 0.267648535 |
| CDYL | 1.2506497 | 7.086889609 | 4.458894919 | 0.00024657 | 0.001318315 | 0.266622869 |
| LRP10 | -1.119079038 | 9.467172322 | -4.45841143 | 0.000246847 | 0.001319393 | 0.265518139 |
| ARRB1 | -0.962540051 | 8.946320957 | -4.457098157 | 0.0002476 | 0.001323014 | 0.262517407 |
| ARL2BP | 0.669760424 | 9.182892877 | 4.456782663 | 0.000247781 | 0.00132358 | 0.261796525 |
| FUNDC2 | -1.02916769 | 9.358657316 | -4.454681191 | 0.000248991 | 0.001329641 | 0.256994764 |
| TAF1 | 0.933130392 | 6.777024642 | 4.453662571 | 0.00024958 | 0.00133238 | 0.254667249 |
| KCTD3 | 0.894405008 | 5.508105514 | 4.452939336 | 0.000249999 | 0.001333873 | 0.253014671 |
| TNKS1BP1 | -1.478019812 | 4.603615063 | -4.452917897 | 0.000250011 | 0.001333873 | 0.252965683 |
| TAS2R20 | 2.061892279 | 2.968061458 | 4.45242723 | 0.000250296 | 0.001334986 | 0.251844515 |
| GMPS | 0.561462772 | 10.4118731 | 4.451916609 | 0.000250592 | 0.001336162 | 0.250677751 |
| ZDHHC22 | -1.223239235 | 4.430635942 | -4.450695186 | 0.000251303 | 0.001339378 | 0.247886795 |
| SNX30 | 0.604647349 | 8.969752332 | 4.450618538 | 0.000251348 | 0.001339378 | 0.247711653 |
| OTP | -4.251470751 | 5.957960477 | -4.450205971 | 0.000251588 | 0.001340149 | 0.246768931 |
| C11orf87 | -2.976552585 | 6.700552634 | -4.450109112 | 0.000251645 | 0.001340149 | 0.246547607 |
| LMO2 | 0.814177255 | 12.14560889 | 4.449468662 | 0.000252019 | 0.001341734 | 0.245084164 |
| THNSL1 | 1.361773309 | 4.356334576 | 4.449267589 | 0.000252136 | 0.001341954 | 0.244624708 |
| SLC5A2 | -1.48728988 | 7.706577388 | -4.448835253 | 0.000252389 | 0.001342893 | 0.243636809 |
| C12orf50 | -0.715815425 | 2.582745952 | -4.448589615 | 0.000252533 | 0.001343252 | 0.243075516 |
| DLAT | 1.305063194 | 6.340997853 | 4.448158858 | 0.000252785 | 0.001343797 | 0.242091221 |
| TRIM58 | -2.155334959 | 13.32505426 | -4.448153809 | 0.000252788 | 0.001343797 | 0.242079683 |
| CSE1L | 0.627737483 | 10.68137518 | 4.445479391 | 0.000254361 | 0.001351748 | 0.235968491 |
| KIFAP3 | 0.87520686 | 9.717993519 | 4.444817376 | 0.000254752 | 0.001353416 | 0.234455738 |
| YIPF5 | 0.637859783 | 7.826207361 | 4.444641211 | 0.000254856 | 0.00135356 | 0.234053188 |
| SELP | -1.214082786 | 8.143811138 | -4.44443289 | 0.000254979 | 0.001353805 | 0.233577159 |
| HSPE1 | 1.323042903 | 11.44883319 | 4.444182744 | 0.000255127 | 0.001354182 | 0.233005553 |
| MOB3C | -0.973690269 | 9.328144546 | -4.443421016 | 0.000255578 | 0.001356167 | 0.231264938 |
| RBM42 | -0.779749292 | 11.07202784 | -4.443135834 | 0.000255747 | 0.001356655 | 0.23061327 |
| P2RY2 | -1.089029025 | 9.255911165 | -4.442600322 | 0.000256065 | 0.001357931 | 0.229389575 |
| RBL1 | 1.045257554 | 6.076947691 | 4.442141012 | 0.000256338 | 0.001358969 | 0.228340003 |
| ENTPD4 | 0.648595435 | 7.729651333 | 4.441861631 | 0.000256504 | 0.001359439 | 0.227701588 |
| ITPKC | -0.797241373 | 9.92746231 | -4.440543942 | 0.000257289 | 0.001363189 | 0.224690516 |
| ITGA1 | 1.609880038 | 3.488532628 | 4.439794032 | 0.000257737 | 0.001365151 | 0.222976876 |
| SPNS2 | -0.988661947 | 8.259951671 | -4.439495274 | 0.000257915 | 0.001365594 | 0.222294175 |
| SAMD9 | 1.361146869 | 7.809986451 | 4.439394564 | 0.000257976 | 0.001365594 | 0.222064039 |
| MPHOSPH6 | 1.072806389 | 6.47404656 | 4.43865518 | 0.000258418 | 0.001367526 | 0.220374444 |
| MAGI3 | 1.217584612 | 5.259988198 | 4.437363074 | 0.000259194 | 0.001371217 | 0.217421792 |
| MAD2L1 | 1.534423528 | 6.652461229 | 4.436997414 | 0.000259414 | 0.001371968 | 0.216586199 |
| WHSC1 | 0.724392437 | 5.911846132 | 4.436842283 | 0.000259507 | 0.001372049 | 0.216231702 |
| HENMT1 | 0.903081964 | 10.62443819 | 4.436623958 | 0.000259639 | 0.001372331 | 0.215732794 |
| PLXND1 | -0.875345157 | 9.869246913 | -4.436227393 | 0.000259878 | 0.001373181 | 0.214826576 |
| PFDN4 | 2.806646878 | 6.162166134 | 4.435224721 | 0.000260483 | 0.001375965 | 0.212535301 |
| CEP128 | 1.022409993 | 6.203208533 | 4.432952386 | 0.000261859 | 0.001382821 | 0.207342588 |
| PLK3 | -0.670906267 | 11.80205052 | -4.43271391 | 0.000262004 | 0.001382832 | 0.206797624 |
| LMBRD1 | 1.893207272 | 11.56693313 | 4.432689965 | 0.000262019 | 0.001382832 | 0.206742904 |
| MTMR3 | -0.710505747 | 8.603762114 | -4.432155741 | 0.000262344 | 0.001384132 | 0.205522094 |
| SRGN | 0.843257078 | 14.77746634 | 4.431125863 | 0.000262971 | 0.001387026 | 0.203168605 |
| TOMM5 | 1.378273788 | 10.52587152 | 4.42924975 | 0.000264118 | 0.001392657 | 0.198881268 |
| LRRC4B | -1.332696335 | 3.813494725 | -4.42810166 | 0.000264822 | 0.001395953 | 0.196257608 |
| ZNF680 | -1.77871428 | 4.572871388 | -4.427924977 | 0.000264931 | 0.001396106 | 0.195853846 |
| RAD51AP1 | 1.55755875 | 3.458774705 | 4.427545818 | 0.000265164 | 0.001396702 | 0.194987373 |
| PRR21 | -2.864194686 | 6.780075723 | -4.4274829 | 0.000265203 | 0.001396702 | 0.194843591 |
| PSMB3 | -0.581544648 | 14.36756907 | -4.426440533 | 0.000265845 | 0.001399664 | 0.192461519 |
| ZNF561 | 0.567151777 | 8.654614159 | 4.426276587 | 0.000265946 | 0.001399777 | 0.192086859 |
| SIGLEC6 | 1.336655628 | 4.675406295 | 4.425365877 | 0.000266508 | 0.001402318 | 0.190005647 |
| MAST3 | -1.065924117 | 10.66848778 | -4.424624447 | 0.000266967 | 0.001404311 | 0.188311279 |
| LAMC3 | -2.317971466 | 6.055503222 | -4.42275682 | 0.000268126 | 0.001409987 | 0.184043227 |
| GPR119 | -1.890817317 | 9.211954577 | -4.422571868 | 0.000268241 | 0.001410171 | 0.183620557 |
| LRP12 | 1.548542804 | 4.002469947 | 4.421302693 | 0.000269032 | 0.001413906 | 0.180720116 |
| 12-Sep | -1.464445834 | 5.543123176 | -4.420237153 | 0.000269698 | 0.001416982 | 0.178285032 |
| RTKN2 | 1.509326605 | 5.632055952 | 4.419664874 | 0.000270056 | 0.001418441 | 0.176977196 |
| MOB3A | -0.621582722 | 12.86383578 | -4.417260884 | 0.000271567 | 0.001425948 | 0.171483301 |
| PPME1 | 0.869392803 | 5.651462057 | 4.416674322 | 0.000271937 | 0.001427464 | 0.170142811 |
| LCE1D | 1.922935506 | 15.1621599 | 4.415454855 | 0.000272707 | 0.001431081 | 0.167355912 |
| RPS16 | 0.570588268 | 16.79845905 | 4.415112375 | 0.000272924 | 0.001431382 | 0.166573227 |
| C4B | -2.651806988 | 8.46012021 | -4.415098952 | 0.000272932 | 0.001431382 | 0.16654255 |
| GEMIN6 | 1.258257595 | 9.091003033 | 4.414827097 | 0.000273104 | 0.001431382 | 0.165921266 |
| DVL3 | -0.613108814 | 8.77300916 | -4.414792581 | 0.000273126 | 0.001431382 | 0.165842386 |
| PPT2 | -0.763316938 | 9.079105561 | -4.414596194 | 0.000273251 | 0.001431382 | 0.165393572 |
| ZNF83 | 1.633345016 | 7.899084774 | 4.414593317 | 0.000273253 | 0.001431382 | 0.165386996 |
| ACAP2 | 0.855150793 | 9.685111006 | 4.413611035 | 0.000273876 | 0.00143398 | 0.163142132 |
| TMEM171 | -1.093765836 | 5.970000038 | -4.413500991 | 0.000273946 | 0.00143398 | 0.162890643 |
| POLR2M | 1.526409222 | 3.086478152 | 4.413426672 | 0.000273993 | 0.00143398 | 0.162720796 |
| ZNF841 | 0.835927212 | 7.625369834 | 4.412164318 | 0.000274797 | 0.001437758 | 0.159835857 |
| IL17F | -1.99881947 | 5.812163095 | -4.411775973 | 0.000275045 | 0.001438626 | 0.158948344 |
| TMEM143 | -0.595401637 | 9.583664895 | -4.411591795 | 0.000275162 | 0.001438813 | 0.158527427 |
| GOLGA4 | 1.22630694 | 7.824449138 | 4.411291468 | 0.000275354 | 0.001439388 | 0.157841068 |
| ZBP1 | -1.023216249 | 9.180657882 | -4.409910405 | 0.000276238 | 0.001443579 | 0.154684812 |
| TP53I11 | -0.933650426 | 7.874235212 | -4.409012982 | 0.000276814 | 0.001445751 | 0.152633851 |
| WWP2 | -0.631814372 | 9.271293779 | -4.409006314 | 0.000276818 | 0.001445751 | 0.152618614 |
| RPL9 | 1.87093528 | 14.88361089 | 4.408710496 | 0.000277008 | 0.001446314 | 0.151942552 |
| TNFSF15 | 0.931470277 | 2.510951318 | 4.407799343 | 0.000277594 | 0.001448533 | 0.149860207 |
| DHX8 | -0.509638609 | 10.28377965 | -4.407794093 | 0.000277598 | 0.001448533 | 0.14984821 |
| AKAP8 | 0.680418596 | 10.14860583 | 4.407616094 | 0.000277712 | 0.001448702 | 0.149441411 |
| OR10W1 | -1.143111506 | 4.119895282 | -4.406970567 | 0.000278129 | 0.001450443 | 0.147966121 |
| CCDC47 | 1.15099671 | 8.006700129 | 4.4064865 | 0.000278441 | 0.001451643 | 0.14685983 |
| FRMD1 | -2.268004414 | 6.651015608 | -4.405611691 | 0.000279007 | 0.001454161 | 0.144860532 |
| HSH2D | -0.761810684 | 11.18109704 | -4.404743356 | 0.00027957 | 0.001456305 | 0.142876026 |
| SH3TC1 | -1.101255719 | 7.780650142 | -4.404637168 | 0.000279639 | 0.001456305 | 0.142633341 |
| HDC | -2.113897661 | 8.935732734 | -4.404593991 | 0.000279667 | 0.001456305 | 0.142534664 |
| PRSS23 | 1.501558691 | 6.754153022 | 4.403736164 | 0.000280224 | 0.001458577 | 0.140574166 |
| SMS | 0.605240933 | 10.06734745 | 4.403666846 | 0.000280269 | 0.001458577 | 0.140415745 |
| NINJ2 | -1.673706161 | 10.79877631 | -4.403476126 | 0.000280393 | 0.001458791 | 0.139979868 |
| ZC3HAV1 | 0.566107584 | 11.08107479 | 4.40311839 | 0.000280626 | 0.001459571 | 0.139162289 |
| CDH5 | -1.942015106 | 5.933420628 | -4.402676395 | 0.000280914 | 0.001460637 | 0.138152142 |
| SLC19A2 | 1.843491967 | 6.181119272 | 4.40240181 | 0.000281093 | 0.001461136 | 0.137524595 |
| ZNF639 | 0.716381719 | 6.941177309 | 4.402262511 | 0.000281184 | 0.001461176 | 0.137206236 |
| COL28A1 | -1.811812419 | 4.9593944 | -4.402059328 | 0.000281317 | 0.001461434 | 0.136741876 |
| TCP10L | -0.924901007 | 6.303346974 | -4.401716506 | 0.000281541 | 0.001462165 | 0.135958377 |
| IMPG2 | -2.349123674 | 6.172617473 | -4.401315585 | 0.000281803 | 0.001463094 | 0.135042098 |
| SH3TC2 | -0.968866772 | 3.207579744 | -4.400806943 | 0.000282135 | 0.001464389 | 0.133879626 |
| PAPOLA | 1.329311531 | 8.851870644 | 4.400432573 | 0.000282381 | 0.00146523 | 0.133024026 |
| CPNE6 | -2.901498372 | 5.231633368 | -4.400133378 | 0.000282577 | 0.001465815 | 0.132340232 |
| AMBN | -1.900582015 | 5.882504189 | -4.399962411 | 0.000282689 | 0.001465964 | 0.131949494 |
| DENND2A | -1.815598443 | 5.39562864 | -4.399442703 | 0.00028303 | 0.001467301 | 0.130761729 |
| SH3GLB2 | -1.196007045 | 7.985912628 | -4.398094592 | 0.000283917 | 0.001471465 | 0.127680684 |
| PPP1R32 | -1.636347405 | 5.770072587 | -4.397810958 | 0.000284104 | 0.001472 | 0.127032451 |
| APOM | -0.912426121 | 8.217594739 | -4.39763049 | 0.000284223 | 0.001472183 | 0.126619999 |
| PLAU | 1.641627541 | 9.45167335 | 4.396592801 | 0.000284908 | 0.001475298 | 0.124248399 |
| PARP10 | -0.665619512 | 12.15088098 | -4.395693194 | 0.000285504 | 0.001477946 | 0.122192379 |
| RPS13 | 0.726475543 | 16.3757542 | 4.395001636 | 0.000285962 | 0.001479884 | 0.120611841 |
| XPO1 | 0.578244201 | 9.127553396 | 4.394678168 | 0.000286177 | 0.00148056 | 0.119872564 |
| COG7 | -1.038898862 | 9.293426113 | -4.392980296 | 0.000287307 | 0.001485969 | 0.115992114 |
| PTGDR2 | -1.410321218 | 7.491240629 | -4.392520257 | 0.000287614 | 0.001486945 | 0.114940703 |
| UBL3 | 1.332478569 | 9.301103658 | 4.392363273 | 0.000287719 | 0.001486945 | 0.114581917 |
| TBC1D8 | -0.8680405 | 8.633971525 | -4.392317409 | 0.000287749 | 0.001486945 | 0.114477097 |
| TSC22D2 | 1.638010256 | 5.91881223 | 4.391545799 | 0.000288265 | 0.001489172 | 0.112713591 |
| C1orf174 | 0.502635378 | 10.9471012 | 4.390831963 | 0.000288743 | 0.001491204 | 0.111082126 |
| MDGA1 | -1.836502015 | 9.205598068 | -4.390284008 | 0.000289111 | 0.001492663 | 0.109829779 |
| GPX7 | 0.682631569 | 7.165833285 | 4.389831199 | 0.000289415 | 0.001493539 | 0.108794884 |
| PRB3 | -1.640202579 | 8.122092088 | -4.38977844 | 0.00028945 | 0.001493539 | 0.108674305 |
| TFDP2 | -1.196084425 | 9.391304072 | -4.388530364 | 0.00029029 | 0.001497432 | 0.105821827 |
| KANSL2 | 0.613799409 | 10.75958522 | 4.387235821 | 0.000291163 | 0.001501498 | 0.102863143 |
| KIAA1549 | -1.314705424 | 3.105483593 | -4.386957802 | 0.000291351 | 0.001502027 | 0.102227728 |
| BTNL2 | -1.04483069 | 6.570026778 | -4.38646124 | 0.000291687 | 0.001503318 | 0.10109283 |
| IGF2BP2 | -1.536603232 | 7.822035915 | -4.385543575 | 0.000292309 | 0.001506083 | 0.098995497 |
| BATF | 0.736712416 | 12.46437836 | 4.38504708 | 0.000292646 | 0.001507378 | 0.097860749 |
| RREB1 | 1.099498498 | 4.822763043 | 4.382767605 | 0.000294199 | 0.001514931 | 0.092650964 |
| CDC42EP4 | -0.939188643 | 5.918990221 | -4.381377902 | 0.00029515 | 0.001519381 | 0.089474759 |
| RASGRP4 | -0.904342205 | 11.01836622 | -4.381142374 | 0.000295311 | 0.001519767 | 0.088936454 |
| HIPK1 | 1.040709087 | 9.824393129 | 4.381004846 | 0.000295405 | 0.001519808 | 0.088622128 |
| TOM1 | -0.609590909 | 13.29901099 | -4.378793133 | 0.000296926 | 0.001526605 | 0.083567185 |
| TASP1 | 0.788909695 | 6.851755617 | 4.378660336 | 0.000297017 | 0.001526605 | 0.083263673 |
| OR1D5 | -1.698985277 | 4.529011348 | -4.378547624 | 0.000297095 | 0.001526605 | 0.083006065 |
| TSC2 | -0.708440713 | 8.508350565 | -4.378487818 | 0.000297136 | 0.001526605 | 0.082869375 |
| ABCB6 | -0.740039697 | 8.221415481 | -4.378452738 | 0.000297161 | 0.001526605 | 0.0827892 |
| PPP1R42 | -1.282735691 | 3.318256367 | -4.37815438 | 0.000297367 | 0.001527217 | 0.082107291 |
| OR2AG2 | -2.154244852 | 7.899887518 | -4.377726976 | 0.000297662 | 0.001528286 | 0.081130442 |
| GPM6A | 1.54573552 | 3.78914784 | 4.374674107 | 0.000299779 | 0.001538707 | 0.074152976 |
| NIPBL | 0.710761741 | 10.38018744 | 4.373940202 | 0.00030029 | 0.001540881 | 0.072475599 |
| RANBP9 | 0.997166497 | 8.182394484 | 4.373265973 | 0.00030076 | 0.001542845 | 0.070934615 |
| ADAM33 | -2.094560805 | 7.494218889 | -4.373133639 | 0.000300853 | 0.001542869 | 0.070632159 |
| KIAA2026 | 0.87206188 | 7.320967108 | 4.371477629 | 0.000302012 | 0.001548361 | 0.066847261 |
| NELL1 | -1.775190989 | 6.004500971 | -4.37105827 | 0.000302306 | 0.001549417 | 0.065888793 |
| EIF5 | 0.722059683 | 9.225876588 | 4.370324568 | 0.000302821 | 0.001551606 | 0.064211875 |
| SSBP1 | 0.653031046 | 11.27517484 | 4.369819934 | 0.000303176 | 0.001552973 | 0.063058504 |
| DCAF4L2 | -1.93740861 | 4.38689207 | -4.369246082 | 0.000303581 | 0.001554591 | 0.061746931 |
| CCNT1 | 0.565409849 | 8.367536949 | 4.368595504 | 0.000304039 | 0.001556271 | 0.060259997 |
| MPHOSPH9 | 1.263172086 | 6.031274626 | 4.368445071 | 0.000304146 | 0.001556271 | 0.059916173 |
| PON3 | -1.894605483 | 5.920001685 | -4.368404571 | 0.000304174 | 0.001556271 | 0.059823608 |
| TOR1AIP1 | 0.804098547 | 9.539985842 | 4.367277882 | 0.000304971 | 0.001559894 | 0.05724849 |
| CNPY2 | 0.668142773 | 12.38692106 | 4.367079372 | 0.000305112 | 0.001560159 | 0.056794782 |
| NOC2L | -0.70773998 | 8.39480282 | -4.366636747 | 0.000305425 | 0.001561309 | 0.055783137 |
| DHTKD1 | -0.670815765 | 9.016177161 | -4.365170432 | 0.000306467 | 0.001565783 | 0.052431778 |
| POLG2 | 0.791151791 | 10.37430311 | 4.365063861 | 0.000306543 | 0.001565783 | 0.052188204 |
| C14orf1 | 0.539249183 | 6.539375766 | 4.365029069 | 0.000306568 | 0.001565783 | 0.052108684 |
| HSPA8 | 0.887015613 | 11.00698839 | 4.363455787 | 0.00030769 | 0.001571057 | 0.048512842 |
| ADAMTS7 | 1.985380346 | 14.04765926 | 4.363165544 | 0.000307897 | 0.00157166 | 0.047849472 |
| WDR11 | 1.036986035 | 8.275249726 | 4.362075545 | 0.000308677 | 0.001575185 | 0.045358205 |
| LILRA1 | -0.936862838 | 9.525546621 | -4.361489977 | 0.000309097 | 0.001576871 | 0.04401985 |
| MAP2K4 | 0.59105504 | 10.33851408 | 4.360905603 | 0.000309517 | 0.001578554 | 0.042684221 |
| CD226 | 1.755977558 | 6.449907324 | 4.360527415 | 0.000309789 | 0.001579483 | 0.041819847 |
| MEAF6 | 0.624205362 | 10.46615737 | 4.360182132 | 0.000310037 | 0.001580292 | 0.041030678 |
| MYB | 1.385130201 | 7.447397223 | 4.35904546 | 0.000310857 | 0.001584009 | 0.038432734 |
| ANKRD7 | -1.479267325 | 4.261097006 | -4.357071147 | 0.000312285 | 0.001590827 | 0.033920301 |
| MAGEA8 | -2.002010049 | 4.276278127 | -4.356734626 | 0.000312529 | 0.001591609 | 0.033151158 |
| DUSP19 | 1.579622702 | 2.866232766 | 4.356573009 | 0.000312647 | 0.001591746 | 0.03278177 |
| WNT9B | -1.744454415 | 4.635317707 | -4.356331661 | 0.000312822 | 0.001592177 | 0.032230152 |
| PACS1 | -0.674688595 | 10.80662186 | -4.355371616 | 0.00031352 | 0.001595269 | 0.0300359 |
| INS | -1.436441241 | 5.878971581 | -4.355078916 | 0.000313733 | 0.001595892 | 0.029366913 |
| FECH | -1.977291647 | 9.421384804 | -4.354284141 | 0.000314313 | 0.001598378 | 0.027550397 |
| RUFY3 | 1.223419202 | 2.752102298 | 4.354118884 | 0.000314433 | 0.001598529 | 0.027172691 |
| ZC3H3 | -0.547860535 | 11.89091527 | -4.352011422 | 0.000315976 | 0.001605566 | 0.022355936 |
| CC2D1A | -0.829248282 | 8.554085565 | -4.351978482 | 0.000316 | 0.001605566 | 0.022280649 |
| WDR62 | -0.623460131 | 7.740394148 | -4.351229065 | 0.000316551 | 0.001607898 | 0.020567804 |
| C9orf116 | -1.221829302 | 5.672704541 | -4.349700676 | 0.000317676 | 0.00161315 | 0.017074563 |
| TMTC3 | 1.184211633 | 5.113821795 | 4.348423353 | 0.00031862 | 0.001617475 | 0.014155152 |
| OCEL1 | -0.743122833 | 9.372802678 | -4.347419098 | 0.000319364 | 0.001620784 | 0.011859857 |
| ITGA5 | -0.723148674 | 9.867933523 | -4.34720609 | 0.000319522 | 0.001621118 | 0.011373015 |
| IPMK | 1.59985258 | 6.16344536 | 4.344857319 | 0.00032127 | 0.001629515 | 0.006004741 |
| MAPK15 | -1.288299516 | 4.510856496 | -4.344663118 | 0.000321415 | 0.00162978 | 0.005560883 |
| PDK2 | -0.830515718 | 8.821499421 | -4.343878961 | 0.000322001 | 0.001631866 | 0.00376864 |
| KRTAP2-1 | -3.02522531 | 9.155830674 | -4.343741236 | 0.000322104 | 0.001631866 | 0.003453862 |
| TRIM62 | -0.904066799 | 5.480780479 | -4.343740225 | 0.000322104 | 0.001631866 | 0.00345155 |
| ZBED4 | 0.913474979 | 6.019650572 | 4.343146695 | 0.000322549 | 0.001633646 | 0.002095 |
| ABCG2 | -1.319942216 | 4.845635403 | -4.34244956 | 0.000323071 | 0.001635822 | 0.000501654 |
| NCOA7 | 0.625889026 | 10.1448353 | 4.342090742 | 0.000323341 | 0.001636715 | -0.00031845 |
| SLC16A1 | 1.003120983 | 4.594549465 | 4.341254884 | 0.000323969 | 0.001639424 | -0.00222885 |
| LPL | 1.408214313 | 5.031945147 | 4.340872253 | 0.000324257 | 0.001640409 | -0.00310338 |
| HERPUD1 | 0.74657904 | 13.77848313 | 4.340084689 | 0.000324851 | 0.00164294 | -0.0049034 |
| RUFY1 | -0.678853124 | 12.04516884 | -4.339610475 | 0.000325209 | 0.001644278 | -0.00598724 |
| PPP2R4 | -0.986271806 | 9.231984938 | -4.339245866 | 0.000325484 | 0.001645198 | -0.00682058 |
| MTRR | 1.349779292 | 6.430022287 | 4.338369693 | 0.000326147 | 0.001647602 | -0.00882312 |
| PEBP1 | 0.698517776 | 11.6026218 | 4.337471264 | 0.000326829 | 0.00165057 | -0.01087653 |
| DSE | 0.819744618 | 9.926283479 | 4.336793675 | 0.000327344 | 0.001652695 | -0.01242519 |
| CHRFAM7A | -2.478408385 | 7.100448175 | -4.335500153 | 0.000328329 | 0.001656208 | -0.0153816 |
| CDC42EP1 | -1.397817241 | 9.864889643 | -4.335408277 | 0.000328399 | 0.001656208 | -0.01559159 |
| MSL2 | 0.753594781 | 7.598850715 | 4.335367484 | 0.00032843 | 0.001656208 | -0.01568482 |
| GPX2 | -1.99773258 | 6.560038339 | -4.335298225 | 0.000328483 | 0.001656208 | -0.01584312 |
| IL15RA | -1.326875118 | 8.402105742 | -4.335261857 | 0.00032851 | 0.001656208 | -0.01592624 |
| CPNE2 | -0.877112929 | 8.417052026 | -4.334552512 | 0.000329052 | 0.001658464 | -0.01754748 |
| ZNF518B | 0.630944006 | 8.976679231 | 4.334420769 | 0.000329153 | 0.001658496 | -0.01784858 |
| TNFRSF10C | -0.938264224 | 14.78470337 | -4.333904471 | 0.000329548 | 0.001660011 | -0.0190286 |
| GNA12 | -1.168210632 | 9.526237678 | -4.33376382 | 0.000329655 | 0.001660078 | -0.01935007 |
| GOT1L1 | -1.065075888 | 3.088672138 | -4.333003913 | 0.000330238 | 0.001662534 | -0.02108687 |
| KEAP1 | -0.65852234 | 9.527136608 | -4.332660142 | 0.000330502 | 0.001663386 | -0.02187257 |
| ITK | 1.351369164 | 9.827649248 | 4.332345114 | 0.000330744 | 0.001664128 | -0.02259258 |
| FUT8 | 0.839804899 | 8.30094517 | 4.332144807 | 0.000330898 | 0.001664426 | -0.02305039 |
| BTF3L4 | 0.670250468 | 7.953449858 | 4.33192799 | 0.000331064 | 0.001664788 | -0.02354593 |
| TMPRSS13 | -0.535731743 | 5.124462891 | -4.331757219 | 0.000331196 | 0.001664973 | -0.02393623 |
| TNFSF9 | 2.157043317 | 4.896218069 | 4.331113752 | 0.000331691 | 0.001666987 | -0.0254069 |
| NUP50 | 0.92442201 | 9.590278591 | 4.330107115 | 0.000332468 | 0.001670412 | -0.02770761 |
| OR2L2 | 1.935048754 | 3.594826012 | 4.329921232 | 0.000332611 | 0.001670656 | -0.02813245 |
| C4orf33 | 0.920110437 | 8.275558814 | 4.328932947 | 0.000333376 | 0.001674018 | -0.0303912 |
| FAM170A | -1.289995789 | 4.335692031 | -4.328586042 | 0.000333644 | 0.001674889 | -0.03118407 |
| FAM193B | -0.579273138 | 8.806903539 | -4.328246942 | 0.000333907 | 0.00167573 | -0.03195909 |
| RASL10A | -1.014943518 | 7.407077407 | -4.327638872 | 0.000334379 | 0.00167762 | -0.03334885 |
| PCID2 | 0.642484256 | 10.08876614 | 4.327472466 | 0.000334509 | 0.00167779 | -0.03372917 |
| GSTZ1 | -0.727147604 | 8.301507814 | -4.326988126 | 0.000334885 | 0.0016792 | -0.03483614 |
| MSRB2 | -0.951778907 | 11.03186477 | -4.326558731 | 0.000335219 | 0.001680397 | -0.03581753 |
| DHX15 | 0.821868759 | 11.23070753 | 4.32636356 | 0.000335371 | 0.00168068 | -0.0362636 |
| KRT20 | -1.545611412 | 4.520355658 | -4.325972029 | 0.000335677 | 0.00168173 | -0.03715845 |
| SYNJ2BP | 0.568908839 | 8.204622374 | 4.325508983 | 0.000336038 | 0.001682673 | -0.03821675 |
| PTGS2 | 1.185460423 | 6.990260493 | 4.325485557 | 0.000336056 | 0.001682673 | -0.03827029 |
| ICK | 1.066729856 | 8.008879256 | 4.324945051 | 0.000336478 | 0.001683828 | -0.03950563 |
| LUZP6 | 2.061534435 | 9.710936822 | 4.324815829 | 0.000336579 | 0.001683854 | -0.03980096 |
| DDX18 | 0.668558046 | 8.717883594 | 4.323580696 | 0.000337547 | 0.001688213 | -0.04262388 |
| CRYZL1 | 0.988792299 | 5.575712171 | 4.322812568 | 0.000338149 | 0.001690747 | -0.04437944 |
| PIK3CG | -0.885911317 | 10.60983782 | -4.322334104 | 0.000338525 | 0.001692146 | -0.04547297 |
| CHAF1A | -0.804090918 | 8.68677663 | -4.322194385 | 0.000338635 | 0.001692161 | -0.0457923 |
| CAB39 | 1.262535314 | 9.19147971 | 4.322085843 | 0.000338721 | 0.001692161 | -0.04604037 |
| PPP2R5D | -0.533741543 | 10.33258491 | -4.321360812 | 0.000339292 | 0.001694532 | -0.04769743 |
| WDSUB1 | 0.634703322 | 8.17371452 | 4.320672993 | 0.000339834 | 0.001696448 | -0.04926944 |
| ATF4 | 0.532958301 | 14.83885036 | 4.320629841 | 0.000339868 | 0.001696448 | -0.04936806 |
| PRSS3 | -1.723592993 | 6.243504295 | -4.320363996 | 0.000340078 | 0.001697015 | -0.04997565 |
| HES6 | -0.970358243 | 9.757429398 | -4.319913639 | 0.000340434 | 0.001698129 | -0.05100494 |
| ZNF266 | 0.775927019 | 9.477944393 | 4.319837167 | 0.000340495 | 0.001698129 | -0.05117972 |
| EIF3J | 0.89884949 | 7.828556348 | 4.319478921 | 0.000340778 | 0.001699061 | -0.05199849 |
| MMP23B | -0.774580156 | 8.454637121 | -4.31790197 | 0.000342029 | 0.001704814 | -0.05560259 |
| KRTAP10-12 | -2.03551873 | 9.27087163 | -4.316908678 | 0.000342819 | 0.001707912 | -0.05787274 |
| 10-Sep | 1.150375063 | 6.15694059 | 4.31687658 | 0.000342845 | 0.001707912 | -0.0579461 |
| GMNN | 1.039202666 | 8.055561394 | 4.315774755 | 0.000343724 | 0.001711805 | -0.06046429 |
| PNPO | -0.601206365 | 10.67318681 | -4.315293376 | 0.000344108 | 0.001713235 | -0.06156447 |
| GRIN2D | -1.427070329 | 4.721576708 | -4.31482361 | 0.000344484 | 0.001714621 | -0.06263811 |
| FHL3 | -1.14193687 | 8.603060017 | -4.314613134 | 0.000344653 | 0.001714974 | -0.06311915 |
| ZNF879 | 1.127337641 | 6.128126089 | 4.314411352 | 0.000344814 | 0.001715293 | -0.06358031 |
| ZNF76 | 0.929951164 | 5.943581334 | 4.313827313 | 0.000345283 | 0.001717136 | -0.06491511 |
| UMODL1 | -1.027227544 | 4.607383075 | -4.313322646 | 0.000345688 | 0.001718297 | -0.06606851 |
| ODF3 | -1.33433262 | 6.098597487 | -4.313293219 | 0.000345711 | 0.001718297 | -0.06613576 |
| PCYT1B | -1.290144387 | 3.600287644 | -4.311964891 | 0.00034678 | 0.001723121 | -0.0691716 |
| E2F1 | -0.976292531 | 6.097209765 | -4.311493646 | 0.00034716 | 0.001724522 | -0.07024861 |
| MCTS1 | 0.884363574 | 10.82766958 | 4.310776164 | 0.000347739 | 0.001726911 | -0.07188838 |
| CWC22 | 1.225372986 | 7.887935371 | 4.309982469 | 0.000348381 | 0.00172961 | -0.07370233 |
| FBXL20 | -0.798238646 | 11.33424221 | -4.309581732 | 0.000348705 | 0.001730733 | -0.07461819 |
| CCDC71 | -0.640056253 | 7.016456201 | -4.309069455 | 0.000349121 | 0.001732305 | -0.07578896 |
| COQ10B | 1.093719918 | 6.931283148 | 4.308648668 | 0.000349462 | 0.001733355 | -0.07675065 |
| EIF4EBP1 | -0.841299008 | 11.28904252 | -4.308566014 | 0.000349529 | 0.001733355 | -0.07693955 |
| MAPKAPK3 | -0.870727045 | 12.20065978 | -4.307172061 | 0.000350663 | 0.001738488 | -0.08012533 |
| CRYBA1 | 1.331962912 | 3.153966088 | 4.30659396 | 0.000351135 | 0.001740334 | -0.08144654 |
| FAM122A | -0.513912105 | 9.1925156 | -4.305827042 | 0.000351761 | 0.001742947 | -0.08319927 |
| TNK1 | -1.330365161 | 8.668955758 | -4.305048858 | 0.000352398 | 0.001745609 | -0.08497774 |
| CASP7 | 0.779256691 | 8.255272247 | 4.304664631 | 0.000352712 | 0.001746676 | -0.08585586 |
| UPRT | 0.991515567 | 7.400543604 | 4.304481148 | 0.000352863 | 0.001746929 | -0.08627519 |
| RRP15 | 0.981255567 | 6.465779499 | 4.304200615 | 0.000353093 | 0.001747576 | -0.08691632 |
| TRIM32 | 0.826556417 | 6.335405869 | 4.304024761 | 0.000353237 | 0.001747799 | -0.08731822 |
| CATSPER1 | -1.111079592 | 9.288179905 | -4.302439113 | 0.000354541 | 0.001753757 | -0.09094206 |
| ZNF606 | 1.454952742 | 4.563327189 | 4.30193054 | 0.00035496 | 0.001755337 | -0.09210435 |
| TMEM235 | -1.49194634 | 5.61299929 | -4.30116119 | 0.000355596 | 0.001757984 | -0.09386262 |
| ZNF37A | 0.926837319 | 9.330207867 | 4.30050896 | 0.000356135 | 0.001760155 | -0.09535321 |
| SLC4A4 | 1.672000105 | 4.656734584 | 4.300373146 | 0.000356247 | 0.001760216 | -0.0956636 |
| TRIM11 | -0.593809047 | 9.460608655 | -4.300056753 | 0.000356509 | 0.001760754 | -0.09638668 |
| KRAS | 0.808284822 | 8.819362949 | 4.3 | 0.000356556 | 0.001760754 | -0.09651638 |
| MAP2K7 | -0.563114855 | 12.52498881 | -4.299797968 | 0.000356724 | 0.001760848 | -0.0969781 |
| IMMP1L | 1.168111002 | 7.009379368 | 4.299735359 | 0.000356776 | 0.001760848 | -0.09712119 |
| BAG2 | 1.100872094 | 7.226693645 | 4.299494299 | 0.000356976 | 0.00176134 | -0.0976721 |
| ADNP | 0.516267712 | 10.34400521 | 4.299281496 | 0.000357152 | 0.001761717 | -0.09815843 |
| CHST8 | -1.591777648 | 7.608864657 | -4.298857658 | 0.000357504 | 0.001762959 | -0.09912706 |
| ZNF442 | 1.008727467 | 4.852187308 | 4.298368517 | 0.000357911 | 0.001764469 | -0.10024493 |
| TPT1 | 0.904730514 | 15.65464296 | 4.298145879 | 0.000358096 | 0.001764887 | -0.10075374 |
| LRRFIP1 | 0.701206007 | 9.373727882 | 4.297073786 | 0.000358989 | 0.001768794 | -0.10320386 |
| GPR183 | 1.166457765 | 11.67731188 | 4.296771846 | 0.000359241 | 0.00176954 | -0.1038939 |
| TCF21 | -2.0409149 | 3.583354042 | -4.295941899 | 0.000359935 | 0.001772349 | -0.10579062 |
| RGAG4 | -0.895289454 | 7.524545518 | -4.295848248 | 0.000360013 | 0.001772349 | -0.10600465 |
| CHD1 | 1.018804865 | 9.140181656 | 4.293943608 | 0.00036161 | 0.001779261 | -0.1103574 |
| NPAS2 | -1.640924113 | 5.128625258 | -4.293927258 | 0.000361624 | 0.001779261 | -0.11039477 |
| ELOVL1 | -0.697839212 | 9.634096648 | -4.293812056 | 0.000361721 | 0.001779261 | -0.11065804 |
| STRN4 | -1.0619902 | 10.76883577 | -4.293113185 | 0.000362309 | 0.001781655 | -0.11225519 |
| IGF1 | -1.846566762 | 5.103418798 | -4.292358167 | 0.000362945 | 0.001784285 | -0.11398065 |
| ACO2 | -0.63513426 | 11.83032813 | -4.292094776 | 0.000363167 | 0.001784878 | -0.11458259 |
| MESP2 | -0.887257114 | 7.299298579 | -4.291940784 | 0.000363297 | 0.001785018 | -0.1149345 |
| SLC30A5 | 0.794874035 | 7.732156056 | 4.291650583 | 0.000363542 | 0.001785723 | -0.11559771 |
| RBM45 | 0.547047978 | 9.234807629 | 4.291181974 | 0.000363938 | 0.00178717 | -0.11666862 |
| CNGA3 | -1.163153254 | 2.859801274 | -4.289519118 | 0.000365348 | 0.001793589 | -0.12046876 |
| UBR5 | 1.295555833 | 7.505324572 | 4.289111807 | 0.000365694 | 0.001794787 | -0.12139959 |
| OR2B2 | -1.569427619 | 6.246826679 | -4.288962468 | 0.00036582 | 0.001794856 | -0.12174087 |
| USP33 | 0.996391384 | 10.7841334 | 4.288855171 | 0.000365912 | 0.001794856 | -0.12198607 |
| FOXK2 | -0.701045824 | 8.621536378 | -4.288154493 | 0.000366508 | 0.001796967 | -0.12358733 |
| CYHR1 | -0.91936701 | 9.664466194 | -4.288094634 | 0.000366559 | 0.001796967 | -0.12372412 |
| CRYZ | 1.734344172 | 6.630865183 | 4.287989296 | 0.000366649 | 0.001796967 | -0.12396485 |
| FAM178B | -1.318203176 | 7.775597458 | -4.287528758 | 0.000367041 | 0.001798391 | -0.12501731 |
| IQCB1 | 0.821019943 | 8.287917739 | 4.286791129 | 0.000367671 | 0.001800974 | -0.126703 |
| RAVER2 | 1.314850382 | 4.294076218 | 4.286110858 | 0.000368253 | 0.001803322 | -0.1282576 |
| ZBTB16 | -1.111758075 | 8.487898567 | -4.28560198 | 0.000368689 | 0.001804953 | -0.12942052 |
| ZMYND11 | 2.03487462 | 7.030147547 | 4.28518496 | 0.000369046 | 0.001806201 | -0.13037352 |
| TUBGCP2 | -0.572713116 | 9.601418513 | -4.283846155 | 0.000370196 | 0.001811325 | -0.13343303 |
| ZUFSP | 1.102384273 | 7.965787124 | 4.283505863 | 0.000370489 | 0.001812254 | -0.13421068 |
| RRAGC | -0.865735767 | 10.6698911 | -4.283124025 | 0.000370818 | 0.001813359 | -0.13508328 |
| PDIA4 | 1.764916241 | 7.518128378 | 4.282598427 | 0.000371272 | 0.001815071 | -0.13628439 |
| KLHDC8B | -1.692014669 | 6.097471274 | -4.28145699 | 0.000372258 | 0.001819387 | -0.13889284 |
| TET2 | 0.715653233 | 9.390360955 | 4.280334753 | 0.00037323 | 0.001823632 | -0.1414574 |
| ANKMY2 | 0.981006323 | 6.601824245 | 4.279937918 | 0.000373574 | 0.001824807 | -0.14236425 |
| GGN | -0.679372454 | 6.211641757 | -4.279005195 | 0.000374385 | 0.00182826 | -0.14449572 |
| PTPN5 | -1.458851518 | 5.596361552 | -4.277898103 | 0.00037535 | 0.001832462 | -0.14702564 |
| MSH6 | 0.655292092 | 10.4132663 | 4.277072502 | 0.000376071 | 0.001834963 | -0.1489123 |
| USP19 | -0.631369191 | 10.44611573 | -4.27666111 | 0.000376431 | 0.00183585 | -0.1498524 |
| GPAM | 1.062805257 | 6.661846888 | 4.276625784 | 0.000376461 | 0.00183585 | -0.14993313 |
| GPX4 | -1.207068295 | 12.94493646 | -4.275290662 | 0.000377632 | 0.001841046 | -0.15298411 |
| FAM53B | -0.754611699 | 9.804455172 | -4.274411572 | 0.000378404 | 0.0018443 | -0.15499297 |
| CREM | 0.811070736 | 6.051949738 | 4.273821151 | 0.000378924 | 0.001846321 | -0.15634217 |
| GOLGA6A | 0.602861947 | 3.905986565 | 4.273130383 | 0.000379533 | 0.001848776 | -0.15792068 |
| GOLGA7 | 0.622983653 | 10.84511316 | 4.272949491 | 0.000379692 | 0.001849041 | -0.15833404 |
| FAM57B | 2.359556223 | 7.802860417 | 4.27268207 | 0.000379928 | 0.001849679 | -0.15894514 |
| MYLIP | 0.728978469 | 11.12391489 | 4.272470109 | 0.000380116 | 0.001850078 | -0.1594295 |
| UGT3A1 | -1.597321284 | 6.581211614 | -4.271314807 | 0.000381138 | 0.00185454 | -0.16206951 |
| GPC4 | -3.55831764 | 7.910691548 | -4.270626114 | 0.000381748 | 0.001856997 | -0.16364326 |
| ST8SIA4 | 1.005465604 | 9.039046092 | 4.269960281 | 0.00038234 | 0.001859359 | -0.16516476 |
| XYLT1 | 0.979991467 | 6.496456381 | 4.269777587 | 0.000382502 | 0.001859634 | -0.16558224 |
| BIRC6 | 0.911309149 | 7.335746983 | 4.268849908 | 0.000383328 | 0.001863134 | -0.16770208 |
| FAR2 | -0.857325908 | 8.319563804 | -4.267582507 | 0.000384459 | 0.001868115 | -0.1705982 |
| UHRF1BP1 | 0.696943284 | 8.537055306 | 4.267240588 | 0.000384764 | 0.001869084 | -0.17137951 |
| SAMD4A | -1.078021683 | 7.523096416 | -4.26711409 | 0.000384878 | 0.001869117 | -0.17166857 |
| RPL24 | 0.920494553 | 15.16865802 | 4.266918785 | 0.000385052 | 0.001869449 | -0.17211486 |
| DNAJA2 | 0.792673076 | 7.747642558 | 4.265300083 | 0.000386504 | 0.001875979 | -0.1758137 |
| MMACHC | -1.634241742 | 7.009243386 | -4.264795553 | 0.000386958 | 0.001877662 | -0.17696658 |
| CCDC158 | -1.728434782 | 5.500615901 | -4.263675769 | 0.000387966 | 0.001882037 | -0.17952533 |
| MSH2 | 1.170295646 | 8.204750554 | 4.262621513 | 0.000388918 | 0.001886135 | -0.18193434 |
| TNFRSF6B | -0.872940408 | 6.030007321 | -4.261433573 | 0.000389994 | 0.001890829 | -0.1846488 |
| PCDHGA12 | -1.331526577 | 5.858832535 | -4.261153115 | 0.000390248 | 0.00189127 | -0.18528965 |
| RSG1 | -0.931947579 | 7.089100988 | -4.261096154 | 0.0003903 | 0.00189127 | -0.18541981 |
| SPATA7 | 0.708210144 | 6.530810534 | 4.257619108 | 0.000393467 | 0.001906095 | -0.19336481 |
| SORT1 | -0.816143968 | 9.96276249 | -4.257419308 | 0.00039365 | 0.001906455 | -0.19382134 |
| TBL1XR1 | 1.227881314 | 8.944510602 | 4.256675248 | 0.000394332 | 0.00190923 | -0.19552149 |
| HIST1H2AL | 2.177414162 | 4.956406504 | 4.255609049 | 0.00039531 | 0.001913441 | -0.19795769 |
| PRPF18 | 1.368660819 | 8.348188079 | 4.255467027 | 0.000395441 | 0.001913546 | -0.1982822 |
| RPS17 | 1.416725898 | 14.41185737 | 4.255227264 | 0.000395661 | 0.001914087 | -0.19883004 |
| SPTA1 | -1.686625855 | 4.557046838 | -4.255044821 | 0.000395829 | 0.001914372 | -0.19924691 |
| CHRM2 | -1.133111481 | 2.932187207 | -4.254629132 | 0.000396212 | 0.001915696 | -0.20019673 |
| TOB2 | 0.762297463 | 7.971360601 | 4.252900108 | 0.000397808 | 0.001922883 | -0.2041474 |
| ABCC10 | -1.101347904 | 7.919625298 | -4.252213487 | 0.000398443 | 0.001925426 | -0.20571626 |
| RARS | 0.861358202 | 11.96235774 | 4.251843774 | 0.000398786 | 0.001926543 | -0.20656102 |
| AP3M1 | 1.123480027 | 7.722028393 | 4.251669836 | 0.000398947 | 0.001926543 | -0.20695844 |
| HIVEP1 | 0.750317833 | 7.180151806 | 4.251609607 | 0.000399003 | 0.001926543 | -0.20709606 |
| GPX5 | -1.967888187 | 7.05362982 | -4.250227108 | 0.000400288 | 0.001932215 | -0.2102549 |
| RPL23 | 1.652412459 | 14.20873006 | 4.249999937 | 0.000400499 | 0.001932705 | -0.21077395 |
| ISOC2 | -0.796994031 | 8.676307366 | -4.248397436 | 0.000401994 | 0.001939387 | -0.21443543 |
| FAM174B | -1.054905935 | 6.781003559 | -4.247606229 | 0.000402734 | 0.001942168 | -0.21624321 |
| TMEM189 | -0.52865157 | 9.998784758 | -4.247531541 | 0.000402804 | 0.001942168 | -0.21641386 |
| FBXL17 | -1.252954987 | 8.059104266 | -4.247427251 | 0.000402902 | 0.001942168 | -0.21665214 |
| UBTD2 | 1.91537546 | 5.509987169 | 4.246433109 | 0.000403834 | 0.001946129 | -0.21892358 |
| CCDC61 | -0.836213306 | 8.9774248 | -4.243644645 | 0.000406461 | 0.001958251 | -0.22529464 |
| ATG13 | -0.757557922 | 7.849864478 | -4.243473724 | 0.000406622 | 0.001958492 | -0.22568516 |
| PRIMA1 | -2.963206686 | 8.628942924 | -4.241613379 | 0.000408385 | 0.001966443 | -0.22993559 |
| GNG13 | -0.822415402 | 7.207878393 | -4.239656926 | 0.000410247 | 0.001974868 | -0.23440556 |
| GPR173 | -2.296373009 | 5.775734401 | -4.238875461 | 0.000410993 | 0.001977919 | -0.23619098 |
| NUCKS1 | 0.918874521 | 6.414700454 | 4.238174092 | 0.000411664 | 0.001980605 | -0.23779339 |
| KCNJ2 | 0.749349672 | 11.47397426 | 4.237326267 | 0.000412476 | 0.00198397 | -0.23973041 |
| MRPL46 | 0.572329906 | 9.002140032 | 4.236957801 | 0.00041283 | 0.001985128 | -0.24057223 |
| OMA1 | 0.857513507 | 9.945089986 | 4.236802415 | 0.000412979 | 0.001985302 | -0.24092724 |
| ZNF330 | 0.874212329 | 8.700755624 | 4.236266021 | 0.000413494 | 0.001987237 | -0.24215272 |
| DGCR8 | -1.227661802 | 8.210884704 | -4.234969981 | 0.000414742 | 0.001992146 | -0.24511372 |
| CUL4B | 0.629929971 | 9.07465649 | 4.234575457 | 0.000415123 | 0.001993429 | -0.24601507 |
| KCTD11 | -1.084169317 | 8.485048047 | -4.233902668 | 0.000415773 | 0.001996005 | -0.24755214 |
| SSB | 1.394294946 | 10.47420232 | 4.233044939 | 0.000416603 | 0.001999444 | -0.24951173 |
| CNKSR3 | -0.818795548 | 2.786518962 | -4.232599645 | 0.000417035 | 0.00200097 | -0.25052905 |
| RPS20 | 0.832985041 | 15.73276856 | 4.231620237 | 0.000417985 | 0.002004985 | -0.2527666 |
| KIAA1841 | 2.318268303 | 5.25357492 | 4.231093971 | 0.000418497 | 0.002006892 | -0.2539689 |
| CTCF | 0.805828843 | 8.15406859 | 4.230880064 | 0.000418705 | 0.002007344 | -0.25445759 |
| MT1X | -1.33035075 | 11.50567922 | -4.22735115 | 0.000422155 | 0.002023332 | -0.26251956 |
| CDK5 | -0.960039544 | 9.144413905 | -4.226839849 | 0.000422658 | 0.002025187 | -0.26368764 |
| TRIM54 | -1.337798534 | 7.29829572 | -4.226385494 | 0.000423105 | 0.002026642 | -0.26472561 |
| CHI3L1 | -1.601158474 | 12.13878563 | -4.22629677 | 0.000423192 | 0.002026642 | -0.2649283 |
| S1PR1 | 1.213222111 | 11.6575261 | 4.226153915 | 0.000423332 | 0.002026747 | -0.26525465 |
| WNT3A | -1.487754663 | 6.549163135 | -4.226040267 | 0.000423444 | 0.002026747 | -0.26551428 |
| NGFRAP1 | -0.888672802 | 11.62044411 | -4.225754032 | 0.000423726 | 0.002027545 | -0.26616819 |
| FAM19A1 | 1.54127588 | 4.86529598 | 4.225463314 | 0.000424013 | 0.002028364 | -0.26683233 |
| STK31 | 0.776421394 | 2.492870232 | 4.224484 | 0.00042498 | 0.002032435 | -0.26906955 |
| LEKR1 | 0.976501965 | 2.932037221 | 4.224201785 | 0.000425259 | 0.002033217 | -0.26971426 |
| MTHFD2 | 1.021526583 | 8.082271631 | 4.223712301 | 0.000425743 | 0.002034979 | -0.27083247 |
| DDX21 | 0.597330446 | 10.37156393 | 4.222603223 | 0.000426842 | 0.00203968 | -0.2733661 |
| LRRC8B | -0.842513892 | 8.516845952 | -4.222452575 | 0.000426992 | 0.00203984 | -0.27371025 |
| KCNA3 | 1.122691845 | 9.527737947 | 4.221861878 | 0.000427579 | 0.002042089 | -0.27505965 |
| ALS2 | 0.848145735 | 4.981188654 | 4.221498423 | 0.00042794 | 0.002043261 | -0.27588994 |
| ACY1 | -0.646150686 | 9.247594102 | -4.221163841 | 0.000428273 | 0.002044297 | -0.27665426 |
| JDP2 | -0.743104567 | 10.6181751 | -4.220024283 | 0.00042941 | 0.002049165 | -0.27925747 |
| AQP7 | -1.436222311 | 4.452271235 | -4.219364978 | 0.000430069 | 0.002051752 | -0.28076358 |
| KRTAP23-1 | -2.919437318 | 7.466213517 | -4.219021104 | 0.000430413 | 0.002052837 | -0.28154912 |
| GALNT6 | -0.969522805 | 9.57883628 | -4.218728934 | 0.000430705 | 0.002053675 | -0.28221654 |
| GRINA | -1.568787055 | 10.78358265 | -4.217608783 | 0.000431829 | 0.002058383 | -0.28477537 |
| INSL3 | -1.15601756 | 10.51090064 | -4.217511251 | 0.000431927 | 0.002058383 | -0.28499817 |
| BAZ1A | 0.810429327 | 10.33924472 | 4.216468508 | 0.000432975 | 0.002062821 | -0.28738014 |
| MYH14 | -0.872942424 | 9.156958819 | -4.216338165 | 0.000433107 | 0.002062888 | -0.28767789 |
| RNF217 | 1.135018363 | 4.940111577 | 4.216029398 | 0.000433418 | 0.002063811 | -0.28838321 |
| NCKAP1 | 1.428167884 | 4.292871861 | 4.21542325 | 0.000434029 | 0.002066162 | -0.28976785 |
| CCDC96 | -0.971062714 | 8.296717299 | -4.21522198 | 0.000434232 | 0.00206657 | -0.29022761 |
| IFIT5 | 1.166175864 | 9.326348935 | 4.214404308 | 0.000435059 | 0.002069944 | -0.29209542 |
| NR1H3 | -0.79404882 | 9.264463571 | -4.213762034 | 0.000435709 | 0.002072477 | -0.29356255 |
| RPL13A | 0.648743148 | 16.42993719 | 4.212510692 | 0.000436979 | 0.002077955 | -0.29642095 |
| RAB3D | -0.838072918 | 8.151618368 | -4.212112334 | 0.000437384 | 0.002079319 | -0.2973309 |
| SGK3 | 0.764956193 | 8.53332353 | 4.21049659 | 0.000439031 | 0.002086019 | -0.30102164 |
| ZNF800 | 1.28722412 | 7.850467704 | 4.210056954 | 0.00043948 | 0.002087589 | -0.30202586 |
| TRIM3 | -2.502746216 | 6.910881435 | -4.209877744 | 0.000439663 | 0.002087895 | -0.30243522 |
| LAMB1 | -2.912791004 | 7.57490718 | -4.209459625 | 0.000440091 | 0.002088984 | -0.30339028 |
| DGCR14 | -0.654130869 | 7.447469453 | -4.209421273 | 0.00044013 | 0.002088984 | -0.30347789 |
| FXR1 | 1.221898659 | 9.948404135 | 4.209131096 | 0.000440427 | 0.002089831 | -0.30414071 |
| TMTC2 | 1.100235444 | 6.71863437 | 4.208825082 | 0.000440741 | 0.002090754 | -0.3048397 |
| PRMT7 | -0.518243715 | 10.54972501 | -4.208565589 | 0.000441007 | 0.002091452 | -0.30543243 |
| GEMIN2 | 0.972689506 | 5.531864833 | 4.208314066 | 0.000441265 | 0.002092112 | -0.30600695 |
| TBC1D7 | -0.6845893 | 9.043131263 | -4.207453874 | 0.000442149 | 0.002095736 | -0.30797178 |
| EPG5 | 0.872186079 | 6.761333135 | 4.207265324 | 0.000442342 | 0.002096091 | -0.30840246 |
| NCAPH | -3.051740746 | 6.03307509 | -4.205674124 | 0.000443982 | 0.002103295 | -0.31203698 |
| MAD2L2 | -0.518336376 | 8.418780274 | -4.205108821 | 0.000444567 | 0.002105495 | -0.3133282 |
| IER5L | -1.003927796 | 9.978774056 | -4.204542734 | 0.000445152 | 0.002107701 | -0.3146212 |
| ECHDC1 | 0.989980435 | 8.830285551 | 4.204190592 | 0.000445517 | 0.00210886 | -0.31542553 |
| CDC40 | 0.603720229 | 10.08856832 | 4.203686546 | 0.00044604 | 0.002110655 | -0.31657681 |
| UQCRB | 3.001881171 | 9.119888439 | 4.202135007 | 0.000447652 | 0.002117257 | -0.32012064 |
| RGS18 | 1.022566073 | 12.3688502 | 4.20184002 | 0.000447959 | 0.00211814 | -0.3207944 |
| TMEM165 | 0.785164189 | 6.314754567 | 4.200024475 | 0.000449855 | 0.002126532 | -0.32494116 |
| GATC | 1.131584145 | 5.898896457 | 4.199298487 | 0.000450615 | 0.002129553 | -0.32659932 |
| OR8G5 | -1.604718226 | 3.489621475 | -4.198932599 | 0.000450999 | 0.002130793 | -0.32743501 |
| EEF1E1 | 1.639825775 | 7.797193533 | 4.198035941 | 0.00045194 | 0.002134668 | -0.32948296 |
| DDI2 | 1.655662186 | 4.810972976 | 4.196941622 | 0.000453092 | 0.002139476 | -0.33198233 |
| ZNF90 | 1.197308165 | 7.491672872 | 4.196837767 | 0.000453201 | 0.002139476 | -0.33221953 |
| FRAT1 | -0.889734997 | 9.496693055 | -4.196668696 | 0.00045338 | 0.002139743 | -0.33260568 |
| TRDMT1 | 1.035348652 | 6.474857994 | 4.19599131 | 0.000454095 | 0.002142542 | -0.33415279 |
| TEAD1 | -1.972760181 | 6.371704702 | -4.195704519 | 0.000454398 | 0.002143397 | -0.3348078 |
| ITGA9 | 3.284248005 | 7.786851649 | 4.19554341 | 0.000454568 | 0.002143625 | -0.33517576 |
| SIGLEC9 | -0.90949195 | 9.113590774 | -4.195343365 | 0.000454779 | 0.002144047 | -0.33563264 |
| HMGN1 | 0.674217565 | 11.84209154 | 4.194321078 | 0.000455862 | 0.002148009 | -0.33796745 |
| FAM72D | 1.000430409 | 5.035868752 | 4.19431923 | 0.000455864 | 0.002148009 | -0.33797167 |
| WBSCR17 | -2.328629037 | 5.124520418 | -4.193819487 | 0.000456394 | 0.002149931 | -0.33911302 |
| TUBGCP3 | 0.680383122 | 8.305640855 | 4.193594723 | 0.000456633 | 0.002150479 | -0.33962636 |
| SERPINA9 | -1.784247745 | 4.478377376 | -4.193044196 | 0.000457218 | 0.002152658 | -0.34088369 |
| DDX26B | 0.777271923 | 8.443908602 | 4.192794775 | 0.000457483 | 0.002153331 | -0.34145333 |
| PSMD14 | 0.772942863 | 9.222893531 | 4.191875084 | 0.000458463 | 0.002157365 | -0.34355377 |
| STARD5 | -0.956014133 | 7.906894972 | -4.19064997 | 0.000459771 | 0.002162942 | -0.34635172 |
| OST4 | -0.59325886 | 14.39344676 | -4.190195297 | 0.000460258 | 0.002164652 | -0.3473901 |
| PRDX5 | -0.765367041 | 13.73735979 | -4.189544635 | 0.000460955 | 0.002166771 | -0.34887608 |
| MFSD6 | 0.695295746 | 9.007349153 | 4.18866551 | 0.000461898 | 0.002169615 | -0.35088381 |
| SDR42E1 | 1.198764558 | 5.797539071 | 4.188653518 | 0.000461911 | 0.002169615 | -0.35091119 |
| GTPBP4 | 0.637844985 | 10.02422994 | 4.188612991 | 0.000461955 | 0.002169615 | -0.35100375 |
| OSBPL5 | -0.891278643 | 10.3150866 | -4.188521405 | 0.000462053 | 0.002169615 | -0.35121291 |
| FOXO1 | 0.820119394 | 9.993382889 | 4.187941125 | 0.000462677 | 0.002171965 | -0.35253813 |
| NFIA | -0.979785452 | 6.807922907 | -4.186496636 | 0.000464235 | 0.002178693 | -0.35583696 |
| TROVE2 | 0.555036085 | 10.78380213 | 4.186118969 | 0.000464643 | 0.002180026 | -0.35669945 |
| TMEM102 | -1.095398114 | 8.550004241 | -4.183562203 | 0.000467414 | 0.002192445 | -0.36253829 |
| ATG7 | -0.565323891 | 8.913889002 | -4.181479031 | 0.000469684 | 0.002202507 | -0.36729549 |
| PERP | 1.774391307 | 7.152981776 | 4.180805352 | 0.000470421 | 0.002205373 | -0.3688339 |
| HIST1H4B | 1.585754372 | 10.42853587 | 4.179805037 | 0.000471517 | 0.002209921 | -0.3711182 |
| EGF | -1.326050002 | 4.692803454 | -4.179341575 | 0.000472026 | 0.002211716 | -0.37217655 |
| GPR142 | -2.397197674 | 7.658189259 | -4.178962266 | 0.000472442 | 0.002212742 | -0.37304272 |
| HNRNPA1L2 | 0.679062705 | 13.14408661 | 4.178913106 | 0.000472496 | 0.002212742 | -0.37315498 |
| VAC14 | -0.707466339 | 7.971351918 | -4.178158273 | 0.000473327 | 0.00221604 | -0.37487867 |
| AKT1 | -1.38852225 | 11.0734572 | -4.176282257 | 0.000475397 | 0.00222514 | -0.37916257 |
| HIST1H2BK | -0.847840233 | 11.51013556 | -4.17609513 | 0.000475604 | 0.002225516 | -0.37958987 |
| THOC5 | -0.666942401 | 8.476259257 | -4.174335536 | 0.000477554 | 0.00223405 | -0.38360783 |
| SESN2 | -0.821441018 | 7.077384078 | -4.173817347 | 0.00047813 | 0.002236149 | -0.38479108 |
| DNAJB6 | 0.700449113 | 10.70692164 | 4.172798521 | 0.000479265 | 0.002240859 | -0.38711748 |
| ACTR6 | 1.355002828 | 8.549679064 | 4.172237483 | 0.000479891 | 0.002243189 | -0.38839855 |
| FNBP4 | 0.690054486 | 10.12217386 | 4.172087513 | 0.000480058 | 0.002243376 | -0.38874099 |
| CYFIP2 | 0.728669864 | 10.54024402 | 4.171950009 | 0.000480212 | 0.002243497 | -0.38905497 |
| LRRC8C | 1.194327411 | 7.620222916 | 4.171733209 | 0.000480454 | 0.002244033 | -0.38955 |
| ACY3 | -3.034965608 | 7.027775736 | -4.170132868 | 0.000482246 | 0.002251804 | -0.39320415 |
| MYO7A | -0.895947239 | 6.061980138 | -4.169686066 | 0.000482747 | 0.002253547 | -0.39422435 |
| FEV | -1.443648256 | 4.276102232 | -4.169065723 | 0.000483445 | 0.002255604 | -0.39564079 |
| PGM2L1 | 0.794858033 | 7.772610881 | 4.168751223 | 0.000483798 | 0.002256656 | -0.39635889 |
| DCP1B | -0.615112046 | 10.46756861 | -4.168222503 | 0.000484394 | 0.002258834 | -0.39756611 |
| HN1 | -0.753030249 | 14.49129406 | -4.167802171 | 0.000484868 | 0.002260445 | -0.39852585 |
| EBAG9 | 0.658352138 | 8.706138258 | 4.167522834 | 0.000485183 | 0.002261315 | -0.39916366 |
| CNOT1 | 1.754955743 | 6.743822964 | 4.167396316 | 0.000485326 | 0.002261381 | -0.39945253 |
| SLIT3 | -1.423493604 | 6.430275195 | -4.16714953 | 0.000485604 | 0.00226208 | -0.40001601 |
| FXYD3 | -0.736961486 | 4.647488881 | -4.16693609 | 0.000485846 | 0.002262604 | -0.40050335 |
| SPTY2D1 | 1.172302979 | 5.947352001 | 4.166315503 | 0.000486547 | 0.002265273 | -0.4019203 |
| WDR48 | -0.732626554 | 11.16261188 | -4.165725509 | 0.000487216 | 0.002267784 | -0.4032674 |
| MTRNR2L10 | -1.346839203 | 6.624353624 | -4.165366439 | 0.000487623 | 0.002269078 | -0.40408723 |
| C11orf54 | 0.706509181 | 8.641123143 | 4.164858185 | 0.0004882 | 0.002271162 | -0.40524768 |
| SWAP70 | 1.084554302 | 9.746813644 | 4.164033237 | 0.000489137 | 0.002274923 | -0.4071312 |
| ATP2A2 | 1.112620243 | 6.794100544 | 4.163891205 | 0.000489299 | 0.002275073 | -0.40745548 |
| LMBR1L | -0.960566858 | 9.960924099 | -4.162992946 | 0.000490323 | 0.002279116 | -0.40950636 |
| API5 | 1.00889043 | 8.012516244 | 4.162900587 | 0.000490428 | 0.002279116 | -0.40971723 |
| CEBPA | -0.832485167 | 11.60885386 | -4.162391565 | 0.000491009 | 0.002281214 | -0.4108794 |
| MOG | -1.724767391 | 7.641399442 | -4.162086166 | 0.000491358 | 0.002282232 | -0.41157667 |
| CTBP2 | -0.597614186 | 12.12617789 | -4.161778585 | 0.00049171 | 0.002282902 | -0.41227892 |
| SETDB1 | -0.543495034 | 10.37957079 | -4.161732983 | 0.000491762 | 0.002282902 | -0.41238303 |
| TMEM206 | 1.223937411 | 6.935847623 | 4.161182786 | 0.000492392 | 0.002285223 | -0.4136392 |
| CLTB | -0.611622283 | 6.795552372 | -4.160974655 | 0.00049263 | 0.002285726 | -0.41411438 |
| NEB | 1.048936502 | 4.252022341 | 4.160096966 | 0.000493637 | 0.002289229 | -0.41611823 |
| KANK4 | -1.294319736 | 3.859467637 | -4.16008974 | 0.000493645 | 0.002289229 | -0.41613473 |
| METTL21A | 1.339157551 | 10.36663096 | 4.159594071 | 0.000494215 | 0.002291266 | -0.41726638 |
| ARL4C | 0.753625872 | 12.74761014 | 4.158979815 | 0.000494922 | 0.002293937 | -0.41866876 |
| RBCK1 | -0.649330224 | 10.10242391 | -4.157278523 | 0.000496884 | 0.002301919 | -0.42255286 |
| PPP2R1A | -1.146515377 | 10.95725888 | -4.157260254 | 0.000496906 | 0.002301919 | -0.42259457 |
| SOAT2 | -0.917759498 | 6.581996975 | -4.156654887 | 0.000497606 | 0.002304556 | -0.42397662 |
| FGF5 | 0.916051598 | 2.75783316 | 4.156388771 | 0.000497914 | 0.002305376 | -0.42458416 |
| CCT2 | 0.901989287 | 12.06108379 | 4.155410947 | 0.000499048 | 0.002310018 | -0.4268165 |
| KPTN | -1.129168047 | 7.811226276 | -4.155264505 | 0.000499218 | 0.002310197 | -0.42715083 |
| SLC22A18AS | -1.294894475 | 6.574873993 | -4.155113364 | 0.000499394 | 0.002310401 | -0.42749587 |
| ZNF649 | 1.285983217 | 4.666315077 | 4.152371371 | 0.000502589 | 0.002323964 | -0.43375561 |
| PGAM2 | -0.96203632 | 7.160738085 | -4.151488885 | 0.000503622 | 0.002328128 | -0.4357702 |
| AMDHD2 | -0.671054083 | 9.457145523 | -4.151295712 | 0.000503849 | 0.002328562 | -0.43621119 |
| HCFC1R1 | -0.558442893 | 11.23421726 | -4.150122794 | 0.000505225 | 0.002334311 | -0.43888876 |
| ASPRV1 | -1.303129788 | 11.30946599 | -4.149957311 | 0.00050542 | 0.002334597 | -0.43926653 |
| PARVG | -0.591698643 | 9.989548778 | -4.1496366 | 0.000505797 | 0.002335726 | -0.43999865 |
| NOTCH1 | -0.739731378 | 7.744158614 | -4.149372342 | 0.000506108 | 0.002336549 | -0.44060189 |
| C2orf82 | -1.12425077 | 7.539082617 | -4.149127252 | 0.000506397 | 0.002337268 | -0.44116138 |
| ZNF140 | 1.312831954 | 2.645631545 | 4.148673693 | 0.000506932 | 0.002339121 | -0.44219676 |
| GTPBP1 | -0.924711592 | 9.332194825 | -4.148541534 | 0.000507087 | 0.002339227 | -0.44249845 |
| TNXB | -1.627804402 | 9.01708297 | -4.148045811 | 0.000507673 | 0.002341312 | -0.44363007 |
| SNURF | 1.03907331 | 10.01677457 | 4.147859807 | 0.000507892 | 0.002341711 | -0.44405467 |
| FANCD2 | 0.840375702 | 5.409436901 | 4.147355215 | 0.000508489 | 0.002343847 | -0.44520652 |
| ST18 | 0.667627991 | 3.997106423 | 4.146493154 | 0.00050951 | 0.002347937 | -0.44717437 |
| RPS15A | 1.318666155 | 14.87776054 | 4.145962559 | 0.000510139 | 0.002350221 | -0.44838556 |
| FAHD2A | -0.585973933 | 8.792742087 | -4.145650202 | 0.00051051 | 0.002351314 | -0.44909858 |
| CSNK1A1L | 1.71490027 | 4.587736135 | 4.144921841 | 0.000511376 | 0.002354685 | -0.4507612 |
| OR2AG1 | -0.939073583 | 5.600756867 | -4.144691488 | 0.00051165 | 0.00235533 | -0.45128701 |
| METTL7A | 0.908165434 | 12.18017792 | 4.14311589 | 0.000513529 | 0.002362724 | -0.45488354 |
| HMGCR | 0.805409542 | 9.596551917 | 4.14304192 | 0.000513617 | 0.002362724 | -0.45505238 |
| TSPAN18 | -1.091705769 | 8.324862219 | -4.143006881 | 0.000513659 | 0.002362724 | -0.45513236 |
| IFI27 | -3.010320647 | 8.724766468 | -4.142496721 | 0.000514269 | 0.002364912 | -0.45629686 |
| RING1 | -0.66804811 | 10.54502986 | -4.142068478 | 0.000514782 | 0.00236665 | -0.45727436 |
| CALD1 | -1.513495621 | 5.645726138 | -4.140908152 | 0.000516173 | 0.002372427 | -0.45992289 |
| DAAM2 | -1.201333642 | 5.886190891 | -4.14070554 | 0.000516417 | 0.002372926 | -0.46038537 |
| RLN2 | 1.179180432 | 5.562455395 | 4.140234136 | 0.000516983 | 0.002374909 | -0.46146137 |
| OR6Y1 | -2.214421922 | 4.937589705 | -4.139978968 | 0.00051729 | 0.002375699 | -0.46204379 |
| ATMIN | -0.918515589 | 8.256025104 | -4.139237837 | 0.000518183 | 0.002379177 | -0.46373544 |
| DGKK | 1.307536275 | 3.367900399 | 4.138340953 | 0.000519265 | 0.002383525 | -0.46578257 |
| HSD3B7 | -1.182518325 | 7.717049564 | -4.137246025 | 0.00052059 | 0.002388981 | -0.46828171 |
| COL16A1 | -0.71318837 | 6.511975737 | -4.137128184 | 0.000520733 | 0.002389012 | -0.46855067 |
| NKIRAS1 | 1.308545157 | 4.249869275 | 4.134739524 | 0.000523635 | 0.002401699 | -0.47400257 |
| KRTAP12-1 | -2.228495698 | 5.127826428 | -4.134603828 | 0.0005238 | 0.002401831 | -0.47431228 |
| SLC16A3 | -0.710792197 | 15.25622399 | -4.134036241 | 0.000524492 | 0.002404378 | -0.47560772 |
| MACC1 | 1.014312706 | 2.540309903 | 4.133161009 | 0.000525561 | 0.002408651 | -0.4776053 |
| PNPT1 | 0.8794355 | 6.959951826 | 4.13271349 | 0.000526109 | 0.002410532 | -0.47862668 |
| AURKAIP1 | -0.754084951 | 12.44257423 | -4.132572056 | 0.000526282 | 0.002410697 | -0.47894947 |
| OGFOD1 | 1.001776157 | 8.247556948 | 4.132023129 | 0.000526954 | 0.00241315 | -0.48020229 |
| OR11A1 | -1.568138086 | 7.170292053 | -4.131163006 | 0.00052801 | 0.002417354 | -0.48216533 |
| PTEN | 0.788112615 | 9.815940265 | 4.129050096 | 0.000530612 | 0.002428635 | -0.48698749 |
| STYXL1 | -0.9201931 | 9.218846905 | -4.128456556 | 0.000531345 | 0.002431358 | -0.48834206 |
| SLC27A1 | -0.741324885 | 10.7056909 | -4.127948947 | 0.000531973 | 0.002432989 | -0.48950052 |
| PPP1R12B | -0.617024207 | 9.526697056 | -4.127870975 | 0.00053207 | 0.002432989 | -0.48967846 |
| METAP2 | 0.922427938 | 10.46366653 | 4.127832892 | 0.000532117 | 0.002432989 | -0.48976537 |
| EXOSC4 | -0.622022015 | 10.39136113 | -4.127496556 | 0.000532534 | 0.002434261 | -0.49053295 |
| KLHL32 | 0.90030534 | 3.702791716 | 4.126692436 | 0.000533531 | 0.002438067 | -0.49236806 |
| SLC17A4 | -2.659655791 | 5.168774036 | -4.12660166 | 0.000533643 | 0.002438067 | -0.49257523 |
| PRKRIR | 0.924085045 | 8.111507399 | 4.124603726 | 0.00053613 | 0.002448791 | -0.49713471 |
| DCTN6 | 1.076221785 | 8.838864053 | 4.122320589 | 0.000538985 | 0.002461194 | -0.50234489 |
| IL1RL1 | 1.578265769 | 3.030396853 | 4.120927732 | 0.000540735 | 0.002468542 | -0.50552335 |
| TBCK | 0.784394375 | 7.889487103 | 4.120137847 | 0.00054173 | 0.002472441 | -0.50732582 |
| CD1C | 1.591482679 | 5.119434538 | 4.119417359 | 0.000542639 | 0.002475835 | -0.50896991 |
| SPATS2 | 0.602136124 | 6.7528414 | 4.119325263 | 0.000542755 | 0.002475835 | -0.50918007 |
| RAN | 0.790862102 | 12.42971985 | 4.118821274 | 0.000543392 | 0.002478097 | -0.51033011 |
| MTX1 | -0.924089541 | 10.63168082 | -4.117547745 | 0.000545004 | 0.002484807 | -0.51323613 |
| SEMA3B | -0.723407195 | 7.981030018 | -4.115089619 | 0.00054813 | 0.002498411 | -0.51884509 |
| C2orf47 | 0.50838317 | 9.577094954 | 4.114930074 | 0.000548334 | 0.002498691 | -0.51920913 |
| TFF1 | -2.480183589 | 9.153232029 | -4.114629595 | 0.000548717 | 0.002499791 | -0.51989475 |
| NBEAL2 | -1.106301779 | 9.917362986 | -4.112439209 | 0.000551521 | 0.002511913 | -0.52489258 |
| KRTAP1-3 | -0.929149572 | 10.33580575 | -4.110319294 | 0.000554248 | 0.00252368 | -0.52972946 |
| INA | -1.870856545 | 6.211449065 | -4.10917173 | 0.00055573 | 0.002529772 | -0.53234773 |
| DPT | -2.626603676 | 7.608936638 | -4.108807484 | 0.000556201 | 0.002531262 | -0.53317878 |
| BMP6 | -1.202447131 | 7.728338534 | -4.108519554 | 0.000556574 | 0.002532015 | -0.53383571 |
| GYPE | -1.047255186 | 6.331435767 | -4.10843837 | 0.000556679 | 0.002532015 | -0.53402093 |
| TANC1 | 1.521589213 | 2.802408474 | 4.108346082 | 0.000556798 | 0.002532015 | -0.53423149 |
| FNDC9 | -1.5939826 | 6.995022689 | -4.108031241 | 0.000557207 | 0.002533215 | -0.53494981 |
| PCSK4 | -0.942033502 | 7.70794275 | -4.106908829 | 0.000558664 | 0.002539183 | -0.5375106 |
| UBE2J2 | -0.652147461 | 10.57227871 | -4.105885152 | 0.000559996 | 0.002544581 | -0.5398461 |
| SLFN12 | 0.801908393 | 7.10097983 | 4.105701135 | 0.000560236 | 0.002545013 | -0.54026592 |
| ITLN2 | -1.464743641 | 4.920627213 | -4.102665076 | 0.000564207 | 0.002562393 | -0.54719238 |
| RSPO3 | 2.475942386 | 5.192977659 | 4.102549491 | 0.000564359 | 0.00256242 | -0.54745607 |
| TRMT6 | 0.655832991 | 8.651419467 | 4.102037319 | 0.000565032 | 0.002564814 | -0.54862451 |
| ROM1 | -0.619298337 | 8.038494339 | -4.100396296 | 0.000567194 | 0.002573961 | -0.55236817 |
| RNF121 | -0.728279991 | 7.507722193 | -4.100265836 | 0.000567366 | 0.002574078 | -0.55266578 |
| UFSP1 | -0.753101758 | 7.252039578 | -4.099592754 | 0.000568255 | 0.002577414 | -0.55420125 |
| PRRG3 | -1.463462961 | 5.134043135 | -4.099487556 | 0.000568394 | 0.002577414 | -0.55444123 |
| PRM1 | -1.40091617 | 5.949472106 | -4.099226618 | 0.000568739 | 0.002578315 | -0.55503649 |
| COX16 | 1.541888706 | 9.494891194 | 4.098981308 | 0.000569064 | 0.002579122 | -0.5555961 |
| MYO5B | -1.586346533 | 4.776625719 | -4.09869056 | 0.000569449 | 0.002580202 | -0.55625935 |
| BCAT2 | -0.664334561 | 9.793815556 | -4.098175928 | 0.000570132 | 0.002582628 | -0.55743333 |
| NFATC2 | 1.824257155 | 6.700687974 | 4.097991709 | 0.000570376 | 0.00258307 | -0.55785357 |
| HSPBP1 | -0.619335759 | 10.74248947 | -4.096073873 | 0.000572927 | 0.00259357 | -0.56222844 |
| LSM6 | 0.504202081 | 10.3638504 | 4.096027023 | 0.000572989 | 0.00259357 | -0.56233531 |
| PDE4A | -0.692395994 | 9.269715403 | -4.095860053 | 0.000573212 | 0.00259391 | -0.56271619 |
| EXD2 | 1.181067248 | 3.871521911 | 4.095512874 | 0.000573675 | 0.002595338 | -0.56350814 |
| PMEL | -2.462634847 | 5.475985377 | -4.094260576 | 0.000575349 | 0.002602242 | -0.56636473 |
| DENND4C | 0.510482759 | 9.013957514 | 4.093727317 | 0.000576064 | 0.002604803 | -0.56758111 |
| GSTA2 | -1.417978577 | 4.919452976 | -4.092134402 | 0.000578203 | 0.002613803 | -0.57121456 |
| DEFB124 | -1.697467286 | 6.570169719 | -4.091715534 | 0.000578767 | 0.00261568 | -0.57216998 |
| CLIC4 | 0.866718013 | 7.539041362 | 4.091312691 | 0.000579309 | 0.00261746 | -0.57308885 |
| CIDEB | -0.927203675 | 9.462599526 | -4.090937864 | 0.000579815 | 0.002619071 | -0.57394381 |
| ASPA | -2.419488713 | 3.6304294 | -4.09043718 | 0.000580491 | 0.002621451 | -0.57508583 |
| COPS2 | 1.341889505 | 9.504273368 | 4.09022114 | 0.000580783 | 0.00262173 | -0.5755786 |
| CDKN1C | -1.160681911 | 12.08402742 | -4.090170716 | 0.000580851 | 0.00262173 | -0.57569361 |
| PPP4R2 | 1.309458826 | 7.398014931 | 4.089544001 | 0.000581698 | 0.002624456 | -0.57712308 |
| SLC41A3 | -0.85015734 | 10.4441078 | -4.089503483 | 0.000581753 | 0.002624456 | -0.57721549 |
| TIAL1 | 0.659724609 | 9.091817821 | 4.089271519 | 0.000582067 | 0.002624619 | -0.57774458 |
| MID2 | 0.912089274 | 6.570598512 | 4.089256282 | 0.000582088 | 0.002624619 | -0.57777933 |
| IRGM | 1.280907681 | 4.955815536 | 4.088359375 | 0.000583304 | 0.002629428 | -0.57982504 |
| PDXK | -0.711508628 | 9.818251737 | -4.087234554 | 0.000584833 | 0.002635343 | -0.58239056 |
| CARD9 | -0.956313811 | 9.406948262 | -4.087173507 | 0.000584916 | 0.002635343 | -0.58252979 |
| PMFBP1 | -0.762640006 | 5.259234944 | -4.086983523 | 0.000585174 | 0.002635833 | -0.58296311 |
| TMEM63B | -1.191737513 | 8.258417769 | -4.085747272 | 0.00058686 | 0.002642749 | -0.5857827 |
| ANKRD17 | 0.869514074 | 7.695565564 | 4.085148107 | 0.000587679 | 0.002645227 | -0.58714924 |
| SYNE1 | -1.779988995 | 11.05434031 | -4.085124477 | 0.000587711 | 0.002645227 | -0.58720313 |
| NARFL | -0.7448261 | 9.115207655 | -4.083706198 | 0.000589654 | 0.002653242 | -0.59043779 |
| CCDC6 | 0.593238505 | 10.42354859 | 4.083604245 | 0.000589794 | 0.002653242 | -0.59067031 |
| GLOD4 | 0.686832524 | 9.357147882 | 4.082983483 | 0.000590646 | 0.002656398 | -0.59208605 |
| CFH | 1.153016208 | 6.891772503 | 4.082128685 | 0.000591822 | 0.002661006 | -0.59403553 |
| HBA2 | -0.613484439 | 18.34190733 | -4.081973988 | 0.000592035 | 0.002661283 | -0.59438833 |
| CNOT4 | 0.568801132 | 7.380777825 | 4.080562218 | 0.000593983 | 0.002669358 | -0.59760798 |
| MUM1 | -0.777402138 | 9.7651784 | -4.078237113 | 0.000597206 | 0.002683153 | -0.60291039 |
| CNPY1 | -1.291693852 | 4.87101758 | -4.077021528 | 0.000598897 | 0.002690065 | -0.60568246 |
| ZNF555 | 0.873366049 | 7.553296948 | 4.076170514 | 0.000600084 | 0.002694021 | -0.60762311 |
| EIF4G1 | -2.085976891 | 9.856518385 | -4.075888521 | 0.000600478 | 0.002695058 | -0.60826616 |
| RCN3 | -1.250557473 | 7.921486641 | -4.075143305 | 0.00060152 | 0.002698402 | -0.60996552 |
| AOAH | -0.768830267 | 10.40290018 | -4.073509982 | 0.000603811 | 0.002707895 | -0.61369001 |
| BROX | 1.526688216 | 6.889417712 | 4.073414735 | 0.000603945 | 0.002707895 | -0.6139072 |
| NAV3 | -1.832091748 | 6.184584773 | -4.073000943 | 0.000604526 | 0.002709813 | -0.61485075 |
| ETHE1 | -0.633341619 | 10.71354502 | -4.071143481 | 0.000607145 | 0.002720856 | -0.61908618 |
| PFN4 | 1.606113483 | 3.764382047 | 4.070461952 | 0.000608108 | 0.00272448 | -0.62064019 |
| REG1B | -1.912335521 | 4.942797568 | -4.069345684 | 0.00060969 | 0.00273087 | -0.62318543 |
| GH2 | 2.217249796 | 4.492410386 | 4.068734607 | 0.000610557 | 0.00273406 | -0.62457875 |
| FAM134B | 1.172719365 | 8.500255622 | 4.066776582 | 0.000613346 | 0.002745845 | -0.62904316 |
| GGT7 | -1.209863476 | 6.840587352 | -4.063401482 | 0.000618181 | 0.00276679 | -0.63673821 |
| LIMK1 | -0.671842366 | 8.750907585 | -4.06199591 | 0.000620206 | 0.002775036 | -0.63994271 |
| ZFPM1 | -0.696779584 | 9.781115463 | -4.061903829 | 0.000620339 | 0.002775036 | -0.64015263 |
| KCTD2 | -1.198685476 | 9.400536804 | -4.06172025 | 0.000620604 | 0.002775516 | -0.64057116 |
| MPST | -0.896118571 | 11.67058276 | -4.061362993 | 0.00062112 | 0.002777118 | -0.64138564 |
| GRIK2 | -0.94514773 | 3.513038125 | -4.061251913 | 0.000621281 | 0.002777129 | -0.64163888 |
| EXOSC6 | 1.162171493 | 7.861102704 | 4.060935269 | 0.000621739 | 0.00277847 | -0.64236076 |
| ZNF257 | 1.385740192 | 8.972999056 | 4.060011825 | 0.000623076 | 0.002783739 | -0.64446599 |
| PAGE2 | -1.523311078 | 8.166831934 | -4.058849624 | 0.000624764 | 0.002790568 | -0.64711548 |
| SFXN5 | -0.865467826 | 9.506448938 | -4.05871909 | 0.000624953 | 0.002790707 | -0.64741306 |
| MTMR4 | 0.516032446 | 11.2122867 | 4.057846471 | 0.000626224 | 0.00279567 | -0.64940234 |
| LRRC52 | -1.560483133 | 4.583466591 | -4.057230762 | 0.000627121 | 0.002798967 | -0.65080594 |
| STXBP4 | 1.334911423 | 6.517591432 | 4.056678063 | 0.000627928 | 0.002801858 | -0.65206588 |
| SAMD8 | 0.724634918 | 5.883042196 | 4.056141498 | 0.000628713 | 0.002804647 | -0.65328903 |
| NPPA | -2.437980495 | 7.779554507 | -4.055980333 | 0.000628949 | 0.002804987 | -0.65365642 |
| TFAM | 0.991008669 | 9.821576567 | 4.05583505 | 0.000629161 | 0.002805224 | -0.6539876 |
| PHLDB1 | -1.569157368 | 5.160338556 | -4.055148831 | 0.000630167 | 0.0028086 | -0.65555188 |
| NDUFB3 | 1.650061062 | 10.89699715 | 4.055100226 | 0.000630238 | 0.0028086 | -0.65566268 |
| MYCBP2 | 0.514854171 | 11.78565982 | 4.053891723 | 0.000632013 | 0.002815796 | -0.65841748 |
| IFRD2 | -0.704230334 | 10.06205518 | -4.052838949 | 0.000633563 | 0.002821783 | -0.66081724 |
| SYT8 | -1.102487236 | 5.659690408 | -4.052761185 | 0.000633678 | 0.002821783 | -0.6609945 |
| CTBS | 0.644132999 | 11.03680304 | 4.052492716 | 0.000634074 | 0.002822831 | -0.66160646 |
| ACTL7A | -2.708861031 | 6.255974442 | -4.051748408 | 0.000635173 | 0.002827009 | -0.66330305 |
| CRAT | -1.25485147 | 10.47552728 | -4.04851444 | 0.00063997 | 0.002847641 | -0.67067434 |
| CHST7 | -0.818926727 | 9.67951353 | -4.04787224 | 0.000640927 | 0.002851178 | -0.67213807 |
| FAM177B | -2.308655604 | 8.036512619 | -4.047175763 | 0.000641967 | 0.00285508 | -0.6737255 |
| OR10J3 | -1.981580618 | 4.843708376 | -4.045141335 | 0.000645013 | 0.002867902 | -0.67836229 |
| TNFSF8 | 2.058264815 | 6.316709188 | 4.045014168 | 0.000645204 | 0.002868026 | -0.67865212 |
| CCDC114 | -0.944057464 | 5.656015182 | -4.044268718 | 0.000646324 | 0.002872279 | -0.68035108 |
| SIRT6 | -0.925073376 | 8.758789628 | -4.043433209 | 0.000647582 | 0.00287612 | -0.68225525 |
| SLC16A7 | 1.449561395 | 6.114762684 | 4.043385615 | 0.000647653 | 0.00287612 | -0.68236372 |
| KLF13 | 0.551889661 | 13.78725329 | 4.043368583 | 0.000647679 | 0.00287612 | -0.68240254 |
| WDR38 | -1.715824982 | 5.355008457 | -4.043228197 | 0.000647891 | 0.002876334 | -0.68272248 |
| UPF3B | 1.207400001 | 6.642689924 | 4.042810845 | 0.00064852 | 0.002878402 | -0.68367364 |
| DAPK2 | -0.900977507 | 10.29229273 | -4.040747281 | 0.000651642 | 0.002891526 | -0.68837645 |
| OTOGL | -0.880880775 | 3.959943123 | -4.039953801 | 0.000652846 | 0.002896139 | -0.69018472 |
| CR1 | 1.493450085 | 6.889963323 | 4.039175916 | 0.000654029 | 0.002900654 | -0.69195743 |
| 3-Mar | -1.095034354 | 6.873832369 | -4.038351558 | 0.000655284 | 0.00290549 | -0.69383601 |
| NOP2 | -0.607664113 | 9.769596189 | -4.037885298 | 0.000655996 | 0.002907911 | -0.69489853 |
| RNPEPL1 | -0.668056685 | 15.88628566 | -4.037171524 | 0.000657086 | 0.00291201 | -0.69652507 |
| ACTN1 | -0.90908932 | 11.83690843 | -4.035748864 | 0.000659265 | 0.002920307 | -0.69976694 |
| KIAA0101 | 1.709940047 | 6.380812205 | 4.035732155 | 0.00065929 | 0.002920307 | -0.69980501 |
| PUS10 | 0.885094477 | 5.395101029 | 4.034229852 | 0.000661599 | 0.002929795 | -0.70322827 |
| FKBP4 | -0.568005455 | 8.408969482 | -4.032622868 | 0.000664077 | 0.00294003 | -0.70688995 |
| PCDH11X | -0.946685786 | 3.245950739 | -4.032068979 | 0.000664934 | 0.002943081 | -0.70815202 |
| IRG1 | -1.2986169 | 5.812827296 | -4.031603839 | 0.000665654 | 0.002945526 | -0.70921186 |
| FGFR4 | 1.377147225 | 6.437919246 | 4.030434989 | 0.000667467 | 0.002952805 | -0.71187508 |
| CCRL2 | -0.826626804 | 7.754921194 | -4.030217044 | 0.000667805 | 0.00295356 | -0.71237166 |
| EIF4B | 0.863936997 | 10.66042354 | 4.0300208 | 0.00066811 | 0.002954166 | -0.71281879 |
| FGL2 | 1.442615809 | 9.004775753 | 4.029559638 | 0.000668827 | 0.002956594 | -0.71386952 |
| CENPA | 1.069545712 | 2.677336875 | 4.028537243 | 0.00067042 | 0.002962891 | -0.71619896 |
| LYZL1 | 3.604206983 | 5.381953059 | 4.026147759 | 0.000674158 | 0.002978661 | -0.72164299 |
| SMYD5 | -0.679037211 | 8.818927173 | -4.025633905 | 0.000674965 | 0.002981476 | -0.72281368 |
| SPC25 | 1.696265884 | 3.8352544 | 4.025038355 | 0.000675901 | 0.002984454 | -0.72417049 |
| CDC42SE1 | -0.812846354 | 9.116170316 | -4.024988933 | 0.000675978 | 0.002984454 | -0.72428308 |
| CCL27 | -0.909051492 | 4.459251945 | -4.024656554 | 0.000676501 | 0.002986013 | -0.72504031 |
| STAB2 | -1.771839302 | 5.264698258 | -4.024236335 | 0.000677163 | 0.002988184 | -0.72599765 |
| PRRG1 | -1.446297418 | 4.929372142 | -4.021841474 | 0.000680947 | 0.003003823 | -0.73145346 |
| ALDH2 | -0.949837289 | 10.680359 | -4.021777285 | 0.000681049 | 0.003003823 | -0.73159969 |
| CYP2S1 | -1.038561685 | 6.370558262 | -4.021087243 | 0.000682143 | 0.003007895 | -0.73317164 |
| NES | -2.348064764 | 7.838158921 | -4.020330246 | 0.000683346 | 0.003012442 | -0.7348961 |
| REPS2 | -0.795915356 | 9.175373931 | -4.020078085 | 0.000683747 | 0.003013455 | -0.73547052 |
| MANBAL | -0.675177868 | 9.314935962 | -4.019245326 | 0.000685073 | 0.003018543 | -0.73736752 |
| LRRC26 | -1.164985332 | 9.812851966 | -4.017791507 | 0.000687394 | 0.003028012 | -0.7406792 |
| TBC1D15 | 0.861823667 | 9.863546113 | 4.017518808 | 0.00068783 | 0.003028683 | -0.74130038 |
| OPN4 | -2.063739357 | 6.675235495 | -4.017480991 | 0.000687891 | 0.003028683 | -0.74138652 |
| SLAMF1 | 0.743193675 | 9.304650493 | 4.015636078 | 0.00069085 | 0.00304095 | -0.74558891 |
| SRPK1 | 0.775938103 | 11.51867017 | 4.015326485 | 0.000691348 | 0.00304238 | -0.74629409 |
| ZBTB37 | 1.362171306 | 6.914912453 | 4.013990902 | 0.000693499 | 0.003051085 | -0.7493362 |
| CYP2B6 | -1.675184867 | 6.607842029 | -4.01349633 | 0.000694298 | 0.003053834 | -0.75046268 |
| DHX34 | -0.727549608 | 10.69620365 | -4.013229773 | 0.000694729 | 0.003054965 | -0.75106981 |
| MAP4K2 | -0.90892076 | 8.729072395 | -4.012331876 | 0.000696182 | 0.003060261 | -0.7531149 |
| CACNA1I | -0.933491261 | 7.46517838 | -4.012270351 | 0.000696281 | 0.003060261 | -0.75325504 |
| CBY1 | -0.676404735 | 5.865873498 | -4.009622683 | 0.000700584 | 0.003078402 | -0.75928525 |
| ZNF331 | 0.732129334 | 6.387634481 | 4.009458621 | 0.000700851 | 0.003078808 | -0.7596589 |
| OR1S2 | -2.647189839 | 5.782074316 | -4.008997724 | 0.000701603 | 0.003081342 | -0.76070858 |
| LZTFL1 | 0.993399183 | 6.039766338 | 4.008434414 | 0.000702523 | 0.003084613 | -0.76199149 |
| ZNF558 | 0.885405925 | 8.512678266 | 4.00720448 | 0.000704537 | 0.00309268 | -0.76479255 |
| SCYL1 | -0.569324832 | 13.45994673 | -4.006255134 | 0.000706095 | 0.003098745 | -0.76695455 |
| MELK | 1.017140987 | 4.322337448 | 4.005461406 | 0.0007074 | 0.003103698 | -0.76876211 |
| FOXD4 | 0.761881012 | 4.905438712 | 4.004944974 | 0.00070825 | 0.003106655 | -0.76993817 |
| LCE3B | -1.006323716 | 4.752109906 | -4.003957048 | 0.00070988 | 0.003113027 | -0.77218791 |
| NAT1 | 0.94198056 | 7.778713186 | 4.00369362 | 0.000710315 | 0.003114159 | -0.7727878 |
| ZBTB7C | -1.373076462 | 6.380078934 | -4.003505351 | 0.000710626 | 0.003114747 | -0.77321652 |
| CCR3 | -1.616658982 | 10.36231273 | -4.003370706 | 0.000710849 | 0.003114947 | -0.77352313 |
| LCTL | 1.067530931 | 3.363460918 | 4.003208731 | 0.000711117 | 0.003115344 | -0.77389198 |
| NDUFS4 | 1.152453462 | 10.70472681 | 4.002951292 | 0.000711543 | 0.003116434 | -0.77447821 |
| RPL31 | 2.132526004 | 13.76736188 | 4.002348141 | 0.000712542 | 0.003120034 | -0.77585168 |
| IGFBP2 | -1.192168989 | 8.395133622 | -4.001890738 | 0.000713301 | 0.003122578 | -0.77689324 |
| MMP15 | -0.947092998 | 6.124275082 | -4.00062232 | 0.000715409 | 0.003131028 | -0.77978154 |
| RBBP6 | 1.419243617 | 6.817979594 | 4.000111784 | 0.000716259 | 0.003133969 | -0.78094405 |
| TIGD2 | 0.773174859 | 7.423097659 | 3.999646693 | 0.000717035 | 0.003136581 | -0.78200306 |
| MGP | -0.932060894 | 4.867991163 | -3.998805295 | 0.00071844 | 0.003141946 | -0.78391891 |
| ST3GAL6 | 0.812391511 | 7.819902681 | 3.998130478 | 0.000719569 | 0.0031461 | -0.78545543 |
| RWDD1 | 1.29685723 | 10.6513474 | 3.997658979 | 0.000720358 | 0.003148771 | -0.78652899 |
| UBE2F | -0.781934482 | 10.64381584 | -3.997071569 | 0.000721344 | 0.003151884 | -0.78786646 |
| PPP1R11 | -0.794256743 | 8.782456203 | -3.9970205 | 0.000721429 | 0.003151884 | -0.78798274 |
| GGNBP2 | 0.664012378 | 9.267301965 | 3.996154097 | 0.000722885 | 0.003157218 | -0.7899554 |
| TLR4 | 0.982520141 | 10.66945951 | 3.996080242 | 0.000723009 | 0.003157218 | -0.79012356 |
| DNPEP | -0.827805401 | 7.209869998 | -3.995031439 | 0.000724776 | 0.003164146 | -0.79251147 |
| C12orf10 | -0.83140985 | 11.05672189 | -3.994434343 | 0.000725783 | 0.003167758 | -0.79387091 |
| CYCS | 0.805056188 | 8.326008845 | 3.993772419 | 0.000726902 | 0.003171853 | -0.79537792 |
| ANKRD33B | 0.649911416 | 6.348108048 | 3.992904683 | 0.000728371 | 0.003177474 | -0.79735348 |
| AMELX | -1.43574122 | 3.670601341 | -3.992650516 | 0.000728802 | 0.003178565 | -0.79793213 |
| TNNI2 | -0.87044632 | 10.23811734 | -3.992244632 | 0.00072949 | 0.003180779 | -0.79885618 |
| ZEB2 | 1.063879993 | 9.553255855 | 3.992137956 | 0.000729671 | 0.00318078 | -0.79909905 |
| CTR9 | 0.690264956 | 9.733666519 | 3.991863412 | 0.000730137 | 0.003182024 | -0.79972408 |
| CPLX2 | -1.963899406 | 7.555512197 | -3.991631663 | 0.000730531 | 0.003182304 | -0.80025168 |
| SERTAD2 | 0.665376685 | 9.612873233 | 3.991565858 | 0.000730643 | 0.003182304 | -0.80040149 |
| OR5A1 | -1.635523719 | 6.363028832 | -3.991506114 | 0.000730745 | 0.003182304 | -0.8005375 |
| PITHD1 | -1.573891181 | 8.002216727 | -3.989772344 | 0.000733698 | 0.003194375 | -0.8044845 |
| TIAM2 | -1.145458171 | 8.782080845 | -3.989154176 | 0.000734754 | 0.00319818 | -0.80589175 |
| EIF2A | 0.912368961 | 8.60982531 | 3.988282786 | 0.000736245 | 0.003203878 | -0.80787542 |
| ZNF81 | 0.807454662 | 5.533909439 | 3.987460453 | 0.000737655 | 0.003209219 | -0.80974738 |
| PRADC1 | -0.756961495 | 8.586168912 | -3.986517695 | 0.000739275 | 0.00321547 | -0.81189343 |
| RSRC1 | 0.961290399 | 7.542297107 | 3.985868694 | 0.000740392 | 0.003219532 | -0.81337076 |
| RNASEL | 0.826771082 | 9.26244352 | 3.985557893 | 0.000740927 | 0.003221064 | -0.81407824 |
| APOPT1 | -0.544508918 | 10.00071843 | -3.985329622 | 0.000741321 | 0.003221979 | -0.81459784 |
| MRPL47 | 1.227628511 | 10.02329713 | 3.984466578 | 0.000742811 | 0.003226971 | -0.81656234 |
| METRNL | -0.587105304 | 12.71582363 | -3.984451709 | 0.000742836 | 0.003226971 | -0.81659619 |
| MST1 | -0.726784972 | 7.940315885 | -3.984034547 | 0.000743558 | 0.003229307 | -0.81754574 |
| AKIRIN1 | 0.679450198 | 10.20494789 | 3.982825842 | 0.000745652 | 0.003237601 | -0.82029695 |
| RPF1 | 0.82180813 | 9.116121328 | 3.979829528 | 0.000750867 | 0.003258639 | -0.82711674 |
| RARG | -1.086145583 | 8.803458464 | -3.978757871 | 0.000752742 | 0.003265967 | -0.82955577 |
| PRSS55 | -1.580117182 | 3.75474669 | -3.978454658 | 0.000753273 | 0.003267465 | -0.83024586 |
| ABO | -0.906275482 | 8.133000503 | -3.977554779 | 0.000754852 | 0.003273505 | -0.83229388 |
| IFT27 | -0.691907698 | 7.826161566 | -3.977404449 | 0.000755116 | 0.003273843 | -0.83263601 |
| SMN1 | 0.821346843 | 10.3461008 | 3.976095303 | 0.000757419 | 0.003283019 | -0.83561537 |
| ZNF623 | 0.919246276 | 4.99957904 | 3.975865758 | 0.000757823 | 0.00328376 | -0.83613777 |
| ZNF784 | -0.668250174 | 9.825355939 | -3.975619733 | 0.000758257 | 0.003284225 | -0.83669766 |
| ZNF549 | 0.971210368 | 5.800781973 | 3.974525375 | 0.00076019 | 0.003291786 | -0.83918812 |
| RIBC2 | -1.499473308 | 4.532976114 | -3.973934349 | 0.000761236 | 0.003295504 | -0.8405331 |
| NOXA1 | -0.95895696 | 9.311005587 | -3.973681011 | 0.000761685 | 0.003296635 | -0.84110961 |
| XRCC4 | 1.394618007 | 7.579594537 | 3.973098213 | 0.000762718 | 0.003300295 | -0.84243584 |
| KLRC3 | 2.126794412 | 6.79634382 | 3.972717055 | 0.000763395 | 0.00330241 | -0.84330321 |
| CARD14 | -1.299338794 | 8.002789928 | -3.972535107 | 0.000763718 | 0.003302996 | -0.84371725 |
| TCERG1 | 0.98670586 | 7.473367853 | 3.971610548 | 0.000765362 | 0.0033087 | -0.84582114 |
| ZNF823 | 1.17019916 | 5.844267283 | 3.971581943 | 0.000765413 | 0.0033087 | -0.84588623 |
| PHTF1 | 0.88731148 | 7.122877097 | 3.971066612 | 0.000766331 | 0.003311855 | -0.84705887 |
| N6AMT2 | -0.637051174 | 8.233563509 | -3.970922699 | 0.000766588 | 0.00331215 | -0.84738635 |
| IL3RA | -1.392662653 | 10.37817383 | -3.970008127 | 0.000768221 | 0.003316971 | -0.84946743 |
| PTPRF | -1.818894045 | 4.605495548 | -3.96991619 | 0.000768385 | 0.003316971 | -0.84967663 |
| CDIPT | -1.019375936 | 9.227554813 | -3.969839115 | 0.000768523 | 0.003316971 | -0.84985201 |
| MYBPH | -1.534460262 | 9.306892844 | -3.969769648 | 0.000768647 | 0.003316971 | -0.85001008 |
| CTPS2 | 0.66460532 | 5.596550776 | 3.969537515 | 0.000769062 | 0.003317949 | -0.85053828 |
| PCCB | -0.662000337 | 7.311793227 | -3.969388222 | 0.000769329 | 0.003318287 | -0.85087798 |
| WDR33 | 0.736010261 | 8.18737817 | 3.9691905 | 0.000769683 | 0.003319 | -0.85132788 |
| BCR | -0.620721657 | 9.098803576 | -3.968522233 | 0.000770881 | 0.003323349 | -0.85284844 |
| SNRPE | 1.589654706 | 8.829913753 | 3.967862519 | 0.000772065 | 0.003327637 | -0.85434952 |
| ALDH4A1 | -0.835002202 | 8.051992523 | -3.966647275 | 0.00077425 | 0.00333624 | -0.85711456 |
| TMCO6 | -0.716934666 | 10.14187899 | -3.965670787 | 0.000776011 | 0.003343008 | -0.8593363 |
| ZC3H12D | 0.999201599 | 5.36005881 | 3.964878005 | 0.000777444 | 0.003348358 | -0.86114003 |
| PEAR1 | -1.385033754 | 4.148086859 | -3.964268404 | 0.000778547 | 0.003352015 | -0.86252696 |
| AASS | 1.046021587 | 4.451409988 | 3.964198279 | 0.000778674 | 0.003352015 | -0.8626865 |
| MIER2 | -0.522038452 | 10.42381798 | -3.963975351 | 0.000779078 | 0.003352933 | -0.86319369 |
| TGFA | -1.01237135 | 9.55344945 | -3.963470919 | 0.000779993 | 0.003355795 | -0.86434132 |
| CLN5 | 0.701000013 | 8.072087247 | 3.963398188 | 0.000780125 | 0.003355795 | -0.86450679 |
| SLC25A25 | 0.597824891 | 9.348941793 | 3.962804647 | 0.000781202 | 0.003359572 | -0.86585714 |
| CD207 | -1.28497983 | 3.869466161 | -3.962704377 | 0.000781385 | 0.003359572 | -0.86608526 |
| LSM14B | 1.623109286 | 7.01649105 | 3.962548625 | 0.000781668 | 0.003359968 | -0.8664396 |
| PTPN23 | -0.61465629 | 9.414065737 | -3.96209837 | 0.000782487 | 0.003361951 | -0.86746394 |
| CSPG4 | -1.096216559 | 7.113258831 | -3.962084874 | 0.000782511 | 0.003361951 | -0.86749464 |
| LCN2 | -1.873578358 | 9.176507268 | -3.96192936 | 0.000782795 | 0.003362346 | -0.86784844 |
| ADAMTS2 | -1.690121111 | 5.053106913 | -3.960516675 | 0.000785371 | 0.003372589 | -0.87106224 |
| CENPE | 1.183303937 | 5.41361359 | 3.960019365 | 0.00078628 | 0.003375669 | -0.87219357 |
| NWD1 | 0.934516849 | 2.491264812 | 3.959241333 | 0.000787705 | 0.003380958 | -0.87396349 |
| CCT6B | 1.457694041 | 4.190303166 | 3.959123007 | 0.000787921 | 0.003381064 | -0.87423267 |
| OR1E1 | -1.904570803 | 4.791806434 | -3.958550689 | 0.000788971 | 0.003384742 | -0.87553459 |
| PLEKHM3 | 0.746745144 | 6.203564092 | 3.956962353 | 0.000791892 | 0.003395922 | -0.87914768 |
| KHDRBS1 | 0.631237039 | 12.4683412 | 3.956923317 | 0.000791963 | 0.003395922 | -0.87923648 |
| NCBP1 | 0.605401148 | 6.767057955 | 3.95659482 | 0.000792569 | 0.00339769 | -0.87998371 |
| ABHD13 | 1.155029474 | 2.620114233 | 3.955927464 | 0.0007938 | 0.003401916 | -0.88150173 |
| DBI | 1.509726862 | 12.26938443 | 3.955850815 | 0.000793942 | 0.003401916 | -0.88167608 |
| ZBTB8A | -1.853380467 | 4.504196664 | -3.955435497 | 0.000794709 | 0.003404351 | -0.88262078 |
| CTSB | -0.801800349 | 11.0058631 | -3.955272817 | 0.00079501 | 0.003404351 | -0.88299082 |
| SNAI3 | -1.131356669 | 9.429410541 | -3.95522904 | 0.000795091 | 0.003404351 | -0.88309039 |
| DAPK3 | -0.521080836 | 12.23276199 | -3.95493443 | 0.000795636 | 0.003405856 | -0.88376051 |
| CCT4 | 0.53983168 | 13.77688513 | 3.952359869 | 0.000800415 | 0.00342548 | -0.88961643 |
| CCND2 | 0.940691091 | 8.853110866 | 3.950516109 | 0.000803855 | 0.003437692 | -0.89380988 |
| NPEPPS | 0.755231675 | 9.027527147 | 3.950333842 | 0.000804196 | 0.003438314 | -0.89422442 |
| HDAC6 | -0.740477096 | 9.205544286 | -3.95015628 | 0.000804528 | 0.003438898 | -0.89462825 |
| C11orf49 | -0.509667089 | 8.220463266 | -3.949404337 | 0.000805936 | 0.003444081 | -0.8963384 |
| CNTROB | -0.630949779 | 8.536269559 | -3.949143713 | 0.000806425 | 0.003445332 | -0.89693113 |
| URB2 | 0.778026466 | 6.434787205 | 3.948968884 | 0.000806753 | 0.003445896 | -0.89732874 |
| ANKLE2 | 0.734193653 | 7.345642992 | 3.947450076 | 0.000809608 | 0.003457252 | -0.90078283 |
| DUOX2 | -1.5551182 | 4.467808481 | -3.946967486 | 0.000810518 | 0.003460294 | -0.90188032 |
| CHST13 | -0.83221099 | 9.759100586 | -3.946070518 | 0.00081221 | 0.003466679 | -0.90392013 |
| PNPLA4 | 1.428712797 | 4.777270292 | 3.945419724 | 0.000813441 | 0.003471089 | -0.90540008 |
| SH3YL1 | 0.96291091 | 8.654979762 | 3.945264514 | 0.000813734 | 0.0034715 | -0.90575303 |
| UBA3 | 1.162404716 | 8.151831154 | 3.944512107 | 0.00081516 | 0.003476737 | -0.90746402 |
| ATP9A | -1.138797818 | 7.365934171 | -3.944234747 | 0.000815686 | 0.003478137 | -0.90809474 |
| VNN3 | 1.523108789 | 6.969391121 | 3.943433199 | 0.000817208 | 0.003483783 | -0.90991742 |
| NPLOC4 | -0.519492123 | 11.85871421 | -3.943062408 | 0.000817913 | 0.003485943 | -0.91076057 |
| LANCL1 | 1.196489818 | 6.91104566 | 3.942944917 | 0.000818137 | 0.003486051 | -0.91102774 |
| PCDHB4 | -2.599187986 | 4.278174399 | -3.941109283 | 0.000821637 | 0.003499731 | -0.91520169 |
| MLH3 | -0.856702706 | 8.398251 | -3.941052751 | 0.000821745 | 0.003499731 | -0.91533024 |
| UBQLN3 | -1.289999915 | 4.770474658 | -3.940786113 | 0.000822255 | 0.003501054 | -0.91593651 |
| NKAIN1 | -2.073242832 | 6.270293834 | -3.940471429 | 0.000822857 | 0.00350277 | -0.91665203 |
| GYLTL1B | -1.231436322 | 8.44136669 | -3.939828823 | 0.000824088 | 0.00350716 | -0.91811315 |
| ISLR | -1.738603405 | 8.427073294 | -3.939691445 | 0.000824351 | 0.003507432 | -0.91842551 |
| DSTN | 0.769011578 | 10.93081749 | 3.938654798 | 0.000826341 | 0.00351373 | -0.92078251 |
| TGIF1 | 0.518439214 | 8.405785351 | 3.938621993 | 0.000826404 | 0.00351373 | -0.9208571 |
| MRVI1 | -1.114181883 | 11.45298235 | -3.938558409 | 0.000826526 | 0.00351373 | -0.92100167 |
| GDAP2 | 0.539841115 | 7.639840916 | 3.938504161 | 0.000826631 | 0.00351373 | -0.92112501 |
| ELK4 | 1.920916268 | 7.961725646 | 3.936267669 | 0.000830942 | 0.003531202 | -0.92620984 |
| ATG9A | -0.799091055 | 8.16420004 | -3.936151555 | 0.000831166 | 0.003531302 | -0.92647382 |
| EYA1 | -2.227932952 | 5.4510907 | -3.935684879 | 0.000832069 | 0.003534283 | -0.9275348 |
| WNT11 | -1.292145956 | 4.737133138 | -3.935462425 | 0.0008325 | 0.003535184 | -0.92804054 |
| WWC3 | -0.593793154 | 11.52616389 | -3.935367685 | 0.000832683 | 0.003535184 | -0.92825593 |
| SLC35D2 | -0.5217726 | 7.834638247 | -3.934969968 | 0.000833454 | 0.003537601 | -0.92916012 |
| ZNF75A | 0.73528761 | 9.591064082 | 3.934516162 | 0.000834334 | 0.003540482 | -0.93019181 |
| ATP6V1C1 | 0.560710566 | 10.5735793 | 3.934315734 | 0.000834723 | 0.003541279 | -0.93064746 |
| MRPL28 | -1.069870736 | 11.13649038 | -3.932942821 | 0.000837392 | 0.003551748 | -0.93376857 |
| SF3B2 | -0.664321927 | 11.51893703 | -3.932833917 | 0.000837605 | 0.003551791 | -0.93401614 |
| ARHGEF12 | -1.060961072 | 4.842091797 | -3.932687255 | 0.00083789 | 0.003552146 | -0.93434954 |
| KRTAP10-7 | -2.013994039 | 5.206350327 | -3.930510796 | 0.000842142 | 0.003568451 | -0.93929712 |
| OR1N2 | -1.618400544 | 4.806899445 | -3.930083697 | 0.000842979 | 0.003571137 | -0.94026798 |
| SUSD2 | -0.734987936 | 5.757249422 | -3.929601652 | 0.000843925 | 0.003574282 | -0.94136372 |
| GNPTAB | 0.581004088 | 9.346141181 | 3.928805581 | 0.000845489 | 0.003580043 | -0.94317325 |
| CHMP6 | -0.869224208 | 9.339416133 | -3.927819884 | 0.000847429 | 0.003587396 | -0.94541376 |
| SSC5D | -0.820264948 | 5.639378438 | -3.927115506 | 0.000848819 | 0.003592413 | -0.94701479 |
| MRPL13 | 1.371607313 | 9.486206351 | 3.926876047 | 0.000849291 | 0.003593549 | -0.94755907 |
| CD48 | 0.837798395 | 12.30994768 | 3.926652907 | 0.000849732 | 0.003594549 | -0.94806625 |
| PAWR | 1.513651708 | 6.574339894 | 3.925812538 | 0.000851395 | 0.003600715 | -0.94997632 |
| ADAM20 | 1.296333522 | 4.715338673 | 3.92503364 | 0.000852938 | 0.003606376 | -0.95174663 |
| ANAPC10 | 1.144393011 | 7.41777251 | 3.924607282 | 0.000853784 | 0.003609086 | -0.95271566 |
| TCEANC | -0.719985238 | 8.217030177 | -3.922499039 | 0.000857981 | 0.003625953 | -0.95750712 |
| PSMA6 | 1.067035534 | 12.51550439 | 3.921775135 | 0.000859426 | 0.00363119 | -0.95915229 |
| ZSWIM6 | 0.93928734 | 9.304750536 | 3.92101427 | 0.000860948 | 0.003636747 | -0.96088143 |
| EXOSC8 | 0.811733591 | 10.99602588 | 3.919523338 | 0.000863939 | 0.003648502 | -0.96426961 |
| SETD7 | 0.826515961 | 5.438094388 | 3.919022996 | 0.000864945 | 0.003651873 | -0.96540661 |
| BBS12 | 0.839761342 | 6.104725652 | 3.917509234 | 0.000867995 | 0.003663871 | -0.96884648 |
| PON2 | 1.096766765 | 7.932657996 | 3.916797456 | 0.000869433 | 0.00366906 | -0.97046387 |
| LINC00493 | 0.790689243 | 12.65325268 | 3.915594359 | 0.000871869 | 0.003678457 | -0.97319762 |
| GTF2IRD2 | 0.905897004 | 6.266223823 | 3.915380808 | 0.000872302 | 0.003678847 | -0.97368286 |
| CHPT1 | -1.087108009 | 9.997528161 | -3.915342497 | 0.000872379 | 0.003678847 | -0.97376991 |
| PGA3 | -2.121782425 | 7.151867337 | -3.915033025 | 0.000873007 | 0.003680613 | -0.97447309 |
| UBXN8 | 0.710367764 | 7.088843958 | 3.913830774 | 0.000875452 | 0.003689637 | -0.97720479 |
| SUB1 | 1.897412629 | 9.261031576 | 3.913773806 | 0.000875568 | 0.003689637 | -0.97733422 |
| GMEB1 | -0.528439089 | 9.513422144 | -3.912467431 | 0.000878231 | 0.003698802 | -0.98030239 |
| TSPYL2 | 1.25373243 | 5.163772448 | 3.912397934 | 0.000878373 | 0.003698802 | -0.98046029 |
| HNRNPL | 1.404531626 | 9.616762764 | 3.911507644 | 0.000880194 | 0.00370558 | -0.98248303 |
| HORMAD1 | 1.426293702 | 5.734439863 | 3.911301668 | 0.000880615 | 0.003706101 | -0.982951 |
| CDHR5 | -1.015449948 | 4.609273958 | -3.911221462 | 0.00088078 | 0.003706101 | -0.98313322 |
| LDHD | -1.296729859 | 7.309474801 | -3.911138455 | 0.00088095 | 0.003706101 | -0.98332181 |
| CUL9 | -0.552398177 | 11.55667139 | -3.909782982 | 0.000883731 | 0.003716122 | -0.9864013 |
| PITPNC1 | 0.88731143 | 6.659008999 | 3.9097715 | 0.000883754 | 0.003716122 | -0.98642739 |
| FAM110C | 1.093693361 | 4.363646039 | 3.909259399 | 0.000884807 | 0.00371966 | -0.98759079 |
| IER5 | 0.637313517 | 13.22361362 | 3.908770286 | 0.000885814 | 0.003723003 | -0.98870196 |
| REV1 | 1.020343989 | 7.274700699 | 3.908290948 | 0.000886802 | 0.003726264 | -0.98979091 |
| ZNF431 | 1.148556556 | 2.50203388 | 3.907207085 | 0.00088904 | 0.003734775 | -0.99225315 |
| CCDC17 | -0.737720456 | 5.179549314 | -3.906815251 | 0.00088985 | 0.003737287 | -0.99314326 |
| RPS6 | 0.821063819 | 15.52017735 | 3.906351693 | 0.00089081 | 0.003740424 | -0.9941963 |
| UBA1 | -0.644553991 | 10.77771795 | -3.906238922 | 0.000891044 | 0.003740512 | -0.99445248 |
| BAD | -0.773931213 | 9.10070267 | -3.905947856 | 0.000891647 | 0.003742151 | -0.99511367 |
| HIVEP2 | 0.711409618 | 11.03477011 | 3.905526063 | 0.000892522 | 0.003744929 | -0.99607181 |
| CDK5RAP2 | -0.698843423 | 9.824652613 | -3.90537183 | 0.000892842 | 0.003745379 | -0.99642216 |
| PDCD4 | 0.588535571 | 9.77607004 | 3.905218543 | 0.00089316 | 0.003745821 | -0.99677036 |
| INVS | 0.747183989 | 7.239337702 | 3.904956828 | 0.000893704 | 0.003747207 | -0.99736486 |
| LRRN2 | -1.214006886 | 7.793547561 | -3.904691835 | 0.000894255 | 0.003748623 | -0.9979668 |
| XPO4 | 0.655027057 | 7.318806045 | 3.904079595 | 0.000895529 | 0.003753069 | -0.9993575 |
| WFDC1 | -0.558821786 | 2.865889235 | -3.903865913 | 0.000895974 | 0.003754039 | -0.99984287 |
| PPP3CA | 0.621944608 | 10.60298582 | 3.902629744 | 0.000898553 | 0.003763948 | -1.00265074 |
| MRS2 | 0.672705559 | 7.683541827 | 3.902357067 | 0.000899123 | 0.003765439 | -1.0032701 |
| HRSP12 | 1.321668221 | 6.599191905 | 3.901193203 | 0.000901559 | 0.003774123 | -1.00591362 |
| LRRC17 | 1.142761271 | 3.014428828 | 3.901161589 | 0.000901626 | 0.003774123 | -1.00598542 |
| SLC35F2 | 0.565329316 | 7.476468081 | 3.899942779 | 0.000904185 | 0.003783933 | -1.00875364 |
| ANKRD20A2 | -1.039079859 | 7.358871467 | -3.898378658 | 0.000907479 | 0.003796531 | -1.01230601 |
| SLC2A3 | 1.117745244 | 7.855300421 | 3.898308521 | 0.000907627 | 0.003796531 | -1.01246529 |
| VPS26A | 0.630382737 | 9.524651279 | 3.89807984 | 0.00090811 | 0.003797647 | -1.01298465 |
| PLGLB1 | 0.845283503 | 5.517037168 | 3.894787511 | 0.000915088 | 0.00382592 | -1.02046145 |
| UCK1 | -0.605324995 | 8.592201793 | -3.894080973 | 0.000916592 | 0.003831299 | -1.02206588 |
| AGPAT2 | -0.653021187 | 13.66464715 | -3.893538381 | 0.000917749 | 0.003835224 | -1.023298 |
| CMTM1 | -0.667012572 | 7.870160108 | -3.892678829 | 0.000919585 | 0.003841983 | -1.02524983 |
| LHFPL3 | 1.976870206 | 3.241778166 | 3.892467723 | 0.000920037 | 0.00384263 | -1.02572919 |
| HHEX | 0.622257469 | 9.452021758 | 3.892331909 | 0.000920327 | 0.00384263 | -1.02603759 |
| PRDM8 | 0.967637418 | 8.179699712 | 3.89229992 | 0.000920396 | 0.00384263 | -1.02611022 |
| IQGAP1 | 0.605235826 | 13.894899 | 3.891934183 | 0.000921179 | 0.003844986 | -1.0269407 |
| GPR45 | -1.576984677 | 5.667996575 | -3.891419169 | 0.000922282 | 0.003848679 | -1.02811012 |
| LARP7 | 1.139705459 | 9.256296306 | 3.887629242 | 0.000930445 | 0.00388182 | -1.03671518 |
| MADD | -0.647668169 | 9.905389138 | -3.886927083 | 0.000931965 | 0.003887221 | -1.03830933 |
| VWA2 | -1.660335797 | 6.216317492 | -3.886827154 | 0.000932181 | 0.003887221 | -1.03853621 |
| OR8U1 | -1.88958331 | 6.093959611 | -3.886649177 | 0.000932567 | 0.003887907 | -1.03894027 |
| PAQR9 | -1.670053158 | 4.041087791 | -3.886352374 | 0.000933211 | 0.003889607 | -1.0396141 |
| PEX12 | 0.624744249 | 7.595161955 | 3.886257275 | 0.000933417 | 0.003889607 | -1.03983001 |
| SMURF1 | 1.216425802 | 3.22189686 | 3.885979171 | 0.000934021 | 0.003891201 | -1.04046138 |
| ITPR2 | 1.166381682 | 8.334625644 | 3.88509086 | 0.000935952 | 0.003898321 | -1.04247806 |
| DONSON | 0.843507168 | 5.090397304 | 3.884920514 | 0.000936323 | 0.003898942 | -1.04286477 |
| DGKZ | -0.638379975 | 13.21537323 | -3.88426784 | 0.000937744 | 0.003903938 | -1.04434646 |
| PAG1 | 0.746596202 | 10.29468414 | 3.884095542 | 0.00093812 | 0.003904578 | -1.0447376 |
| RAB36 | -0.765892185 | 8.310789244 | -3.883443077 | 0.000939544 | 0.00390958 | -1.04621877 |
| ACSBG1 | -1.117813034 | 4.516514578 | -3.883087043 | 0.000940322 | 0.003911277 | -1.047027 |
| EIF5A2 | 0.600996059 | 7.758380932 | 3.88305288 | 0.000940397 | 0.003911277 | -1.04710455 |
| TMEM89 | -0.808255967 | 5.260908422 | -3.882346941 | 0.000941941 | 0.003916775 | -1.04870706 |
| RPL34 | 2.349562947 | 13.92885345 | 3.880500073 | 0.000945994 | 0.003932697 | -1.05289936 |
| RGMB | -1.321695348 | 7.419029323 | -3.880352224 | 0.00094632 | 0.00393312 | -1.05323496 |
| C15orf62 | -1.692006626 | 6.982196606 | -3.880062191 | 0.000946958 | 0.003934842 | -1.0538933 |
| MED7 | 1.211208691 | 7.301605561 | 3.879265268 | 0.000948714 | 0.003941208 | -1.05570218 |
| FAM3D | -1.21728118 | 6.220113349 | -3.878824654 | 0.000949686 | 0.003943762 | -1.05670228 |
| ZNF33B | 0.680413878 | 9.957743826 | 3.878783372 | 0.000949777 | 0.003943762 | -1.05679598 |
| SLC38A7 | -0.675067472 | 9.241630718 | -3.878635656 | 0.000950103 | 0.003944132 | -1.05713126 |
| VIT | -1.484781493 | 5.851039812 | -3.878539868 | 0.000950315 | 0.003944132 | -1.05734868 |
| SNPH | -1.000931467 | 8.244703063 | -3.87777054 | 0.000952016 | 0.00395026 | -1.05909484 |
| GAN | 1.891102329 | 5.944497763 | 3.877533782 | 0.00095254 | 0.003951502 | -1.05963221 |
| DNAJC22 | -2.459228748 | 5.536812176 | -3.876990025 | 0.000953745 | 0.003955567 | -1.06086635 |
| C9orf78 | -1.331862409 | 12.27373562 | -3.876636079 | 0.00095453 | 0.003957807 | -1.06166968 |
| ZNF461 | 0.931906348 | 5.611862741 | 3.876543708 | 0.000954735 | 0.003957807 | -1.06187933 |
| CDC37L1 | 1.119890487 | 7.221607994 | 3.875483584 | 0.000957091 | 0.003966296 | -1.06428536 |
| RAP1GAP2 | -0.867100638 | 6.515919711 | -3.875419205 | 0.000957234 | 0.003966296 | -1.06443147 |
| FAM131C | -2.659023939 | 12.22926331 | -3.875182664 | 0.00095776 | 0.003967543 | -1.06496831 |
| FCN3 | -1.56909347 | 6.876390825 | -3.8742168 | 0.000959913 | 0.003975524 | -1.06716032 |
| SLC29A1 | -1.08434506 | 9.009597937 | -3.873738828 | 0.00096098 | 0.003978403 | -1.06824504 |
| C1D | 1.056808369 | 8.572523764 | 3.873702775 | 0.000961061 | 0.003978403 | -1.06832686 |
| PTGER3 | -0.998754255 | 4.535613693 | -3.871677335 | 0.000965597 | 0.003996238 | -1.07292328 |
| SLC19A3 | -1.239006968 | 7.318474978 | -3.871503494 | 0.000965987 | 0.003996912 | -1.07331777 |
| KIF5C | 1.170675659 | 6.564377522 | 3.870761725 | 0.000967654 | 0.004002868 | -1.07500101 |
| TNNI3 | -1.160598049 | 5.425230014 | -3.86898604 | 0.000971656 | 0.004018479 | -1.07903029 |
| FAM71E2 | -1.284061978 | 6.938807798 | -3.868560793 | 0.000972617 | 0.004021507 | -1.0799952 |
| ACRC | 0.831881425 | 8.644400257 | 3.867565237 | 0.00097487 | 0.004029876 | -1.08225413 |
| SESN1 | 1.171134145 | 7.721745163 | 3.866773876 | 0.000976665 | 0.004036347 | -1.08404969 |
| SAV1 | 0.648868966 | 7.831218428 | 3.866390784 | 0.000977535 | 0.004038994 | -1.08491889 |
| NRXN1 | 1.064043839 | 3.091872757 | 3.865746018 | 0.000979001 | 0.004044101 | -1.08638178 |
| RALGPS2 | 1.084519143 | 5.889959163 | 3.865211376 | 0.000980219 | 0.004048179 | -1.08759479 |
| RASGRP3 | 1.347546364 | 6.515002095 | 3.864310407 | 0.000982274 | 0.004055713 | -1.08963888 |
| ZNF705A | -1.003999202 | 3.215443387 | -3.863640232 | 0.000983805 | 0.004061082 | -1.09115932 |
| CCL14 | -1.600967088 | 3.318530087 | -3.863535778 | 0.000984044 | 0.004061115 | -1.0913963 |
| MMP28 | -1.052136715 | 8.029251308 | -3.862312382 | 0.000986846 | 0.004070768 | -1.09417174 |
| RGS4 | 0.980746215 | 14.88560516 | 3.861827183 | 0.000987959 | 0.004074406 | -1.09527245 |
| MYO3A | 2.275008376 | 4.619982191 | 3.861346939 | 0.000989063 | 0.004078 | -1.09636191 |
| KHDRBS2 | 2.154583079 | 4.23913912 | 3.860219141 | 0.000991659 | 0.004087236 | -1.0989203 |
| CSTF3 | 0.613175228 | 8.013489638 | 3.860171862 | 0.000991768 | 0.004087236 | -1.09902755 |
| ZFAND4 | 0.961027302 | 6.156255757 | 3.859193015 | 0.000994027 | 0.004095585 | -1.10124797 |
| TSPYL5 | 1.153638585 | 5.043792366 | 3.857885362 | 0.000997052 | 0.004107089 | -1.10421415 |
| OS9 | -0.524119756 | 12.72625035 | -3.857406668 | 0.000998162 | 0.004110698 | -1.10529995 |
| ZNF155 | 0.608182705 | 5.917405851 | 3.857016298 | 0.000999068 | 0.004112625 | -1.1061854 |
| GCC1 | -0.694582204 | 9.514431162 | -3.857003615 | 0.000999098 | 0.004112625 | -1.10621417 |
| C3orf58 | 1.202970187 | 6.240523578 | 3.855480905 | 0.00100264 | 0.00412593 | -1.10966791 |
| CNTRL | 0.753057304 | 7.600208444 | 3.855332121 | 0.001002986 | 0.00412593 | -1.11000536 |
| ZNF131 | 0.507731567 | 10.05925609 | 3.855311777 | 0.001003034 | 0.00412593 | -1.1100515 |
| ZBTB33 | 0.819783362 | 8.261632674 | 3.854300889 | 0.001005393 | 0.004134667 | -1.11234425 |
| SOCS7 | 1.532360641 | 4.041086229 | 3.854111505 | 0.001005836 | 0.004135521 | -1.11277377 |
| AK7 | -0.715669941 | 2.768348552 | -3.853891823 | 0.001006349 | 0.004136665 | -1.113272 |
| AKAP9 | 0.580908683 | 8.955244907 | 3.853703091 | 0.001006791 | 0.004137513 | -1.11370004 |
| OR1C1 | -1.19228784 | 3.043322283 | -3.853496825 | 0.001007274 | 0.00413853 | -1.11416785 |
| RNF112 | -0.684188582 | 7.047107141 | -3.853393536 | 0.001007515 | 0.004138556 | -1.1144021 |
| SLC22A14 | -1.590704777 | 6.972426069 | -3.853157423 | 0.001008068 | 0.004139861 | -1.11493759 |
| LRBA | 0.702151457 | 8.202025495 | 3.851693717 | 0.001011503 | 0.004152997 | -1.11825709 |
| SHARPIN | -0.727504882 | 12.23651826 | -3.850898273 | 0.001013375 | 0.00415971 | -1.12006098 |
| RRAGB | 1.335565543 | 4.476755501 | 3.850751336 | 0.001013721 | 0.00416016 | -1.1203942 |
| ZNF543 | 0.727555873 | 6.692774419 | 3.850271277 | 0.001014853 | 0.004163832 | -1.12148284 |
| QPCTL | 1.247667232 | 3.496468993 | 3.850085606 | 0.001015291 | 0.004164147 | -1.12190389 |
| DDX1 | 0.727243465 | 11.27671687 | 3.850037982 | 0.001015403 | 0.004164147 | -1.12201189 |
| MARVELD1 | -0.762628569 | 10.95990006 | -3.849667022 | 0.001016279 | 0.004166767 | -1.1228531 |
| IFRD1 | 0.959972898 | 9.33571025 | 3.849416386 | 0.001016871 | 0.004168223 | -1.12342146 |
| MSC | 1.235554621 | 2.841238423 | 3.849155086 | 0.001017489 | 0.004169783 | -1.12401399 |
| ODF3B | -0.896736867 | 11.40404243 | -3.84900603 | 0.001017841 | 0.004170255 | -1.124352 |
| CDC16 | 0.599262319 | 11.83136815 | 3.848761467 | 0.00101842 | 0.004171481 | -1.12490657 |
| RCN1 | 0.843088193 | 8.291085632 | 3.848679139 | 0.001018615 | 0.004171481 | -1.12509326 |
| IL15 | 1.06594467 | 8.918437059 | 3.846939261 | 0.001022741 | 0.004187406 | -1.12903848 |
| NOVA2 | -1.380527364 | 3.85886088 | -3.846636287 | 0.001023462 | 0.00418938 | -1.12972545 |
| TMED4 | 0.738928822 | 9.524529313 | 3.846052456 | 0.001024851 | 0.004194091 | -1.13104924 |
| SH3BGRL3 | -0.672762931 | 14.42563432 | -3.845927877 | 0.001025148 | 0.00419433 | -1.13133171 |
| ZNF322 | 0.632222736 | 8.86650367 | 3.8457292 | 0.001025621 | 0.00419529 | -1.13178219 |
| SGPP2 | 1.141022549 | 4.886625565 | 3.844405404 | 0.001028781 | 0.004207237 | -1.13478365 |
| UBAC1 | -0.75894171 | 10.87632362 | -3.844088887 | 0.001029538 | 0.004208376 | -1.13550128 |
| LCE1E | -1.963290536 | 7.671103417 | -3.843943909 | 0.001029885 | 0.004208815 | -1.13582998 |
| DEDD2 | -0.999376276 | 11.94884831 | -3.843821389 | 0.001030179 | 0.004209036 | -1.13610776 |
| TRIM21 | -0.727554216 | 9.473771947 | -3.842771651 | 0.001032695 | 0.004218335 | -1.13848771 |
| SETD1A | 0.530135026 | 10.52821914 | 3.842234946 | 0.001033983 | 0.004222619 | -1.13970449 |
| LGR4 | 0.903126514 | 4.197986274 | 3.841073038 | 0.001036779 | 0.004233052 | -1.1423386 |
| KRT1 | -2.131654969 | 13.12400142 | -3.840916713 | 0.001037155 | 0.004233607 | -1.14269299 |
| SEMA4D | -0.693291849 | 11.28633514 | -3.840570731 | 0.00103799 | 0.004236028 | -1.14347733 |
| METTL23 | 0.547094546 | 11.03388205 | 3.838706081 | 0.001042497 | 0.004253435 | -1.14770432 |
| PLA2G5 | -2.344795359 | 6.078616871 | -3.838314522 | 0.001043446 | 0.004255332 | -1.14859191 |
| SLC39A6 | 1.412658353 | 5.202396889 | 3.837955129 | 0.001044317 | 0.0042579 | -1.14940658 |
| ZNF302 | 0.790373862 | 9.576167098 | 3.837266718 | 0.001045989 | 0.004263727 | -1.15096704 |
| UST | 0.925810636 | 5.174899914 | 3.836636598 | 0.001047522 | 0.004268793 | -1.15239533 |
| ANKRD50 | 0.980179696 | 4.333909042 | 3.836500731 | 0.001047853 | 0.004268793 | -1.1527033 |
| NAA20 | 0.702554781 | 8.854144379 | 3.836456499 | 0.00104796 | 0.004268793 | -1.15280356 |
| SLC9A7 | 1.243499967 | 7.120135796 | 3.833782909 | 0.001054491 | 0.004294399 | -1.15886341 |
| STX1A | -0.919582912 | 7.449591274 | -3.833432671 | 0.001055349 | 0.0042969 | -1.1596572 |
| NDUFAF3 | -0.559321234 | 14.58835247 | -3.833177321 | 0.001055975 | 0.004298455 | -1.16023593 |
| SPI1 | -0.604431773 | 14.05691641 | -3.832296328 | 0.001058139 | 0.004306266 | -1.16223259 |
| GAS2 | 0.921087541 | 2.553131322 | 3.831046872 | 0.001061215 | 0.004316897 | -1.16506423 |
| MX2 | -0.765006664 | 12.28548344 | -3.831035948 | 0.001061242 | 0.004316897 | -1.16508898 |
| SLC26A1 | 0.704280004 | 5.267742816 | 3.829823437 | 0.001064236 | 0.004328074 | -1.16783677 |
| NFKB2 | -0.784548287 | 8.411250732 | -3.829276731 | 0.001065589 | 0.004332573 | -1.16907567 |
| YPEL3 | -0.934993216 | 11.85445608 | -3.828764146 | 0.001066859 | 0.004336733 | -1.17023723 |
| ZNF397 | 0.532232419 | 8.36583553 | 3.827670693 | 0.001069572 | 0.004346759 | -1.17271502 |
| ZNF517 | -1.516529194 | 3.705312752 | -3.826893534 | 0.001071505 | 0.004352603 | -1.17447602 |
| MASTL | 0.858643291 | 6.785151937 | 3.826408468 | 0.001072713 | 0.004356505 | -1.17557513 |
| FBXO9 | -0.68104443 | 12.78069672 | -3.825974399 | 0.001073796 | 0.004359893 | -1.17655868 |
| RPAP1 | -0.535856366 | 10.44039784 | -3.824631777 | 0.00107715 | 0.004372504 | -1.17960078 |
| ZDHHC3 | -0.61604392 | 11.06733399 | -3.823555859 | 0.001079846 | 0.004382436 | -1.18203848 |
| UBE2C | -0.884842232 | 7.823259487 | -3.823111477 | 0.001080961 | 0.00438595 | -1.18304529 |
| CA1 | -2.286760098 | 12.73741966 | -3.822291205 | 0.001083023 | 0.004392343 | -1.18490369 |
| PRKCZ | -0.764388195 | 11.54794068 | -3.822285914 | 0.001083037 | 0.004392343 | -1.18491568 |
| SCRIB | -0.519316325 | 12.05951727 | -3.8219582 | 0.001083861 | 0.004394675 | -1.18565812 |
| RAPH1 | -1.882026307 | 10.33243989 | -3.821690369 | 0.001084536 | 0.004396396 | -1.1862649 |
| RNF144A | 1.05352075 | 5.679942614 | 3.821467187 | 0.001085099 | 0.004397663 | -1.18677052 |
| SLC24A2 | -0.738261658 | 3.93234477 | -3.821345935 | 0.001085404 | 0.004397888 | -1.18704521 |
| SPAG16 | 1.16649045 | 5.175336782 | 3.819671496 | 0.001089634 | 0.004411978 | -1.19083851 |
| PTPN4 | 0.953720732 | 8.457705458 | 3.819328497 | 0.001090503 | 0.004413968 | -1.19161551 |
| CLRN2 | -1.448205956 | 3.76549241 | -3.819279277 | 0.001090628 | 0.004413968 | -1.19172701 |
| KRCC1 | 1.235137231 | 8.667420049 | 3.819018517 | 0.001091289 | 0.004415626 | -1.19231771 |
| ROCK2 | 0.872103096 | 6.812087512 | 3.818683763 | 0.001092138 | 0.004418044 | -1.19307602 |
| OSBPL2 | -0.501574403 | 10.16009347 | -3.818266518 | 0.001093197 | 0.004421069 | -1.19402119 |
| RBM15 | 0.929037787 | 7.260492267 | 3.818191161 | 0.001093388 | 0.004421069 | -1.19419189 |
| SRGAP1 | -1.156277994 | 3.69445204 | -3.817551018 | 0.001095015 | 0.00442488 | -1.19564194 |
| AHR | 1.202965369 | 8.77303694 | 3.817526367 | 0.001095078 | 0.00442488 | -1.19569778 |
| SERPINA3 | -0.919720748 | 5.918639557 | -3.81752334 | 0.001095086 | 0.00442488 | -1.19570464 |
| LRMP | 0.929032749 | 11.99363487 | 3.816361503 | 0.001098045 | 0.00443582 | -1.19833634 |
| DIDO1 | 0.593012566 | 8.672566852 | 3.815929416 | 0.001099148 | 0.004439254 | -1.19931505 |
| SOS2 | 0.879825761 | 9.018439809 | 3.815525673 | 0.001100179 | 0.004442399 | -1.20022954 |
| H2AFY2 | -0.996363289 | 6.232153781 | -3.815154576 | 0.001101128 | 0.004444359 | -1.20107007 |
| ZG16 | -1.182830635 | 3.516016554 | -3.815004108 | 0.001101513 | 0.004444359 | -1.20141088 |
| C17orf50 | -1.214612184 | 6.13293223 | -3.814986058 | 0.001101559 | 0.004444359 | -1.20145176 |
| MRI1 | 0.881750537 | 7.133900608 | 3.814873824 | 0.001101846 | 0.004444359 | -1.20170596 |
| TFAP4 | 1.164827256 | 2.838137781 | 3.81477055 | 0.001102111 | 0.004444359 | -1.20193988 |
| LHB | -2.16938985 | 8.948666967 | -3.814743072 | 0.001102181 | 0.004444359 | -1.20200211 |
| USP20 | -0.683527055 | 10.70423184 | -3.813665268 | 0.001104944 | 0.004454125 | -1.20444324 |
| SLC22A17 | -1.842404002 | 7.710172829 | -3.813570519 | 0.001105187 | 0.004454125 | -1.20465783 |
| IRF9 | -0.54477768 | 11.53093802 | -3.813502132 | 0.001105363 | 0.004454125 | -1.20481272 |
| ABHD4 | -0.786424161 | 10.08865807 | -3.813355374 | 0.001105739 | 0.004454623 | -1.2051451 |
| ZBTB25 | 0.922985697 | 7.78065041 | 3.81290628 | 0.001106894 | 0.004458251 | -1.20616221 |
| LACC1 | 1.201121065 | 4.188568816 | 3.812199649 | 0.001108712 | 0.004464552 | -1.20776257 |
| MAP1LC3B | -0.666123283 | 11.21575422 | -3.811156782 | 0.001111401 | 0.004474355 | -1.21012435 |
| STAC3 | -0.957049373 | 10.1869096 | -3.811050477 | 0.001111675 | 0.004474436 | -1.2103651 |
| ZBED2 | 1.240830955 | 3.728775897 | 3.810949756 | 0.001111935 | 0.004474459 | -1.21059319 |
| STAC2 | -1.212185634 | 5.573521501 | -3.810362055 | 0.001113454 | 0.004479546 | -1.21192411 |
| SLC25A38 | -0.87798681 | 9.434749991 | -3.808932147 | 0.001117159 | 0.00449342 | -1.21516219 |
| FMNL1 | -0.740355957 | 10.26816151 | -3.808407936 | 0.00111852 | 0.004497312 | -1.21634924 |
| STOML1 | -0.700589192 | 10.35928645 | -3.80836252 | 0.001118638 | 0.004497312 | -1.21645208 |
| ARF6 | 1.043612055 | 8.28722779 | 3.808025404 | 0.001119514 | 0.004499806 | -1.21721545 |
| TFB2M | 0.80533208 | 8.872637179 | 3.80752388 | 0.001120819 | 0.004504021 | -1.21835109 |
| BATF2 | -1.826554882 | 9.504162682 | -3.807330639 | 0.001121322 | 0.004504317 | -1.21878866 |
| TBCA | 1.115764509 | 11.43839123 | 3.807298852 | 0.001121405 | 0.004504317 | -1.21886064 |
| CYP4A22 | -1.580023315 | 4.108427428 | -3.806741657 | 0.001122857 | 0.00450912 | -1.2201223 |
| CEP41 | 0.921962046 | 5.796176804 | 3.806017253 | 0.001124748 | 0.004515682 | -1.22176255 |
[truncated: 545,455 more chars]
